# Tracking U.S. Pertussis Incidence: Correlation of Public Health Surveillance and Google Search Data Varies by State

**DOI:** 10.1038/s41598-019-56385-z

**Published:** 2019-12-24

**Authors:** Christopher H. Arehart, Michael Z. David, Vanja Dukic

**Affiliations:** 1 Department of Applied Mathematics, University of Colorado Boulder, Boulder, Colorado 80309 United States of America; 2 Division of Infectious Diseases, Department of Medicine, University of Pennsylvania, Philadelphia, Pennsylvania 19104 United States of America

**Keywords:** Bacterial infection, Disease prevention, Epidemiology

## Abstract

The Morbidity and Mortality Weekly Reports of the U.S. Centers for Disease Control and Prevention document a raw proxy for counts of pertussis cases in the U.S., and the Project Tycho (PT) database provides an improved source of these weekly data. These data are limited because of reporting delays, variation in state-level surveillance practices, and changes over time in diagnosis methods. We aim to assess whether Google Trends (GT) search data track pertussis incidence relative to PT data and if sociodemographic characteristics explain some variation in the accuracy of state-level models. GT and PT data were used to construct auto-correlation corrected linear models for pertussis incidence in 2004–2011 for the entire U.S. and each individual state. The national model resulted in a moderate correlation (adjusted R^2^ = 0.2369, p < 0.05), and state models tracked PT data for some but not all states. Sociodemographic variables explained approximately 30% of the variation in performance of individual state-level models. The significant correlation between GT models and public health data suggests that GT is a potentially useful pertussis surveillance tool. However, the variable accuracy of this tool by state suggests GT surveillance cannot be applied in a uniform manner across geographic sub-regions.

## Introduction

**Figure 1:**
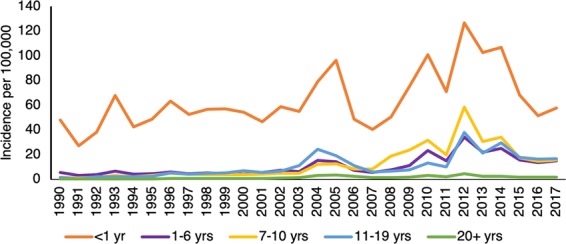
U.S. time-series showing pertussis incidence per 100,000 people categorized by age group for 1990–2017. After introduction of an acellular vaccine, there was an increase in incidence among school age and adolescent age groups. Data from the National Notifiable Diseases Surveillance System^9^.

Pertussis is a human infectious disease that is caused by the bacterial species *Bordetella pertussis* and is transmitted from person to person through sneezing or coughing. Also known as whooping cough, pertussis is highly contagious, and people of all ages worldwide are at risk^1^. Infected individuals tend to show symptoms within 7 to 10 days after exposure and are most likely to infect others during the first three weeks of coughing. Children under 6 months of age are especially susceptible to the severe and sometimes life-threatening disease. Before a vaccine successfully reduced incidence rates in the United States (U.S.), over one million cases of pertussis were reported in the early 1940’s^2^. The effectiveness of the vaccine, however, has not been universal as immunity often wanes over time and vaccinated individuals can asymptomatically transmit pertussis to naïve hosts^3,4^. Recently, the number of cases of pertussis has increased^5^, and epidemic outbreaks have been recorded in some U.S. states. In 2012 alone, there were 48,277 cases reported in the U.S.^2^, and in 2014 pertussis ranked as the deadliest vaccine-preventable disease^1^. While acellular vaccines are safer than whole-cell vaccines, the move toward an acellular pertussis vaccine in the 1990’s resulted in a faster rate of waning immunity^6–8^. Over the past decade, this waning of immunity (estimated between 5–8 years) is believed to be responsible for the recent shift toward increased pertussis incidence in children in older age groups (Fig. 1)^9,10^.

The U.S. Centers for Disease Control and Prevention’s (CDC) Morbidity and Mortality Weekly Reports (MMWR) are the best available raw proxy for weekly counts of pertussis cases in the U.S. However, there are several factors that affect the accuracy of the MMWR data, and which may interfere with public health interventions to curtail the spread of this disease. For example, MMWR surveillance data are impacted by variable reporting lags in different states and members of the reporting network; the median national reporting delay for a pertussis case in 2004 was 40 days from the date of onset^11^. Coughing in pertussis can last for many weeks, resulting in late diagnosis, and this lag can also affect the accuracy of surveillance data. The MMWR-defined reporting week may reflect the week the report was submitted to the CDC, the week of pertussis onset, or the week of laboratory diagnosis, and thus is often misaligned with the time of the patient’s illness. In addition, disease surveillance and reporting practices can vary among states due to differences in reporting systems^12^. Disease surveillance in the U.S. is organized at the regional level, and the federal government has limited ability to standardize state reporting methods. Surveillance capacity also depends on available funding which varies by state^13^.

A change in diagnostic methods for pertussis from clinical diagnosis and serological testing to polymerase chain reaction [PCR]-based testing further complicates the interpretation and standardization of time-series data from the past decade. Clinical criteria may lack specificity and sensitivity; and, while PCR test platforms can rapidly and accurately identify *Bordetella* in clinical specimens using bacterial DNA target sequences, they can sometimes generate false-negative and false-positive results^14^. For these reasons, reports on disease incidence now include some cases identified by culture, PCR, serology, or clinical diagnosis^15^. The discrepancies related to reporting delays, state reporting practices, and changing diagnostic methods for pertussis make it challenging to define a consistent and optimal time-series to track pertussis at the national and state levels.

To address some of the problems with the quality of CDC surveillance data, additional sources of data may be usefully incorporated into the national surveillance system. Internet Protocol (IP) surveillance, which uses internet search data to track disease incidence, may help to improve both the accuracy and timeliness of disease reporting. Among IP surveillance tools, Google Trends (GT), Google’s anonymized repository of data on the popularity of Google search queries across geographic regions and timespans^16^, has become a resource for what has been termed “computational epidemiology”^17^ and sometimes “infodemiology”^18^. Google Trends data have been used to predict incidence of many infectious diseases, ranging from influenza to Lyme disease^19–22^.

Operating on the hypothesis that individuals who are infected by, exposed to, or treating a disease tend to use Google to search for disease-related terms, studies have demonstrated the potential of using GT to track pertussis outbreaks in California^23^ and in Australia^24^. In this paper, we focus on a gap in IP epidemiology research (addressed in Ricketts & Silva^25^) through state-level modeling. This analysis is designed to elaborate on variations in model accuracy by exploring if there are socio-demographic differences between states that allow for some states’ models to perform better than others. Because individual states vary significantly in their health care infrastructures and health outcomes^26^, we included state-level characteristics in our IP surveillance analyses.

More specifically, the present study aims to (1) investigate the feasibility of using GT to monitor pertussis at the national level in the U.S., (2) explore differences in these models at the state level, and (3) assess which state-specific sociodemographic variables influence the accuracy of these state-based GT models. IP surveillance using GT at the state level may improve its utility as a public health tool, potentially informing local policy makers and alerting public health officials of pertussis outbreaks in real time.

## Methods

Project Tycho (PT) originated at the University of Pittsburgh as an effort to improve standards, machine readability, and availability of health data^27^. As a part of that effort, historical U.S. surveillance data for eight childhood diseases, including pertussis, have been made available to researchers. The PT Level 1 archive provides an alternative, cleaner source for pertussis surveillance data that is more complete than MMWR reports. For this study, we used the publicly available PT Level 1 pertussis data and received permissions to extract GT data from Google’s Application Program Interface (API). PT and GT incidence trends were obtained for the U.S. overall as well as for each of the 50 U.S. states and the District of Columbia (DC) individually. We thus analyzed a total of 52 geographic regions. We studied data from 2004–2011, a period encompassing the overlap of GT data, which began in 2004 and PT Level 1 data, which ended in 2011. All GT and PT data were anonymized, and thus no Institutional Review Board approval was necessary for this project.

### Google Trends (GT) data

In order to extract the most informative GT search terms, we developed a broad list of pertussis-related key words and phrases, shown in Table 1. This list was derived from the search terms used in prior literature^23,24^, Google Correlate, and terms found on popular health information websites for the general public, including common misspellings.

**Table 1:** Terms describing selection of the 14 GT searches used for modeling incidence.

	Preliminary Word Bank	Source	Final Word Bank	Exclusion Justification
1	“bordatella”	health information websites	yes	
2	“bordetella”	common misspelling	yes	
3	“CDC pertussis”	Pollet *et al*., 2015	no	https://trends.google.com/trends/explore?geo = US-CA&q = %22CDC%20pertussis%22
4	“chronic cough”	health information websites	yes	
5	“coqueluche”	Spanish term	yes	
6	“coughing fits”	health information websites	yes	
7	“coughing spell”	health information websites	no	https://trends.google.com/trends/explore?geo = US-CA&q = %22coughing%20spell%22
8	“exhaustion after cough”	health information websites	no	https://trends.google.com/trends/explore?geo = US-CA&q = %22exhaustion%20after%20cough%22
9	“infant pertussis”	health information websites	no	https://trends.google.com/trends/explore?geo = US-CA&q = %22infant%20pertussis%22
10	“infant whooping cough”	health information websites	no	https://trends.google.com/trends/explore?geo = US-CA&q = %22infant%20whooping%20cough%22
11	“pertusis”	common misspelling	yes	
12	“pertussis”	Pollet *et al*., 2015; Zhang *et al*., 2017	yes	
13	“pertussis kids”	health information websites	no	https://trends.google.com/trends/explore?geo = US-CA&q = %22pertussis%20kids%22
14	“pertussis symptoms”	Pollet *et al*., 2015	yes	
15	“pertussis treatment”	health information websites	yes	
16	“prolonged cough”	health information websites	no	https://trends.google.com/trends/explore?geo = US-CA&q = %22prolonged%20cough%22
17	“puking after cough”	health information websites	no	https://trends.google.com/trends/explore?geo = US-CA&q = %22puking%20after%20cough%22
18	“symptoms whooping cough”	Pollet *et al*., 2015	no	https://trends.google.com/trends/explore?geo = US-CA&q = %22symptoms%20whooping%20cough%22
19	“tired after cough”	health information websites	no	https://trends.google.com/trends/explore?geo = US-CA&q = %22tired%20after%20cough%22
20	“tos ferina”	Spanish term	yes	
21	“uncontrollable cough”	health information websites	no	https://trends.google.com/trends/explore?geo = US-NY&q = %22uncontrollable%20cough%22
22	“vomiting after cough”	health information websites	no	https://trends.google.com/trends/explore?geo = US-CA&q = %22vomiting%20after%20cough%22
23	“whooping cough adults”	Pollet *et al*., 2015	yes	
24	“whooping cough pertussis”	Pollet *et al*., 2015	no	https://trends.google.com/trends/explore?geo = US-CA&q = %22whooping%20cough%20pertussis%22
25	“whooping cough symptoms”	health information websites	yes	
26	“whooping cough treatment”	Pollet *et al*., 2015	yes	
27	“whooping cough”	Pollet *et al*., 2015; Zhang *et al*., 2017	yes	
28	“whooping”	Pollet *et al*., 2015; Zhang *et al*., 2017	no	Collinear with “whooping cough” https://trends.google.com/trends/explore?geo = US&q = %22whooping%20cough%22,%22whooping%22

Specific GT links are provided to illustrate how many terms in the preliminary word bank were excluded because they failed to return nonzero results above the privacy threshold – even in the most populated states such as California and New York.

To minimize noise from illnesses such as the common cold or other upper respiratory infections, less specific terms (e.g., “cough”) were not included, and quotations around each word or phrase were used to specify exact search terms. Terms that returned null results from the GT API for the majority of the 51 sub-regions were not included in the final word bank for further analysis.

When queried, the GT API returned a list of weekly time series data representing the probability of the search occurring in a short search-session (few consecutive searches), in the given geographic area and chosen timespan. Prior to delivery, this probability was then multiplied by 10 million to be more human-readable. Importantly, when receiving the GT data in this format, zeros in the time series may either indicate no volume or that there were too few distinct searches to exceed the Google privacy threshold. Probabilities from the GT API also may have varied slightly between queries because they are calculated on a random sub-sample of Google web searches that are updated daily.

### Project Tycho (PT) data

Even though the PT dataset had some inconsistencies and missing counts, as expected, it proved to be cleaner and more complete than the raw data from CDC MMWR reports. This was our motivation for using the PT Level 1 dataset rather than raw CDC MMWR reports produced by the US Nationally Notifiable Disease Surveillance System (NNDSS) as the gold standard pertussis surveillance dataset. Annual state population from the U.S. Census were used to convert weekly pertussis counts into incidence for each state-specific time series^28,29^.

### Modeling

To address multicollinearity and strong dependencies within GT search terms, we combined the search term “pertussis” time series with its common misspelling “pertusis” time series as follows:

$${{\rm{pertusispertussis}}}_{{\rm{i}}}=\frac{{{\rm{pertusis}}}_{{\rm{i}}}+{{\rm{pertussis}}}_{{\rm{i}}}}{{\rm{\max }}\,[{\rm{pertusis}}+{\rm{pertussis}}]}.$$

This was also done for “bordetella” and its common misspelling “bordatella.” Each GT time series was also normalized, so that the maximum of each term was 1. Finally, a full set of 2^*n*^−1 (where *n* represents the number of nonzero GT timeseries notated by *T*_1_…*T*_*n*_) linear models was constructed to span every possible combination of search terms for each geographic region:

$${P}_{ik}(t)={\beta }_{0}^{ik}+{\beta }_{1}^{ik}{T}_{1}(t)+\ldots +{\beta }_{m}^{ik}{T}_{m}(t).$$

P_*ik*_(*t*) notates the values predicted by the i^*th*^ model for the k^*th*^ region consisting of *m* ≤ *n* search terms. Every model was built using GT and PT data from the 1^st^ week of 2004 to the 52^nd^ week of 2010, and the last 52 weeks of 2011 were reserved for model forecast testing.

For each geographic region, these models were compared via their relative Akaike Information Criterion (AIC) and Bayesian Information Criterion (BIC) values and the models with the best values, denoted *AIC*(i_*_) and *BIC*(i_*_), were selected. AIC and BIC are widely used for statistical model selection as the model with the smallest AIC or BIC value corresponds to the most parsimonious model best supported by the data^30^. In addition, we used the models’ BIC values to compute model probabilities and perform model averaging. If we let *BIC*(i_*_) represent the smallest BIC value among the k^*th*^ region’s models *M*_1k_, *M*_2k_, …, *M*_(2_^*n*^_−1)*k*_ and ${\Delta }_{i}=BIC(i)-BIC({i}_{\ast })$ then the i^*th*^ model, M_ik_, has the probability of being the true model computed as follows:

$$P(i|y)\approx \frac{{e}^{-\frac{1}{2}{\Delta }_{i}}}{{\sum }_{j=1}^{{2}^{n}-1}-\frac{1}{2}{\Delta }_{j}}.$$

Note that these probabilities are in one-to-one correspondence with the BIC values.

The above model probabilities were then used to compute (1) the model averaged forecasts using all ${M}_{1k},{M}_{2k},\ldots ,{M}_{({2}^{n}-1)k}$ models and (2) the model averaged forecasts using only the top fraction of most probable *M*_1k_, …, *M*_*ik*_ models. The all-model average (1) was computed by

$${P}_{1k}\ast P(1|y)+{P}_{2k}\ast P(2|y)+\ldots +{P}_{({2}^{n}-1)k}\ast P({2}^{n}-1|y).$$

To find (2), the “most probable” models average, we used histograms of the posterior probabilities for each k^*th*^ region’s models to select the few *M*_1k_, …, *M*_*ik*_ models that had notably larger posterior probabilities than the rest of the models in the same group. The posterior probabilities for the subset were normalized, and the same model averaging methods were applied.

As we were analyzing data in time series, it was reasonable to expect some level of autocorrelation in the outcomes (PT pertussis counts), even after adjusting for all the time series of predictors (GT counts). Indeed, the Durbin-Watson test on each of these models revealed that autocorrelation was significantly different from 0. We corrected for autocorrelation in the models using the simple AR(1) Cochrane-Orcutt correction. Recreating the models and model averages by this iterative method returned the corrected regression estimates for each linear model and resulted in better inference and more generalizable results when using GT to predict PT data.

In the end, we had in total 6 methods for forecasting the reserved 52 weeks of PT testing data: *AIC*(i_*_), All Models Average, Top Models Average, AR(1) *AIC*(i_*_), AR(1) All Models Average, and AR(1) Top Models Average. For each method we computed the root-mean-square error (RMSE) between the 52-week predicted model forecasting and the observed weekly PT incidence rates during those 12 months. A lower RMSE indicates a better forecast.

### Model evaluation and sociodemographic differences between states

Using the above RMSE as the outcome, we assessed which sociodemographic variables may influence the accuracy of the best state-based GT model predictions. The sociodemographic variables examined for each of the 50 states and the District of Colombia are shown in Table 2.

**Table 2:** The states’ sociodemographic abbreviated variable names, data sources, and descriptions used in the explanatory linear model.

Variable Name	Data Source	Description
ACEP	2014 American College of Emergency Physicians (ACEP) Report Card	Scores based on access to care, quality of patient safety, public health, medical liability, disaster preparedness
Age	2010 Census	Percent of population between 20–49 years of age
Poverty	2010 Census	Percent of population in poverty
Internet	2010 Census	Percent of individuals living in a household with internet access
Education	2010 Census	Percent of population with bachelor’s degree or higher
Urban	2010 Census	Percent of individuals living in urban areas
Vaccinated	2014 CDC Childhood Diphtheria toxoid, Tetanus toxoid, acellular Pertussis (DTaP) Vaccination Coverage Report	Percent DTaP vaccination coverage among children aged 19–35 months
Republican	Federal Elections 2012: Election Results for the U.S. President, the U.S. Senate, and the U.S. House of Representatives	Percent of people who voted for Mitt Romney (Republican) in the 2012 presidential election
Job	U.S. Department of Labor, Bureau of Labor Statistics: May 2017 State Occupational Employment and Wage Estimates	Percent of employed population working in Healthcare Practitioners/Technical Occupations and Healthcare Support Occupations (occupation codes 29–0000 and 31–0000)
Population	US Census Bureau Annual Estimates of the Resident Population	2010 census population
Household	2010 Census	Average number of individuals per household
Birth	2010 CDC births by race of mother, United States, each state and territory	Births per 100,000 individuals
Immigration	Department of Homeland Security: Persons Obtaining Lawful Permanent Resident Status by State or Territory Of Residence: Fiscal Year 2012	Number of people obtaining permanent residence in the United States.

We hypothesized that sociodemographic variables may have accounted for the variability in how closely state models tracked the PT data. We thus sought to examine whether the variation in model accuracy among states could be explained by state-specific differences including, but not limited to (see Table 2), vaccination rates, American College of Emergency Physicians (ACEP) grades, educational attainment, percent of population working in healthcare, internet access, age, urbanization, political preferences, birth rates, and number of new permanent residents in 2012. An exploratory model was constructed by standardizing each sociodemographic variable before including it as a predictor in the following linear model:

$$\begin{array}{ccc}RMSE(k) & = & {\beta }_{0}+{\beta }_{1}ACEP(k)+{\beta }_{2}age(k)+{\beta }_{3}poverty(k)+{\beta }_{4}internet(k)\\  &  & +\,{\beta }_{5}education(k)+{\beta }_{6}urban(k)+{\beta }_{7}vaccinated(k)+{\beta }_{8}republican(k)\\  &  & +\,{\beta }_{9}job(k)+{\beta }_{10}population(k)+{\beta }_{11}household(k)+{\beta }_{12}birth(k)\\  &  & +\,{\beta }_{13}immigration(k).\end{array}.$$

### Additional comments

The Orcutt R package was very helpful for applying the AR(1) Cochrane-Orcutt correction, however the predict.orcutt method does not include rho*residual in the predict function and does not allow for forecasting of new data. Please visit the supplemental materials for our analysis methods regarding the AR(1) model predictions.

## Results

The six tested GT models significantly tracked with the PT data for the overall U.S. models. However, it was difficult to select a single optimal model, or even the model average. AIC and BIC (for both uncorrected and corrected versions of the models) yielded remarkably similar models. In addition, as described in Table 3, there was little variation between the RMSE values and the adjusted R^2^ values for each state’s 6 forecasting models. For that reason, below we present results for each geographic region’s AR(1) *AIC*(i_*_) model (the lowest AIC autocorrelation corrected model) which allowed us to correct for autocorrelation and to explore the variability between the search terms included in each state’s best model. As expected, the variation in the search term composition was slightly richer with AIC selectors, since AIC tends to favor slightly larger models than BIC.

**Table 3:** Modeling results for each method described by the 52-week forecasting RMSE and adjusted R^2^ values for the U.S. overall and the average for 51 U.S. regions.

	United States overall		Average for 50 states and Washington, D.C.	
	52-Week Forecasting RMSE	2004–2011 Mean Adjusted R^2^	Mean 52-Week Forecasting RMSE	2004–2011 Adjusted R^2^
*AIC*(i_*_)	2.3342	0.2682	0.1823	0.0593
All Models Average	2.5345	0.2560	0.1859	0.0577
Top Models Average	2.5453	0.2543	0.1861	0.0567
AR(1) *AIC*(i_*_)	1.9788	0.2369	0.1808	0.0735
AR(1) All Models Average	1.8954	0.2249	0.1785	0.0713
AR(1) Top Models Average	1.8982	0.2682	0.1786	0.0707

Abbreviations: *AIC*(i_*_): lowest AIC model, All Models Average: average of 2^n^−1 models using posterior probabilities, Top Models Average: average of few most probable models using posterior probabilities, AR(1): models using the simple AR(1) Cochrane-Orcutt correction, RMSE: root-mean-square error.

**Figure 2:**
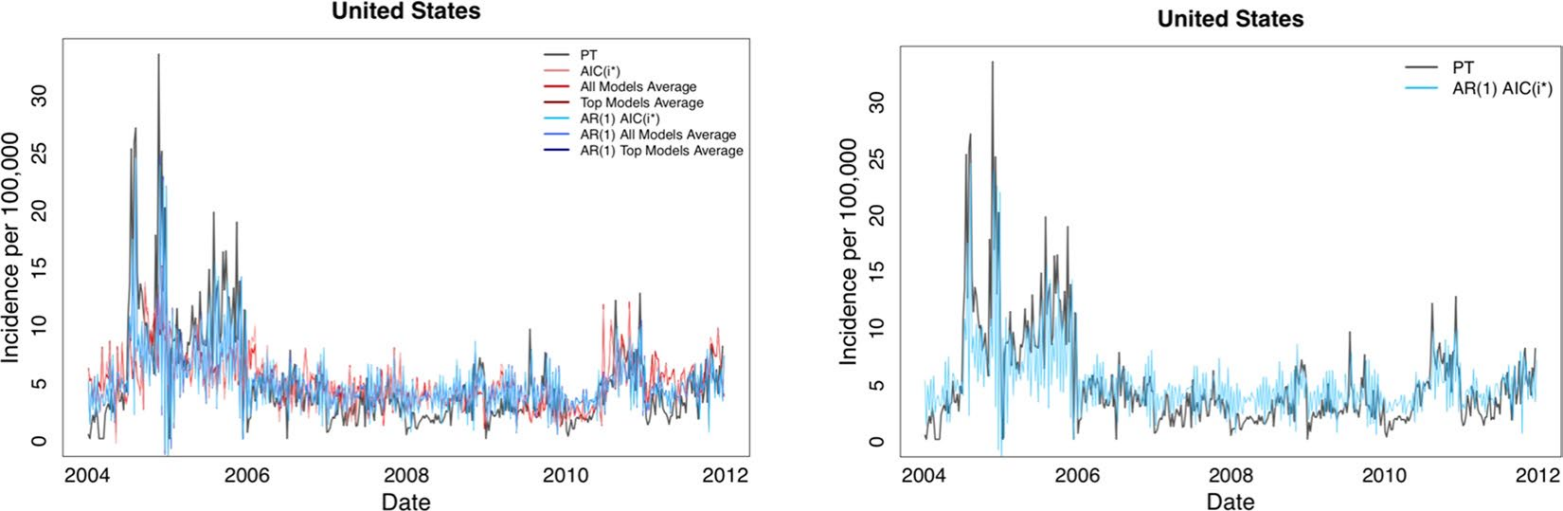
Time-series graphs showing PT pertussis incidence (black) per 100,000 people as a function of year for national U.S. data for 2004–2011. The left panel shows the results of all 6 modeling methods (see text), and the right panel shows the optimized AR(1) *AIC*(i_*_) model. The accuracy of this model supports previous findings that in larger geographic regions such as California^23^ and Australia^24^, GT models can track incidence. Some state-level models may be less accurate because they expose new sources of cultural and sociodemographic variability that are inconsequentially combined in the national model. Abbreviations: PT: Project Tycho, *AIC*(i_*_): lowest AIC model, All Models Average: average of 2^n^−1 models using posterior probabilities, Top Models Average: average of few most probable models using posterior probabilities, AR(1): models using the simple AR(1) Cochrane-Orcutt correction.

**Figure 3:**
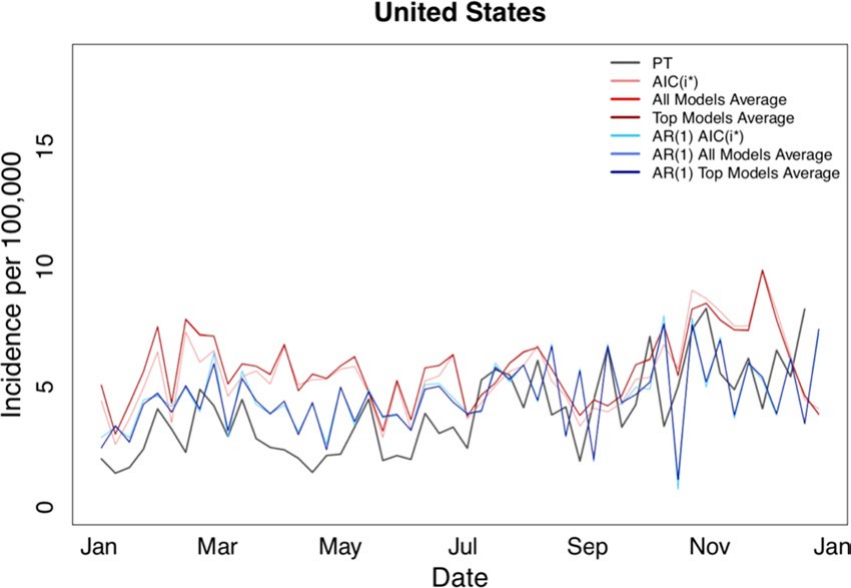
Estimated pertussis incidence per 100,000 population, all modeling methods for the 52-week 2011 forecasting period, United States. Abbreviations: PT: Project Tycho, *AIC*(i_*_): lowest AIC model, All Models Average: average of 2^n^−1 models using posterior probabilities, Top Models Average: average of few most probable models using posterior probabilities, AR(1): models using the simple AR(1) Cochrane-Orcutt correction.

The AR(1) *AIC*(i_*_) model produced a predictive time series that rose and fell with the trends of the U.S. PT incidence rates and had an adjusted R^2^ of 0.2369 (p < 0.05) (Fig. 2) and 52-week forecasting RMSE of 1.9788 (Fig. 3).

**Figure 4:**
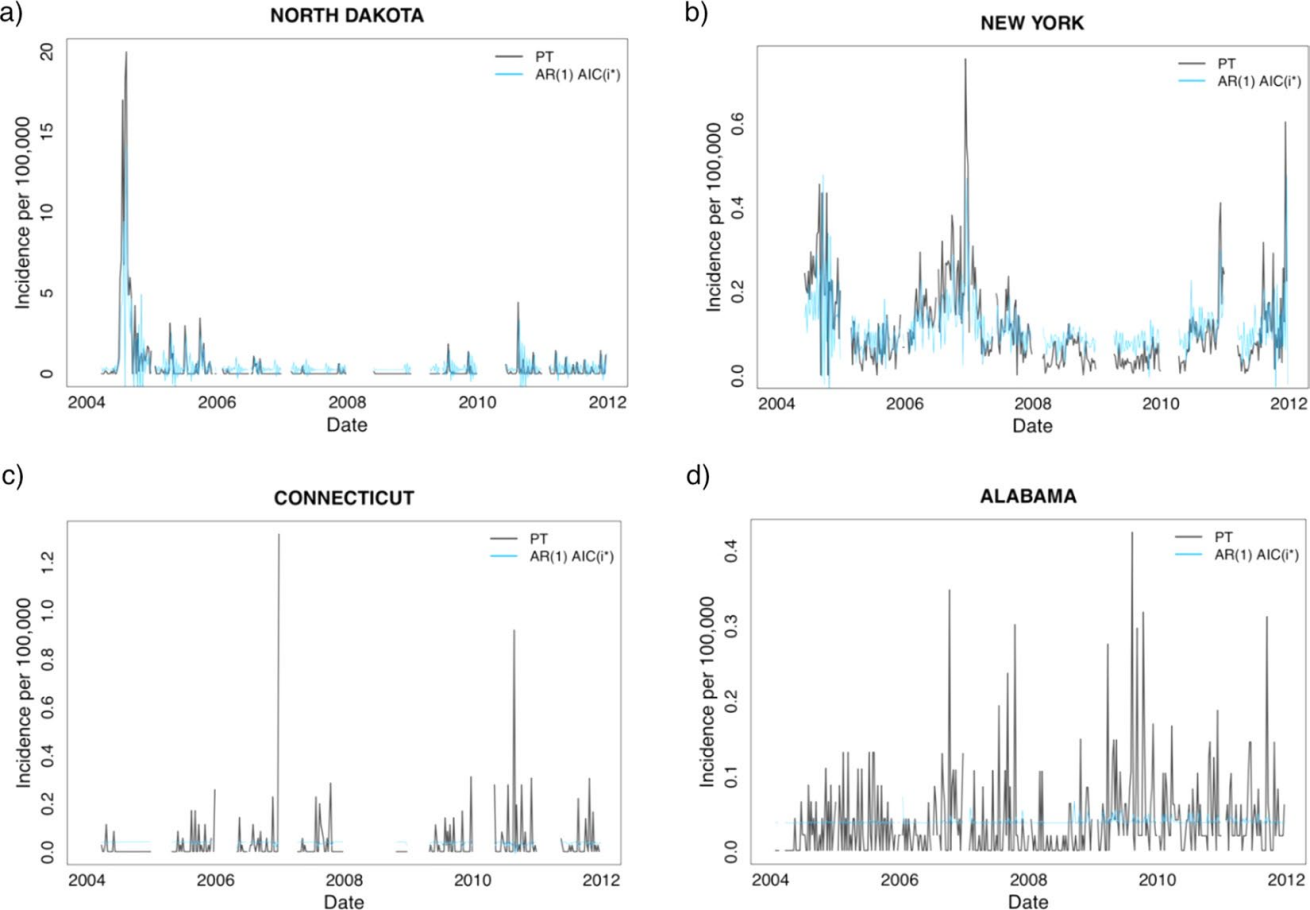
Time-series data showing recorded incidence from PT (black) and AR(1) *AIC*(i_*_) modeled incidence (blue) for 2004–2011 for 2 states (North Dakota and New York) with well performing models in the top panels and 2 states (Connecticut and Alabama) with poorly performing models in the bottom panels. The variability between state-model accuracy suggests that GT surveillance approaches cannot be performed uniformly across regions of the U.S.

In general, the AR(1) *AIC*(i_*_) models performed well for the individual states as well. All but 5 (Alabama, Connecticut, Louisiana, New Jersey, and Tennessee) of the state models showed a significant association between PT and GT data, with p < 0.05. The lowest observed adjusted R2 value was −0.0004 for Connecticut, and the largest was 0.3675 for North Dakota (see Fig. 4a). Other states for which our model performed well were Missouri (adjusted R^2^ = 0.2358), Mississippi (adjusted R^2^ = 0.2210), Delaware (adjusted R^2^ = 0.2065), and New York (adjusted R^2^ = 0.1673). The average adjusted R2 across all 50 states and the District of Colombia was 0.0735. Examples are shown in Fig. 4.

**Figure 5:**
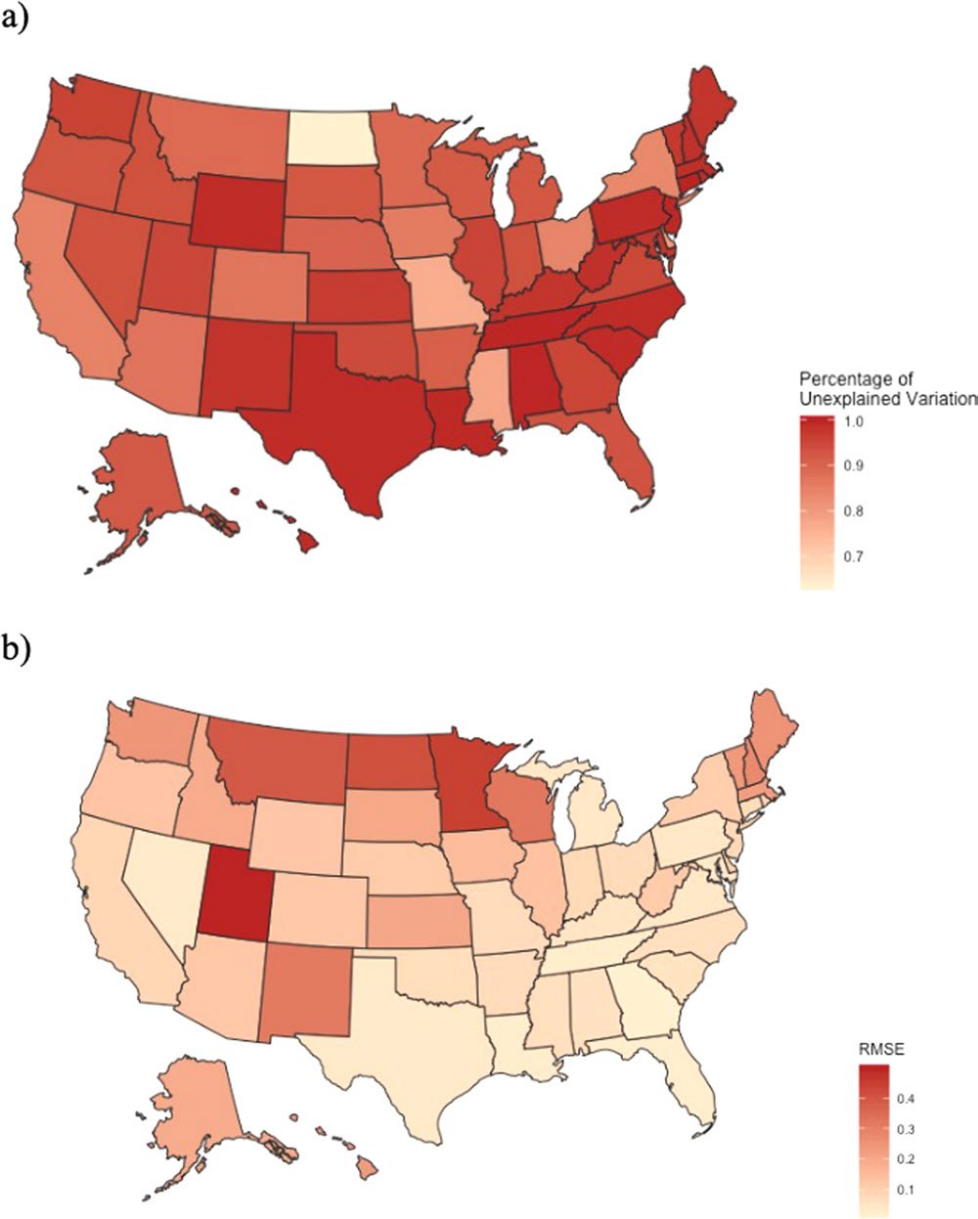
(**a**) Heat map displaying the percentage of unexplained variation (1−*adjusted R*^2^) in the AR(1) *AIC*(i_*_) models spanning the 2004–2010 timeframe in the US. A larger model explanatory power (R^2^) adjusted for the number of predictors (adjusted R^2^) is indicated with lighter shading. (**b**) Is a heat map illustrating the state models’ predictive accuracy (52-week forecasting RMSE in 2011) where lighter shading represents a lower RMSE value and a better performing state AR(1) *AIC*(i_*_) model.

There were notable differences in search terms that were included in each region’s AR(1) *AIC*(i_*_) model. For example, the Spanish terms “tos ferina” and “coqueleche” appeared only in the U.S., California, and New York AR(1) *AIC*(i_*_) models. The variability between independent variables in all 52 of the AR(1) *AIC*(i_*_) models illustrates how the density of search terms was not uniform throughout the country and among states (see Supplemental Table 1). We also observed a spectrum of regional differences between the 52-week forecasting RMSE computations (see Fig. 5b) as some models forecasted the PT incidence data better than others. These RMSE values ranged from 0.0155 (Georgia) to 0.6238 (North Dakota) with a mean of 0.1808.

The differences observed in RMSE values and model accuracy (Fig. 4) piqued our curiosity; might state-variable sociodemographic factors be responsible for some regional GT models working much better than others? A linear model was constructed incorporating state sociodemographic characteristics as independent variables and the 52-week forecasting RMSE values as the dependent variable. Notable associations are summarized in Table 4.

**Table 4:** Summary of coefficients, standard error, p values and a brief interpretation of each variable’s effect on the 52-week forecast RMSE in the exploratory sociodemographic model (see Table 2 for definitions of variables).

Variable	Coefficient	Standard Error	p	Interpretation
Intercept	0.1258	0.0262	2.57E-05	
ACEP	0.0196	0.0224	0.3869	Inconclusive
Age	−0.0916	0.0360	0.0153	States/regions with a higher population of younger adults (age 20–49 years) may produce more accurate GT data with their search patterns.
Poverty	−0.0099	0.0311	0.7519	Inconclusive
Internet	0.0332	0.0331	0.3233	More people with household internet access in a state/region corresponds to less accurate model forecasting.
Education	0.0646	0.0427	0.1393	More people with a bachelor’s degree in a state/region corresponds to less accurate model forecasting.
Urban	−0.0526	0.0246	0.0393	People might produce more accurate GT data (i.e., better model forecasting) in urban settings.
Vaccinated	−0.0234	0.0250	0.3557	Inconclusive
Republican	−0.0239	0.0324	0.4657	Inconclusive
Job	−0.0001	0.0260	0.9984	Inconclusive
Population	−0.2627	0.2991	0.3854	Inconclusive, however we note that (because of the GT privacy threshold) larger populations in a state/region associate with more available data from GT.
Household	−0.0208	0.0178	0.2509	States/regions with more people per household may have more accurate GT data.
Birth	0.0903	0.0301	0.0048	A higher birth rate might correspond with less accurate GT model predictions.
Immigration	0.1272	0.1823	0.4896	Inconclusive

Interpretations were deemed inconclusive if the standard error was larger than the magnitude of the coefficient – otherwise the interpretation explains the coefficient’s directionality (how an increase in the variable relates to the forecasting RMSE).

Abbreviations: GT: Google Trends. See Table 2 for variable descriptions.

The variables with the largest corresponding coefficients, i.e., having an impact on the similarity between GT forecasting and PT data were state population, number of immigrants, percent of the population aged 20–49 years, birth rate, and percent with a bachelor’s degree. Other variables had smaller coefficients such as average household size, percent in poverty, and percent with a job in the health sector (healthcare practitioners/technical occupations and healthcare support occupations). The sign on each coefficient shown in Table 4 illustrates the variable’s directional effect where negative coefficients correspond to smaller RMSE values (i.e., a more accurate 52-week forecast). This exploratory model (adjusted R^2^ = 0.3184 and p = 0.00707) suggested that some of the selected sociodemographic factors might help to explain the variability in the 52-week RMSE forecasts.

## Discussion

We found that search data from GT on pertussis and pertussis-related key words could be used to predict national pertussis incidence trends in U.S. public health surveillance data for this resurgent disease. However, when we examined the relationship of GT and surveillance data at the state level, the correlation varied – some states showed a strong correlation while others did not. We are curious as to why some states with a relatively high adjusted R2 did not necessarily also boast a low RMSE in the forecasting (Fig. 4a,b). For example, North Dakota’s model had the highest adjusted R^2^ yet did not perform relatively well in the 52-week RMSE forecasting. This may be due to the limited 52-week snapshot of the forecasting; we speculate that if the 52 weeks of the forecasting time period did not have enough pertussis cases to ignite exciting search patterns (or to boost GT search data above the privacy threshold) that the models may have not been robust enough to create accurate predictions. We found that some of the variation in the predictive power among regional models could be explained by state population characteristics, including the percentage of younger adults (p = 0.0153) and the birth rate (p = 0.0048). Our approach, using IP surveillance as an adjunctive means of collecting data on incidence, may be important as data now collected on pertussis incidence is often delayed, and the disease may be underdiagnosed. This may be a model that is useful for IP surveillance of other diseases, and our findings of geographic variability raise a new and important caveat to this approach.

The findings of the present study extend the framework of existing GT literature. Dukic *et al*. (2012) demonstrated that Google Flu Trends and Google News counts could be utilized as proxies for influenza surveillance^31^. Majumder *et al*. at HealthMap showed that IP data can be used to provide preemptive alerts for global health threats and can successfully be used to monitor outbreaks of mumps^32^. Our results support the findings of Pollet *et al*. in California and Zhang *et al*. in Australia, who both demonstrated that GT models were effective in large-scale regional tracking of pertussis incidence^23,24^. This was evident in our overall U.S. model which was quantitatively significant and produced a qualitatively similar time series of incidence.

IP surveillance has increased in popularity in recent years as internet search queries are being used to model various infectious diseases around the world. Such studies include analyses of the West Nile virus in the U.S.^33^, and Zika virus in Brazil and Colombia^34^. Most research efforts in the field, however, have addressed influenza incidence trends. Lu *et al*. recently incorporated a self-correcting statistical method to track influenza at the state level, and their improved modeling methodology boasts higher correlations with lower errors^35^. The findings from these recent studies suggest that IP surveillance could play an important role in disease surveillance as digital connection and search engine use proliferates in the twenty-first century. IP surveillance could prove especially useful if accurate state or regional models can be used to inform local policy in real time. The apparent sources of inaccuracy in CDC reporting of pertussis incidence result in limited ability to evaluate whether the GT models can yield a superior predictor for pertussis incidence than the MMWR or PT data – the current “gold standard” source of data in the U.S. As discussed earlier, the CDC pertussis incidence data are limited due to sources of error including false-positives, false-negatives, underdiagnosis, underreporting, common use of clinical diagnosis without laboratory testing, patients with pertussis often not seeking medical care, and subclinical cases – all of which lead to missing or inaccurate data points in the timeseries.

Despite the promising literature suggesting the utility of GT surveillance, it is important to note that there are limitations to our study and to general approaches of using internet search data to estimate epidemiologic trends. In the case of pertussis, other concerns include pollution of the GT data by people searching the terms for non-clinical or academic purposes (unrelated to actual disease) or people performing internet searches of pertussis-related terms because of a sick relative in another state or even another country. These limitations might explain the somewhat counter-intuitive result that states with higher education and more household internet access had higher RMSE outcomes (positively signed coefficients in Table 4). GT data may be useful for capturing subclinical/unreported cases of pertussis, yet there is no guarantee that any infected individual will cause a ripple of pertussis-related Google searches. Also, search activity patterns in a population related to pertussis disease may change over time. For example, others have noted that the ability of GT data to predict outbreaks of influenza (based on searches for influenza symptoms) has decreased over time due to over-predicting complications^36,37^. The public health incidence data themselves are not a perfect reflection of actual disease incidence, complicating interpretation of our conclusions.

To conclude, we found that the use of sociodemographic variables accounted for some of the variability in the ability of Google search data to forecast state-level pertussis incidence. While decreasing an IP surveillance model’s geographic area to the state level is useful for local policy makers, doing so may expose new sources of cultural and sociodemographic variability that are inconsequentially combined in the national model. Assessing local epidemiology and using local IP and sociodemographic data may reduce error in forecasting disease estimates of public health importance. The variation between the accuracy of state-based models motivates a new direction for future research questions – some of which should pertain to sociodemographic factors. These findings may be relevant to the future development of artificial intelligence algorithms aimed at the forecasting or “nowcasting” of epidemiologic trends of infectious diseases.

## Supplementary information

Supplementary Table1 and Time-series Figures

## Data Availability

The data that support the findings of this study are available from Google Trends but restrictions apply to the public availability of these data. Contact Google Trends API for access to these data. All other data used for this analysis can be found at the publicly available websites (e.g. Project Tycho and U.S. Census Bureau) mentioned in the methods section.
