# Supplementary material for: Tracking U.S. Pertussis Incidence: Correlation of Public Health Surveillance and Google Search Data Varies by State
Source: Sci Rep. 2019 Dec 24;9:19801. doi: 10.1038/s41598-019-56385-z (PMC6930253; doi:10.1038/s41598-019-56385-z)
Supplement: Supplementary file 1 — Supplementary Table1 and Time-series Figures [file 41598_2019_56385_MOESM1_ESM.pdf]

Tracking U.S. Pertussis Incidence: Correlation of Public Health Surveillance and Google Search Data Varies by State

Christopher H. Arehart[1], Michael Z. David[2], Vanja Dukic[1]

[1]Department of Applied Mathematics, University of Colorado Boulder,
Boulder, Colorado, 80309, United States of America
[2]Division of Infectious Diseases, Department of Medicine, University of Pennsylvania,
Philadelphia, Pennsylvania, 19104, United States of America

Supplemental Table 1. Results for each geographic region's Cochrane Orcutt corrected lowest AIC, AR(1) $AIC(i_*)$, model using GT data to predict PT incidence rates. These results include the models' adjusted R^2 from 2004 to the 52nd week of 2010, the 2011 52-week RMSE of PT incidence and the model-generated predictive forecast, and the GT terms included as independent variables in the model.

Region	Adjusted R^2	52-Week Forecasting RMSE	GT Search Terms in AR(1) $AIC(i_*)$
United States	0.2369	1.9788	bordatellabordetella + \`pertussis symptoms\` + \`tos ferina\` + \`whooping cough symptoms\`
Alaska	0.0801	0.1656	bordatellabordetella + pertusispertussis + \`whooping cough\`
Alabama	0.0026	0.0551	\`whooping cough symptoms\`
Arkansas	0.0931	0.0654	\`chronic cough\` + \`whooping cough symptoms\`
Arizona	0.1268	0.1187	\`whooping cough symptoms\` + \`whooping cough\`
California	0.1534	0.0781	coqueluche + pertusispertussis + \`pertussis symptoms\` + \`whooping cough\`
Colorado	0.1319	0.1397	\`chronic cough\` + pertusispertussis + \`whooping cough\`
Connecticut	-0.0004	0.0711	\`whooping cough symptoms\`
Delaware	0.0370	0.0643	\`whooping cough symptoms\`
District of Colombia	0.2065	0.0995	bordatellabordetella + pertusispertussis
Florida	0.0677	0.0195	\`pertussis treatment\`
Georgia	0.0464	0.0155	\`whooping cough symptoms\`
Hawaii	0.0096	0.2329	\`whooping cough\`
Iowa	0.1438	0.1378	pertusispertussis + \`whooping cough\`
Idaho	0.0661	0.1896	pertusispertussis + \`whooping cough\`
Illinois	0.0449	0.1312	\`chronic cough\` + \`whooping cough symptoms\` + \`whooping cough treatment\`
Indiana	0.0657	0.0758	bordatellabordetella + pertusispertussis + \`whooping cough symptoms\` + \`whooping cough\`
Kansas	0.0324	0.2048	\`whooping cough\`
Kentucky	0.0230	0.0470	bordatellabordetella + \`whooping cough\`
Louisiana	0.0060	0.0225	\`chronic cough\`
Massachusetts	0.0130	0.2194	\`chronic cough\`
Maryland	0.0240	0.0353	bordatellabordetella + pertusispertussis + \`whooping cough symptoms\`
Maine	0.0205	0.2495	bordatellabordetella
Michigan	0.0778	0.0367	\`whooping cough symptoms\` + \`whooping cough\`
Minnesota	0.1105	0.4626	bordatellabordetella + \`chronic cough\` + pertusispertussis + \`whooping cough symptoms\`
Missouri	0.2358	0.0668	pertusispertussis + \`whooping cough symptoms\` + \`whooping cough\`
Mississippi	0.2210	0.0600	\`whooping cough symptoms\`
Montana	0.1118	0.4130	pertusispertussis
North Carolina	0.0110	0.0583	pertusispertussis
North Dakota	0.3675	0.6238	pertusispertussis + \`whooping cough\`
Nebraska	0.0986	0.0977	\`whooping cough symptoms\` + \`whooping cough\`

New Hampshire	0.0157	0.2587	bordatellabordetella
New jersey	0.0040	0.0782	\`whooping cough\`
New Mexico	0.0190	0.3143	\`whooping cough symptoms\`
Nevada	0.0654	0.0361	\`whooping cough symptoms\` + \`whooping cough\`
New York	0.1673	0.1280	bordatellabordetella + \`chronic cough\` + coqueluche + \`whooping cough adults\` + \`whooping cough symptoms\` + \`whooping cough treatment\` + \`whooping cough\`
Ohio	0.1580	0.0620	pertusispertussis + \`whooping cough symptoms\` + \`whooping cough\`
Oklahoma	0.0555	0.0645	\`whooping cough symptoms\`
Oregon	0.0735	0.1382	\`whooping cough symptoms\` + \`whooping cough\`
Pennsylvania	0.0092	0.0405	\`chronic cough\`
Rhode Island	0.0126	0.0854	\`whooping cough symptoms\`
South Carolina	0.0111	0.0499	\`chronic cough\`
South Dakota	0.0774	0.1805	\`whooping cough\`
Tennessee	0.0023	0.0249	pertusispertussis
Texas	0.0094	0.0326	\`whooping cough\`
Utah	0.0478	0.5777	bordatellabordetella + \`chronic cough\` + pertusispertussis + \`whooping cough symptoms\` + \`whooping cough\`
Virginia	0.0508	0.0412	bordatellabordetella + \`pertussis symptoms\` + \`whooping cough\`
Vermont	0.0284	0.2207	pertusispertussis
Washington	0.0414	0.2472	\`whooping cough\`
Wisconsin	0.0837	0.3517	pertusispertussis + \`whooping cough symptoms\` + \`whooping cough\`
West Virginia	0.0158	0.1116	bordatellabordetella
Wyoming	0.0109	0.1203	\`whooping cough\`
State Average	0.0735	0.1808	

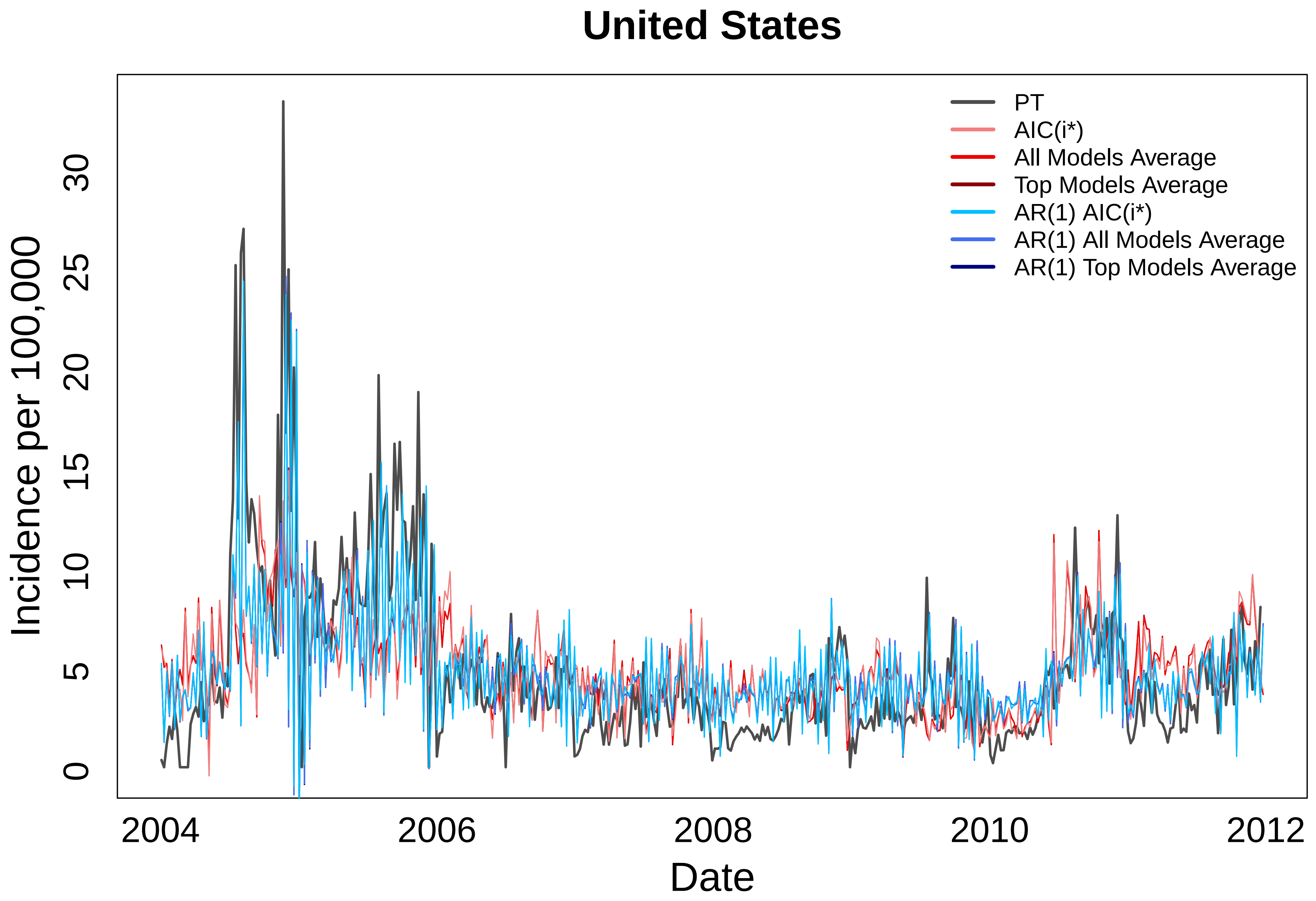

United States
PT
AIC(i*)
All Models Average
Top Models Average
AR(1) AIC(i*)
AR(1) All Models Average
AR(1) Top Models Average
Incidence per 100,000
0
5
10
15
20
25
30
2004
2006
2008
2010
2012
Date

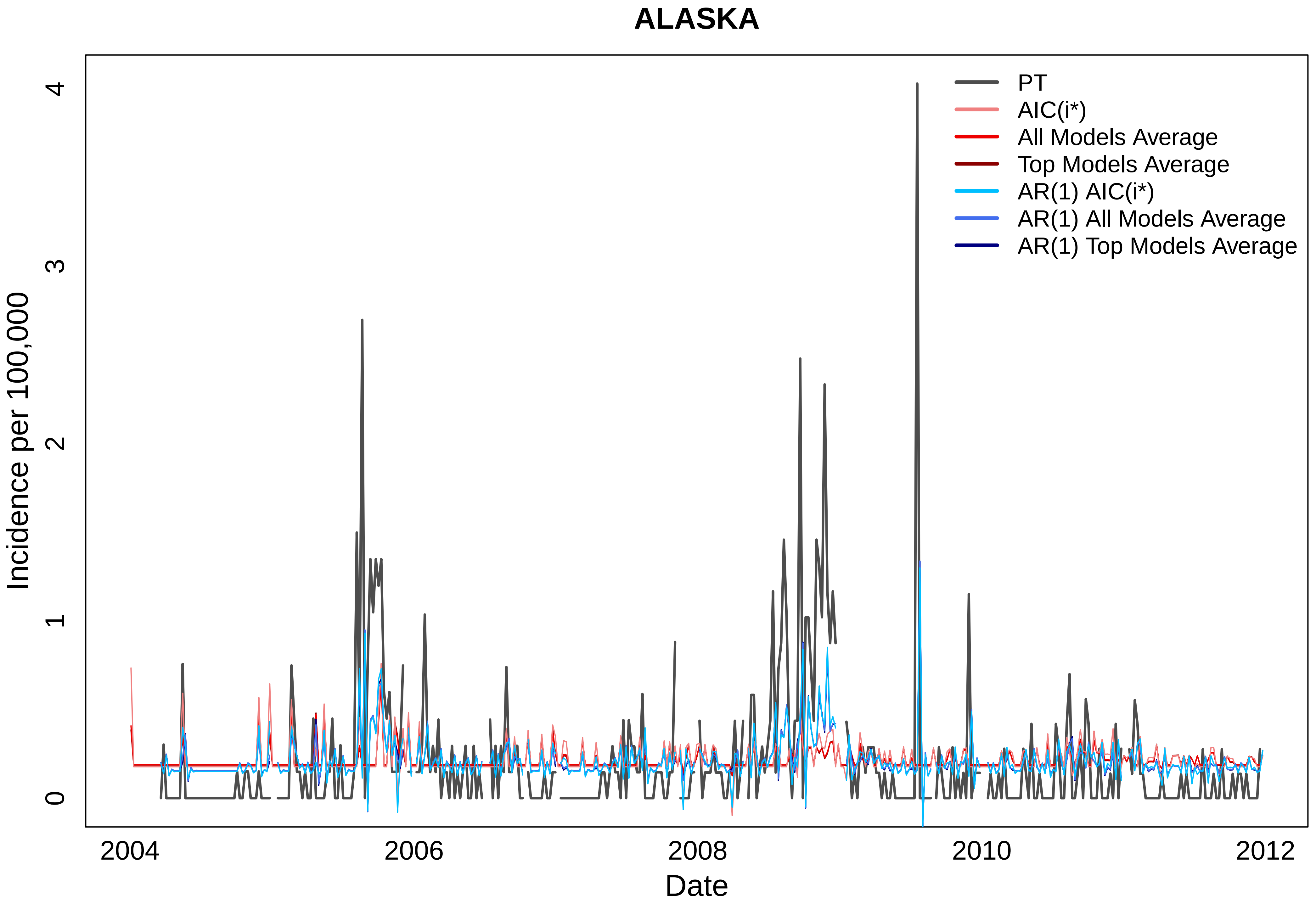
ALASKA
PT
AIC(i*)
All Models Average
Top Models Average
AR(1) AIC(i*)
AR(1) All Models Average
AR(1) Top Models Average
Incidence per 100,000
0
1
2
3
4
2004
2006
2008
2010
2012
Date

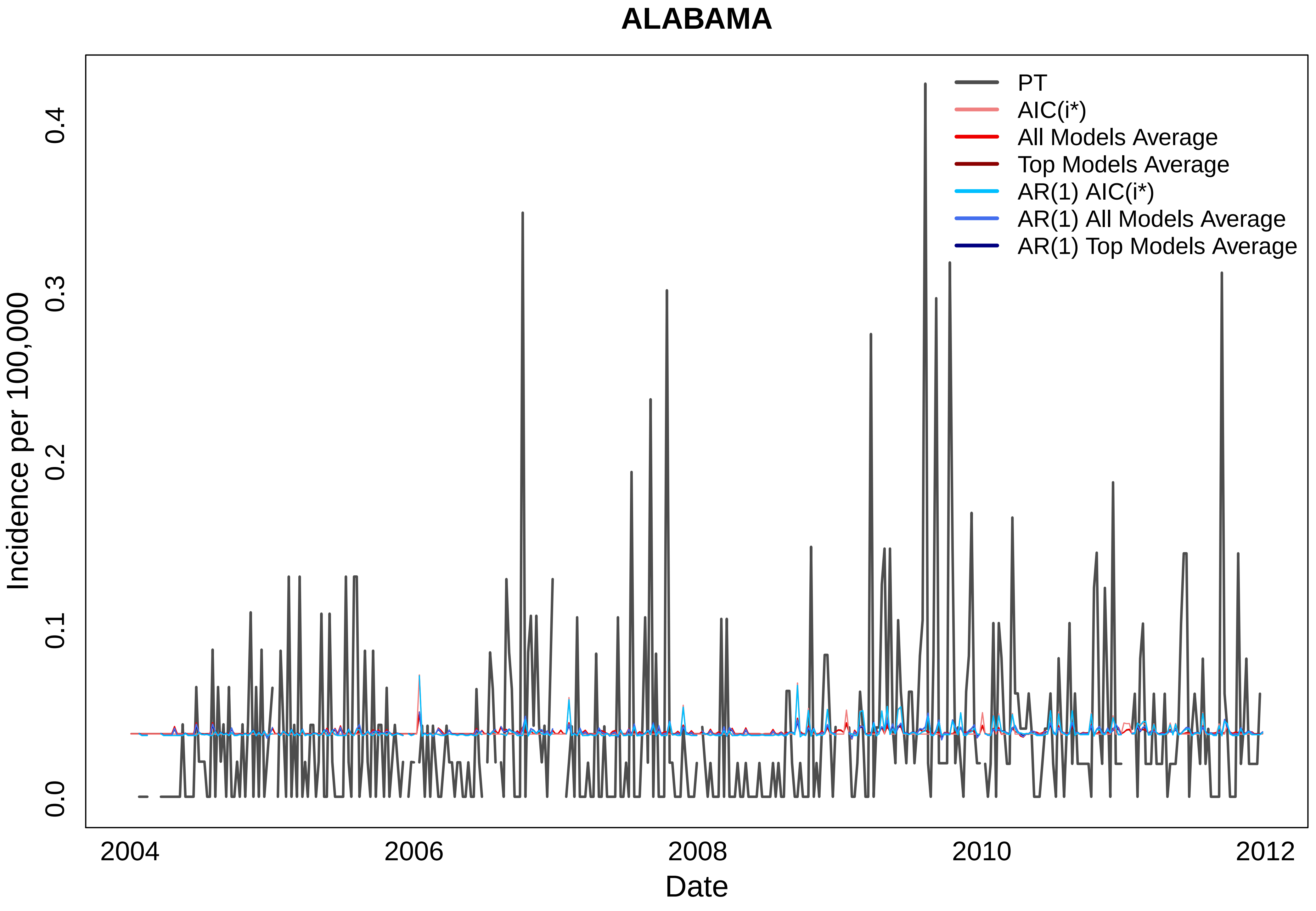

ALABAMA
PT
AIC(i*)
All Models Average
Top Models Average
AR(1) AIC(i*)
AR(1) All Models Average
AR(1) Top Models Average
Incidence per 100,000
0.0
0.1
0.2
0.3
0.4
2004
2006
2008
2010
2012
Date

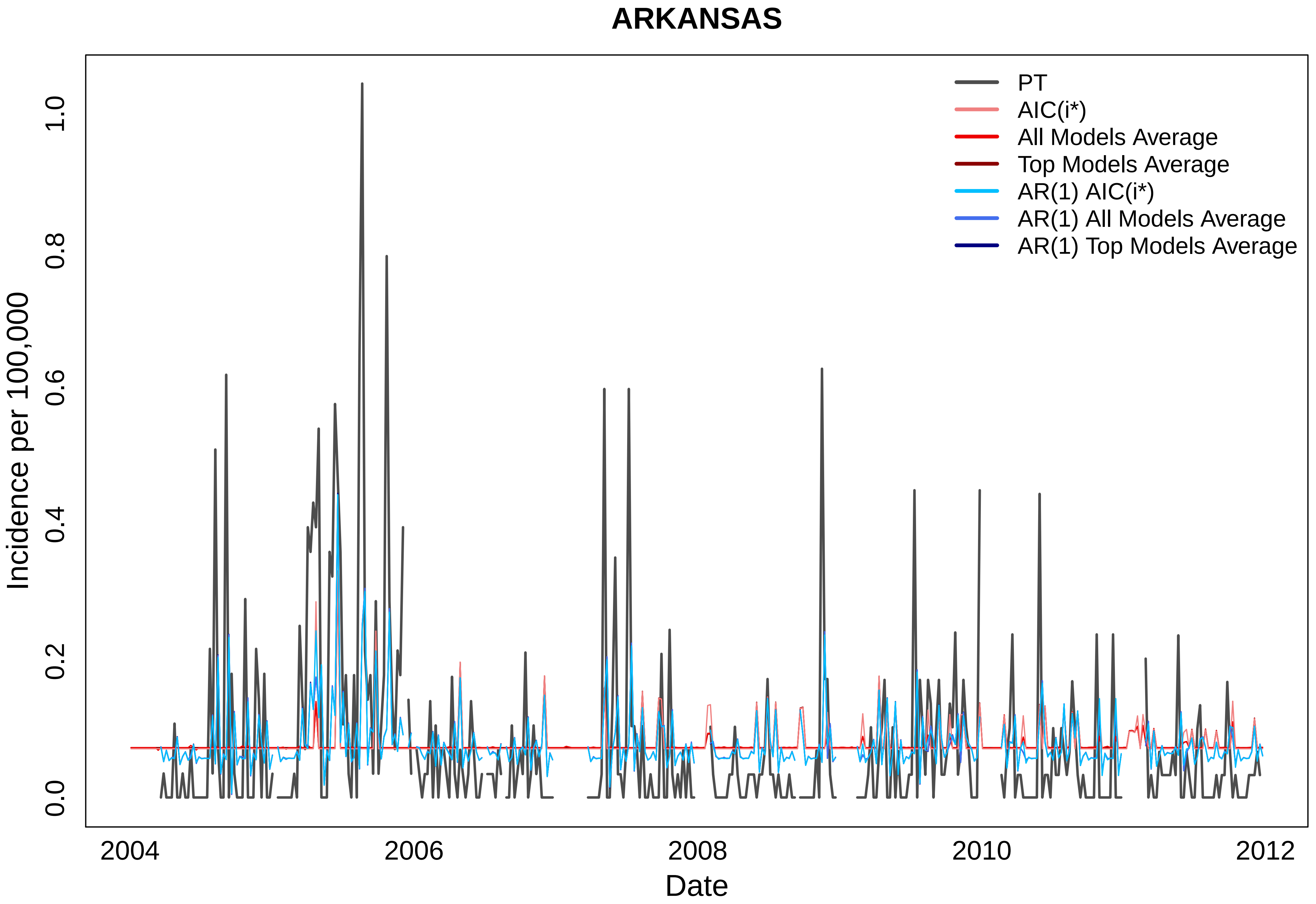
ARKANSAS
PT
AIC(i*)
All Models Average
Top Models Average
AR(1) AIC(i*)
AR(1) All Models Average
AR(1) Top Models Average
Incidence per 100,000
0.0
0.2
0.4
0.6
0.8
1.0
2004
2006
2008
2010
2012
Date

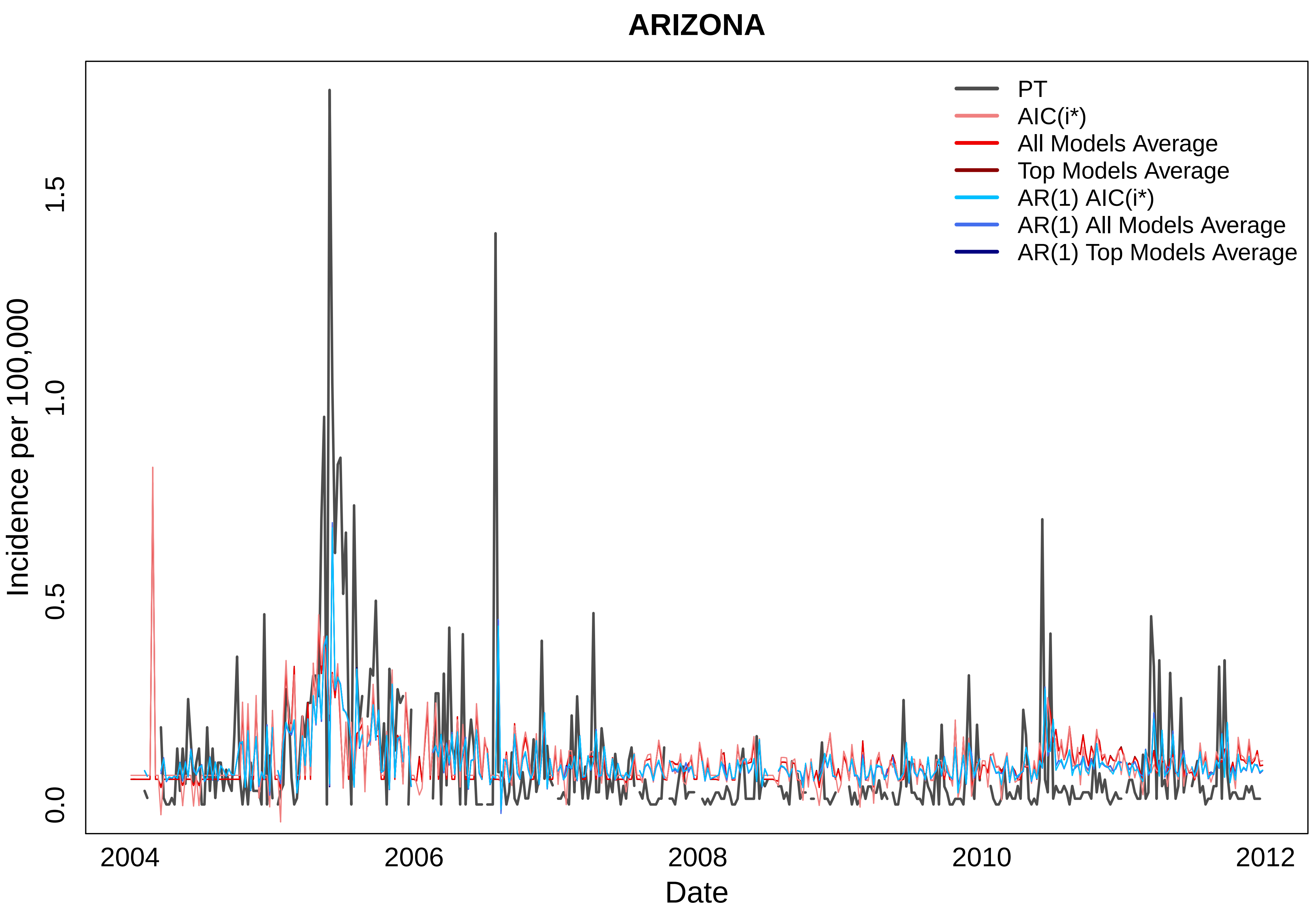
ARIZONA
PT
AIC(i*)
All Models Average
Top Models Average
AR(1) AIC(i*)
AR(1) All Models Average
AR(1) Top Models Average
Incidence per 100,000
0.0
0.5
1.0
1.5
2004
2006
2008
2010
2012
Date

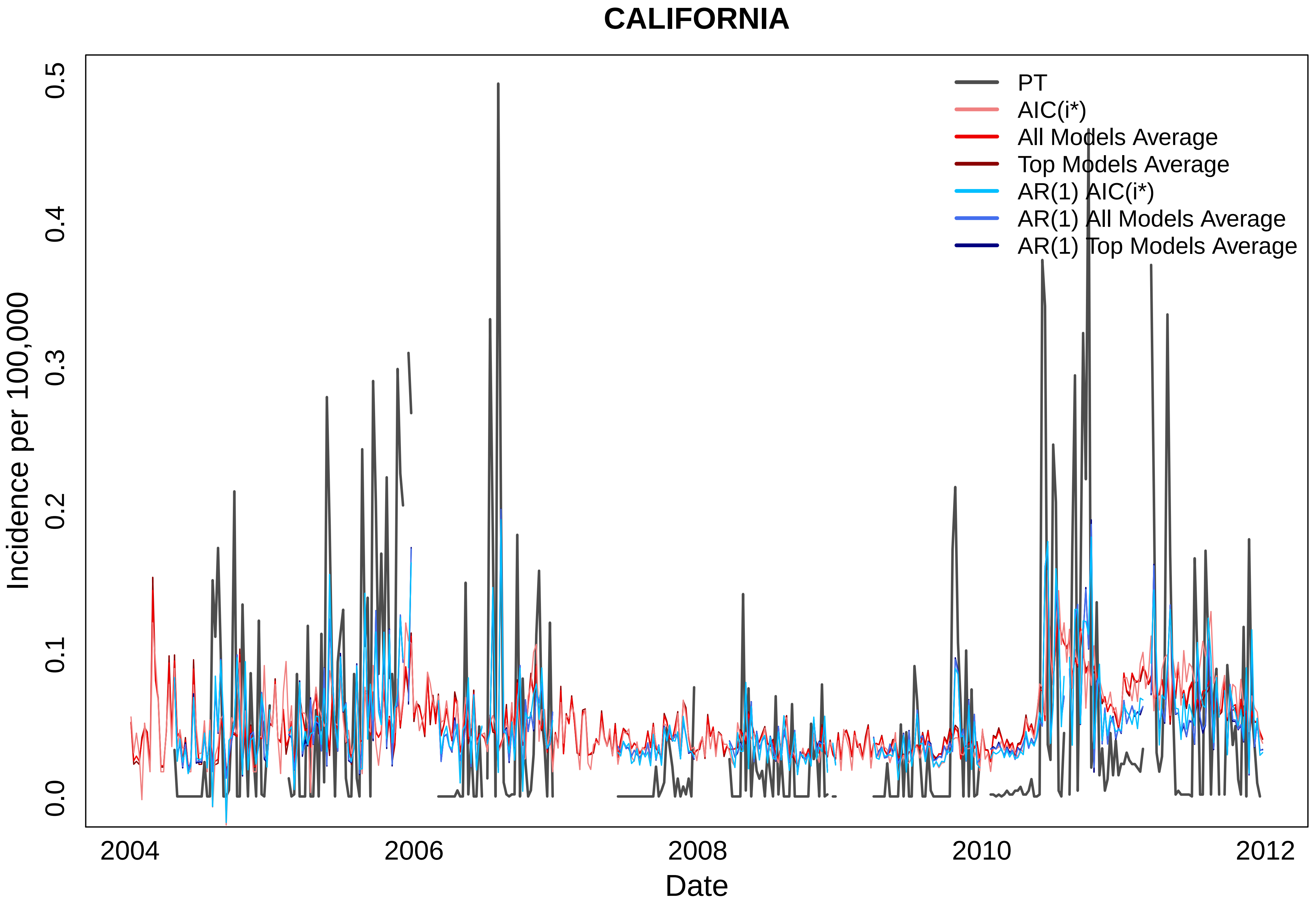

CALIFORNIA
PT
AIC(i*)
All Models Average
Top Models Average
AR(1) AIC(i*)
AR(1) All Models Average
AR(1) Top Models Average
Incidence per 100,000
0.0
0.1
0.2
0.3
0.4
0.5
2004
2006
2008
2010
2012
Date

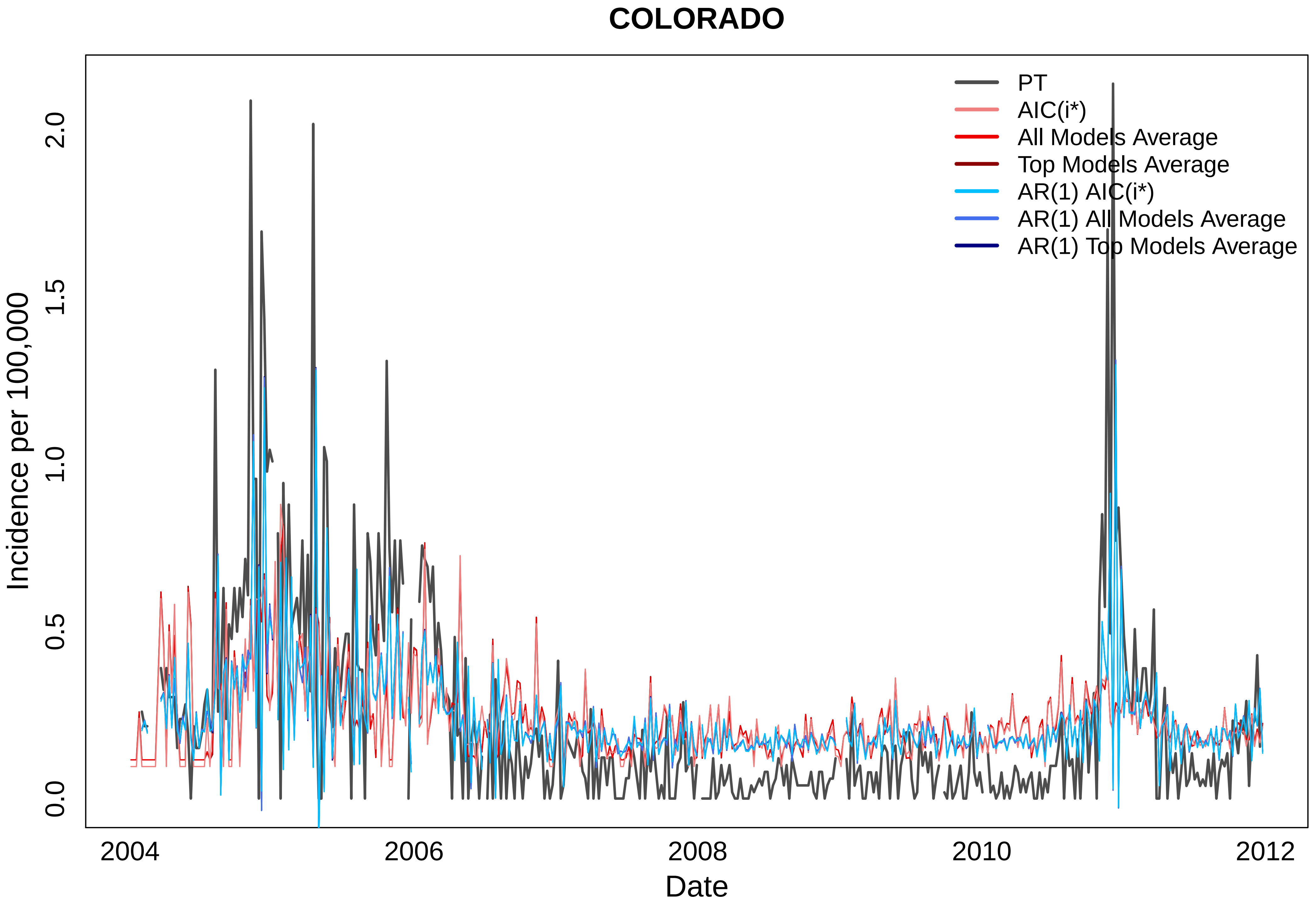
COLORADO
PT
AIC(i*)
All Models Average
Top Models Average
AR(1) AIC(i*)
AR(1) All Models Average
AR(1) Top Models Average
Incidence per 100,000
0.0
0.5
1.0
1.5
2.0
2004
2006
2008
2010
2012
Date

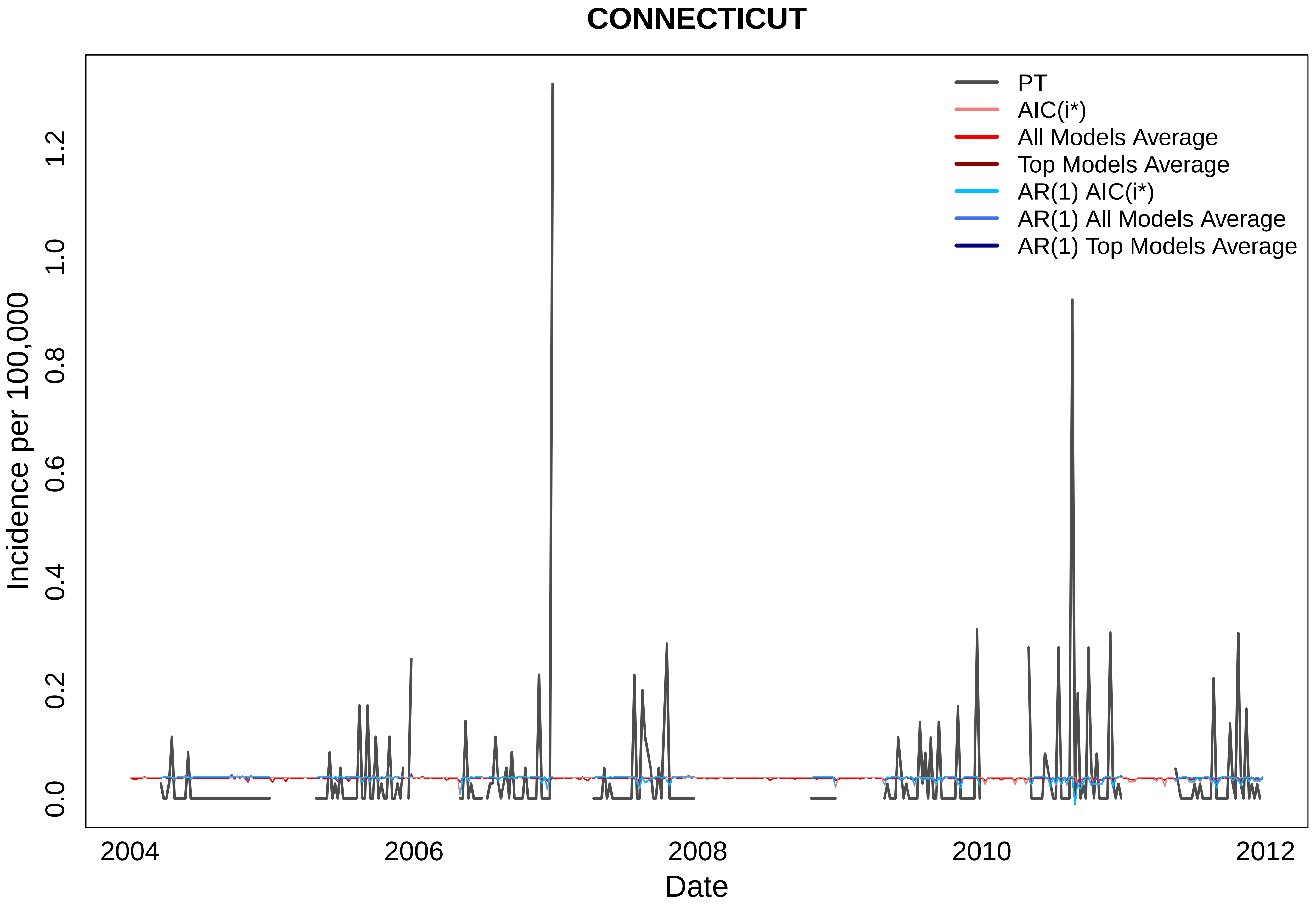
CONNECTICUT
PT
AIC(i*)
All Models Average
Top Models Average
AR(1) AIC(i*)
AR(1) All Models Average
AR(1) Top Models Average
Incidence per 100,000
0.0
0.2
0.4
0.6
0.8
1.0
1.2
2004
2006
2008
2010
2012
Date

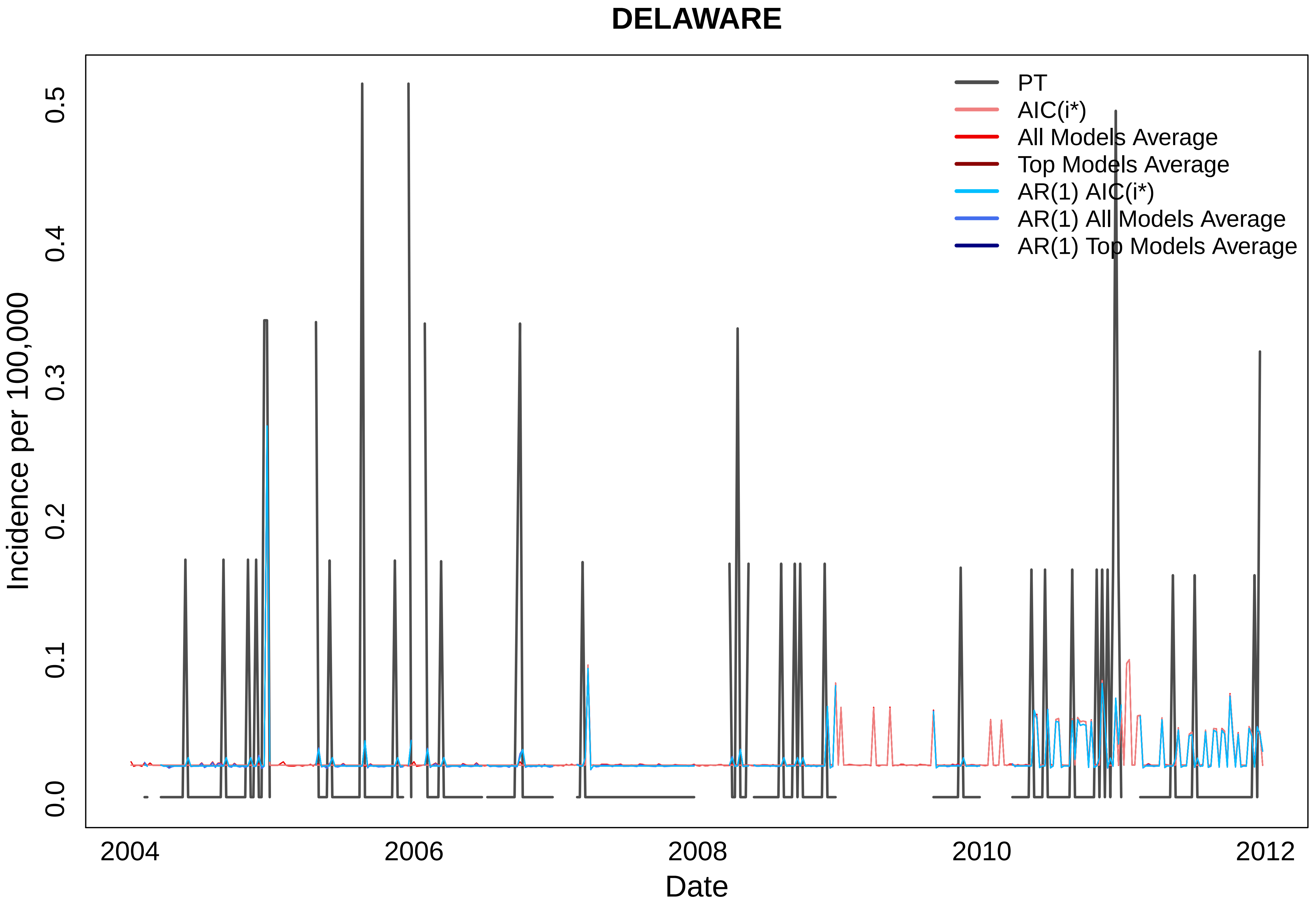
DELAWARE
PT
AIC(i*)
All Models Average
Top Models Average
AR(1) AIC(i*)
AR(1) All Models Average
AR(1) Top Models Average
Incidence per 100,000
0.0
0.1
0.2
0.3
0.4
0.5
2004
2006
2008
2010
2012
Date

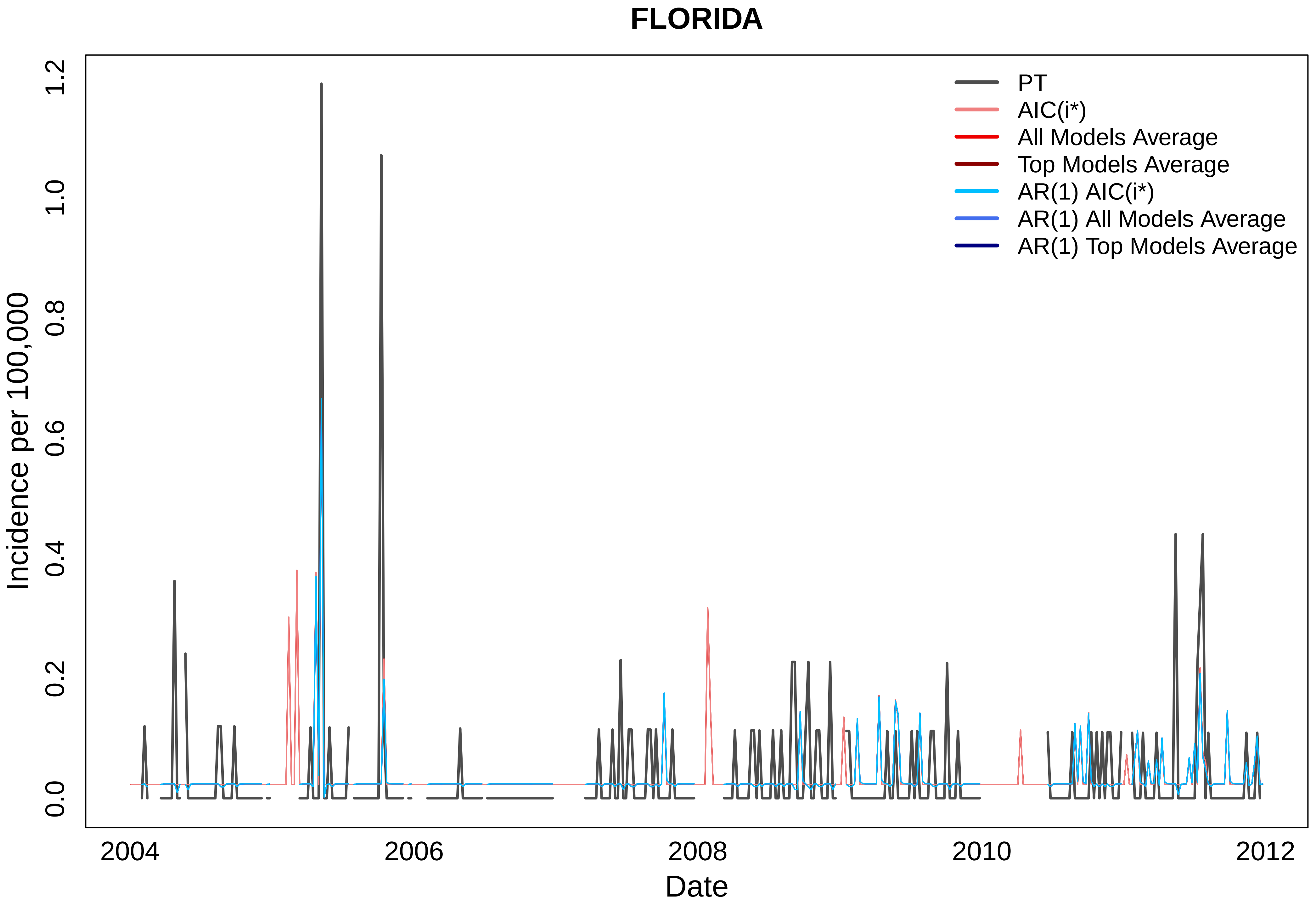

FLORIDA
PT
AIC(i*)
All Models Average
Top Models Average
AR(1) AIC(i*)
AR(1) All Models Average
AR(1) Top Models Average
Incidence per 100,000
0.0
0.2
0.4
0.6
0.8
1.0
1.2
2004
2006
2008
2010
2012
Date

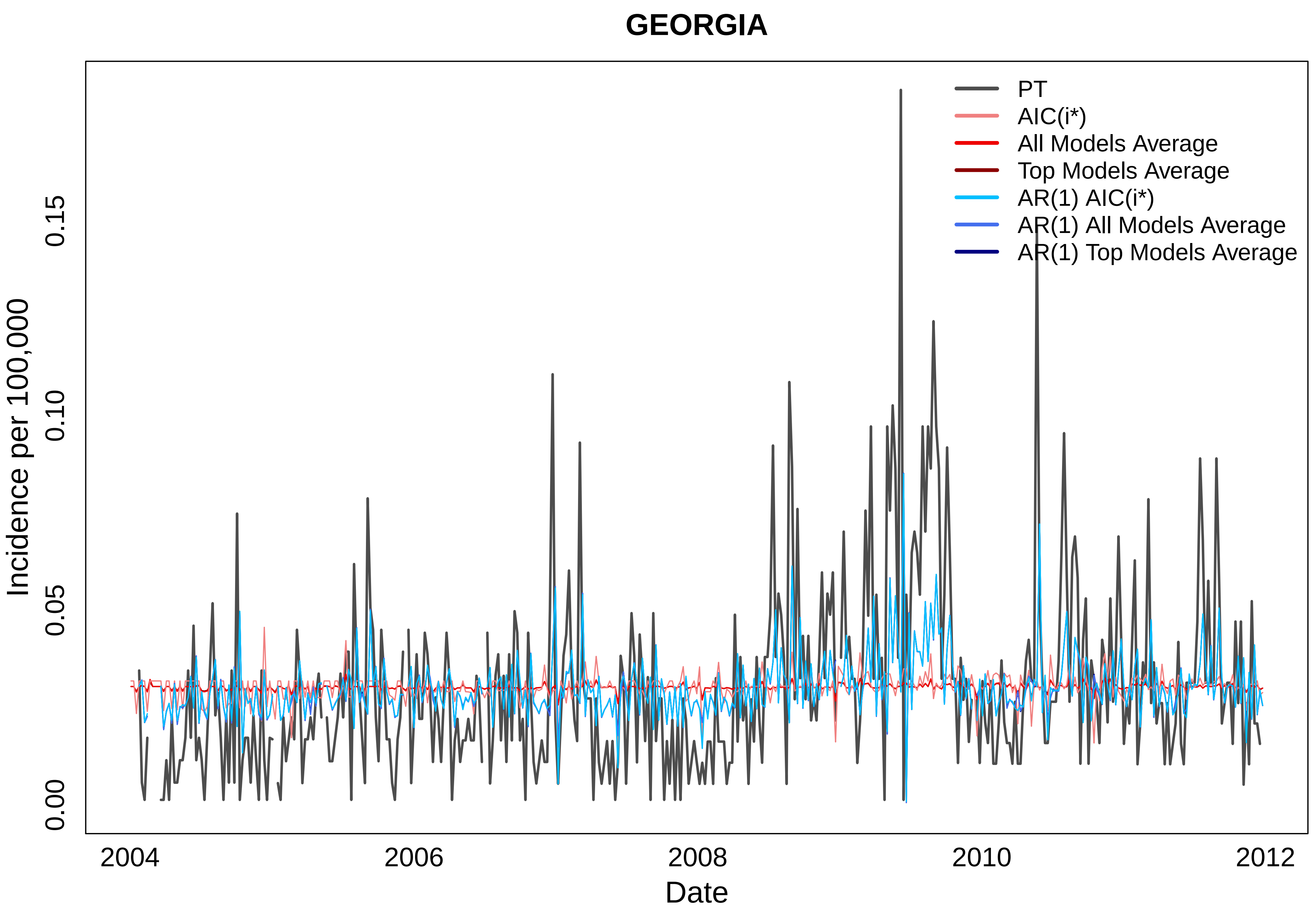
GEORGIA
PT
AIC(i*)
All Models Average
Top Models Average
AR(1) AIC(i*)
AR(1) All Models Average
AR(1) Top Models Average
Incidence per 100,000
0.00
0.05
0.10
0.15
2004
2006
2008
2010
2012
Date

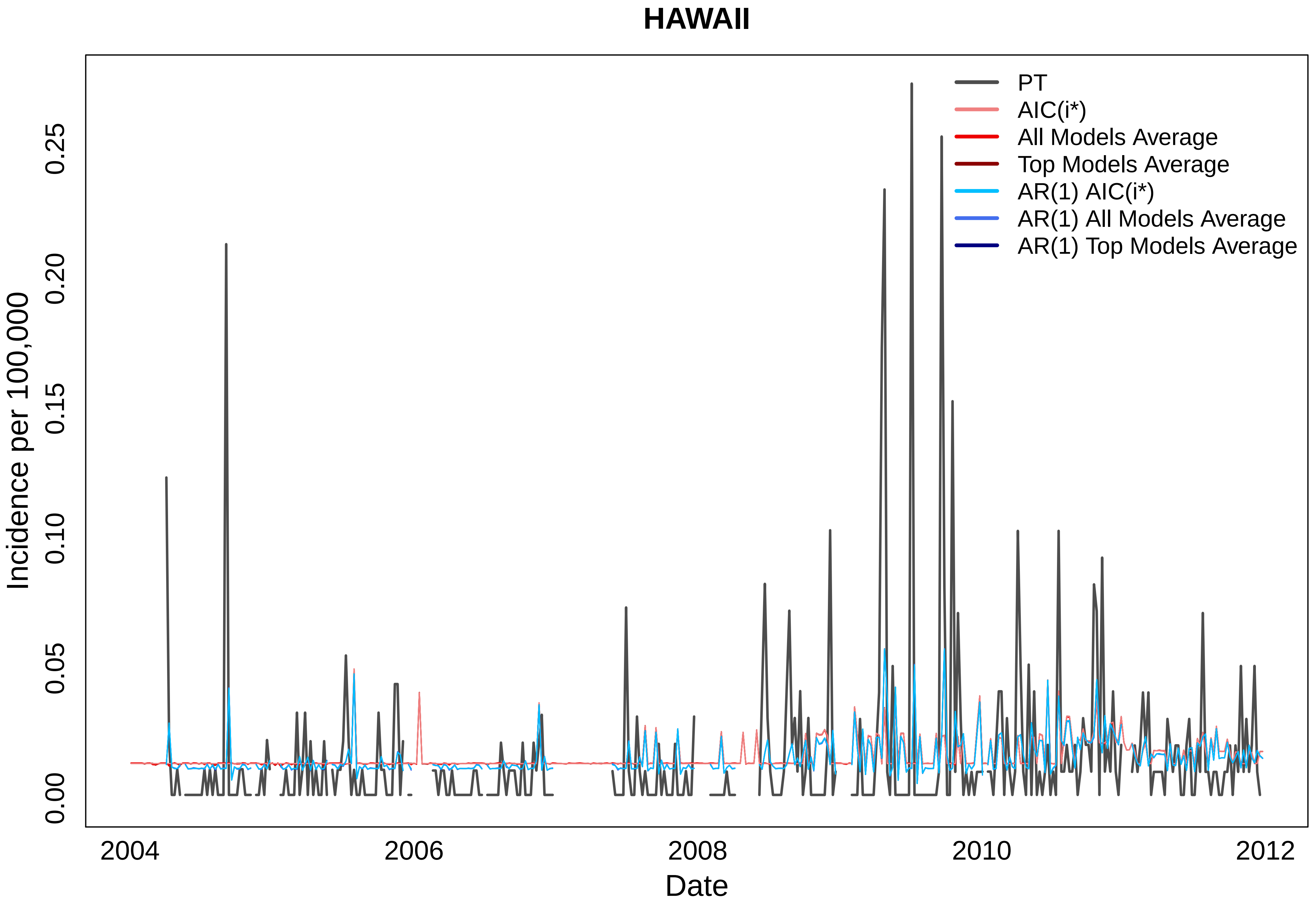

HAWAII
PT
AIC(i*)
All Models Average
Top Models Average
AR(1) AIC(i*)
AR(1) All Models Average
AR(1) Top Models Average
Incidence per 100,000
0.00
0.05
0.10
0.15
0.20
0.25
2004
2006
2008
2010
2012
Date

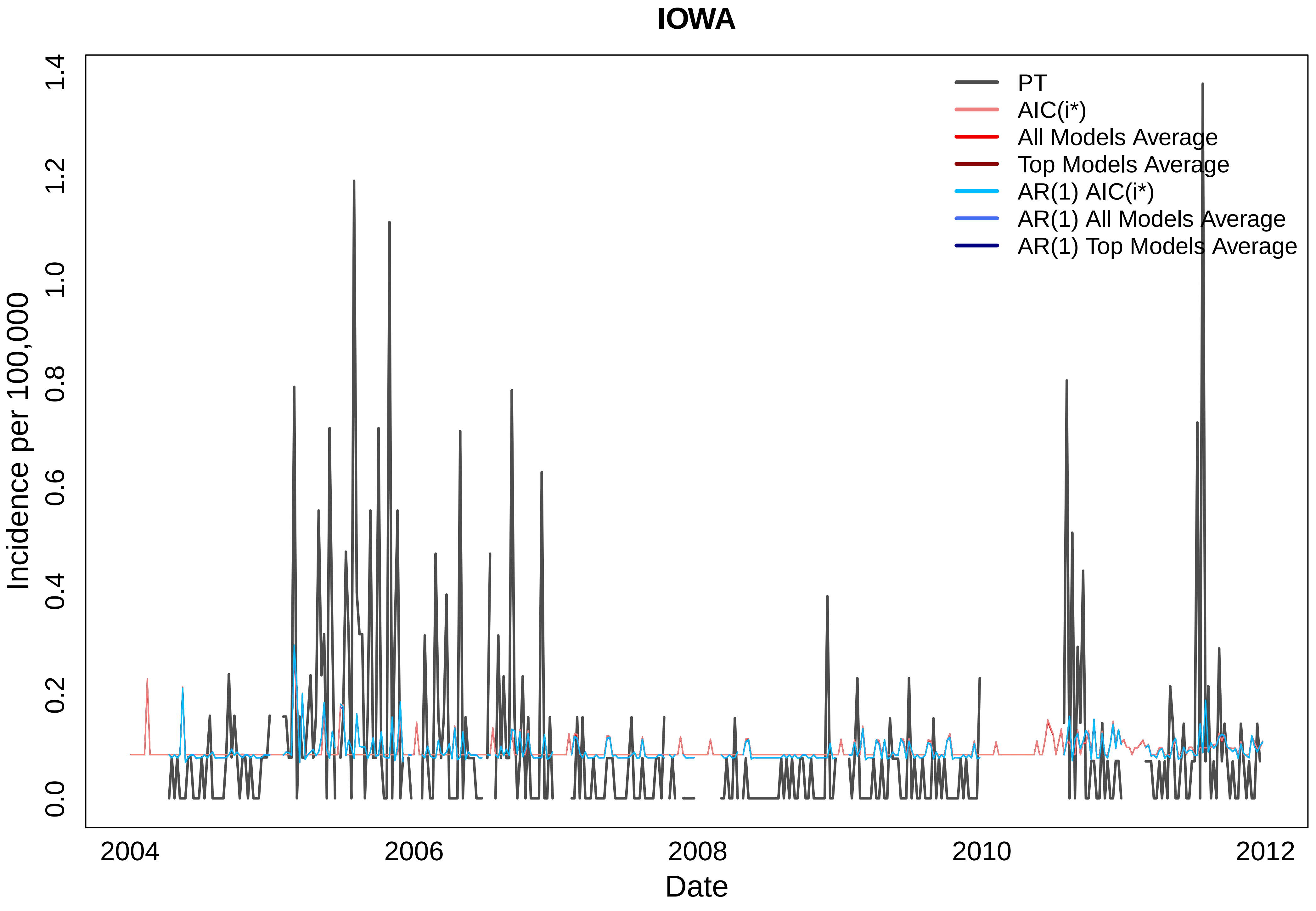
IOWA
PT
AIC(i*)
All Models Average
Top Models Average
AR(1) AIC(i*)
AR(1) All Models Average
AR(1) Top Models Average
Incidence per 100,000
0.0
0.2
0.4
0.6
0.8
1.0
1.2
1.4
2004
2006
2008
2010
2012
Date

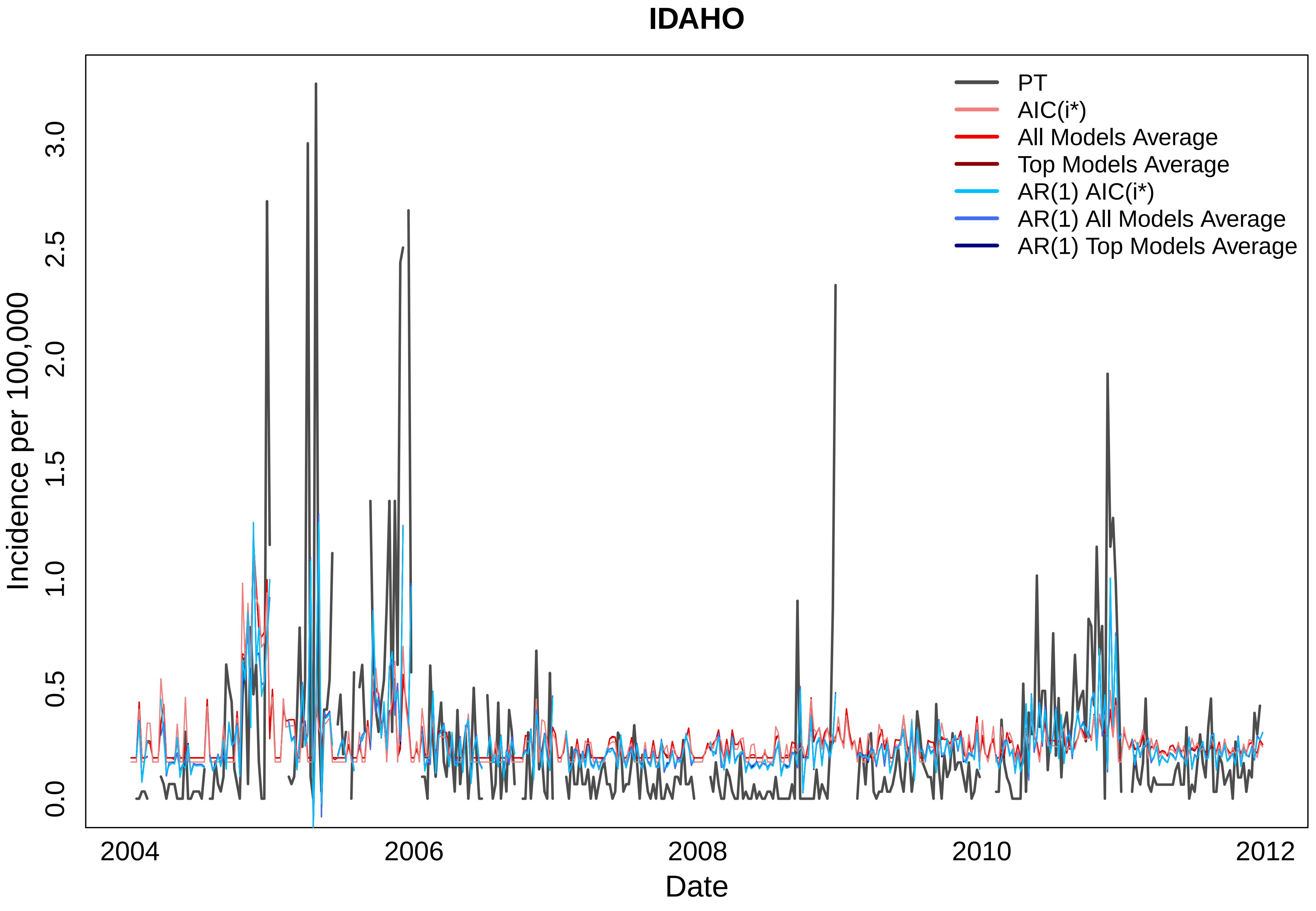
IDAHO
Incidence per 100,000
0.0
0.5
1.0
1.5
2.0
2.5
3.0
2004
2006
2008
2010
2012
Date
PT
AIC(i*)
All Models Average
Top Models Average
AR(1) AIC(i*)
AR(1) All Models Average
AR(1) Top Models Average

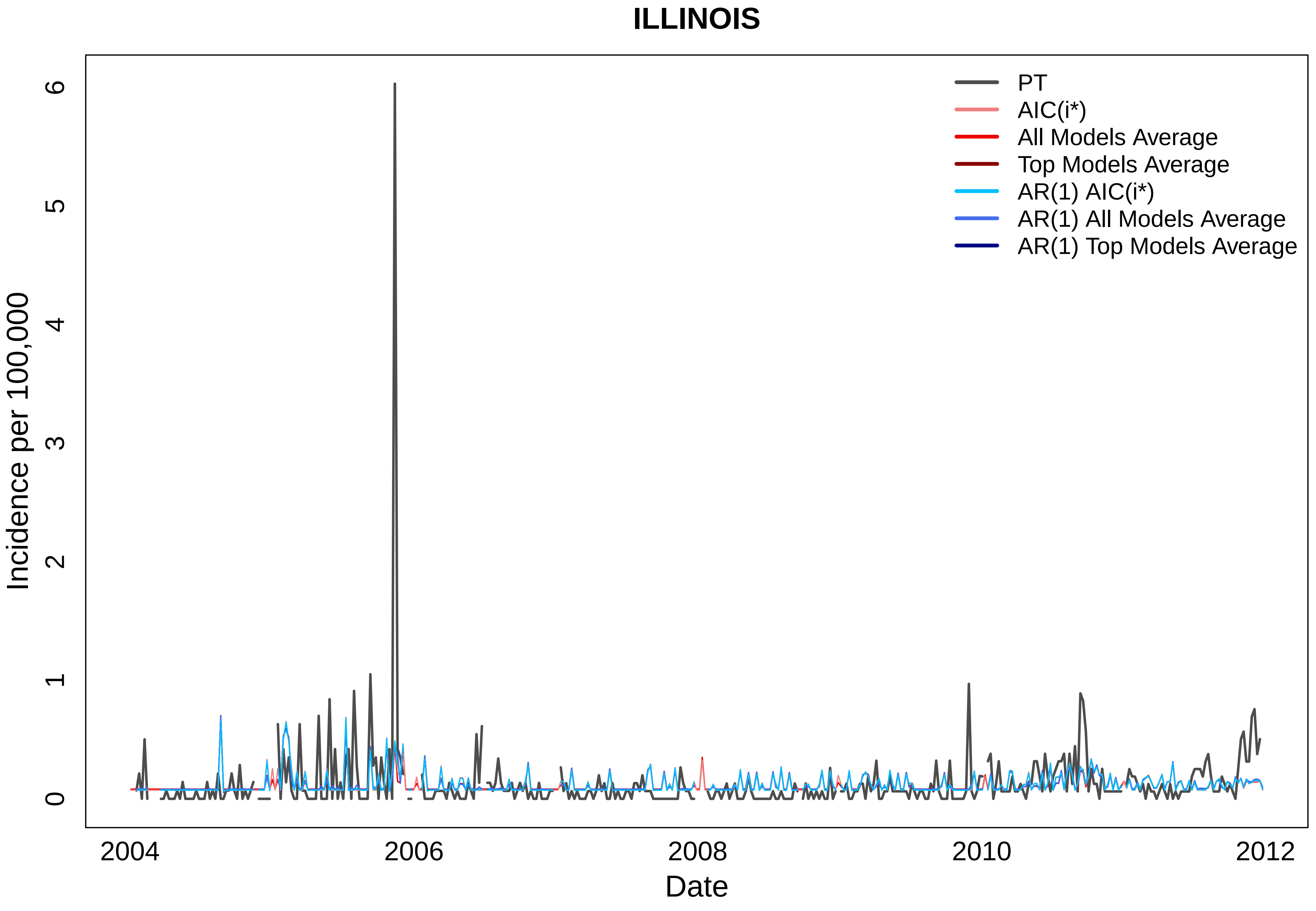

ILLINOIS
Incidence per 100,000
0
1
2
3
4
5
6
Date
2004
2006
2008
2010
2012
PT
AIC(i*)
All Models Average
Top Models Average
AR(1) AIC(i*)
AR(1) All Models Average
AR(1) Top Models Average

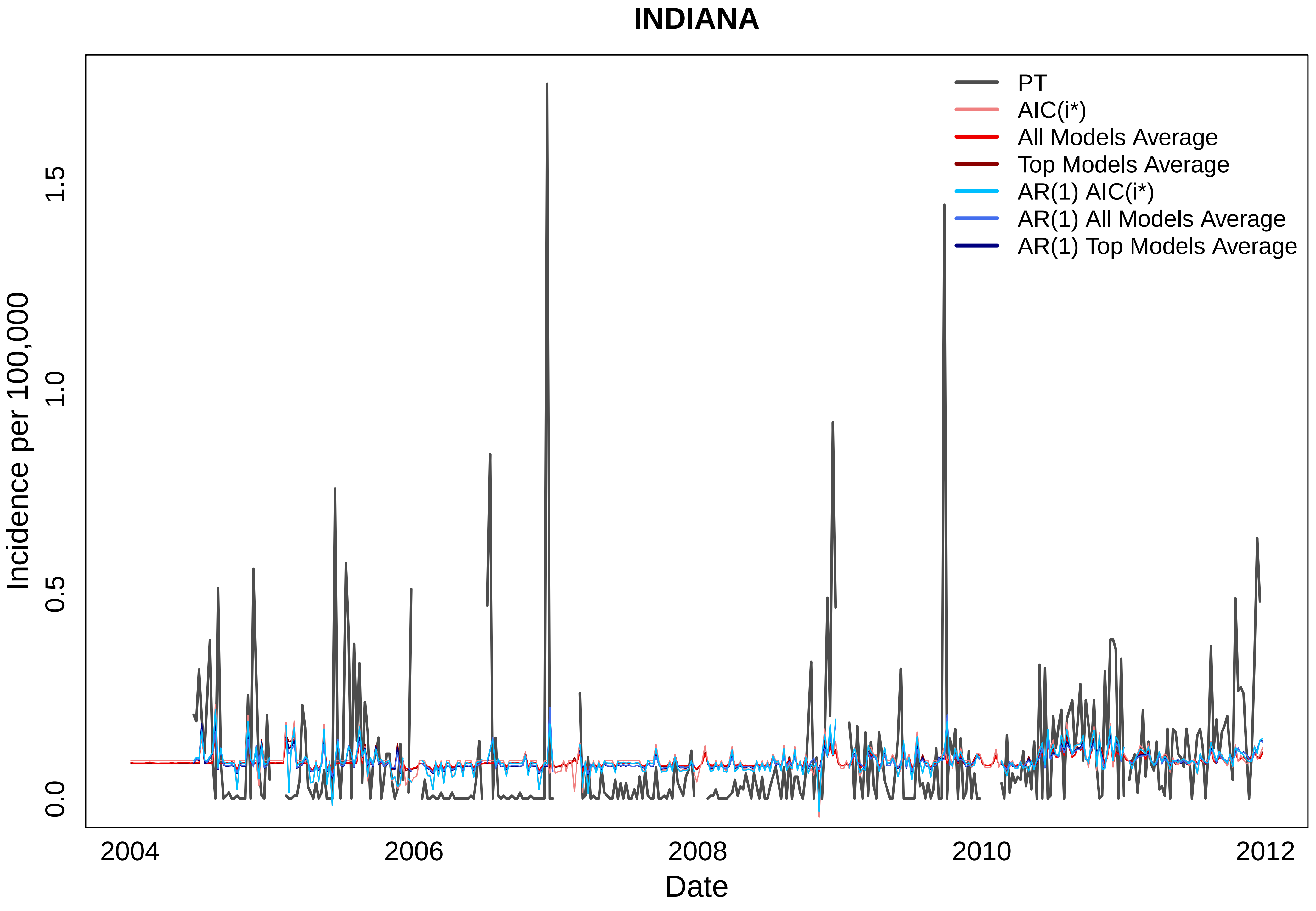
INDIANA
PT
AIC(i*)
All Models Average
Top Models Average
AR(1) AIC(i*)
AR(1) All Models Average
AR(1) Top Models Average
Incidence per 100,000
0.0
0.5
1.0
1.5
2004
2006
2008
2010
2012
Date

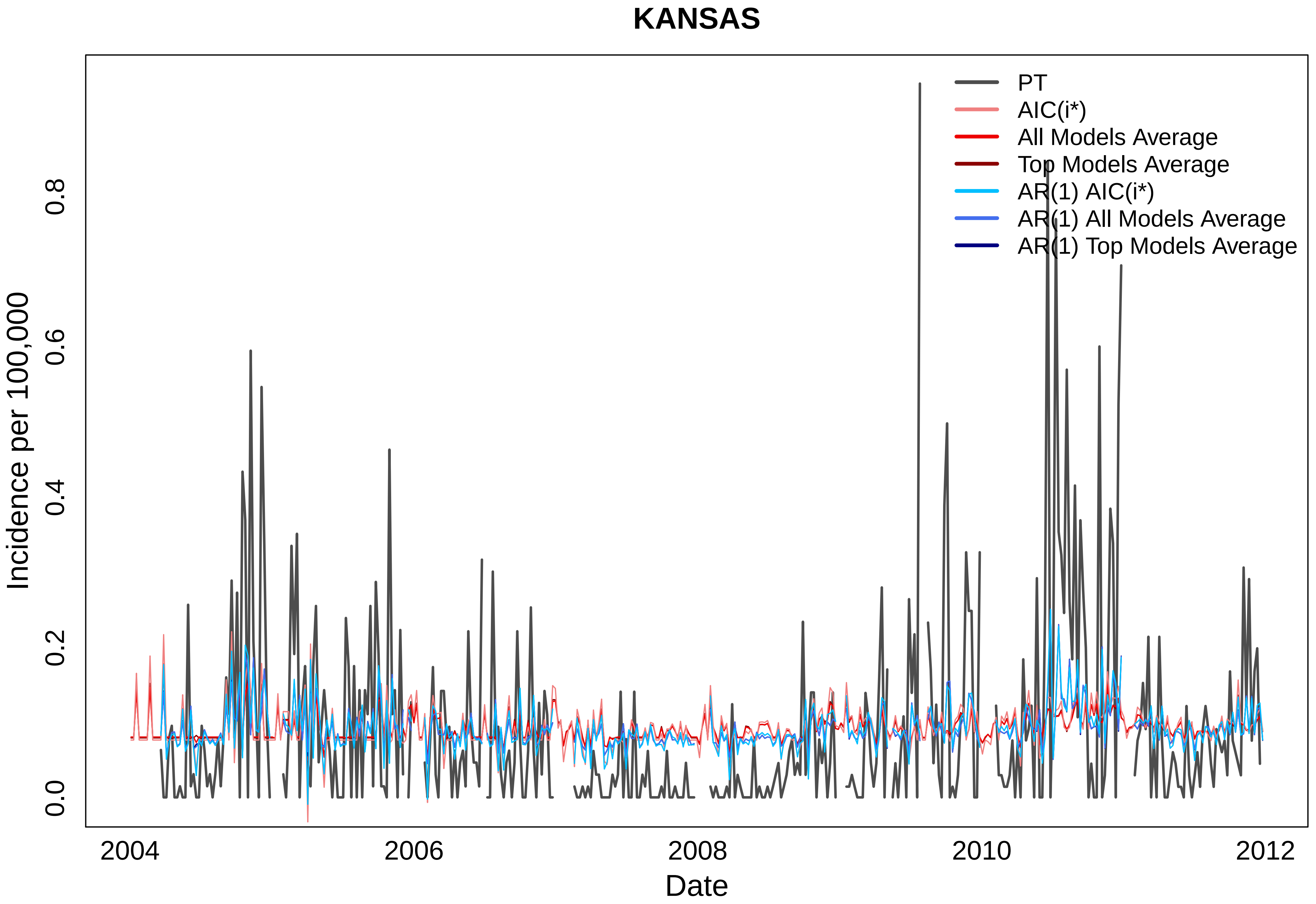

KANSAS
PT
AIC(i*)
All Models Average
Top Models Average
AR(1) AIC(i*)
AR(1) All Models Average
AR(1) Top Models Average
Incidence per 100,000
0.0
0.2
0.4
0.6
0.8
2004
2006
2008
2010
2012
Date

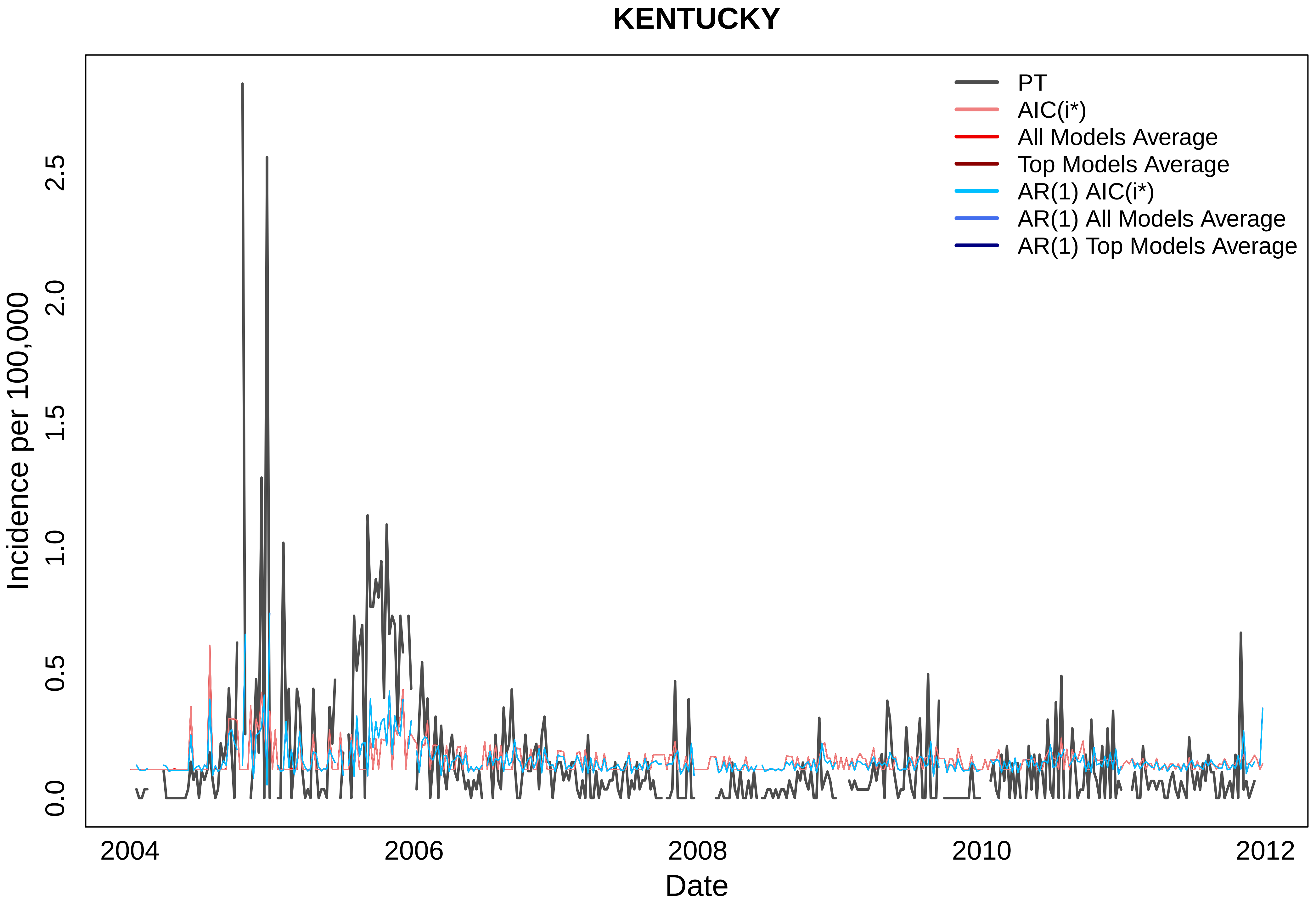
KENTUCKY
PT
AIC(i*)
All Models Average
Top Models Average
AR(1) AIC(i*)
AR(1) All Models Average
AR(1) Top Models Average
Incidence per 100,000
0.0
0.5
1.0
1.5
2.0
2.5
2004
2006
2008
2010
2012
Date

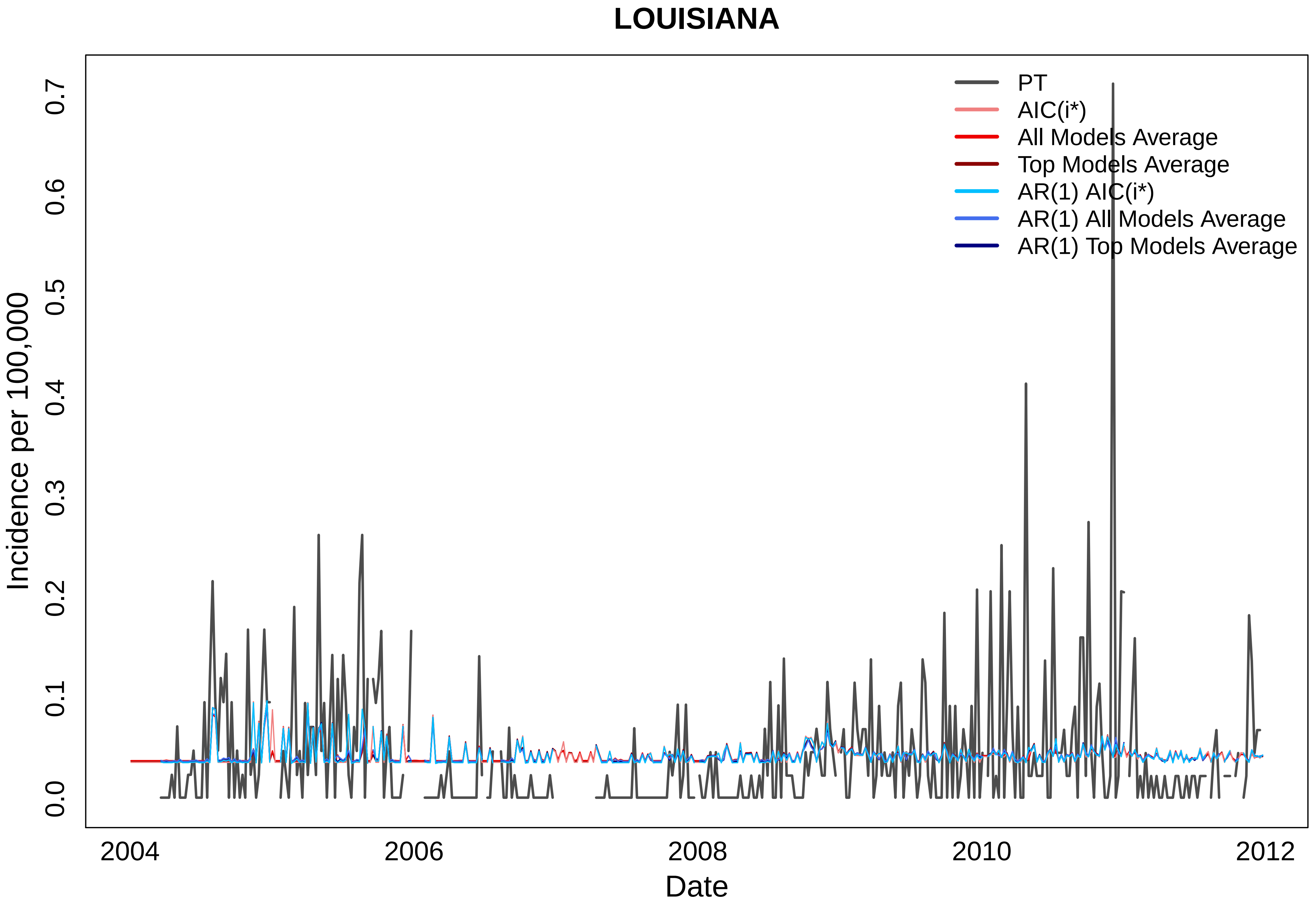
LOUISIANA
PT
AIC(i*)
All Models Average
Top Models Average
AR(1) AIC(i*)
AR(1) All Models Average
AR(1) Top Models Average
Incidence per 100,000
0.0
0.1
0.2
0.3
0.4
0.5
0.6
0.7
2004
2006
2008
2010
2012
Date

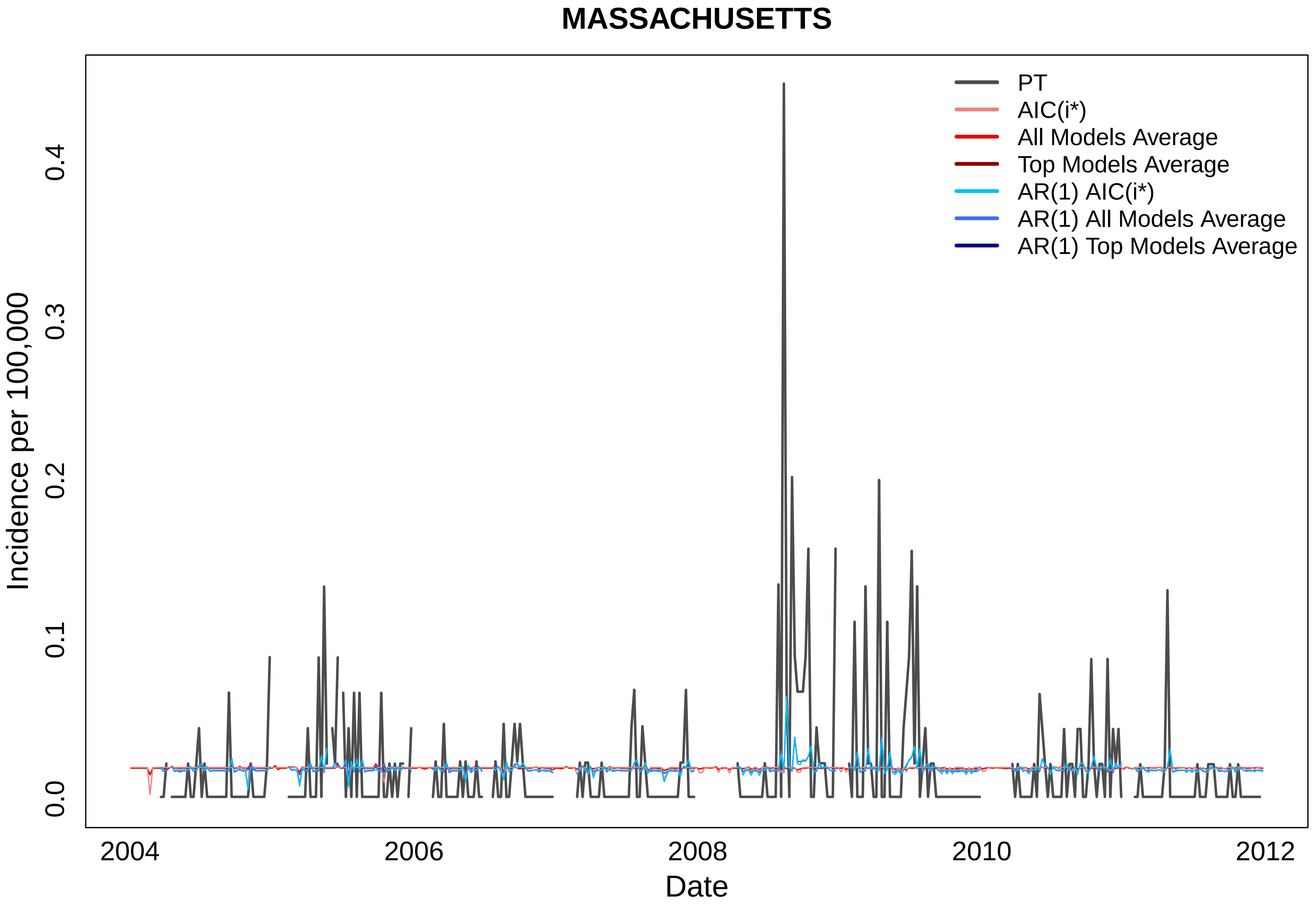
MASSACHUSETTS
PT
AIC(i*)
All Models Average
Top Models Average
AR(1) AIC(i*)
AR(1) All Models Average
AR(1) Top Models Average
Incidence per 100,000
0.0
0.1
0.2
0.3
0.4
2004
2006
2008
2010
2012
Date

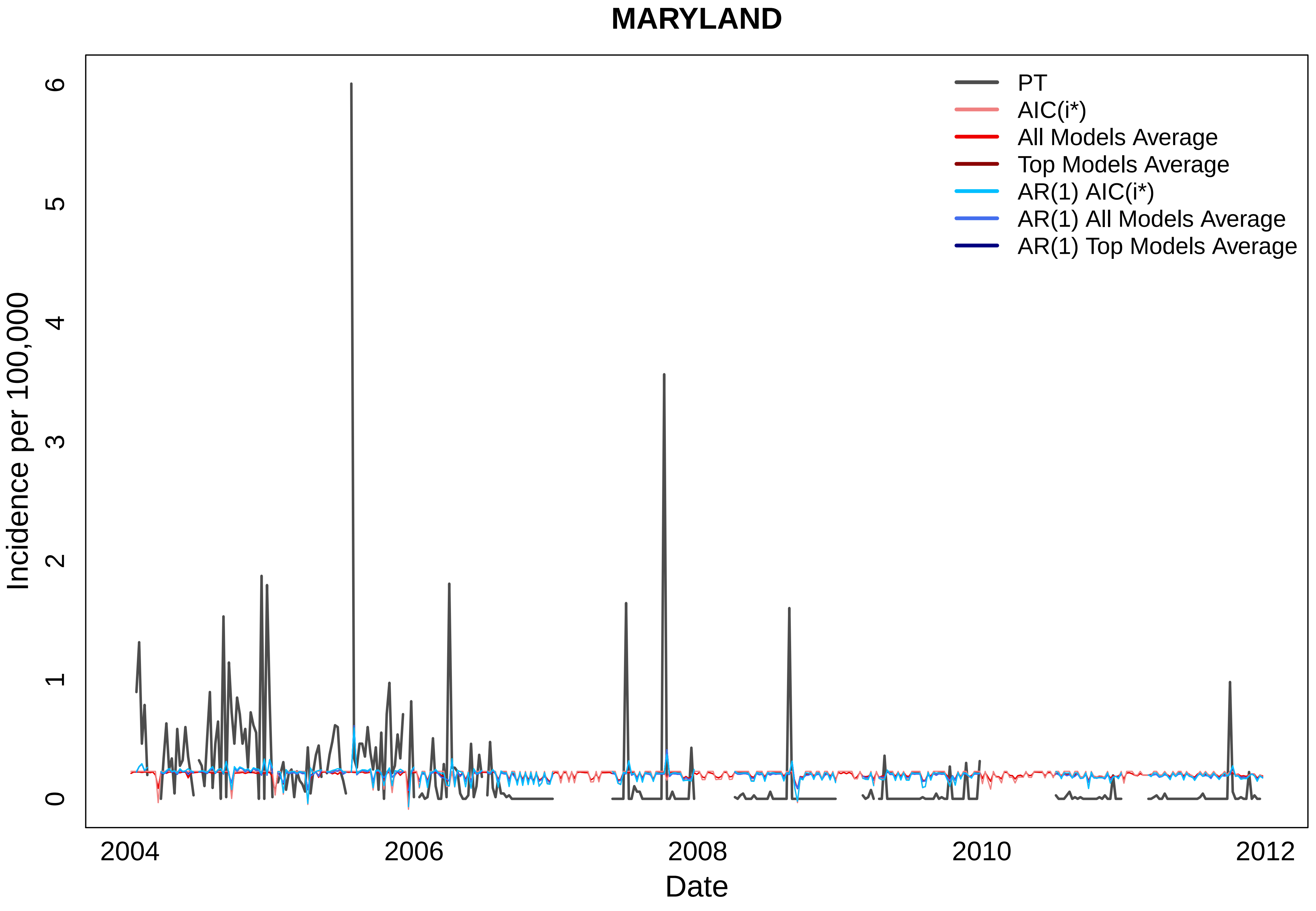
MARYLAND
PT
AIC(i*)
All Models Average
Top Models Average
AR(1) AIC(i*)
AR(1) All Models Average
AR(1) Top Models Average
Incidence per 100,000
0
1
2
3
4
5
6
2004
2006
2008
2010
2012
Date

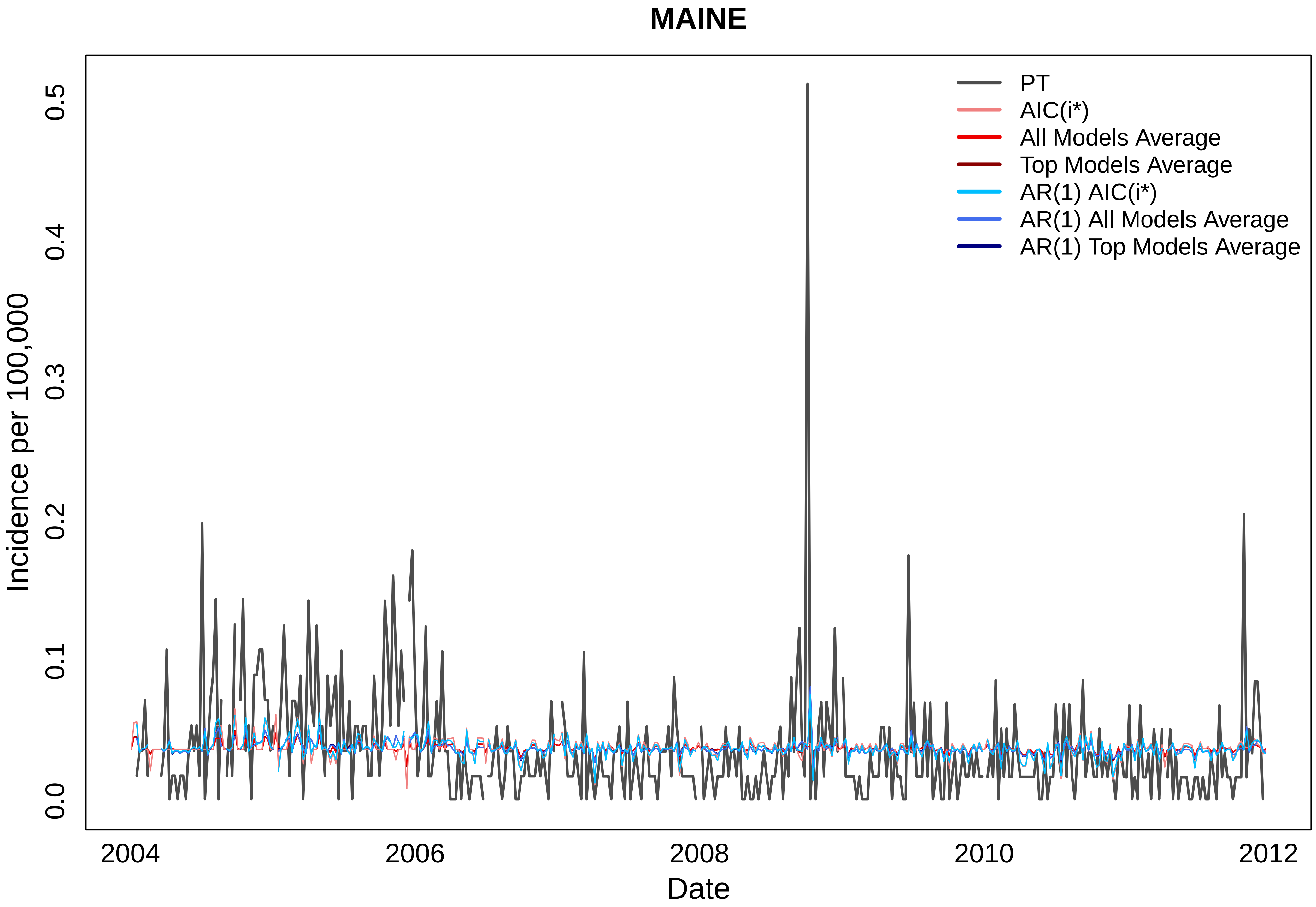

MAINE
PT
AIC(i*)
All Models Average
Top Models Average
AR(1) AIC(i*)
AR(1) All Models Average
AR(1) Top Models Average
Incidence per 100,000
0.0
0.1
0.2
0.3
0.4
0.5
2004
2006
2008
2010
2012
Date

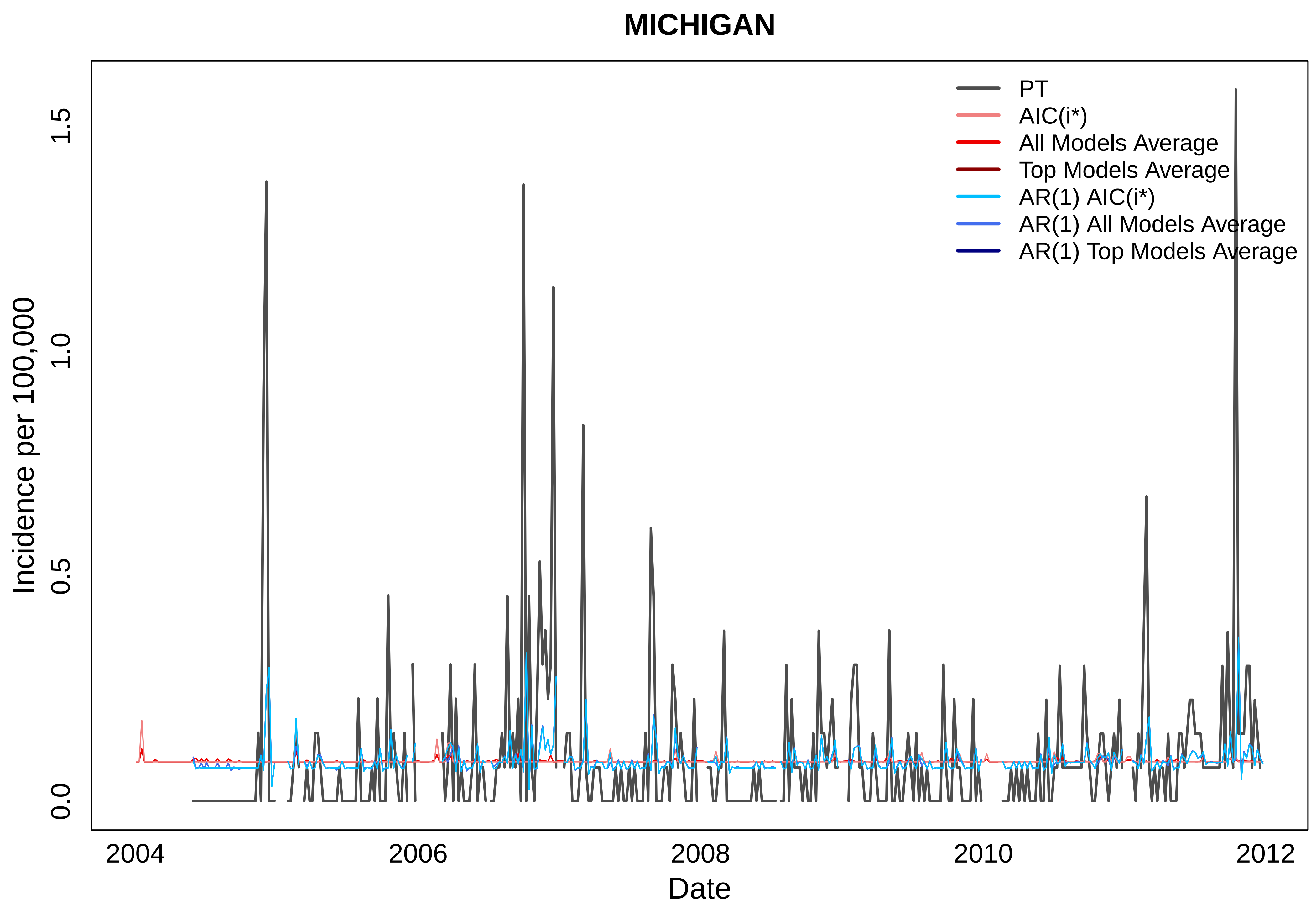
MICHIGAN
PT
AIC(i*)
All Models Average
Top Models Average
AR(1) AIC(i*)
AR(1) All Models Average
AR(1) Top Models Average
Incidence per 100,000
0.0
0.5
1.0
1.5
2004
2006
2008
2010
2012
Date

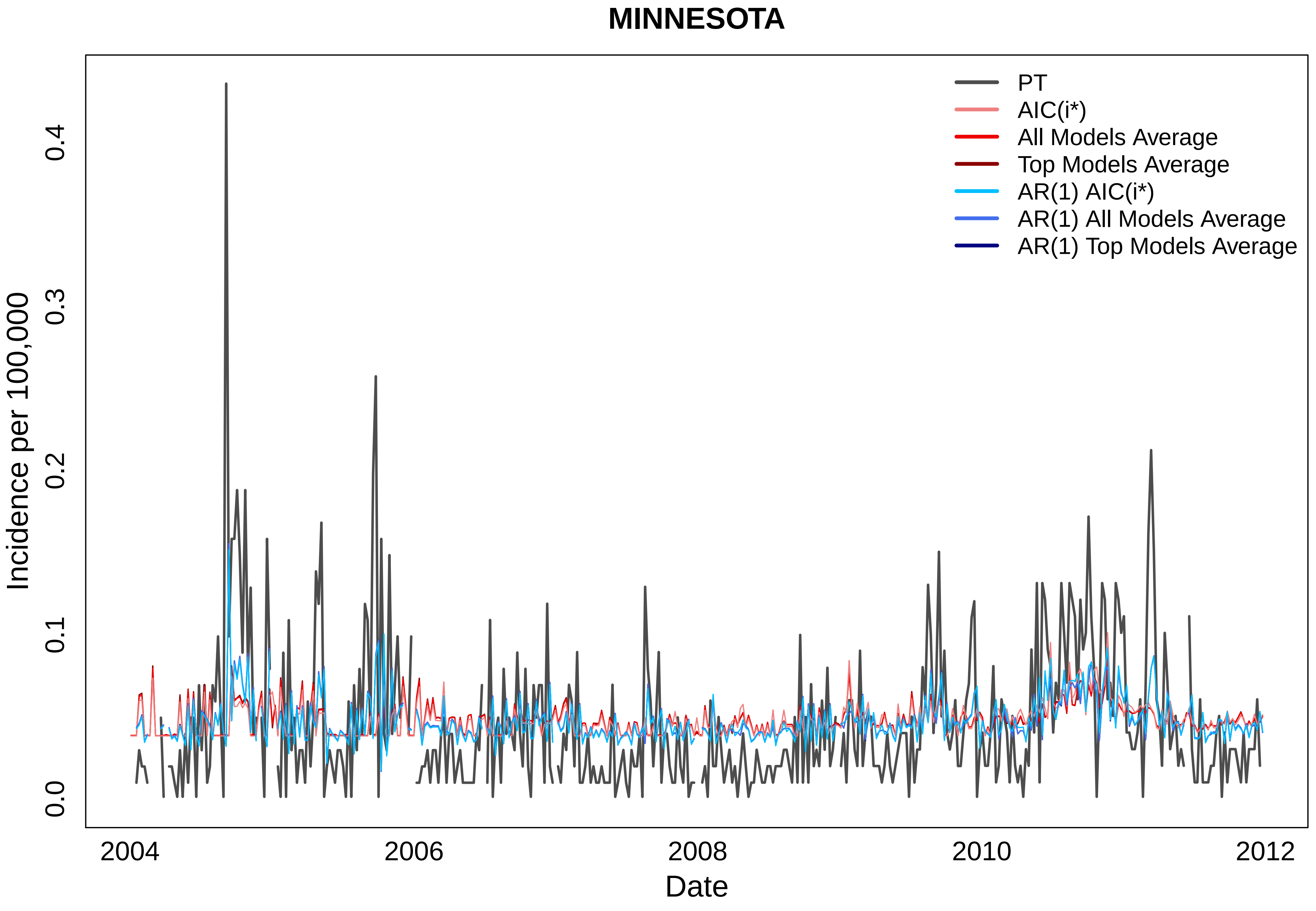
MINNESOTA
PT
AIC(i*)
All Models Average
Top Models Average
AR(1) AIC(i*)
AR(1) All Models Average
AR(1) Top Models Average
Incidence per 100,000
0.0
0.1
0.2
0.3
0.4
2004
2006
2008
2010
2012
Date

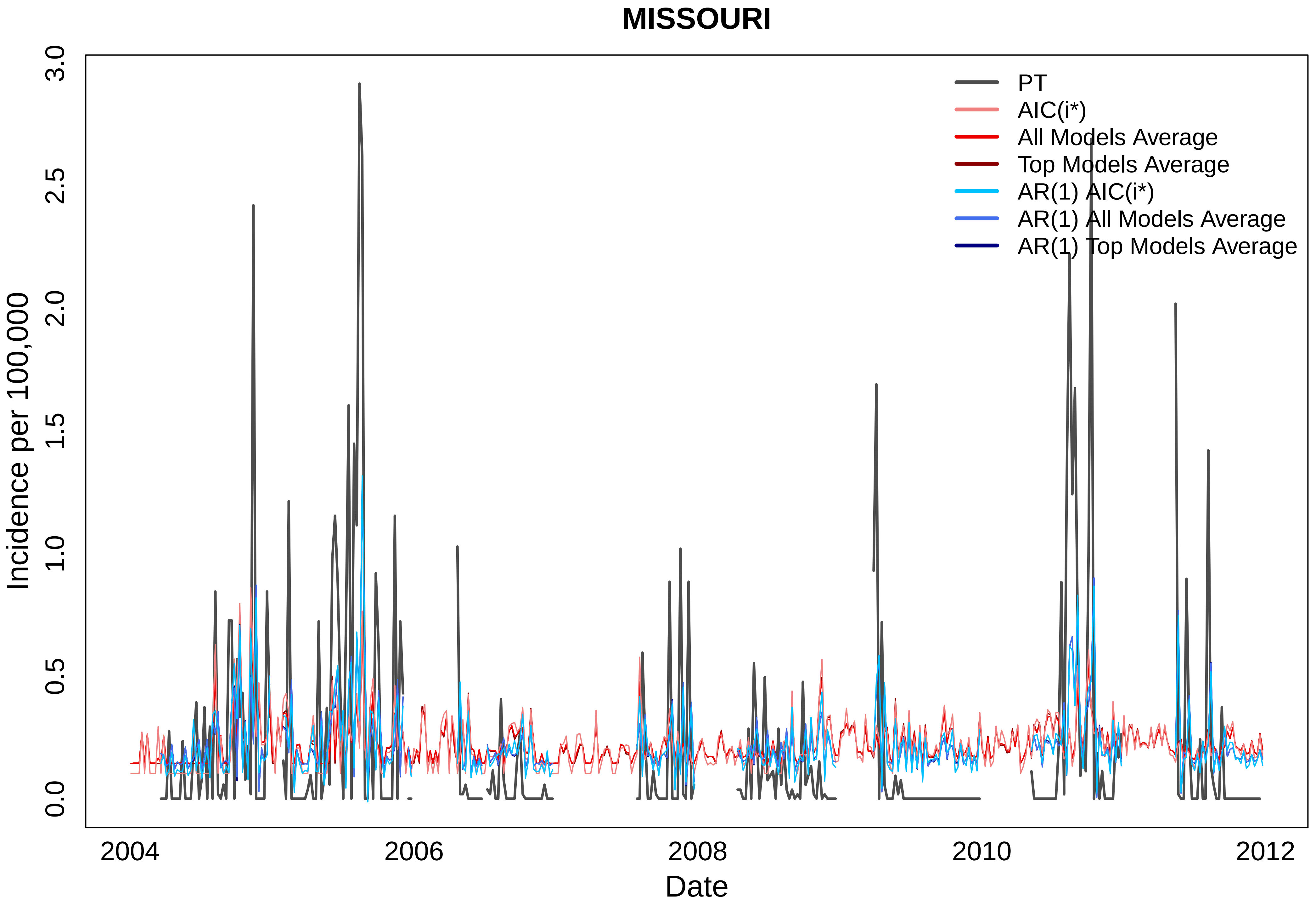
MISSOURI
PT
AIC(i*)
All Models Average
Top Models Average
AR(1) AIC(i*)
AR(1) All Models Average
AR(1) Top Models Average
Incidence per 100,000
0.0
0.5
1.0
1.5
2.0
2.5
3.0
2004
2006
2008
2010
2012
Date

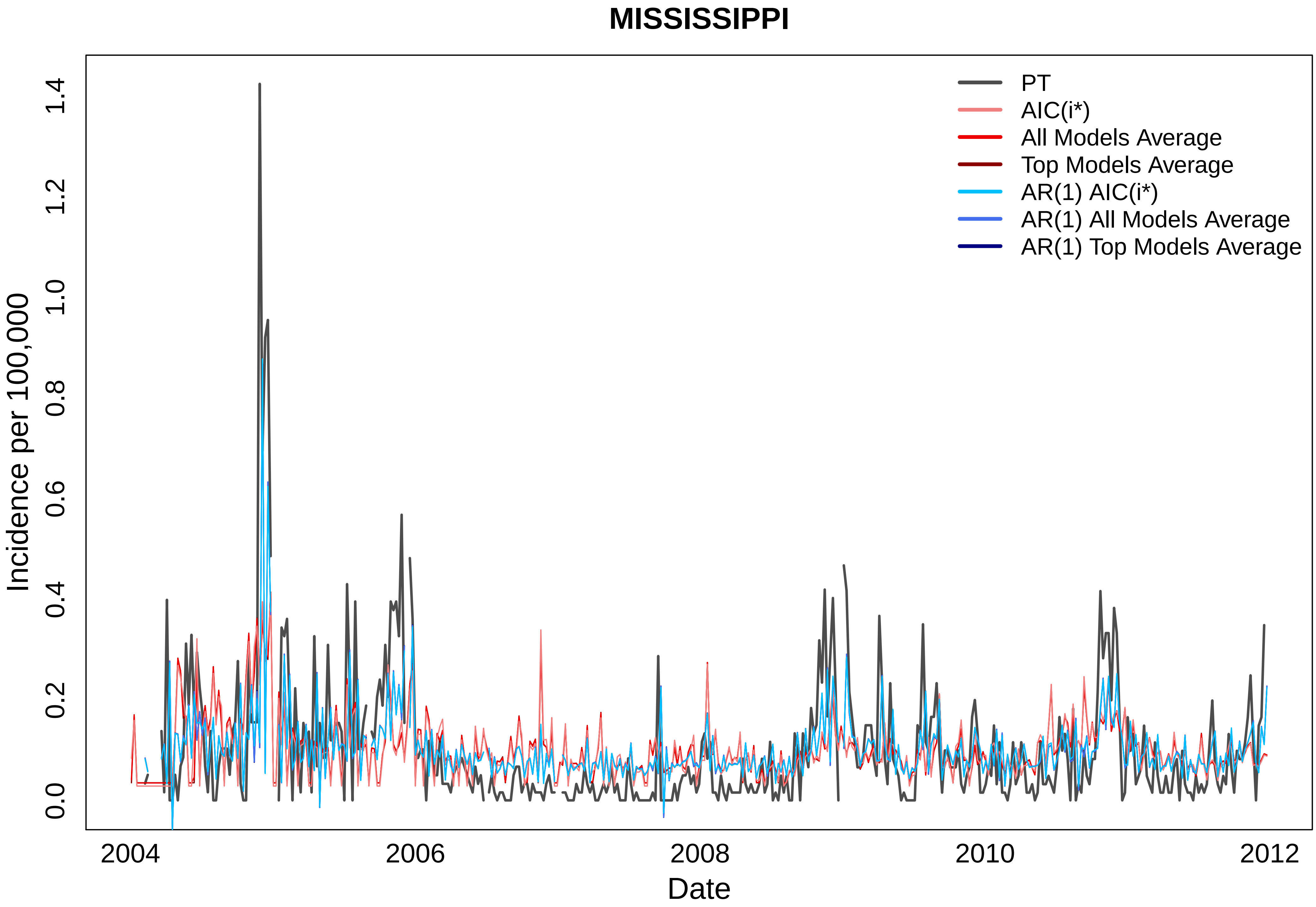
MISSISSIPPI
PT
AIC(i*)
All Models Average
Top Models Average
AR(1) AIC(i*)
AR(1) All Models Average
AR(1) Top Models Average
Incidence per 100,000
0.0
0.2
0.4
0.6
0.8
1.0
1.2
1.4
2004
2006
2008
2010
2012
Date

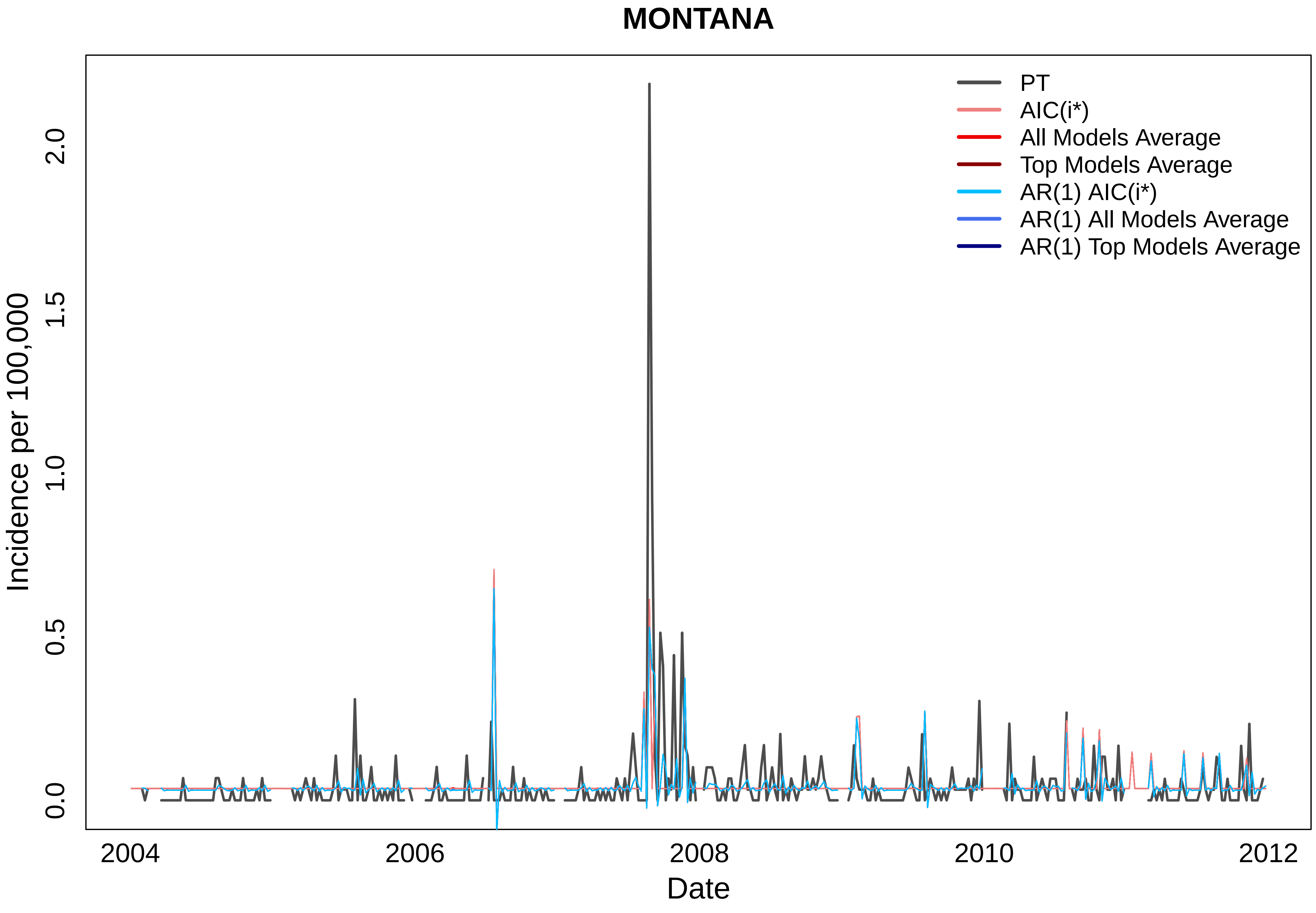
MONTANA
PT
AIC(i*)
All Models Average
Top Models Average
AR(1) AIC(i*)
AR(1) All Models Average
AR(1) Top Models Average
Incidence per 100,000
0.0
0.5
1.0
1.5
2.0
2004
2006
2008
2010
2012
Date

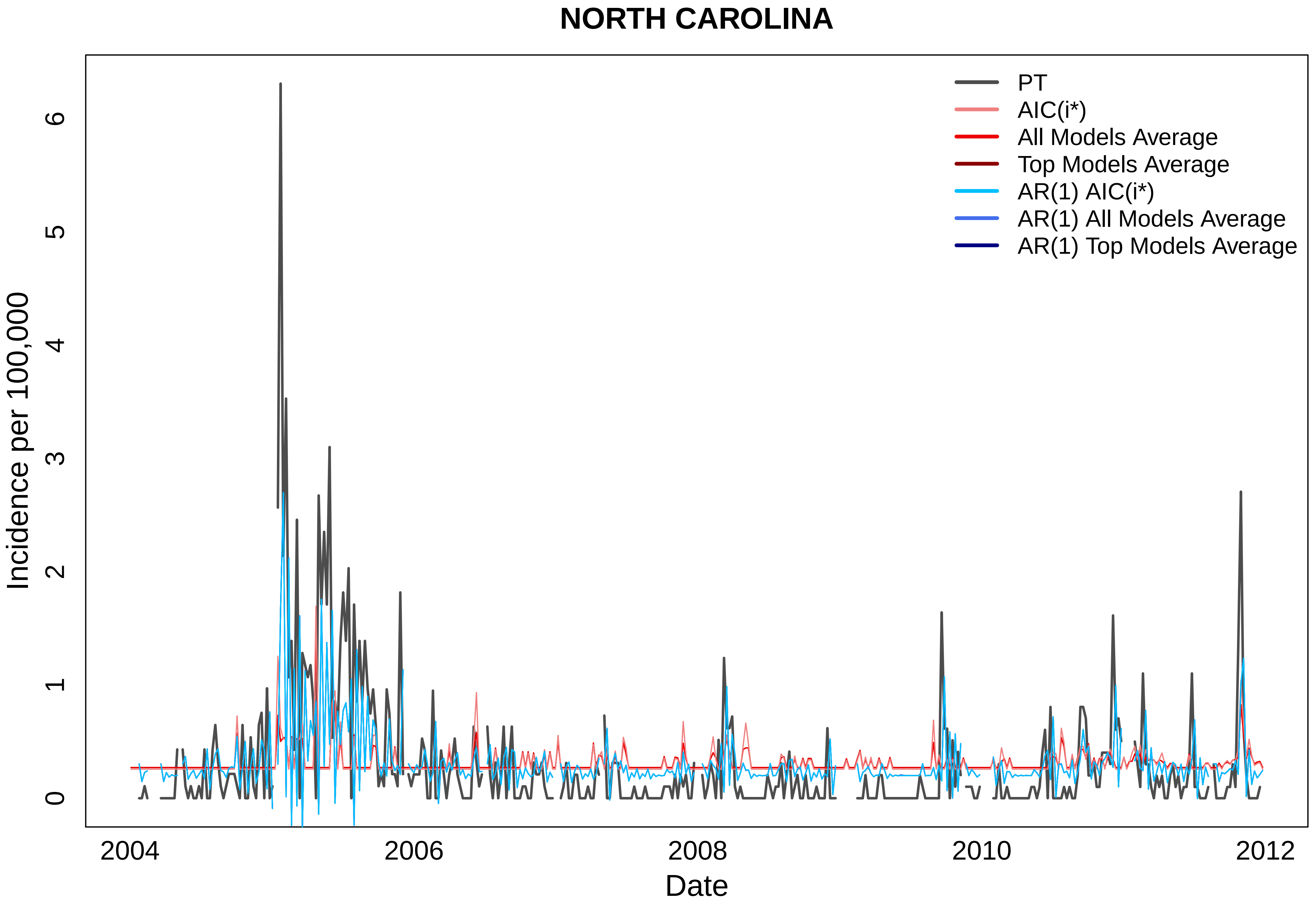

NORTH CAROLINA
PT
AIC(i*)
All Models Average
Top Models Average
AR(1) AIC(i*)
AR(1) All Models Average
AR(1) Top Models Average
Incidence per 100,000
0
1
2
3
4
5
6
2004
2006
2008
2010
2012
Date

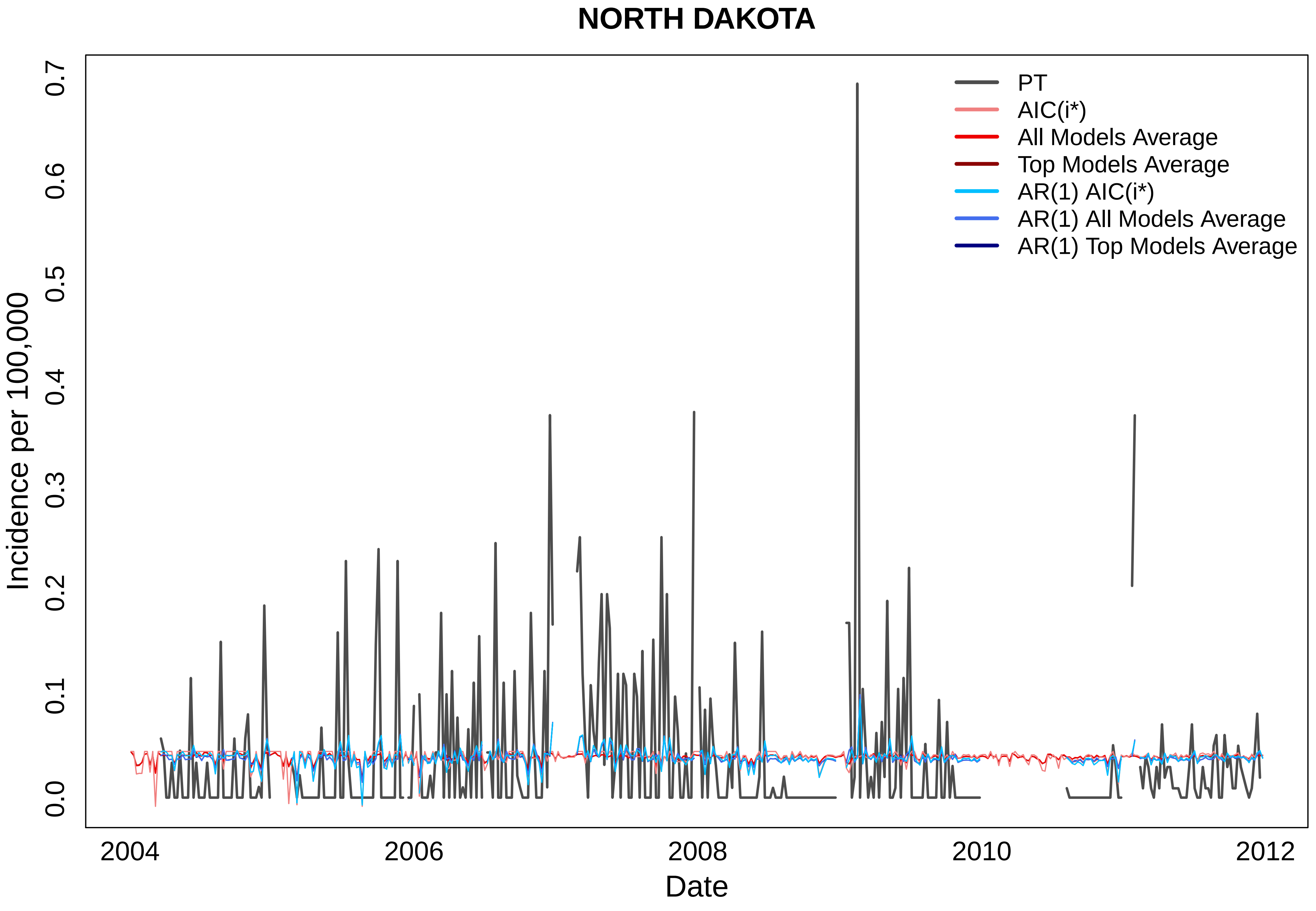
NORTH DAKOTA
PT
AIC(i*)
All Models Average
Top Models Average
AR(1) AIC(i*)
AR(1) All Models Average
AR(1) Top Models Average
Incidence per 100,000
0.0
0.1
0.2
0.3
0.4
0.5
0.6
0.7
2004
2006
2008
2010
2012
Date

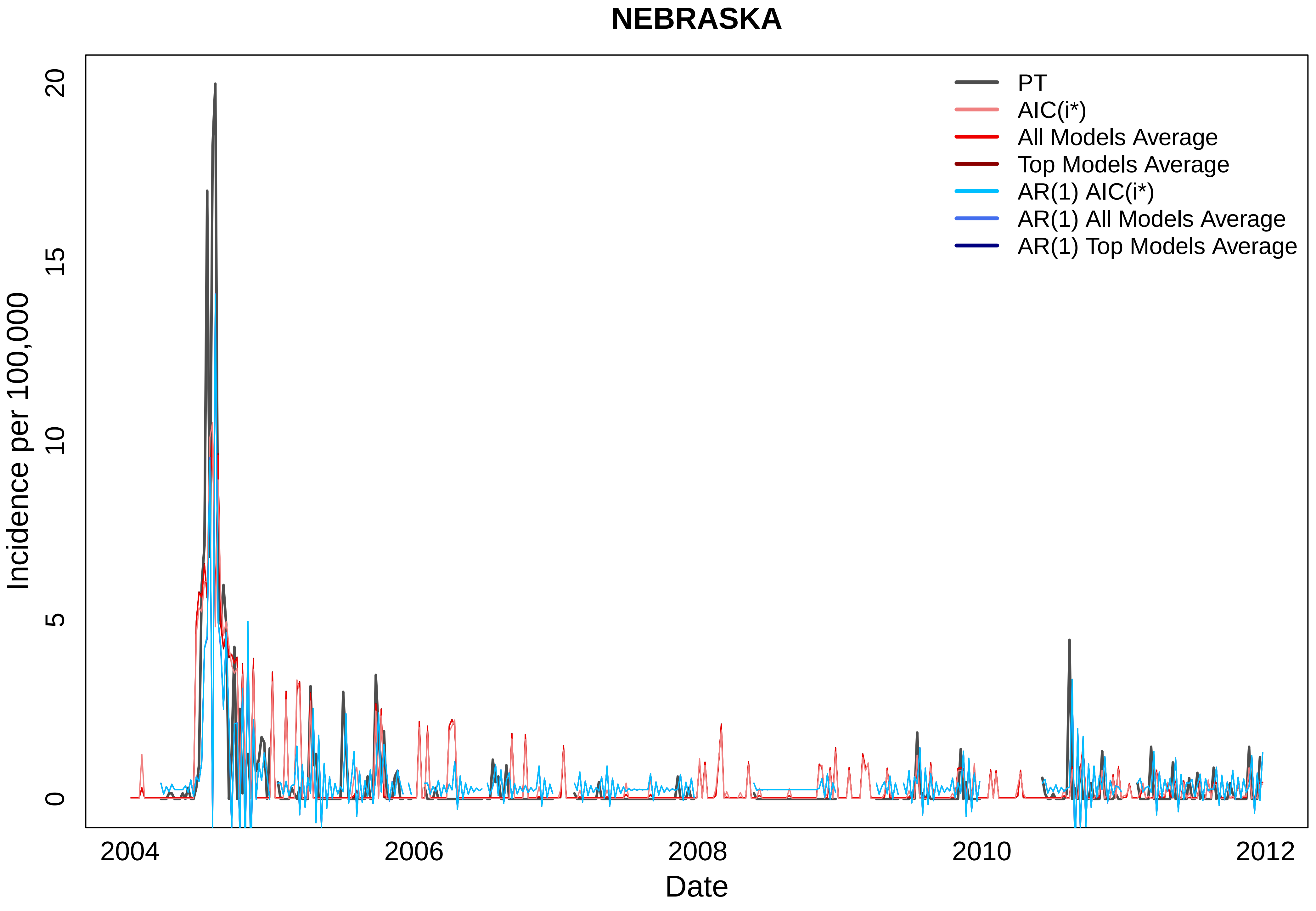
NEBRASKA
PT
AIC(i*)
All Models Average
Top Models Average
AR(1) AIC(i*)
AR(1) All Models Average
AR(1) Top Models Average
Incidence per 100,000
0
5
10
15
20
2004
2006
2008
2010
2012
Date

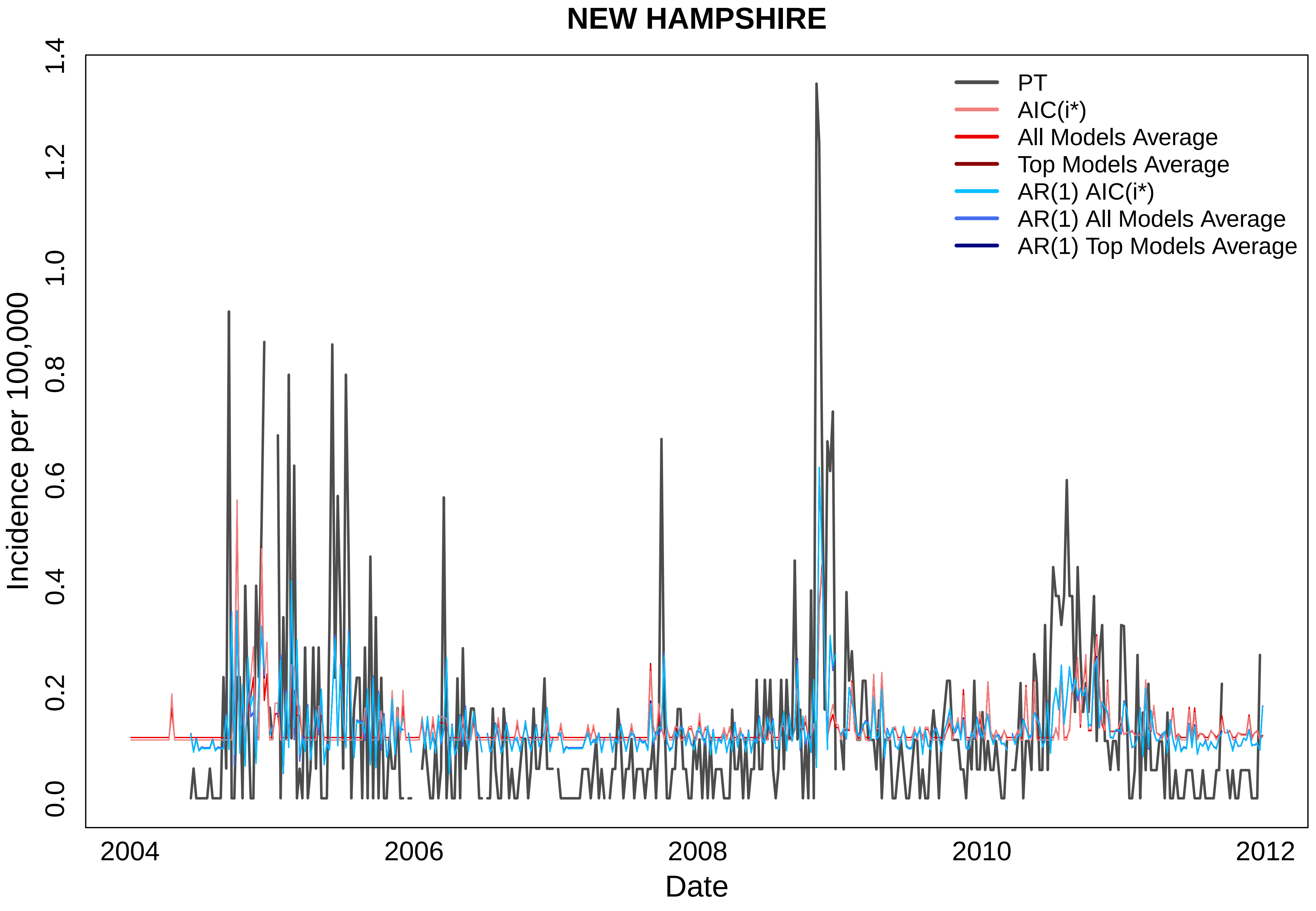

NEW HAMPSHIRE
Incidence per 100,000
0.0
0.2
0.4
0.6
0.8
1.0
1.2
1.4
Date
2004
2006
2008
2010
2012
PT
AIC(i*)
All Models Average
Top Models Average
AR(1) AIC(i*)
AR(1) All Models Average
AR(1) Top Models Average

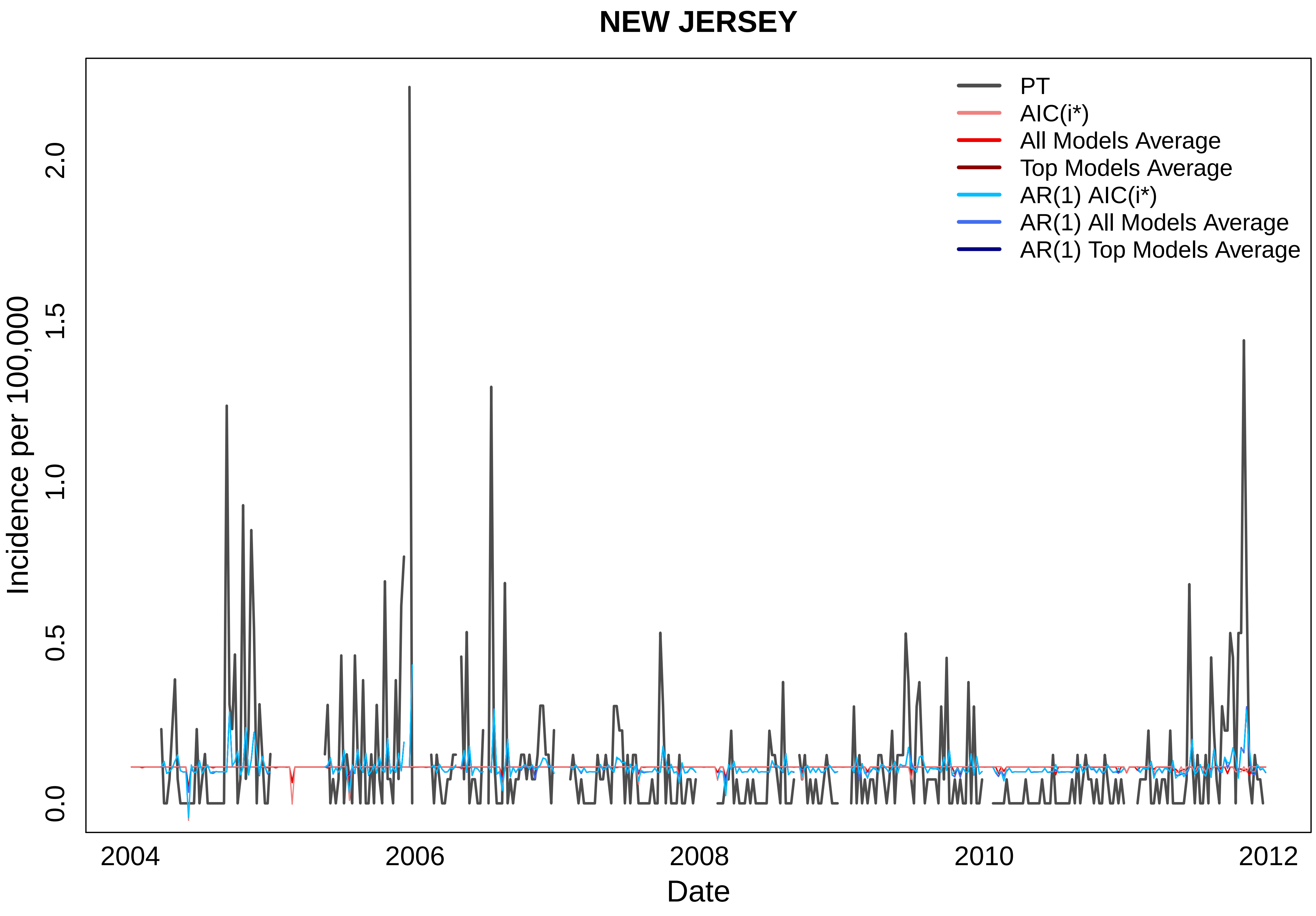

NEW JERSEY
PT
AIC(i*)
All Models Average
Top Models Average
AR(1) AIC(i*)
AR(1) All Models Average
AR(1) Top Models Average
Incidence per 100,000
0.0
0.5
1.0
1.5
2.0
2004
2006
2008
2010
2012
Date

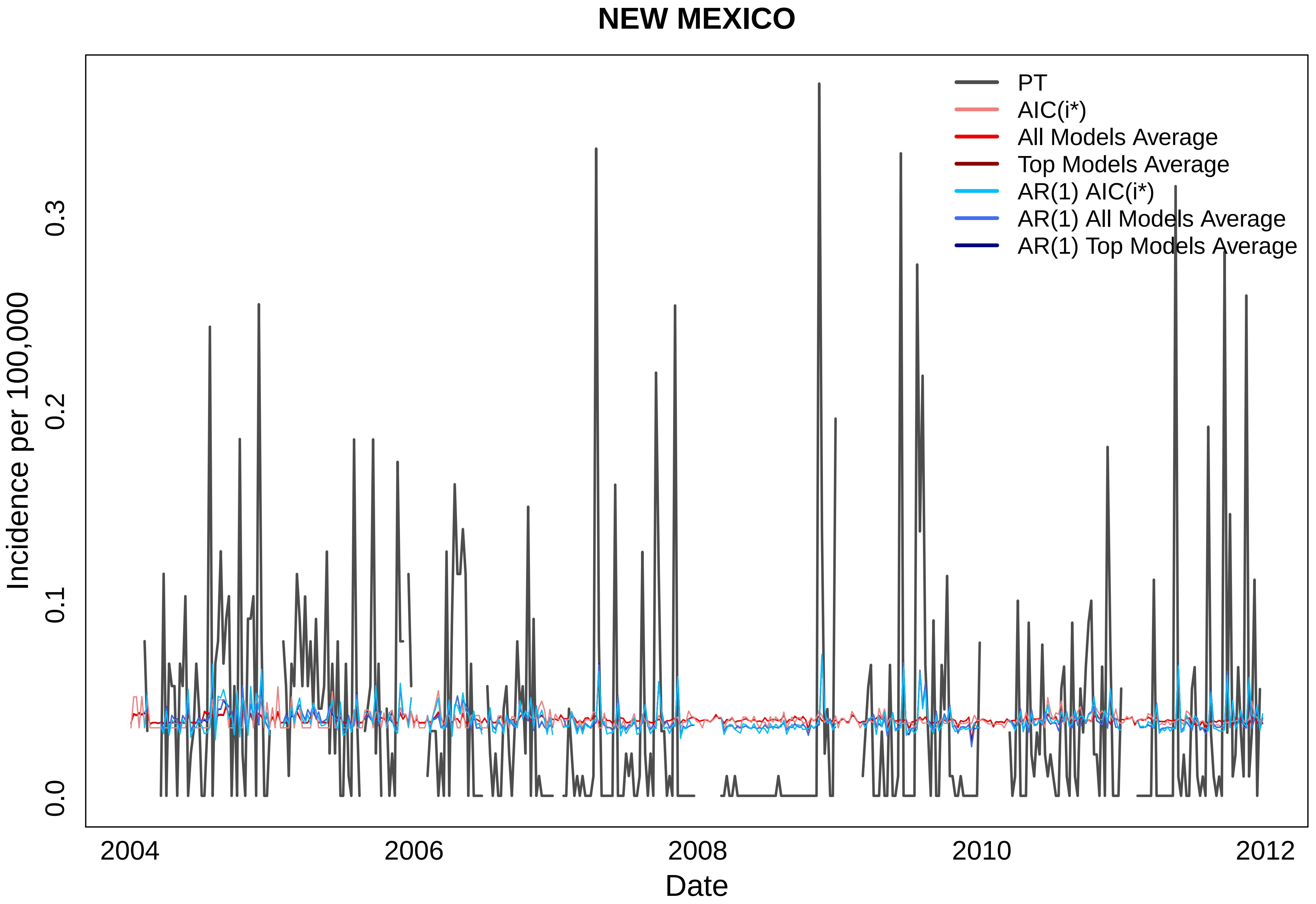
NEW MEXICO
PT
AIC(i*)
All Models Average
Top Models Average
AR(1) AIC(i*)
AR(1) All Models Average
AR(1) Top Models Average
Incidence per 100,000
0.0
0.1
0.2
0.3
2004
2006
2008
2010
2012
Date

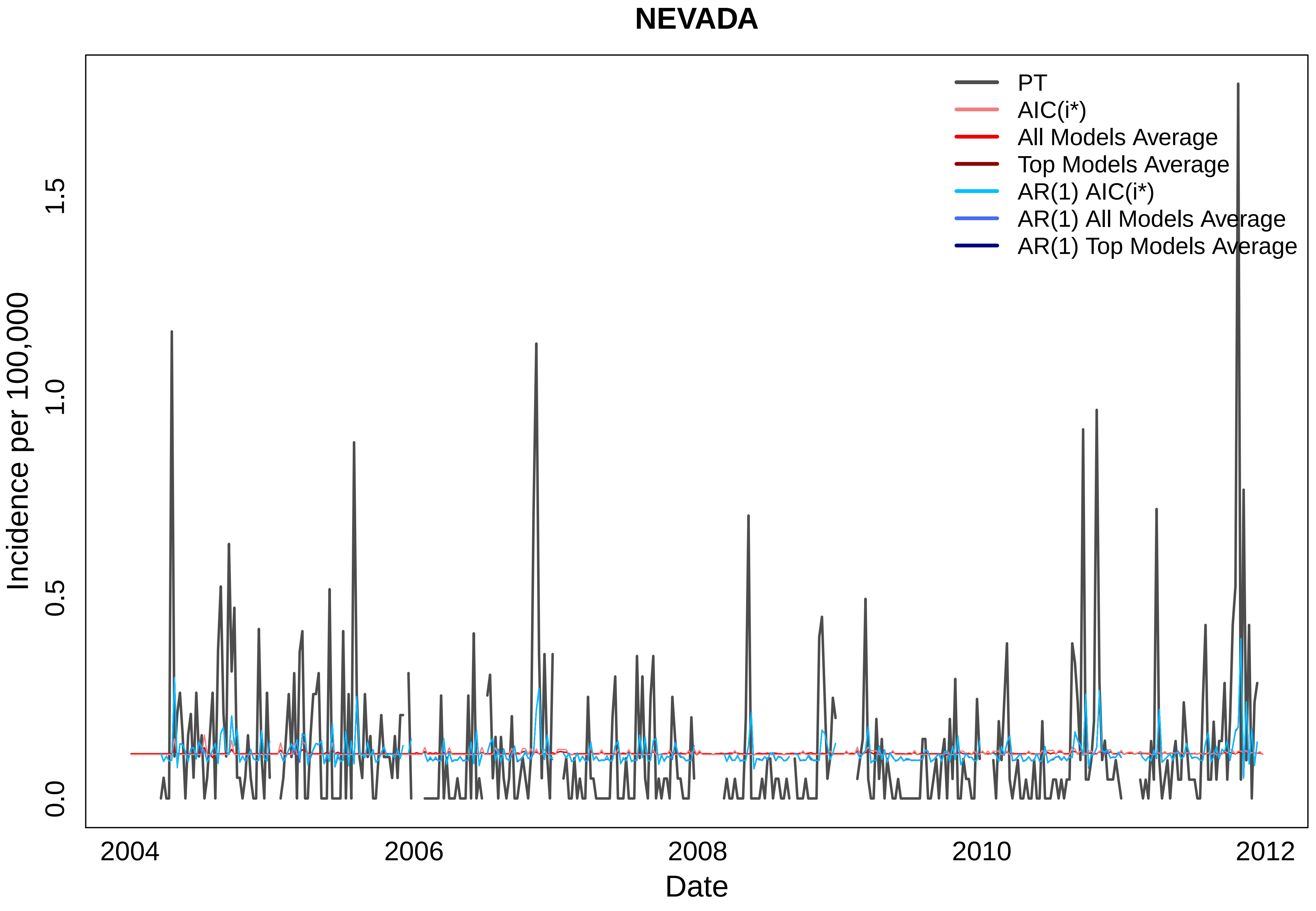
NEVADA
PT
AIC(i*)
All Models Average
Top Models Average
AR(1) AIC(i*)
AR(1) All Models Average
AR(1) Top Models Average
Incidence per 100,000
0.0
0.5
1.0
1.5
2004
2006
2008
2010
2012
Date

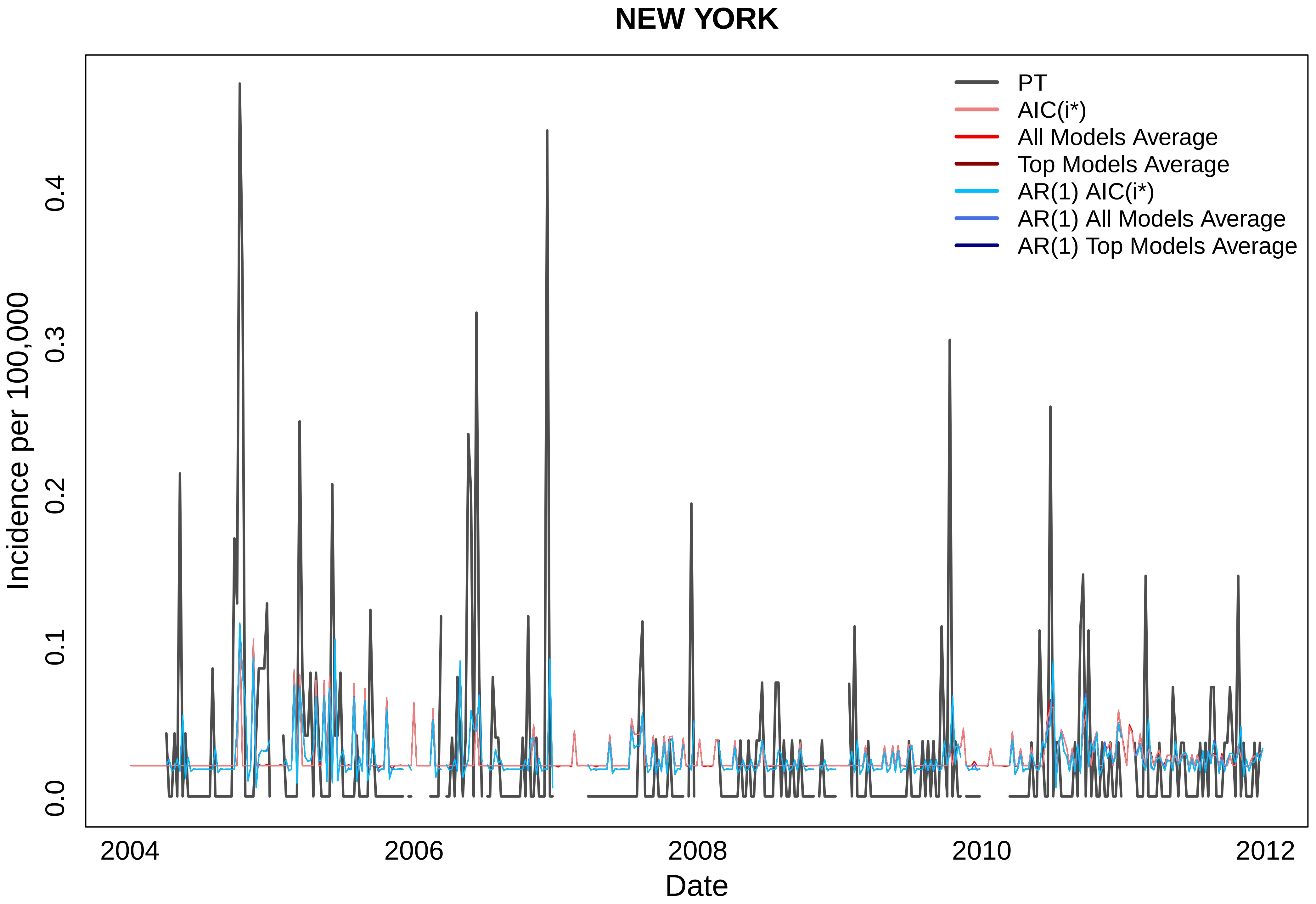
NEW YORK
PT
AIC(i*)
All Models Average
Top Models Average
AR(1) AIC(i*)
AR(1) All Models Average
AR(1) Top Models Average
Incidence per 100,000
0.0
0.1
0.2
0.3
0.4
2004
2006
2008
2010
2012
Date

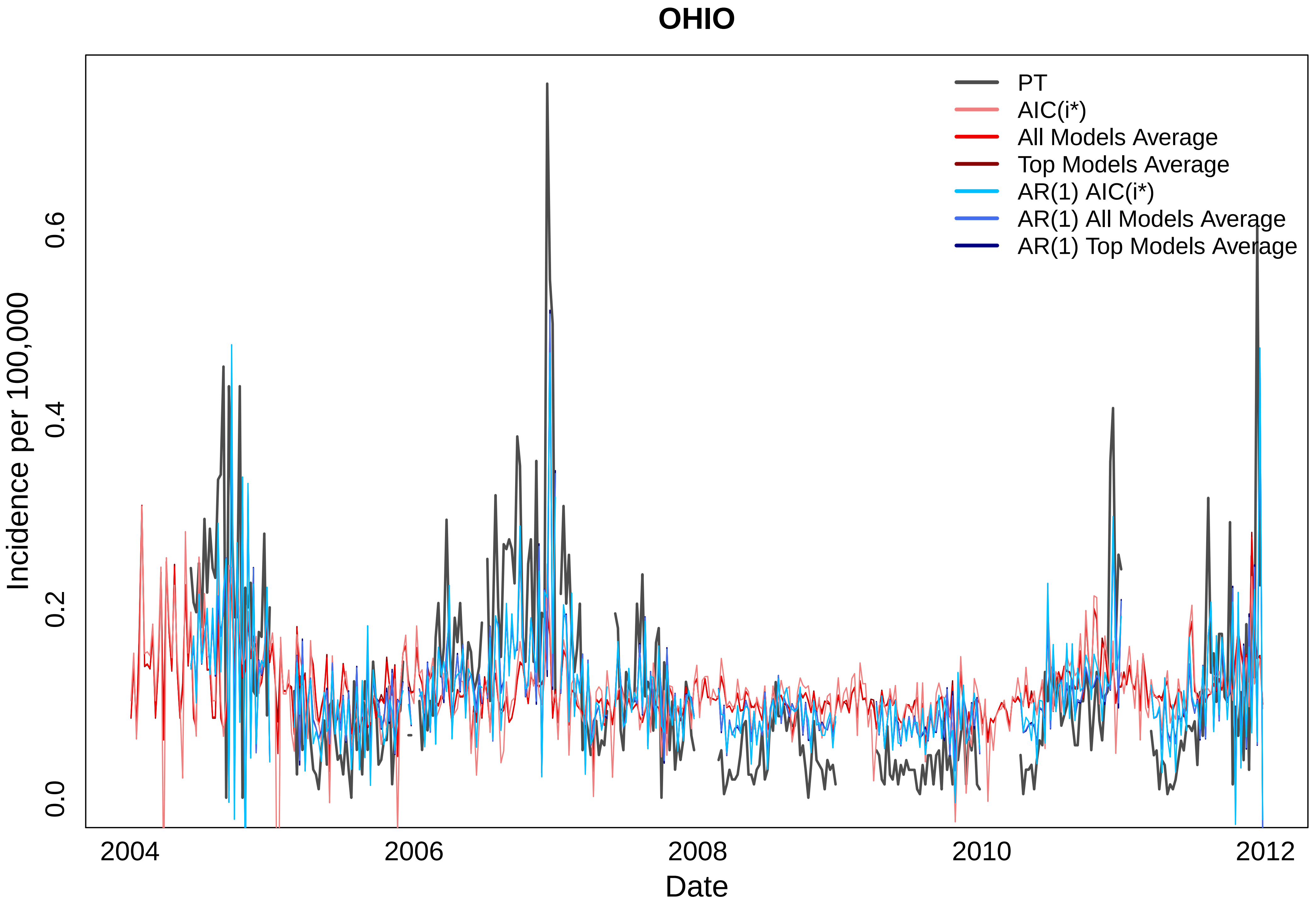
OHIO
PT
AIC(i*)
All Models Average
Top Models Average
AR(1) AIC(i*)
AR(1) All Models Average
AR(1) Top Models Average
Incidence per 100,000
0.0
0.2
0.4
0.6
2004
2006
2008
2010
2012
Date

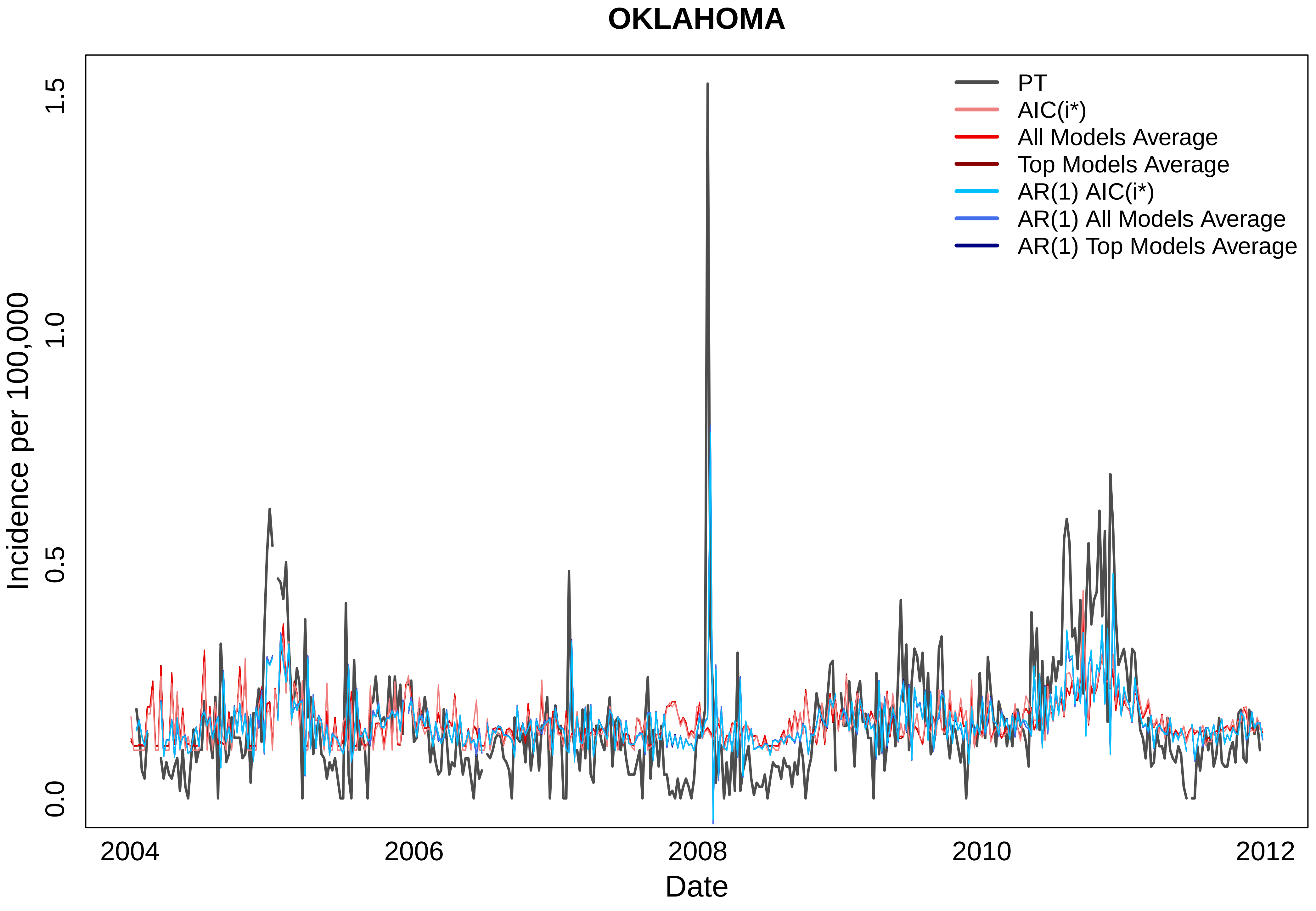

OKLAHOMA
PT
AIC(i*)
All Models Average
Top Models Average
AR(1) AIC(i*)
AR(1) All Models Average
AR(1) Top Models Average
Incidence per 100,000
0.0
0.5
1.0
1.5
2004
2006
2008
2010
2012
Date

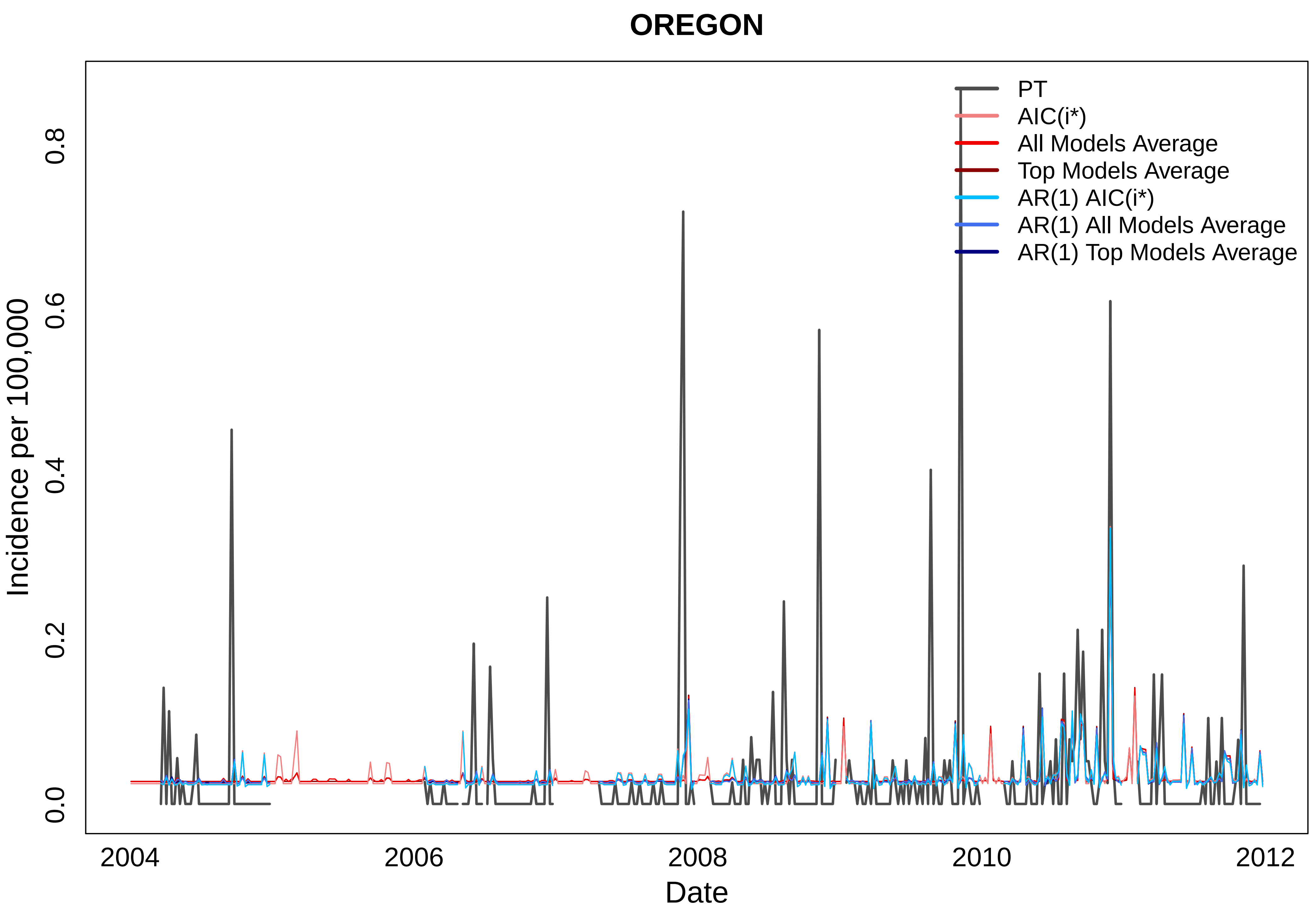
OREGON
PT
AIC(i*)
All Models Average
Top Models Average
AR(1) AIC(i*)
AR(1) All Models Average
AR(1) Top Models Average
Incidence per 100,000
0.0
0.2
0.4
0.6
0.8
2004
2006
2008
2010
2012
Date

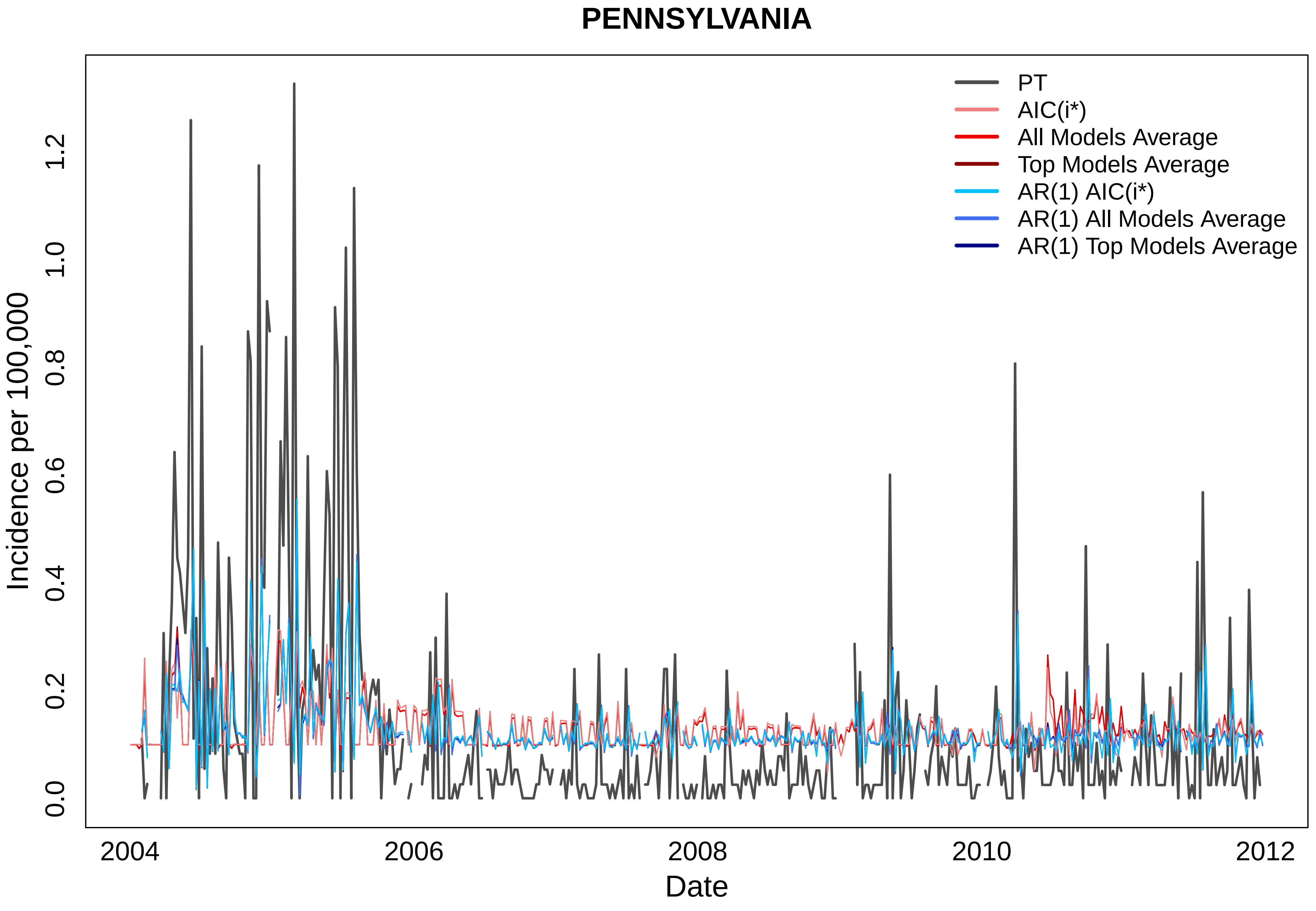
PENNSYLVANIA
PT
AIC(i*)
All Models Average
Top Models Average
AR(1) AIC(i*)
AR(1) All Models Average
AR(1) Top Models Average
Incidence per 100,000
0.0
0.2
0.4
0.6
0.8
1.0
1.2
2004
2006
2008
2010
2012
Date

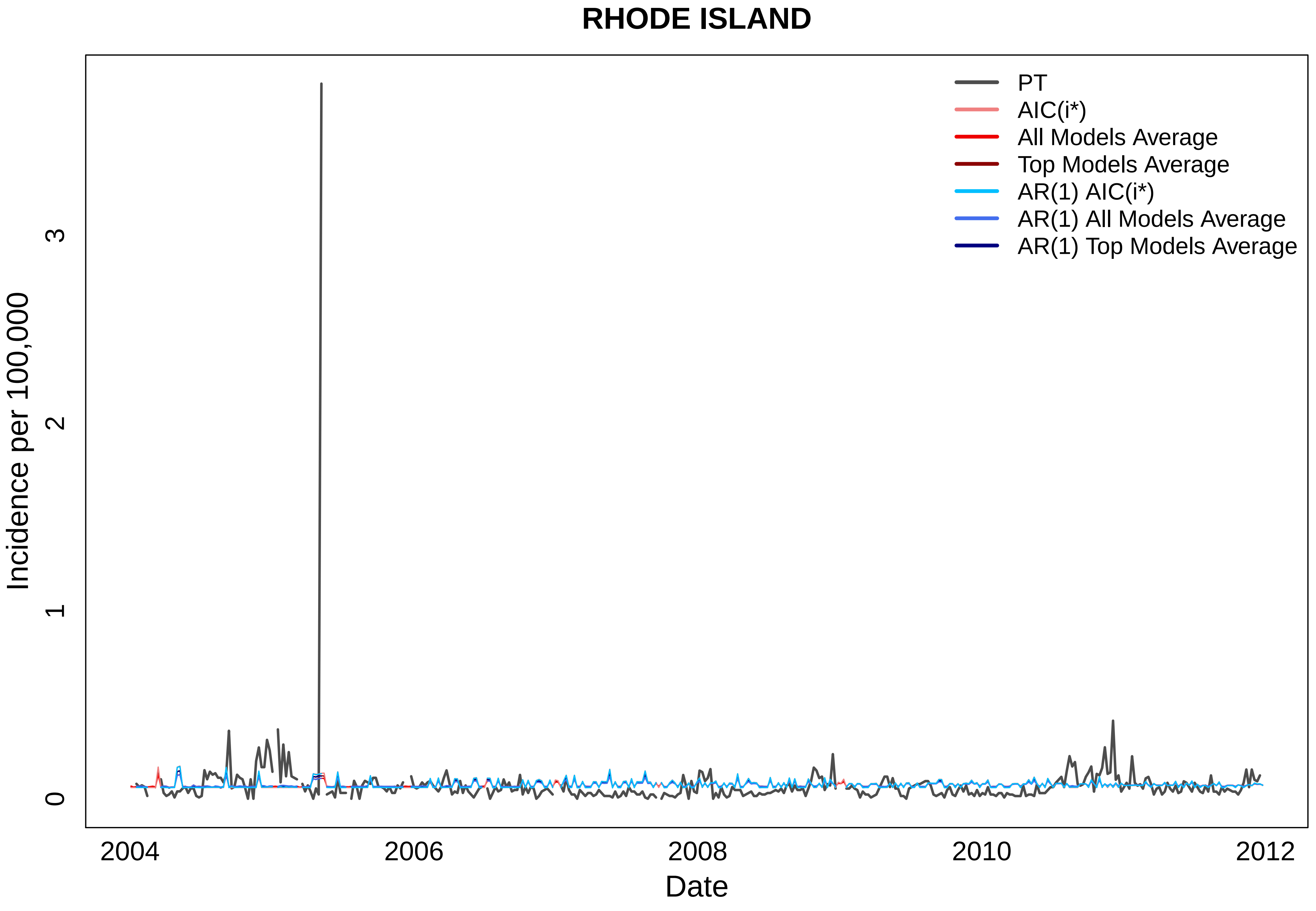

RHODE ISLAND
PT
AIC(i*)
All Models Average
Top Models Average
AR(1) AIC(i*)
AR(1) All Models Average
AR(1) Top Models Average
Incidence per 100,000
0
1
2
3
2004
2006
2008
2010
2012
Date

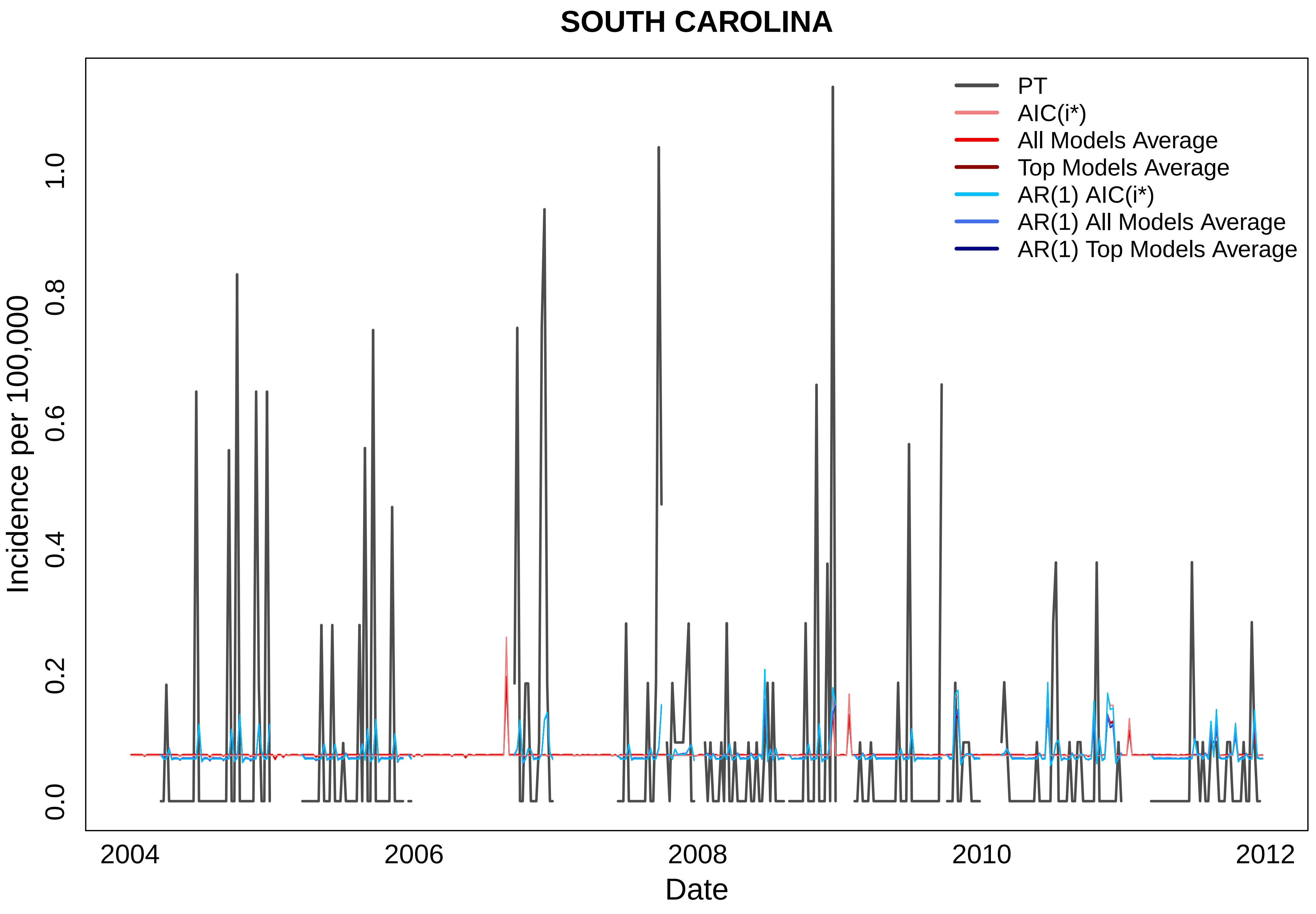

SOUTH CAROLINA
PT
AIC(i*)
All Models Average
Top Models Average
AR(1) AIC(i*)
AR(1) All Models Average
AR(1) Top Models Average
Incidence per 100,000
0.0
0.2
0.4
0.6
0.8
1.0
2004
2006
2008
2010
2012
Date

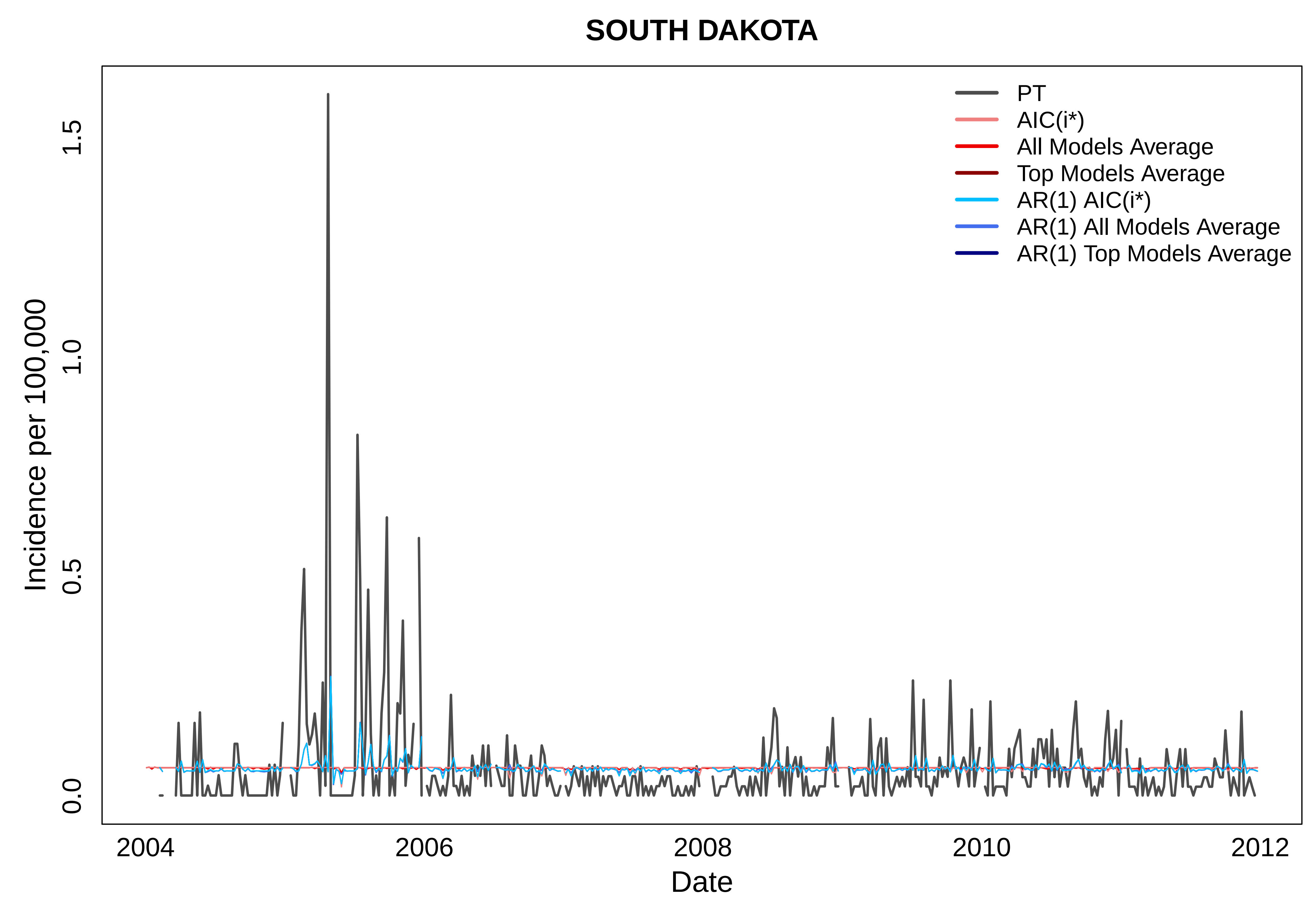

SOUTH DAKOTA
PT
AIC(i*)
All Models Average
Top Models Average
AR(1) AIC(i*)
AR(1) All Models Average
AR(1) Top Models Average
Incidence per 100,000
0.0
0.5
1.0
1.5
2004
2006
2008
2010
2012
Date

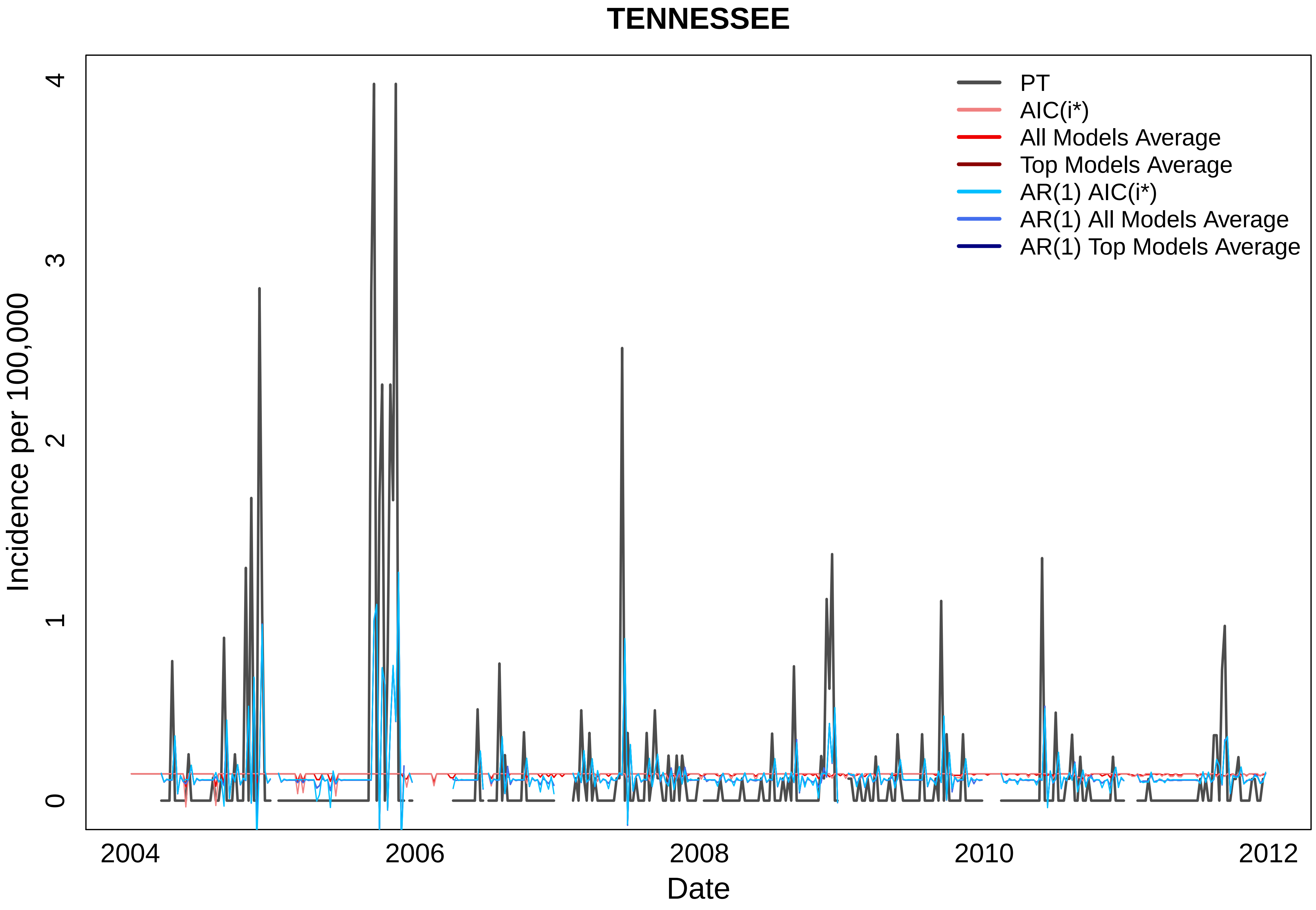

TENNESSEE
PT
AIC(i*)
All Models Average
Top Models Average
AR(1) AIC(i*)
AR(1) All Models Average
AR(1) Top Models Average
Incidence per 100,000
0
1
2
3
4
2004
2006
2008
2010
2012
Date

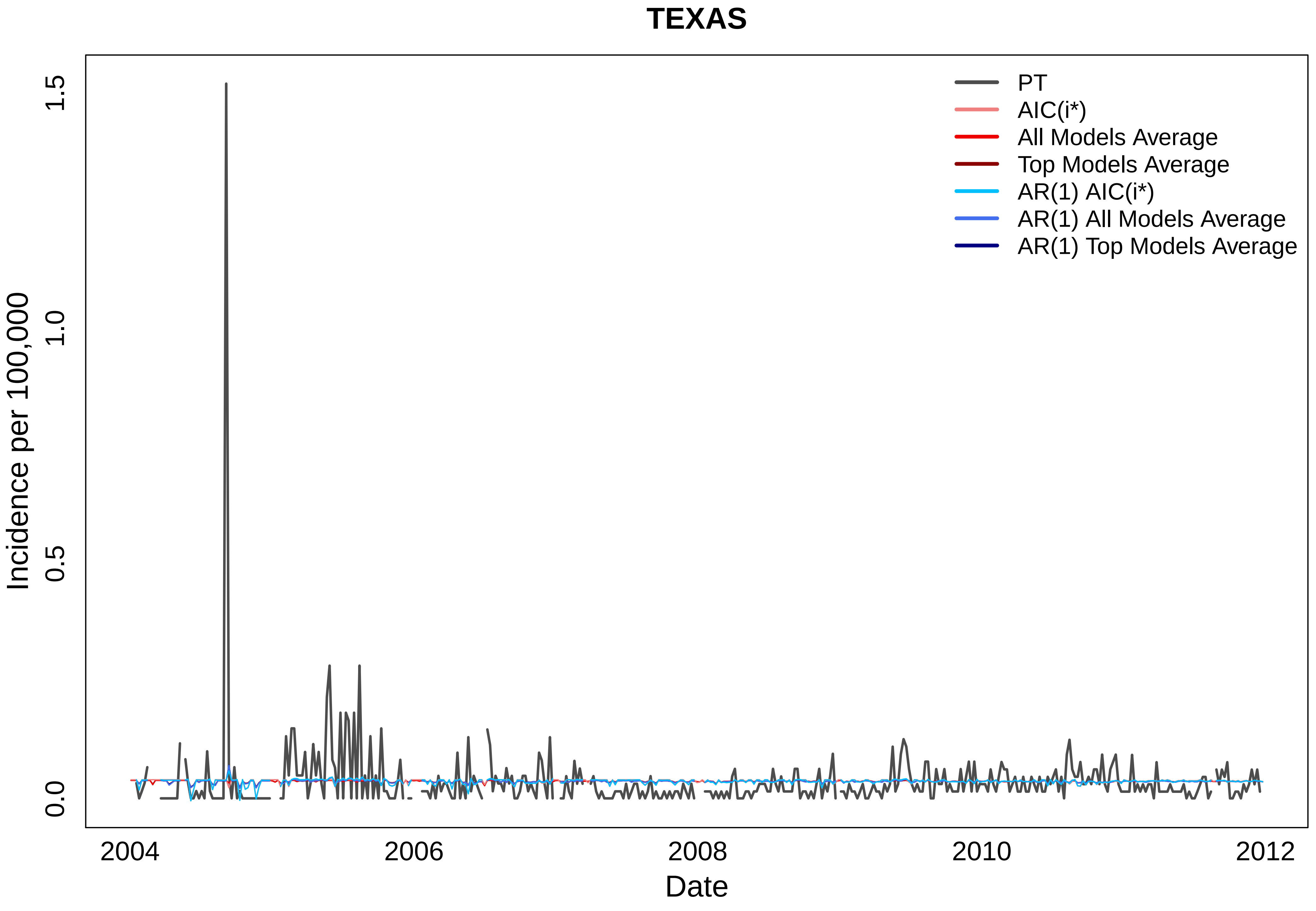
TEXAS
PT
AIC(i*)
All Models Average
Top Models Average
AR(1) AIC(i*)
AR(1) All Models Average
AR(1) Top Models Average
Incidence per 100,000
0.0
0.5
1.0
1.5
2004
2006
2008
2010
2012
Date

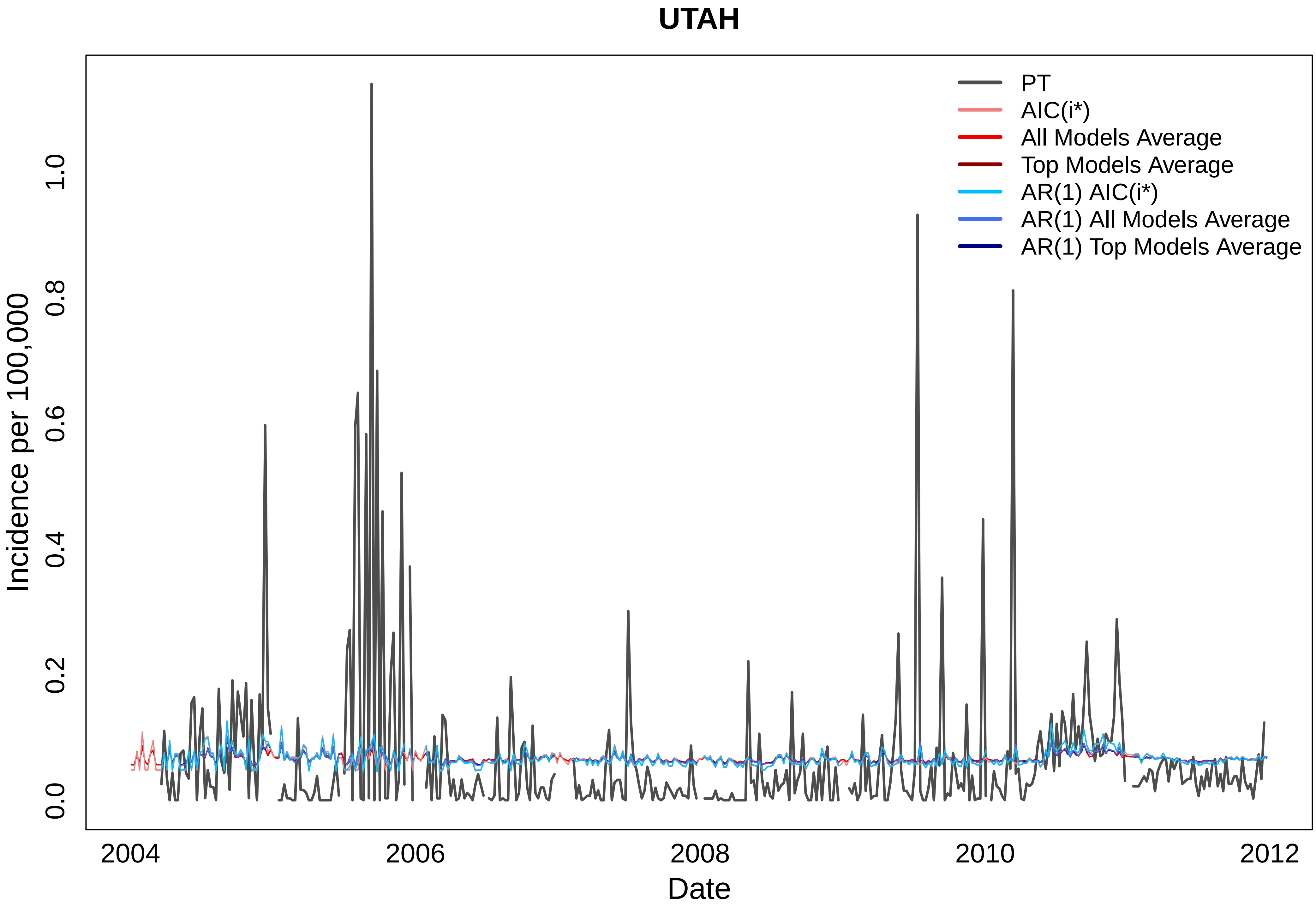

UTAH
PT
AIC(i*)
All Models Average
Top Models Average
AR(1) AIC(i*)
AR(1) All Models Average
AR(1) Top Models Average
Incidence per 100,000
0.0
0.2
0.4
0.6
0.8
1.0
2004
2006
2008
2010
2012
Date

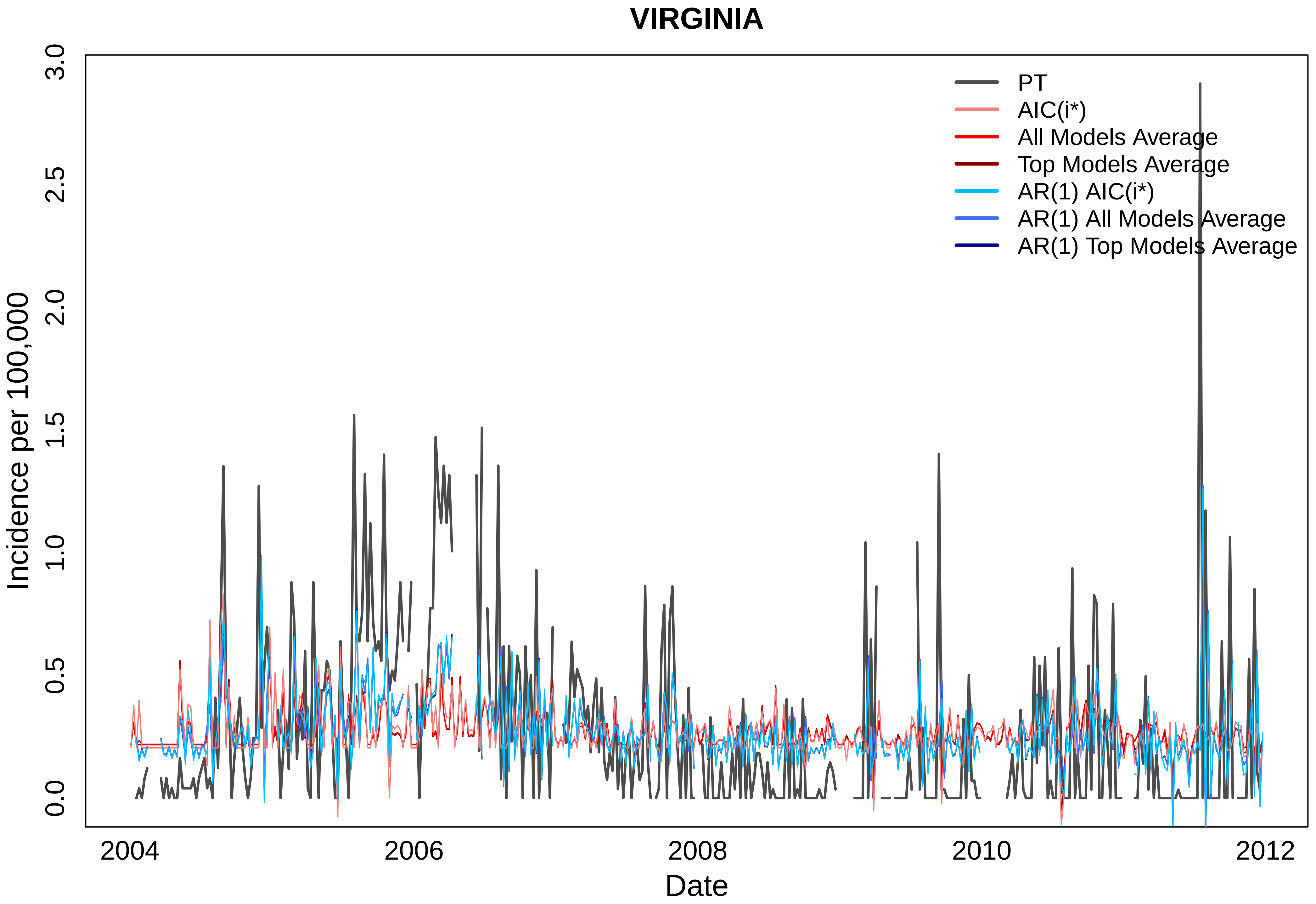

VIRGINIA
PT
AIC(i*)
All Models Average
Top Models Average
AR(1) AIC(i*)
AR(1) All Models Average
AR(1) Top Models Average
Incidence per 100,000
0.0
0.5
1.0
1.5
2.0
2.5
3.0
2004
2006
2008
2010
2012
Date

VERMONT

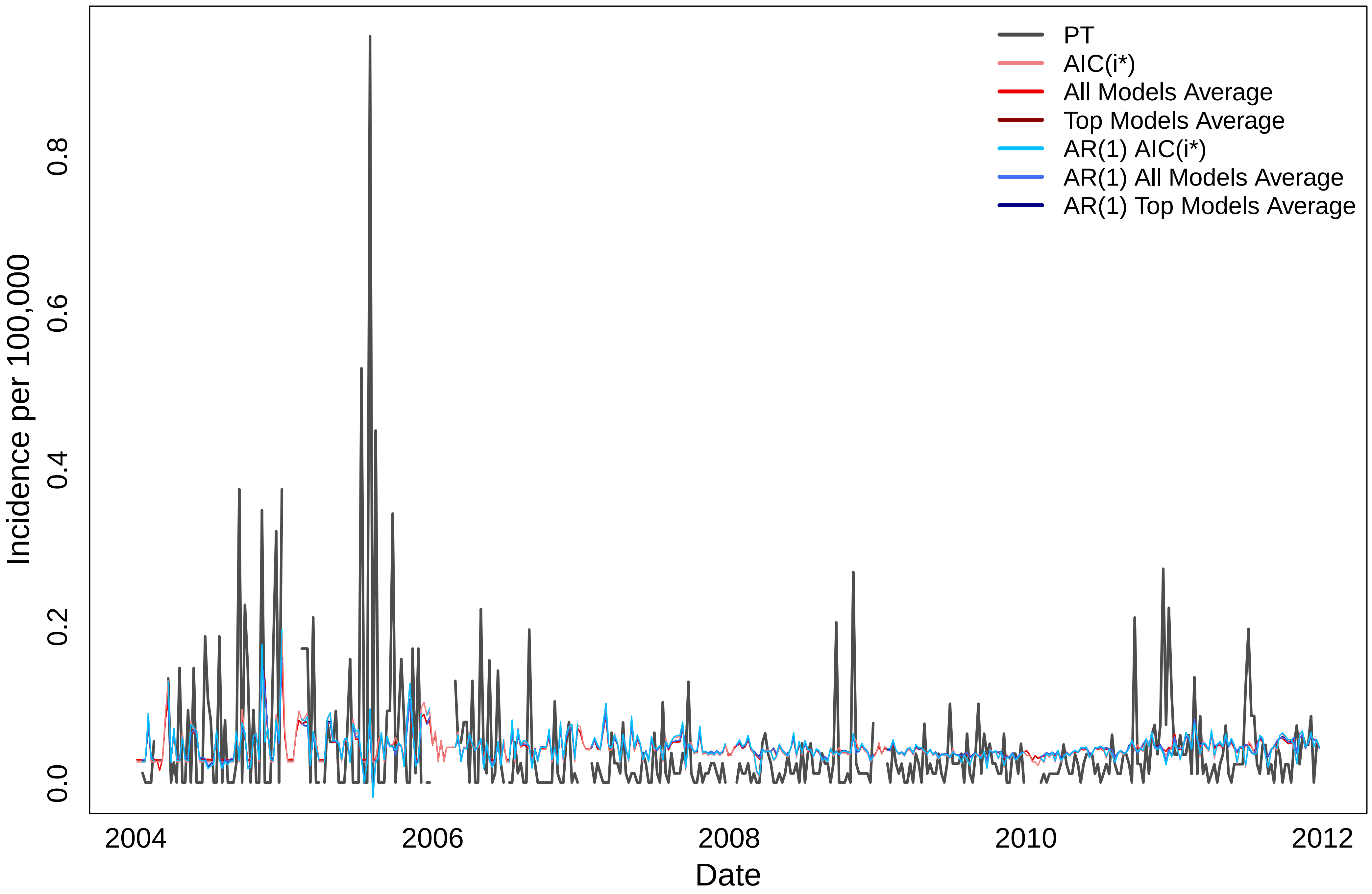

PT
AIC(i*)
All Models Average
Top Models Average
AR(1) AIC(i*)
AR(1) All Models Average
AR(1) Top Models Average
Incidence per 100,000
0.0
0.2
0.4
0.6
0.8
2004
2006
2008
2010
2012
Date

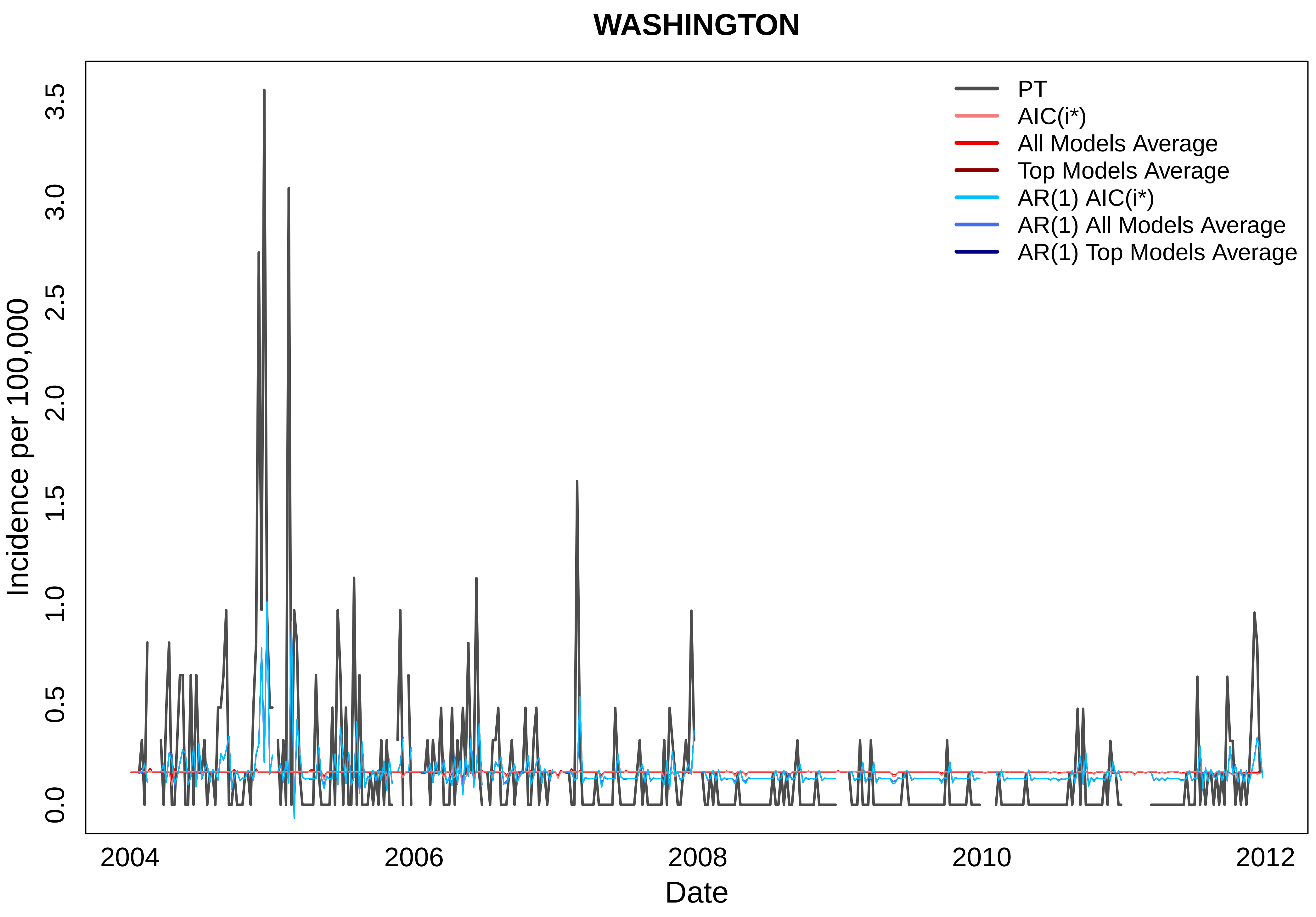
WASHINGTON
PT
AIC(i*)
All Models Average
Top Models Average
AR(1) AIC(i*)
AR(1) All Models Average
AR(1) Top Models Average
Incidence per 100,000
0.0
0.5
1.0
1.5
2.0
2.5
3.0
3.5
2004
2006
2008
2010
2012
Date

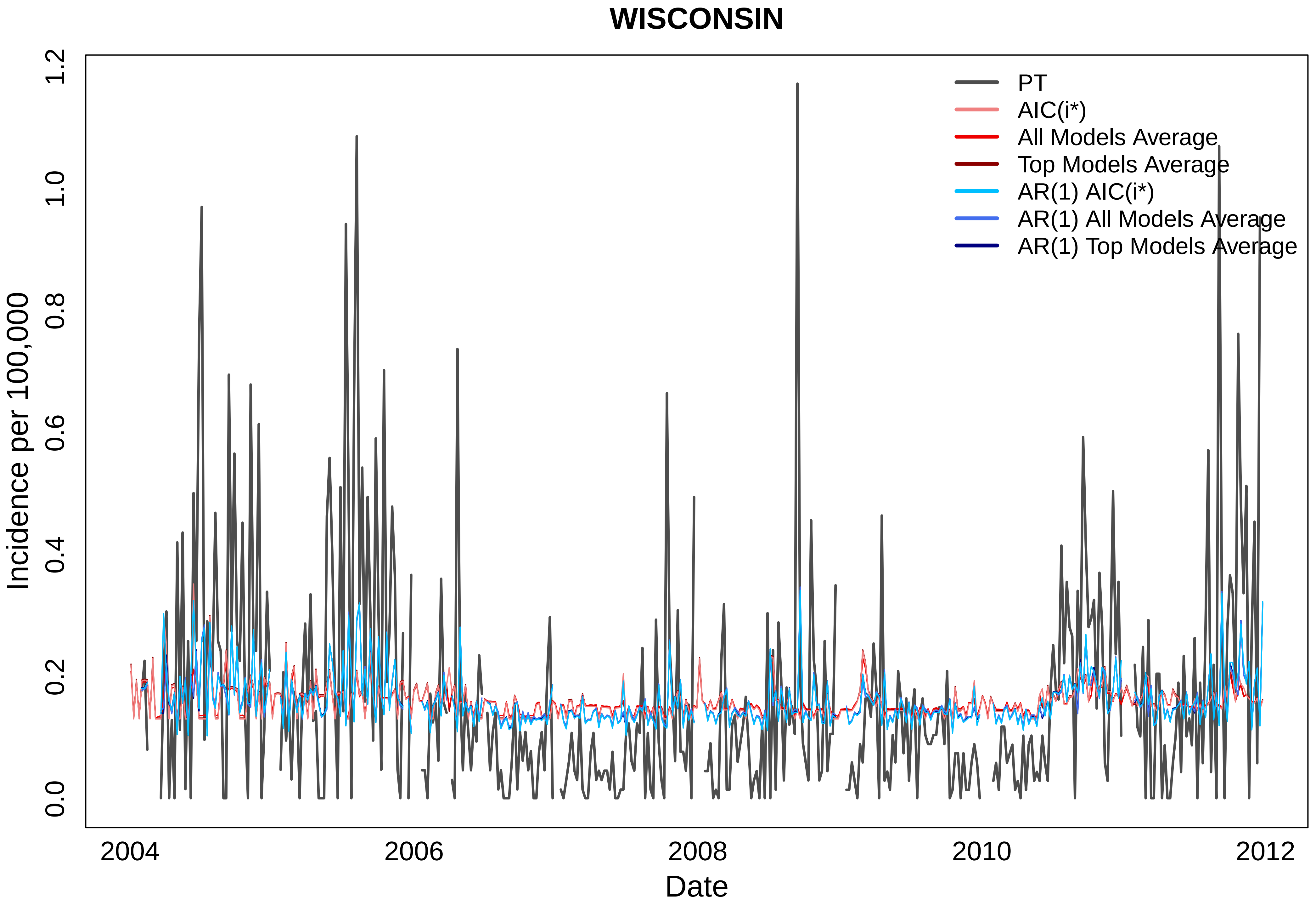
WISCONSIN
Incidence per 100,000
0.0
0.2
0.4
0.6
0.8
1.0
1.2
2004
2006
2008
2010
2012
Date
PT
AIC(i*)
All Models Average
Top Models Average
AR(1) AIC(i*)
AR(1) All Models Average
AR(1) Top Models Average

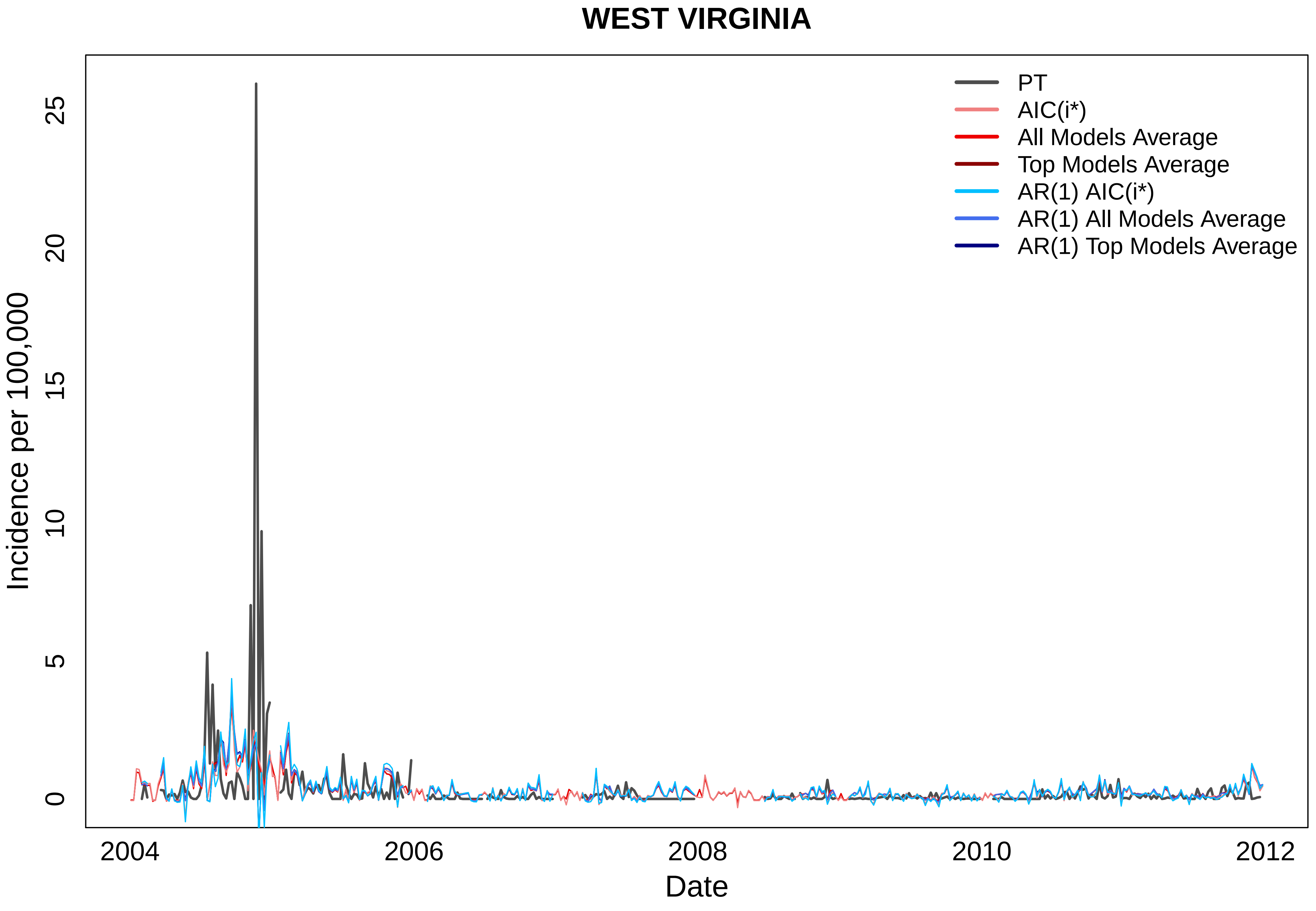
WEST VIRGINIA
PT
AIC(i*)
All Models Average
Top Models Average
AR(1) AIC(i*)
AR(1) All Models Average
AR(1) Top Models Average
Incidence per 100,000
0
5
10
15
20
25
2004
2006
2008
2010
2012
Date

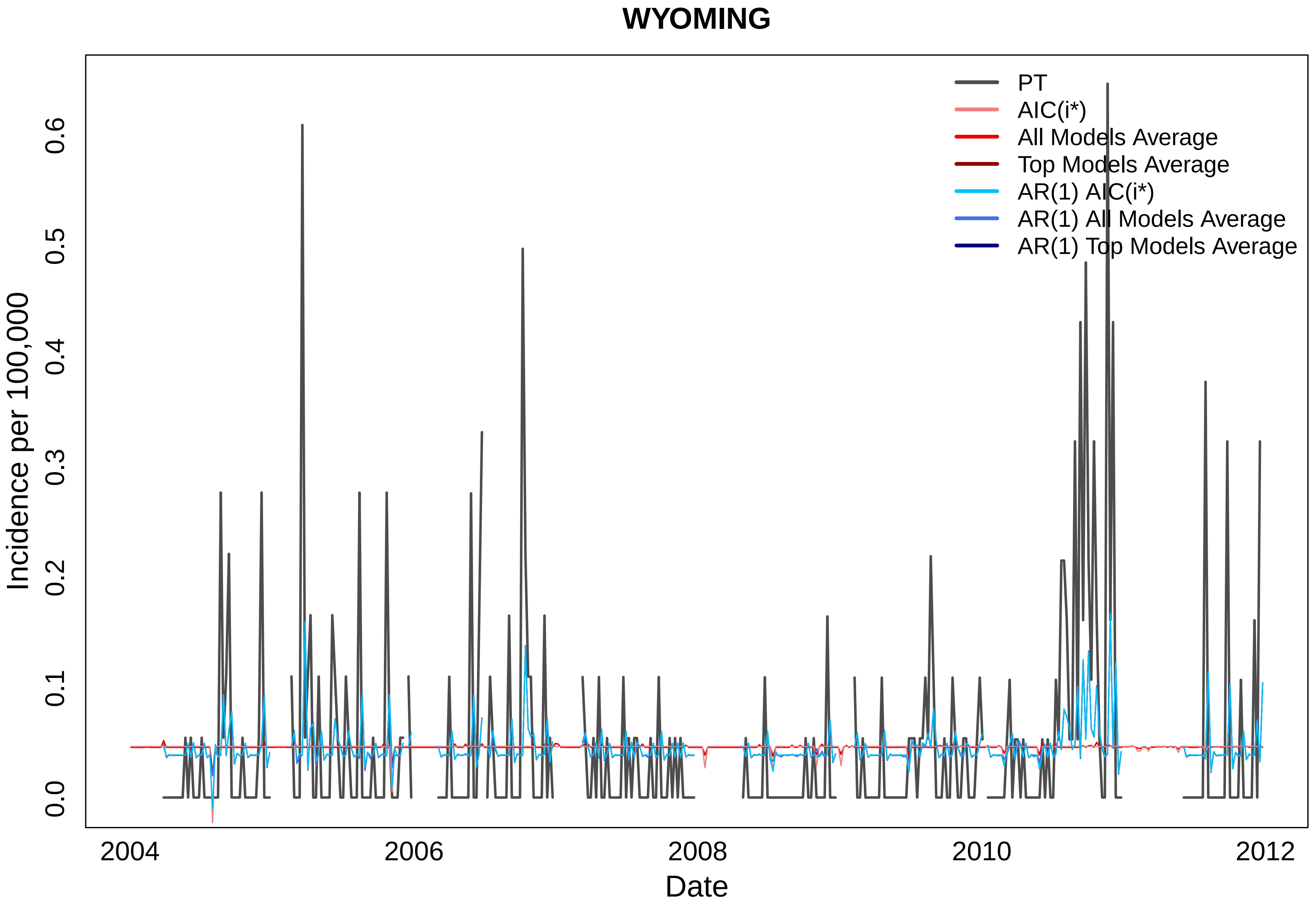
WYOMING
PT
AIC(i*)
All Models Average
Top Models Average
AR(1) AIC(i*)
AR(1) All Models Average
AR(1) Top Models Average
Incidence per 100,000
0.0
0.1
0.2
0.3
0.4
0.5
0.6
2004
2006
2008
2010
2012
Date

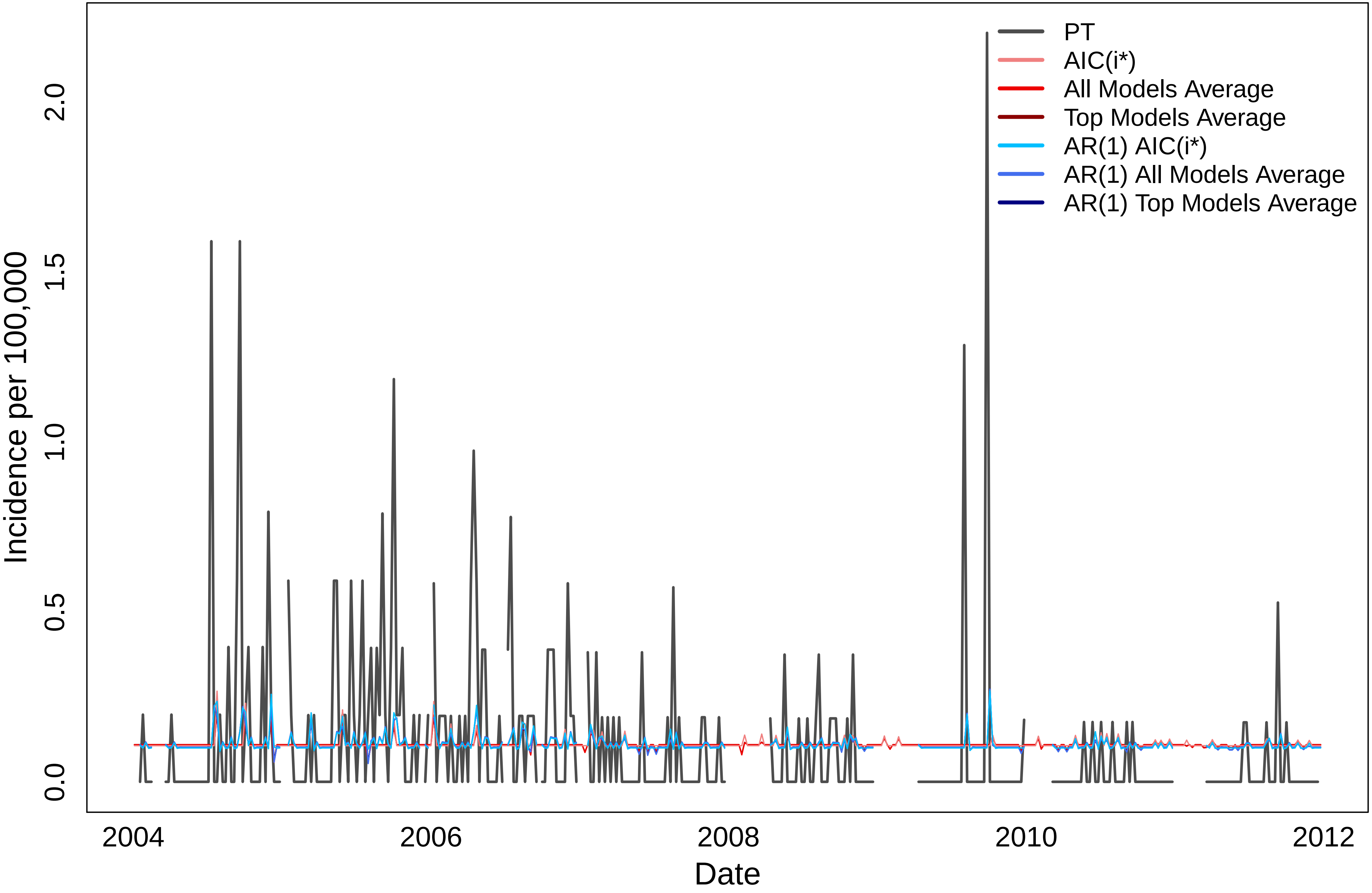

PT
AIC(i*)
All Models Average
Top Models Average
AR(1) AIC(i*)
AR(1) All Models Average
AR(1) Top Models Average
Incidence per 100,000
0.0
0.5
1.0
1.5
2.0
2004
2006
2008
2010
2012
Date

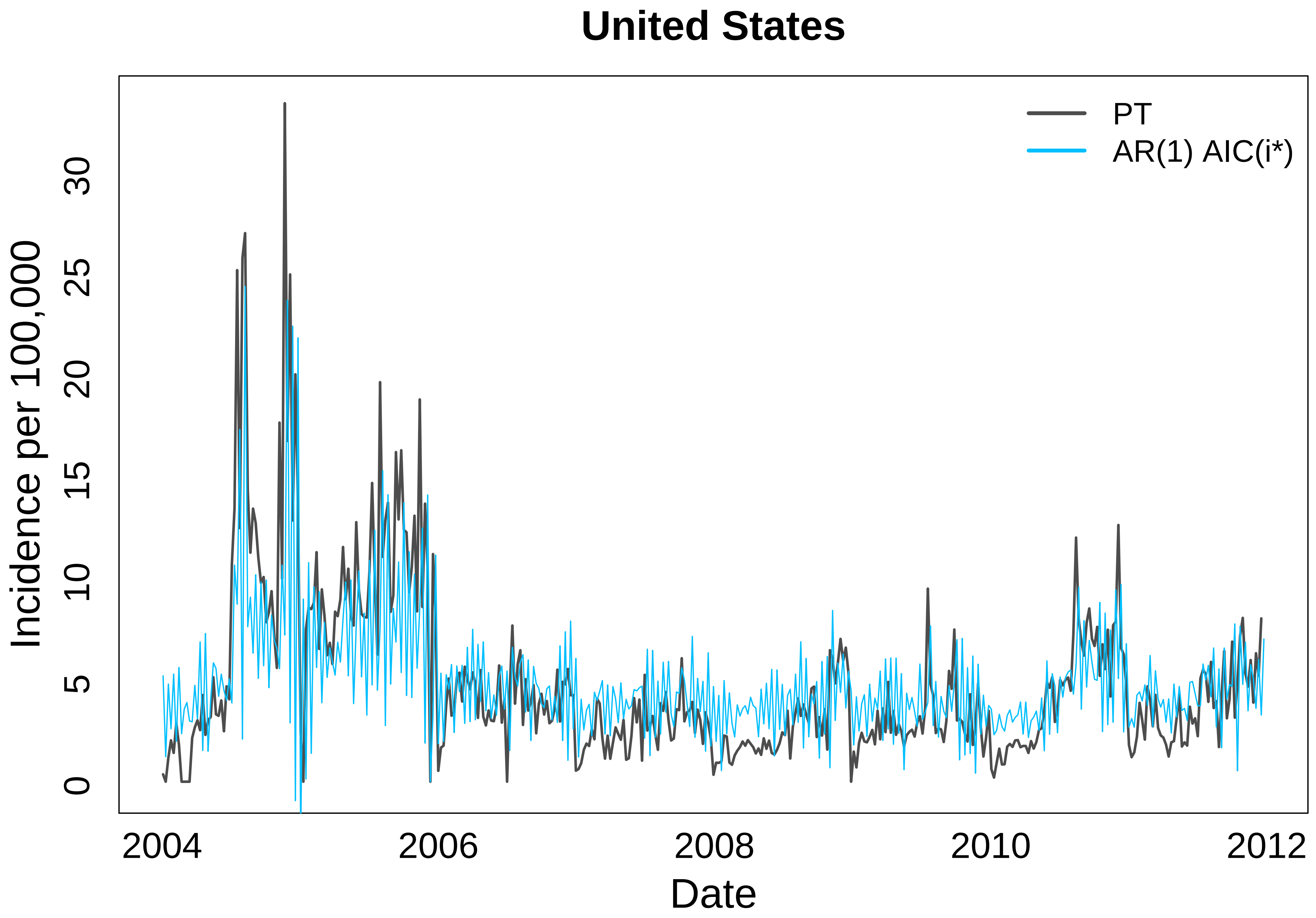

United States
PT
AR(1) AIC(i*)
Incidence per 100,000
Date
0
5
10
15
20
25
30
2004
2006
2008
2010
2012

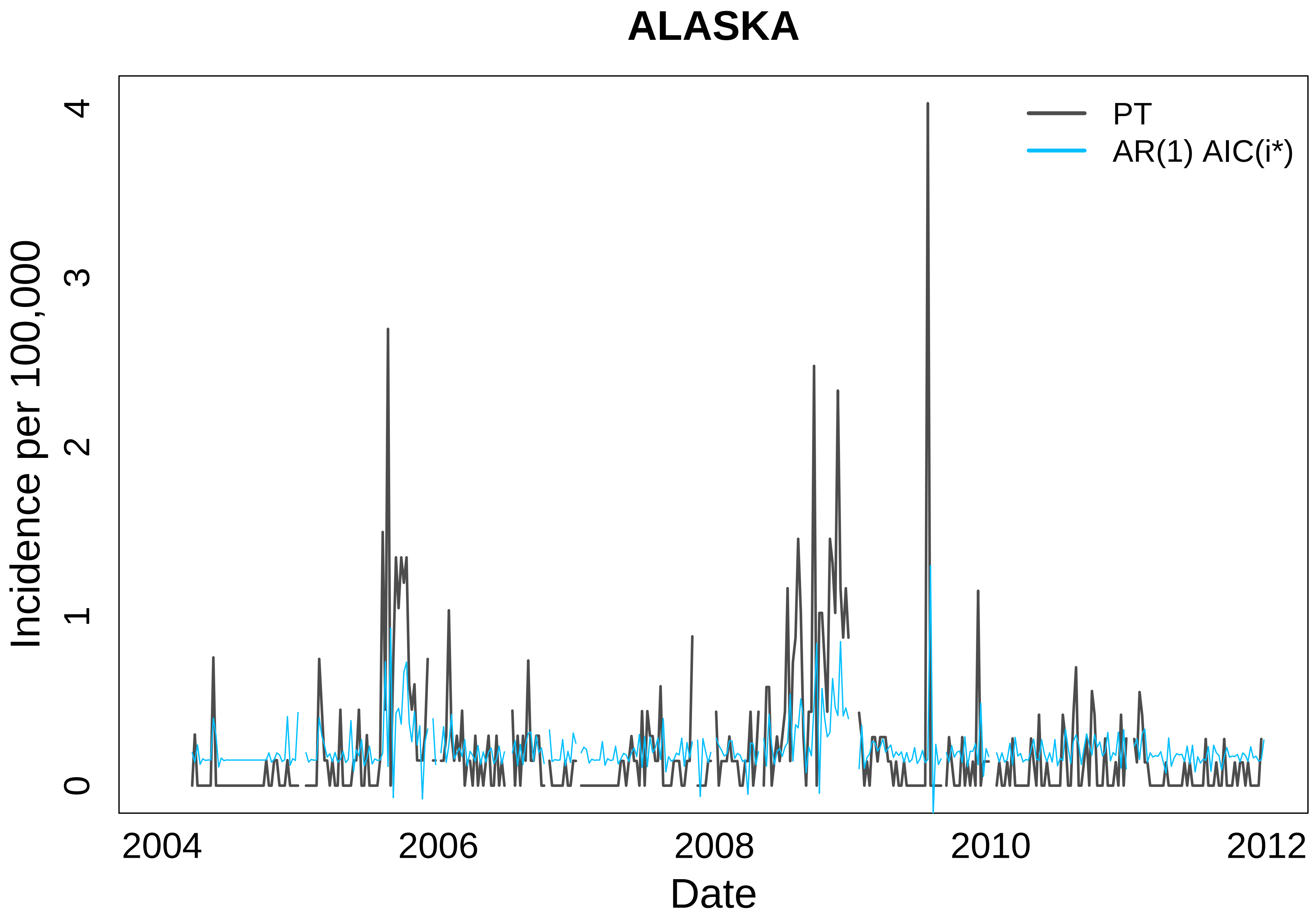

ALASKA
PT
AR(1) AIC(i*)
Incidence per 100,000
0
1
2
3
4
2004
2006
2008
2010
2012
Date

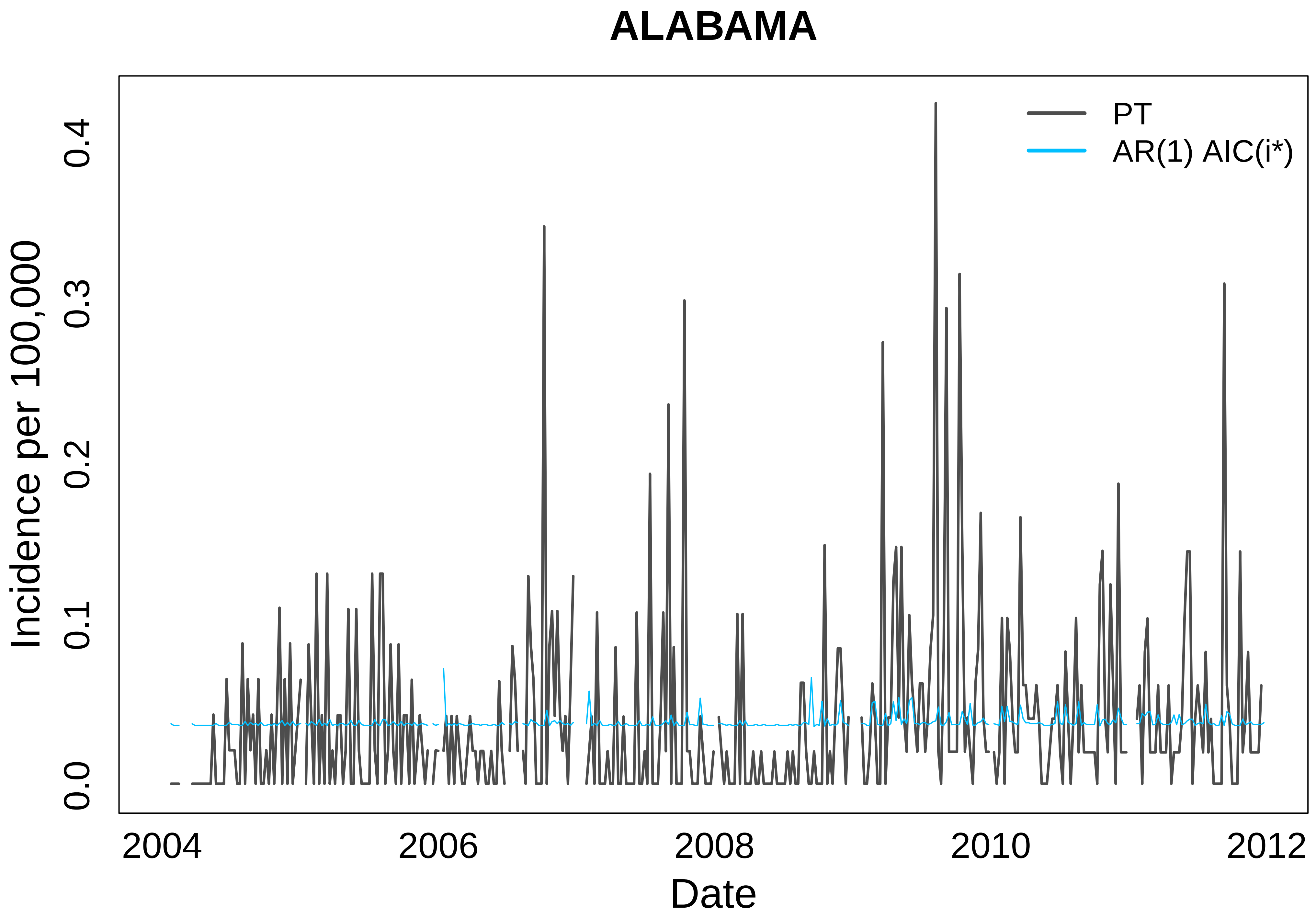

ALABAMA
PT
AR(1) AIC(i*)
Incidence per 100,000
0.0
0.1
0.2
0.3
0.4
2004
2006
2008
2010
2012
Date

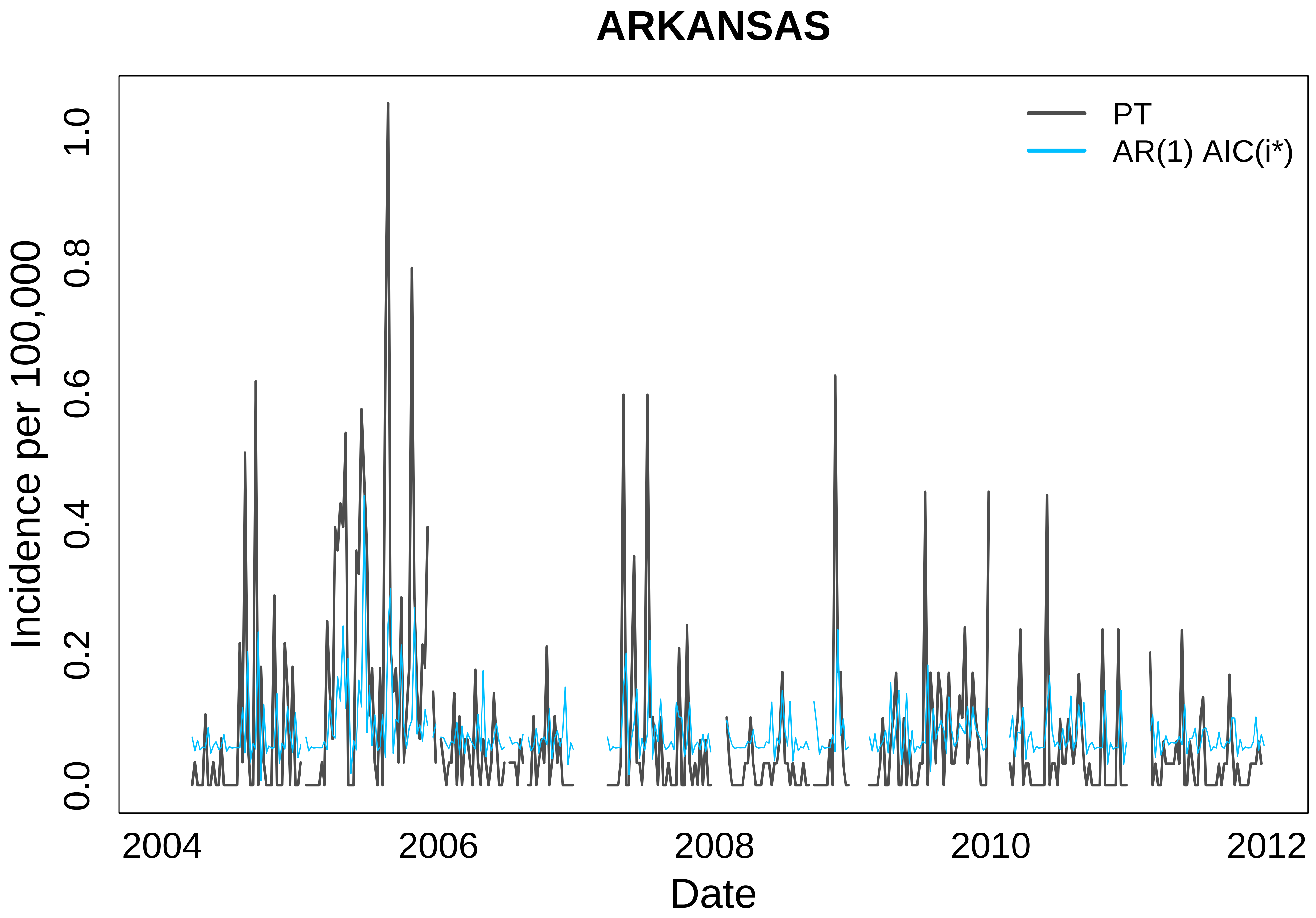
ARKANSAS
PT
AR(1) AIC(i*)
Incidence per 100,000
0.0
0.2
0.4
0.6
0.8
1.0
2004
2006
2008
2010
2012
Date

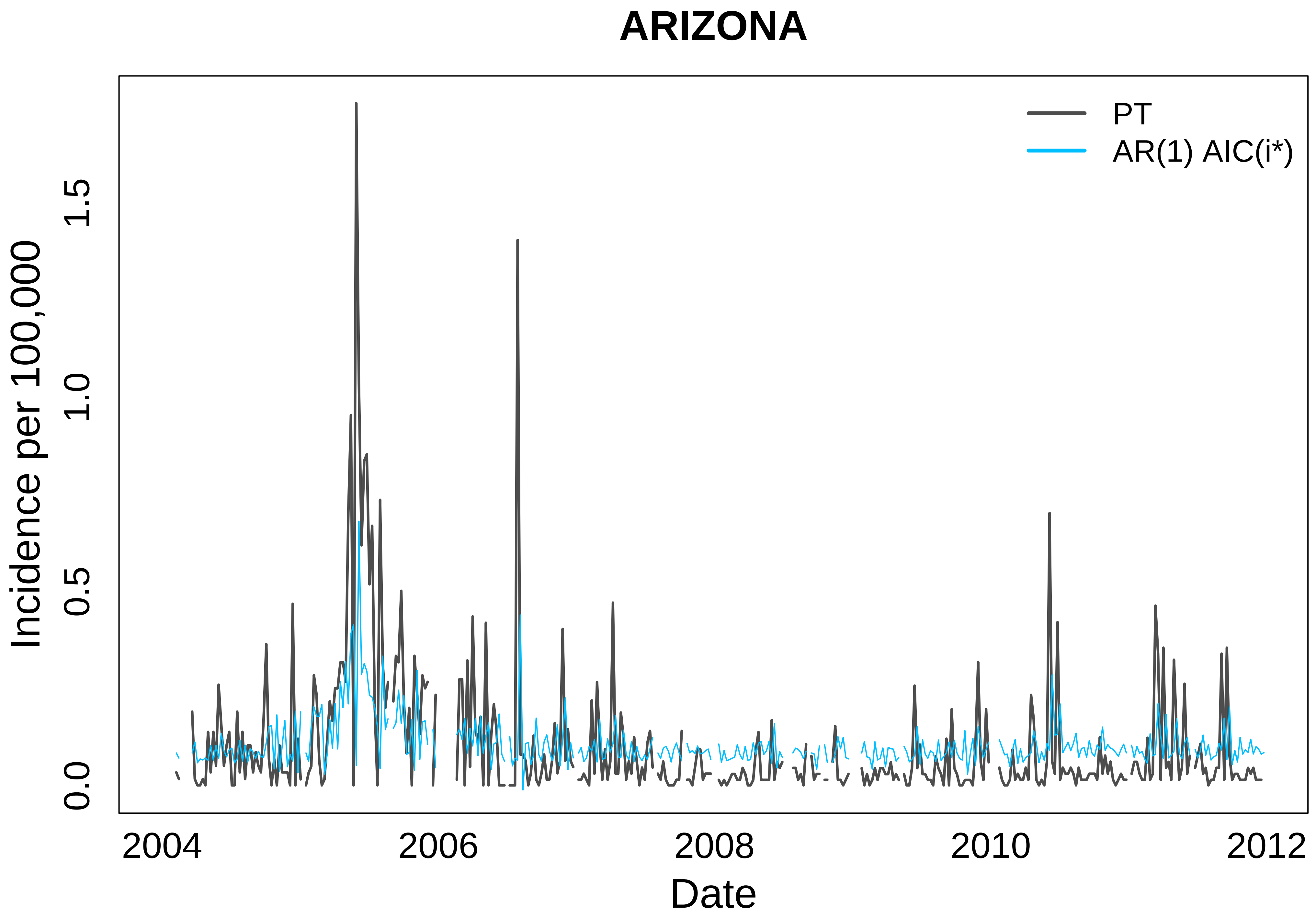
ARIZONA
PT
AR(1) AIC(i*)
Incidence per 100,000
0.0
0.5
1.0
1.5
2004
2006
2008
2010
2012
Date

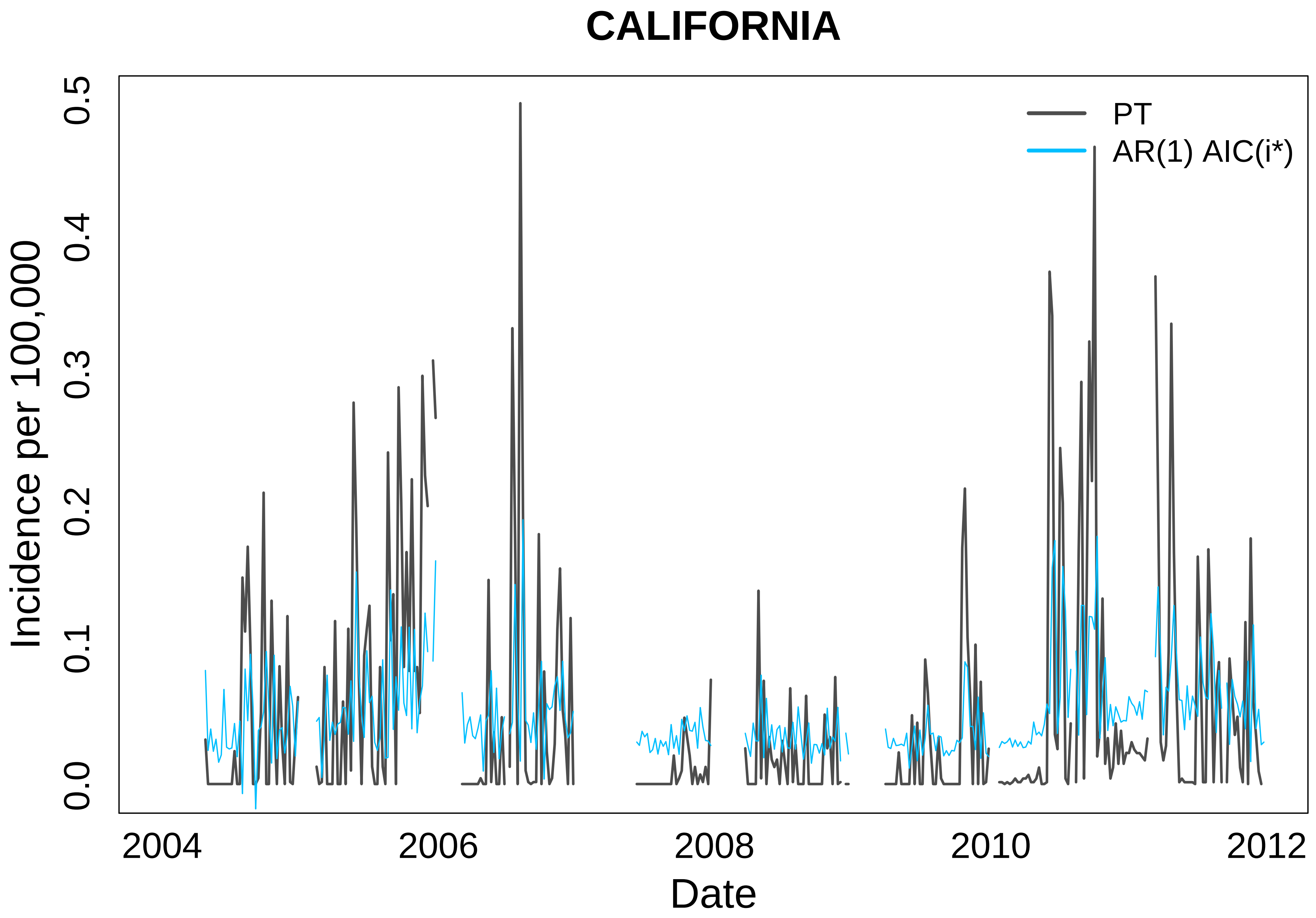

CALIFORNIA
Incidence per 100,000
0.0
0.1
0.2
0.3
0.4
0.5
PT
AR(1) AIC(i*)
2004
2006
2008
2010
2012
Date

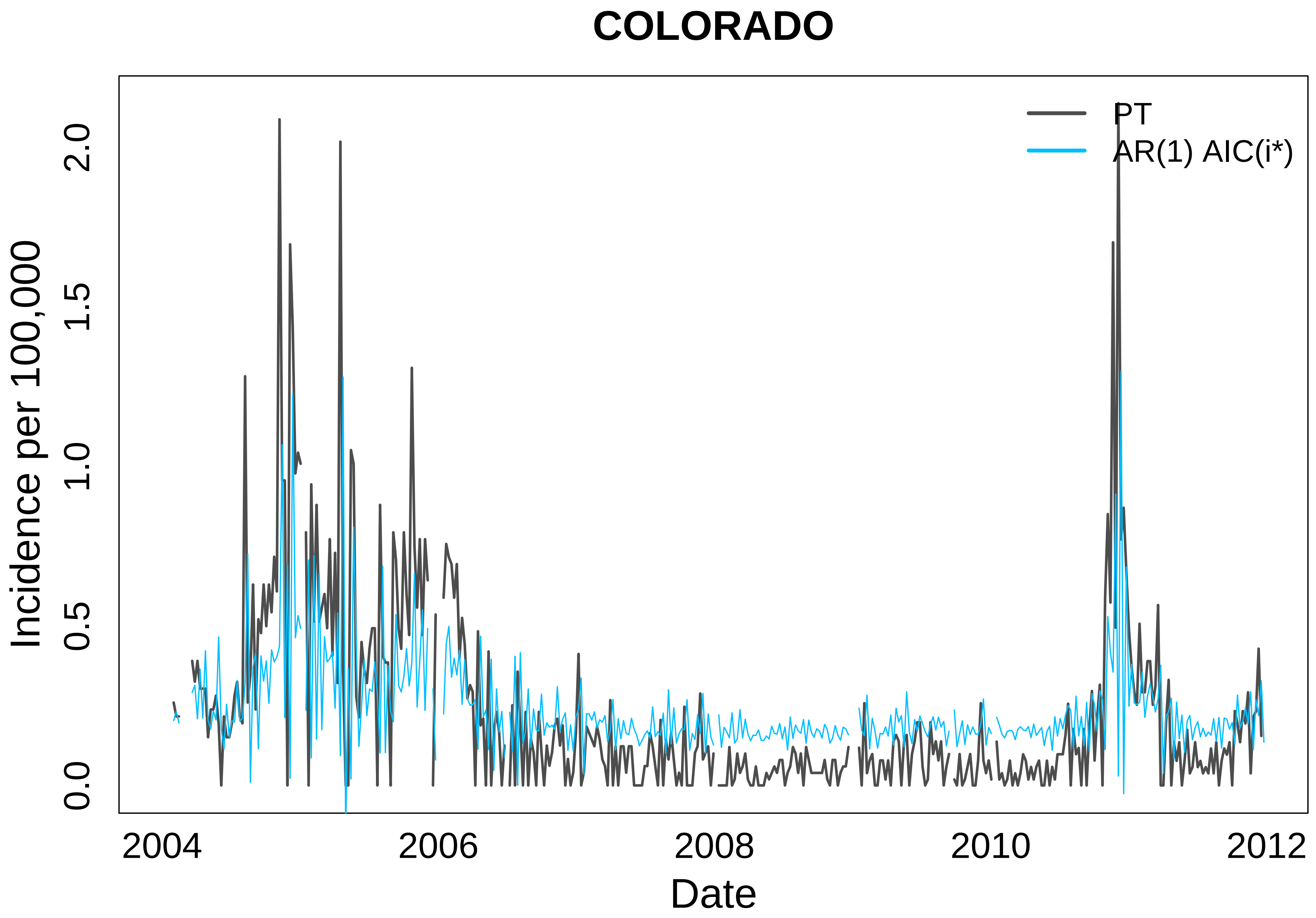

COLORADO
PT
AR(1) AIC(i*)
Incidence per 100,000
0.0
0.5
1.0
1.5
2.0
2004
2006
2008
2010
2012
Date

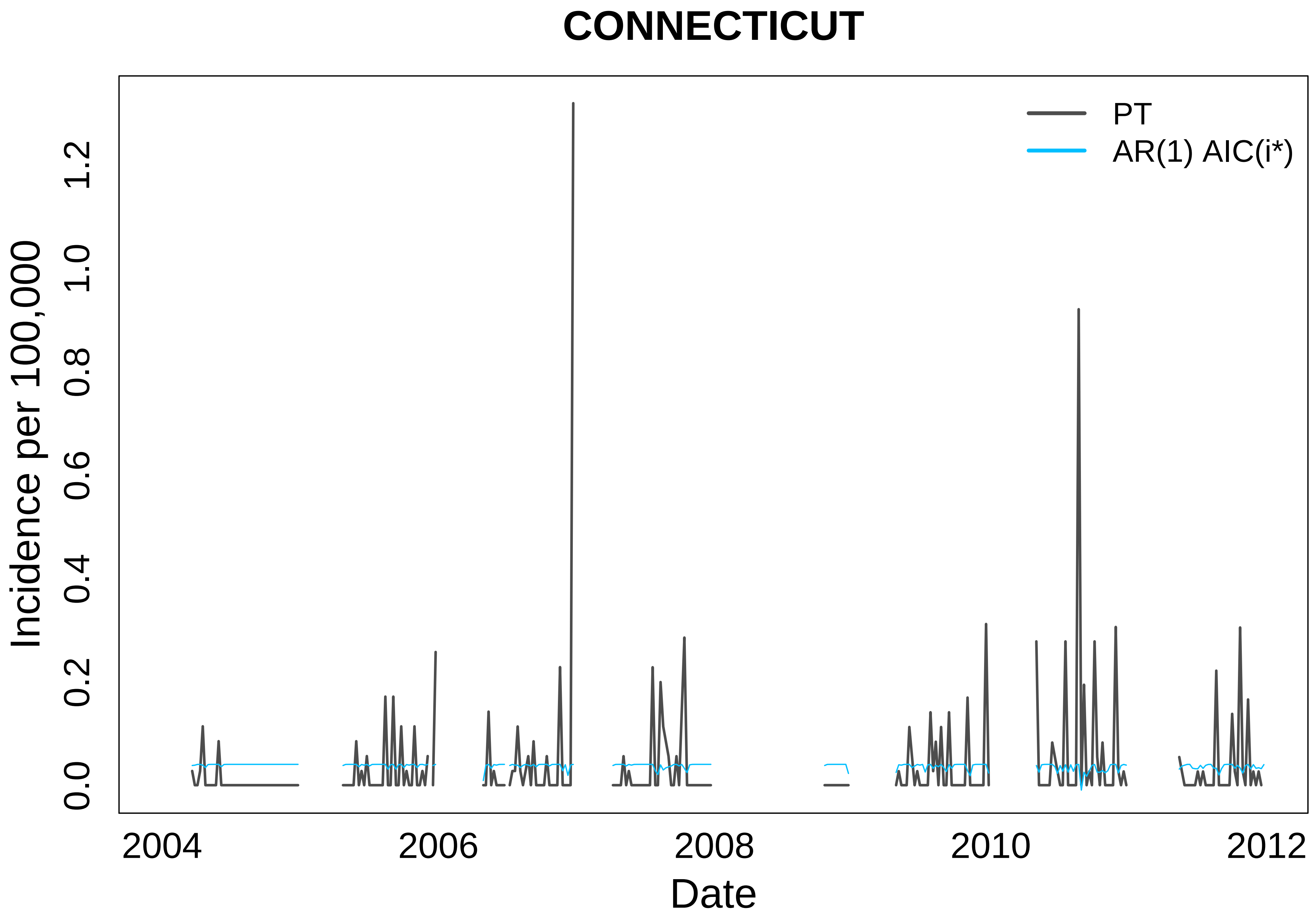

CONNECTICUT
PT
AR(1) AIC(i*)
Incidence per 100,000
0.0
0.2
0.4
0.6
0.8
1.0
1.2
2004
2006
2008
2010
2012
Date

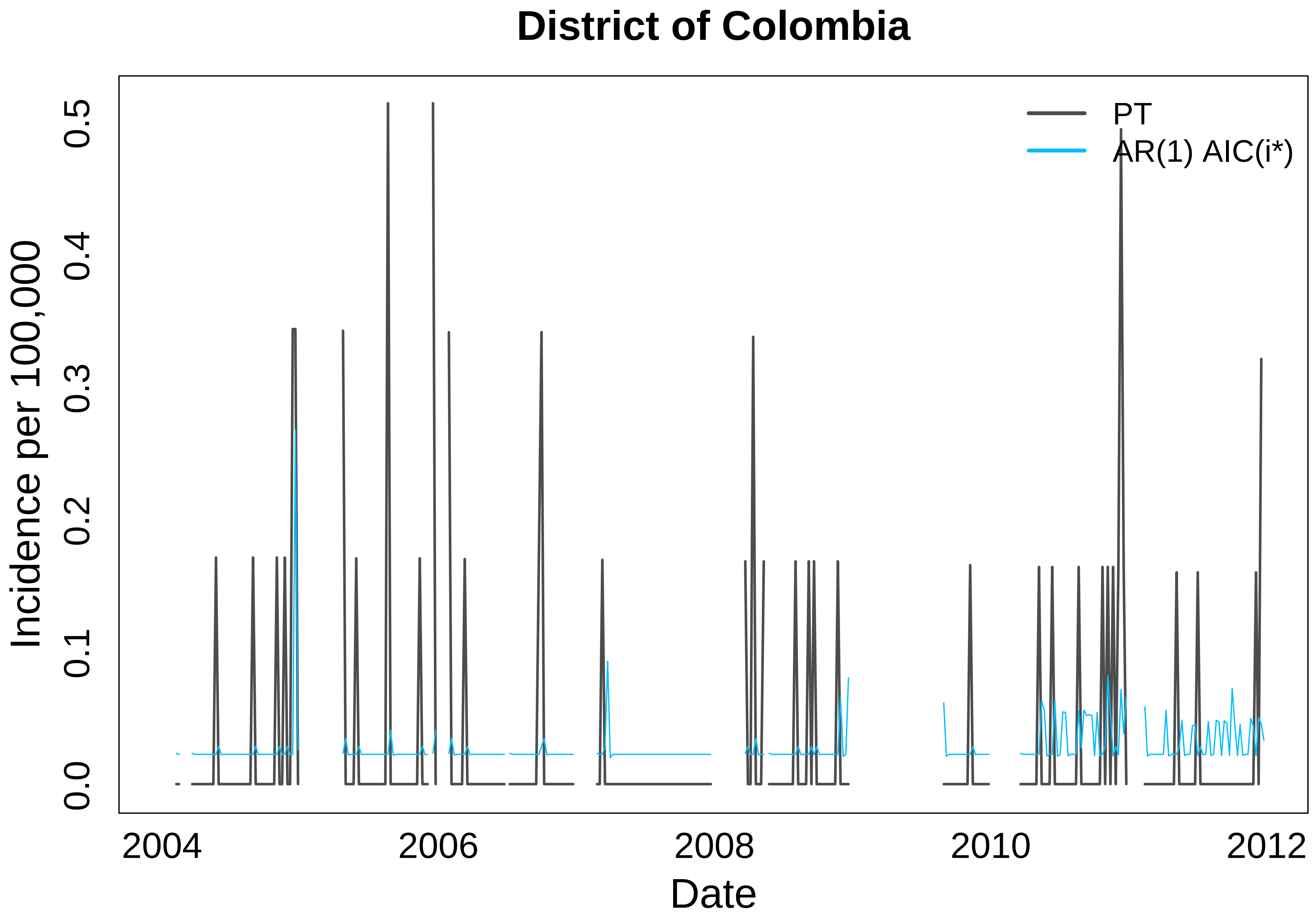

District of Colombia
PT
AR(1) AIC(i*)
Incidence per 100,000
0.0
0.1
0.2
0.3
0.4
0.5
2004
2006
2008
2010
2012
Date

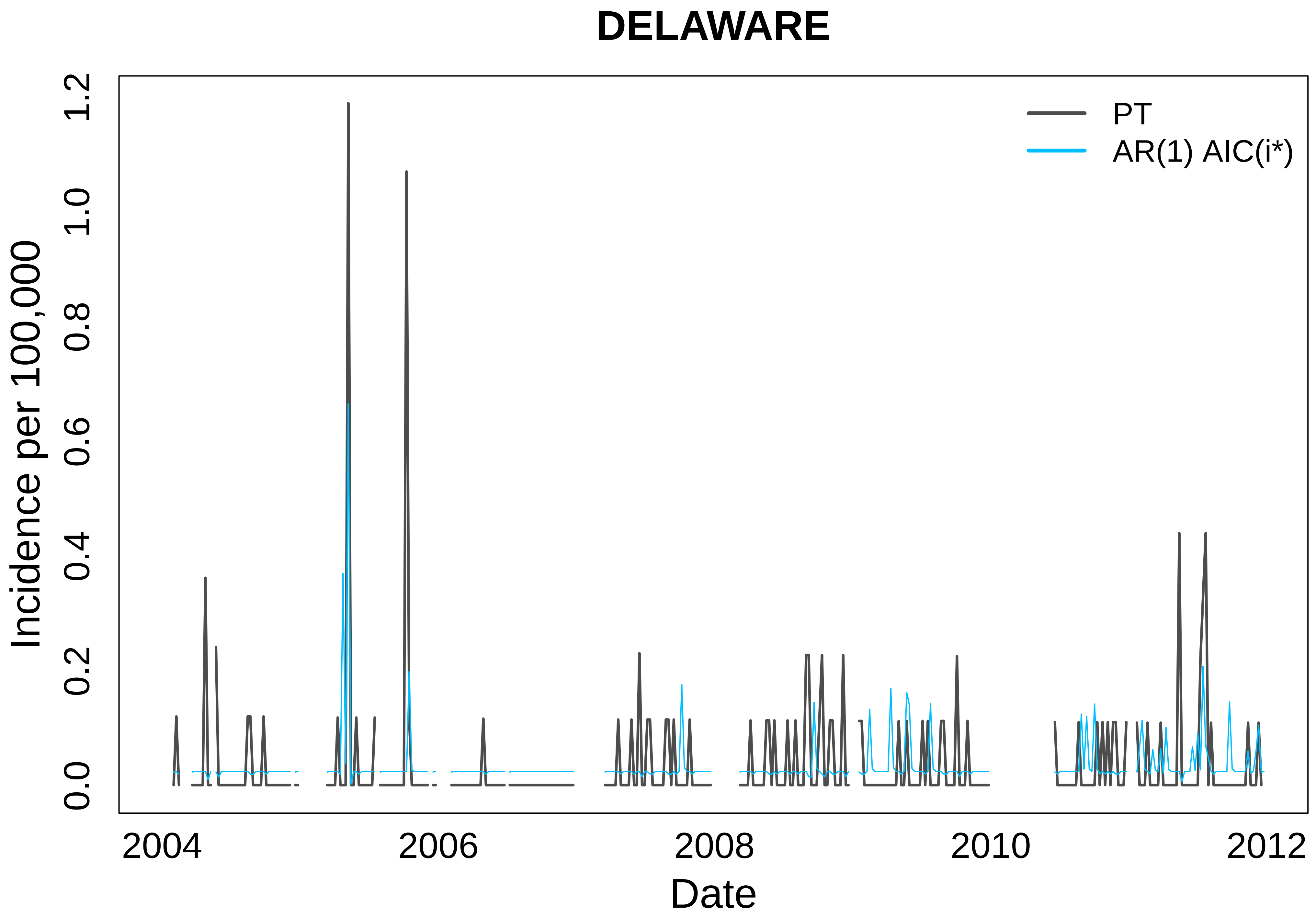

DELAWARE
PT
AR(1) AIC(i*)
Incidence per 100,000
0.0
0.2
0.4
0.6
0.8
1.0
1.2
2004
2006
2008
2010
2012
Date

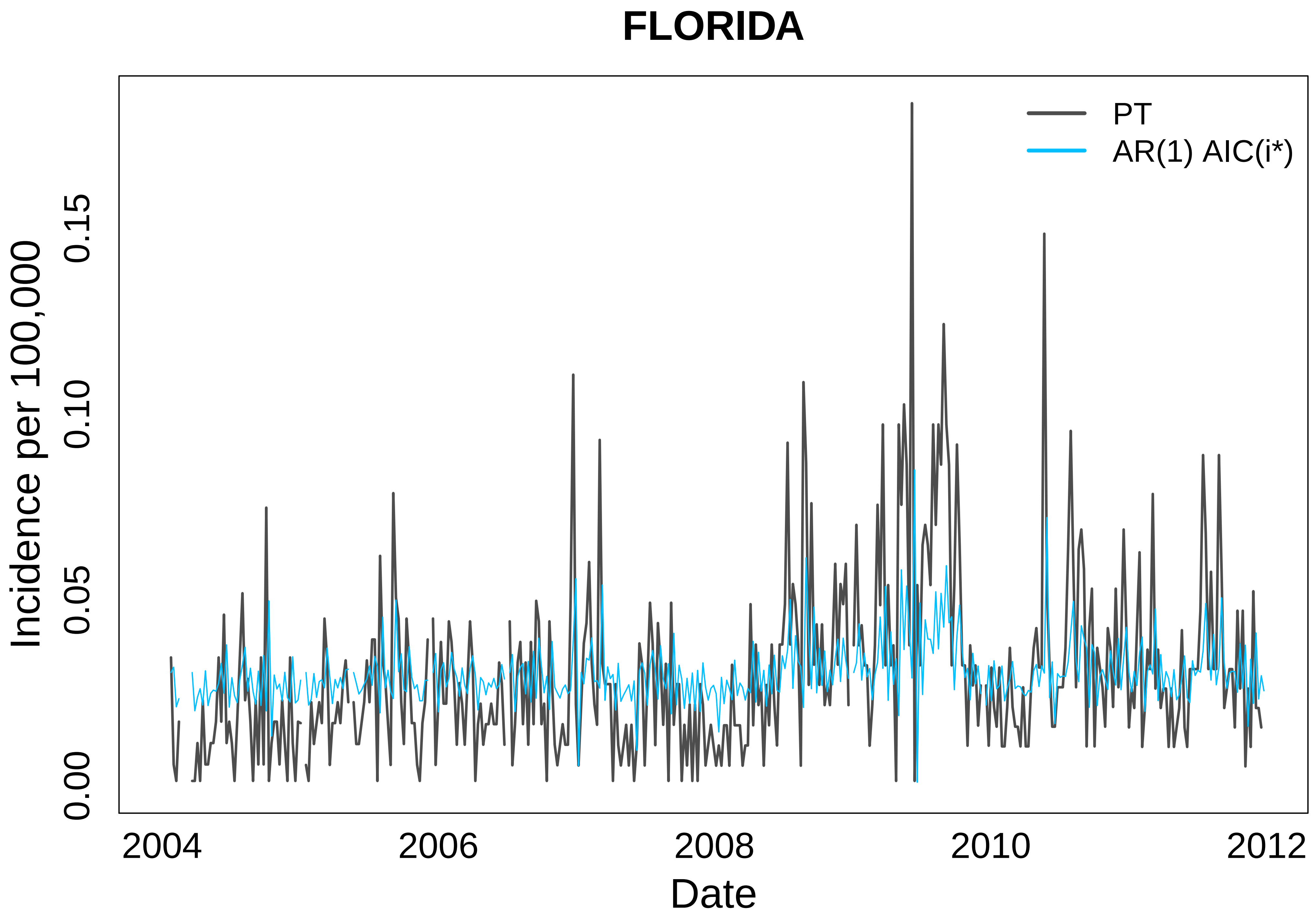
FLORIDA
PT
AR(1) AIC(i*)
Incidence per 100,000
0.00
0.05
0.10
0.15
2004
2006
2008
2010
2012
Date

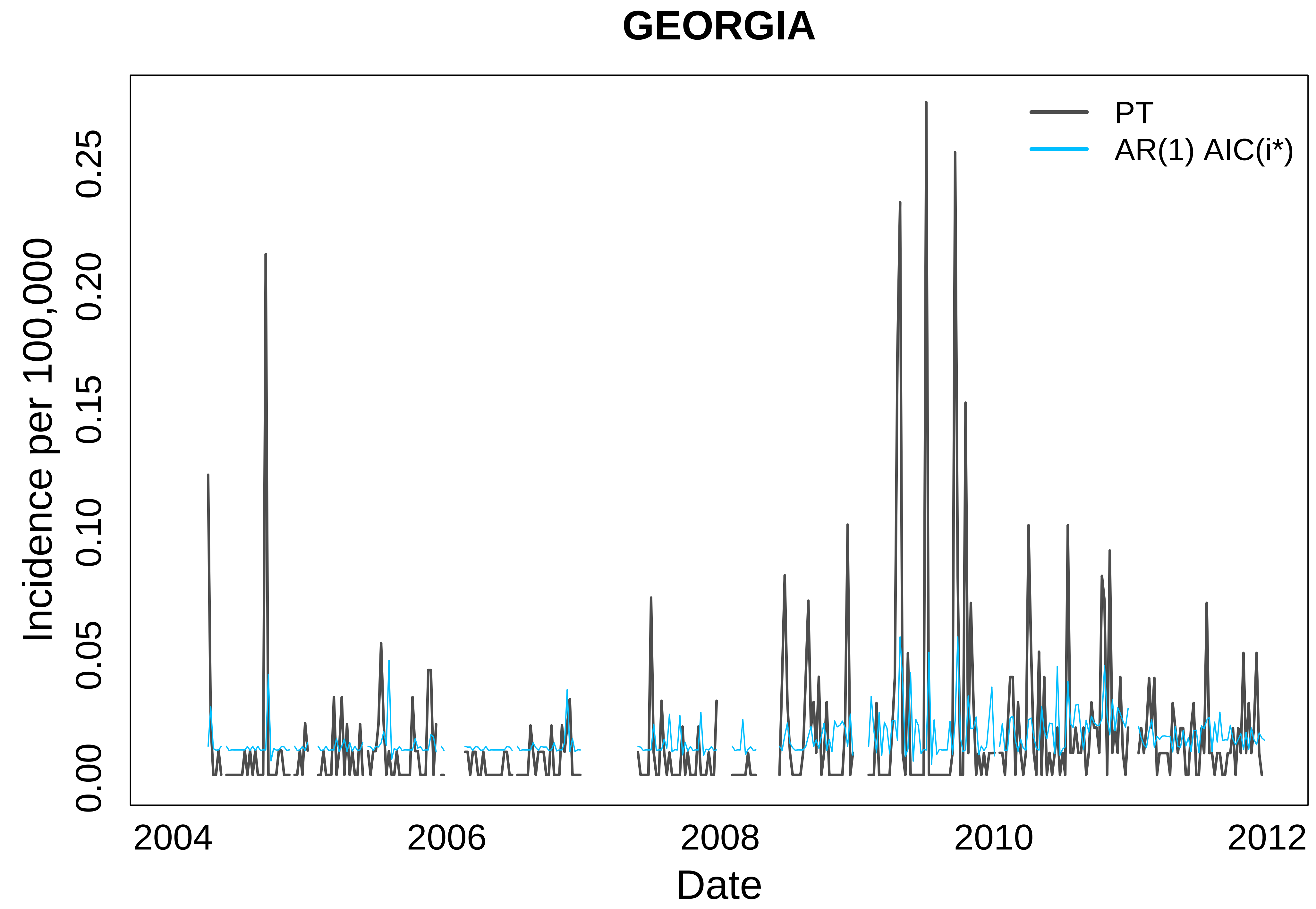

GEORGIA
PT
AR(1) AIC(i*)
Incidence per 100,000
0.00
0.05
0.10
0.15
0.20
0.25
2004
2006
2008
2010
2012
Date

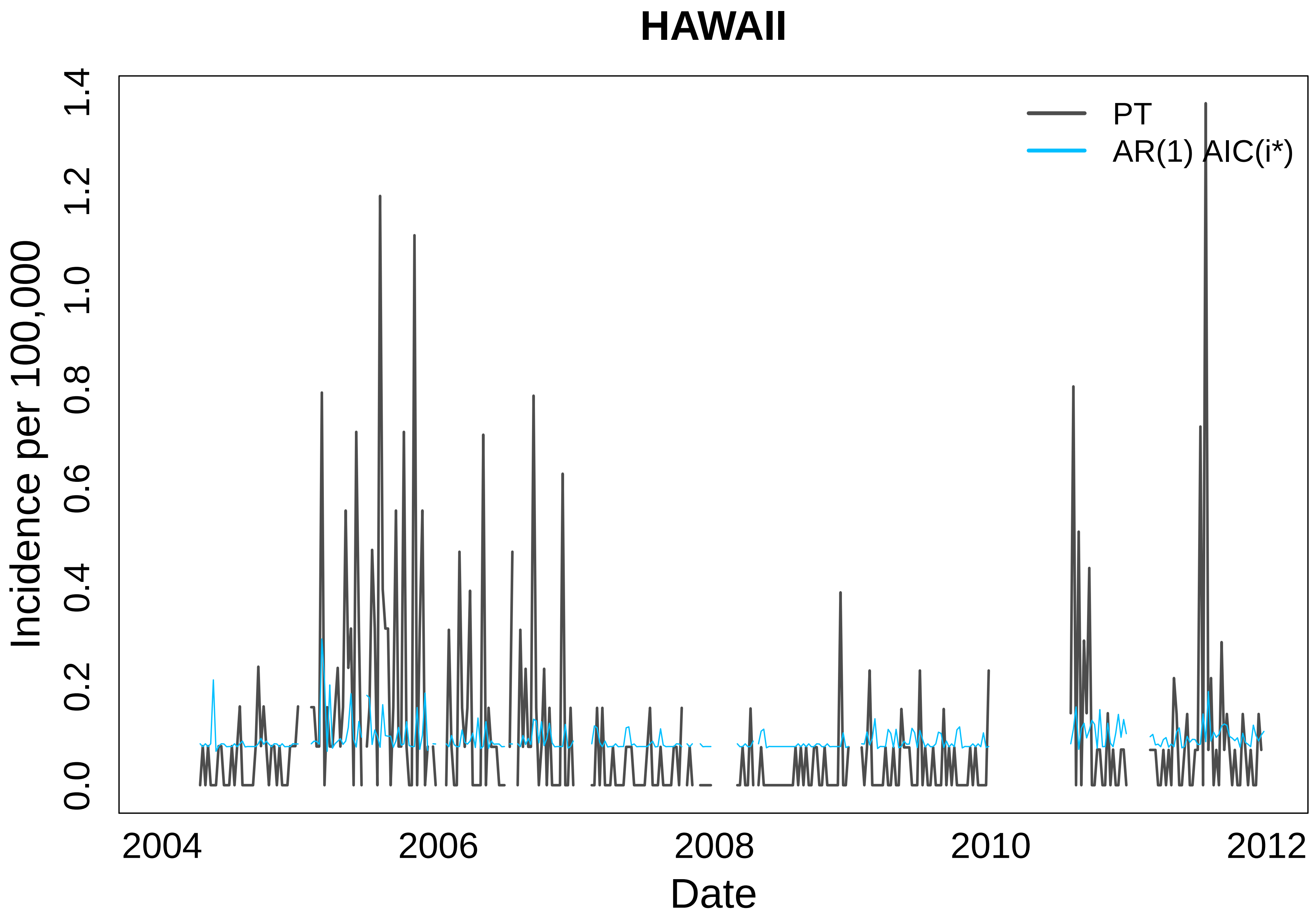

HAWAII
PT
AR(1) AIC(i*)
Incidence per 100,000
0.0
0.2
0.4
0.6
0.8
1.0
1.2
1.4
2004
2006
2008
2010
2012
Date

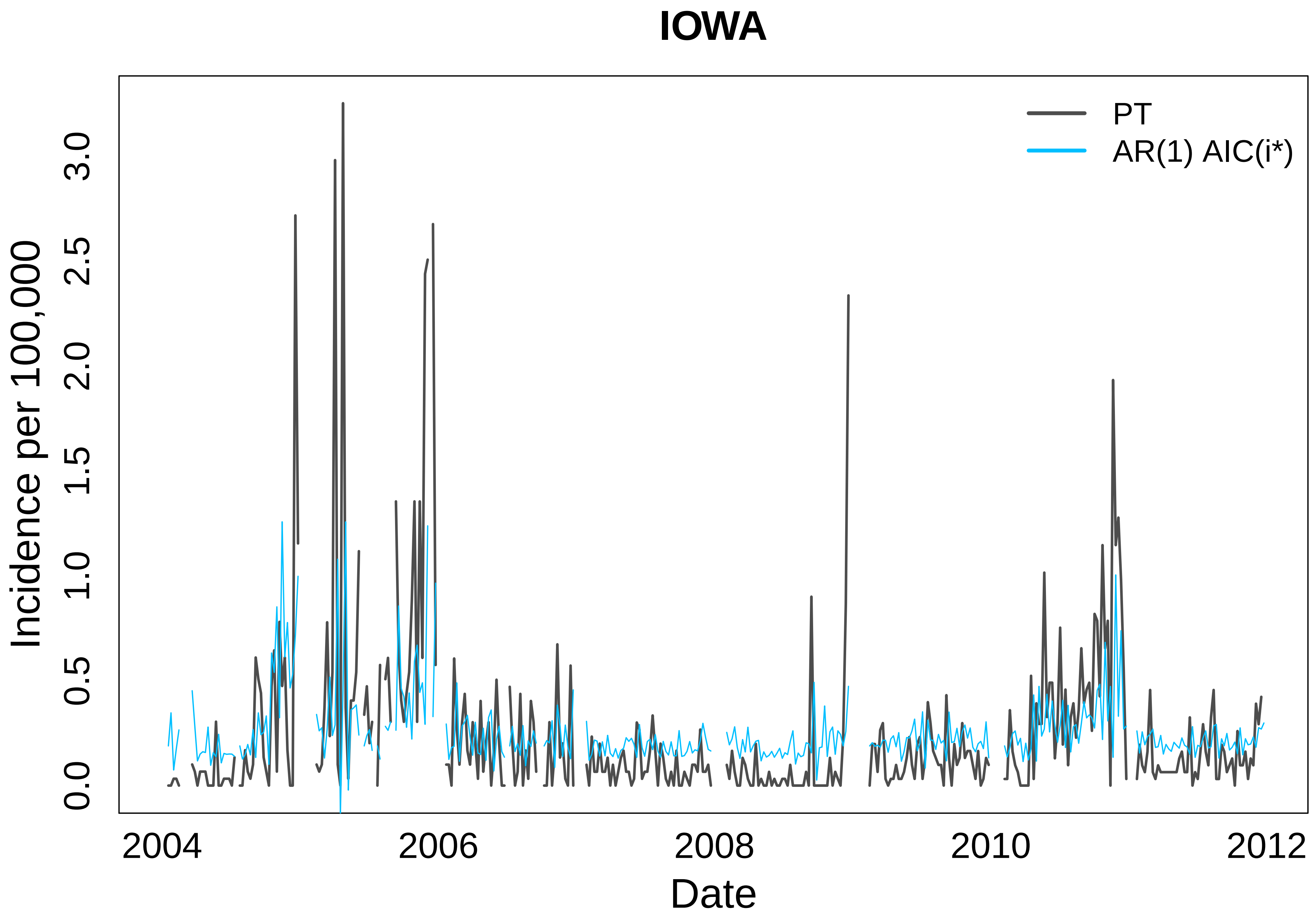
IOWA
PT
AR(1) AIC(i*)
Incidence per 100,000
0.0
0.5
1.0
1.5
2.0
2.5
3.0
2004
2006
2008
2010
2012
Date

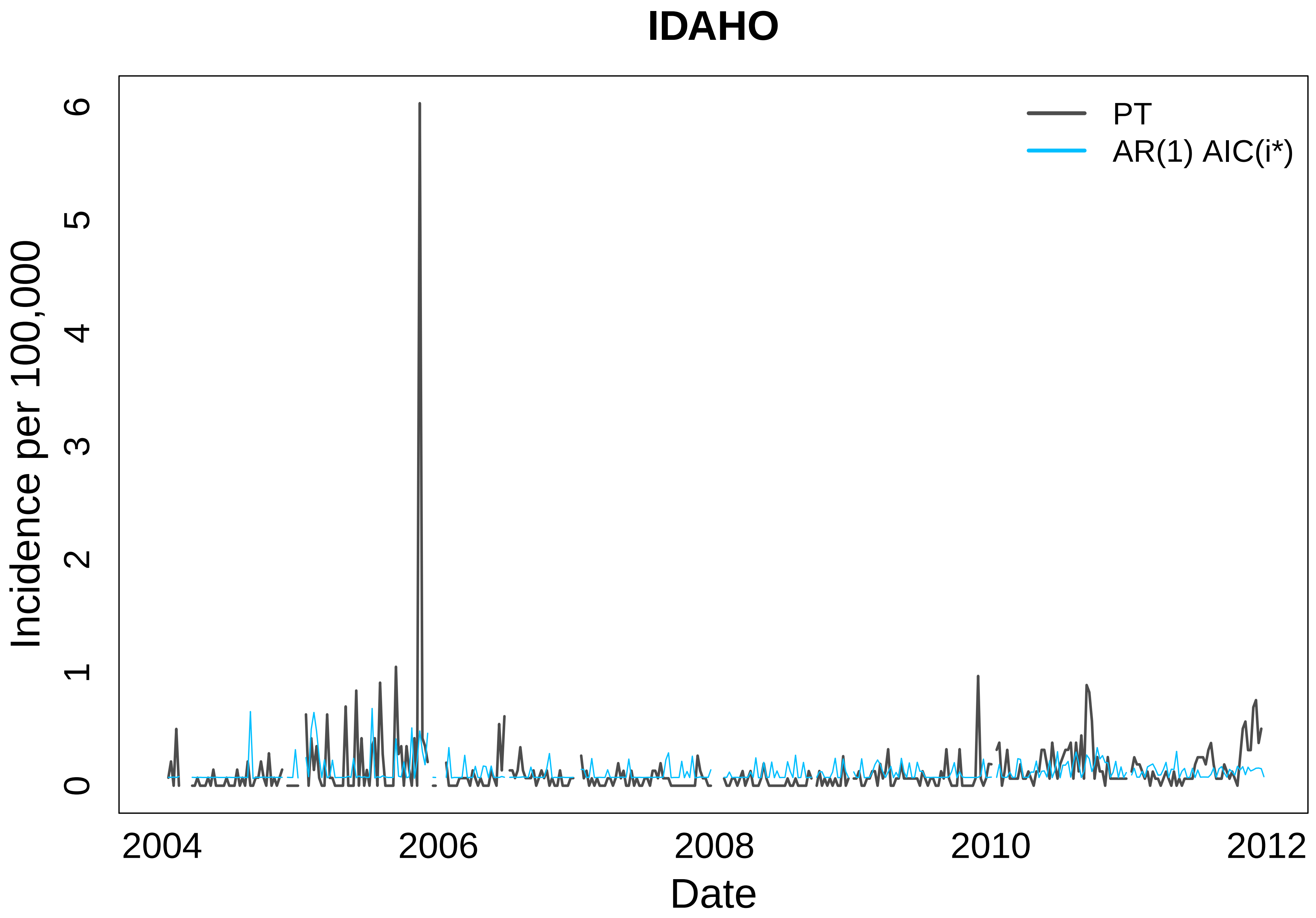
IDAHO
PT
AR(1) AIC(i*)
Incidence per 100,000
0
1
2
3
4
5
6
2004
2006
2008
2010
2012
Date

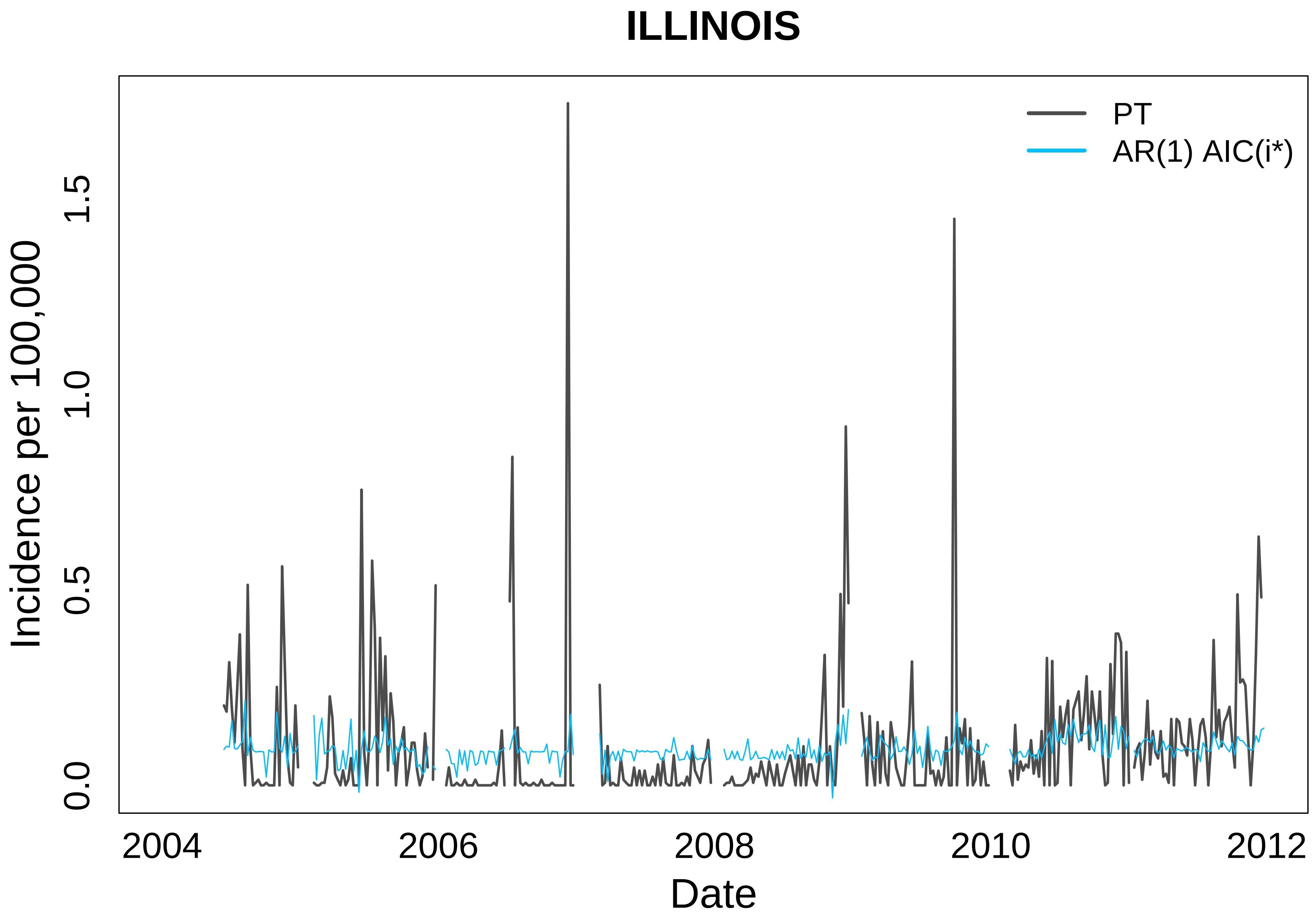

ILLINOIS
PT
AR(1) AIC(i*)
Incidence per 100,000
0.0
0.5
1.0
1.5
2004
2006
2008
2010
2012
Date

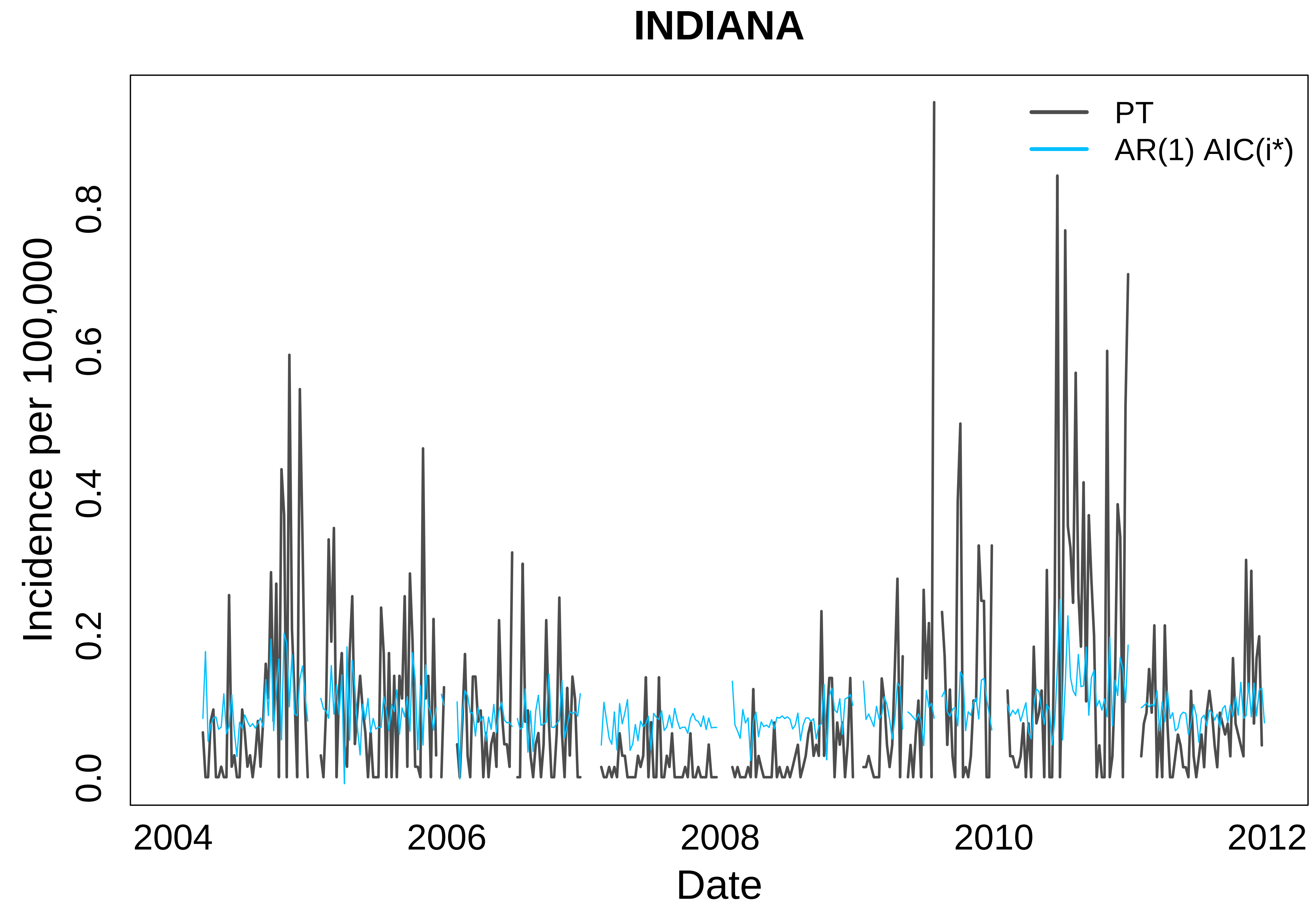

INDIANA
PT
AR(1) AIC(i*)
Incidence per 100,000
0.0
0.2
0.4
0.6
0.8
2004
2006
2008
2010
2012
Date

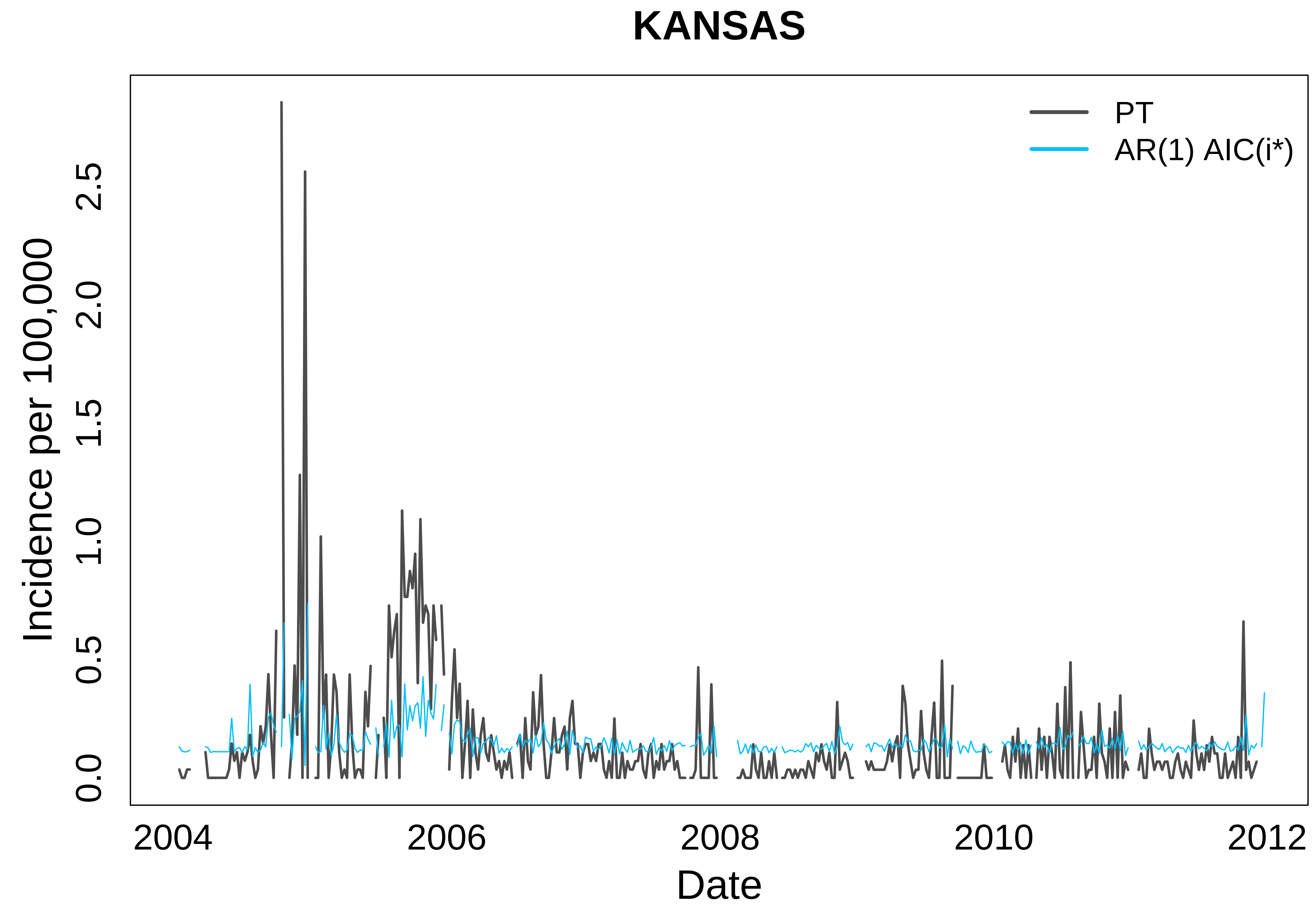

KANSAS
PT
AR(1) AIC(i*)
Incidence per 100,000
0.0
0.5
1.0
1.5
2.0
2.5
2004
2006
2008
2010
2012
Date

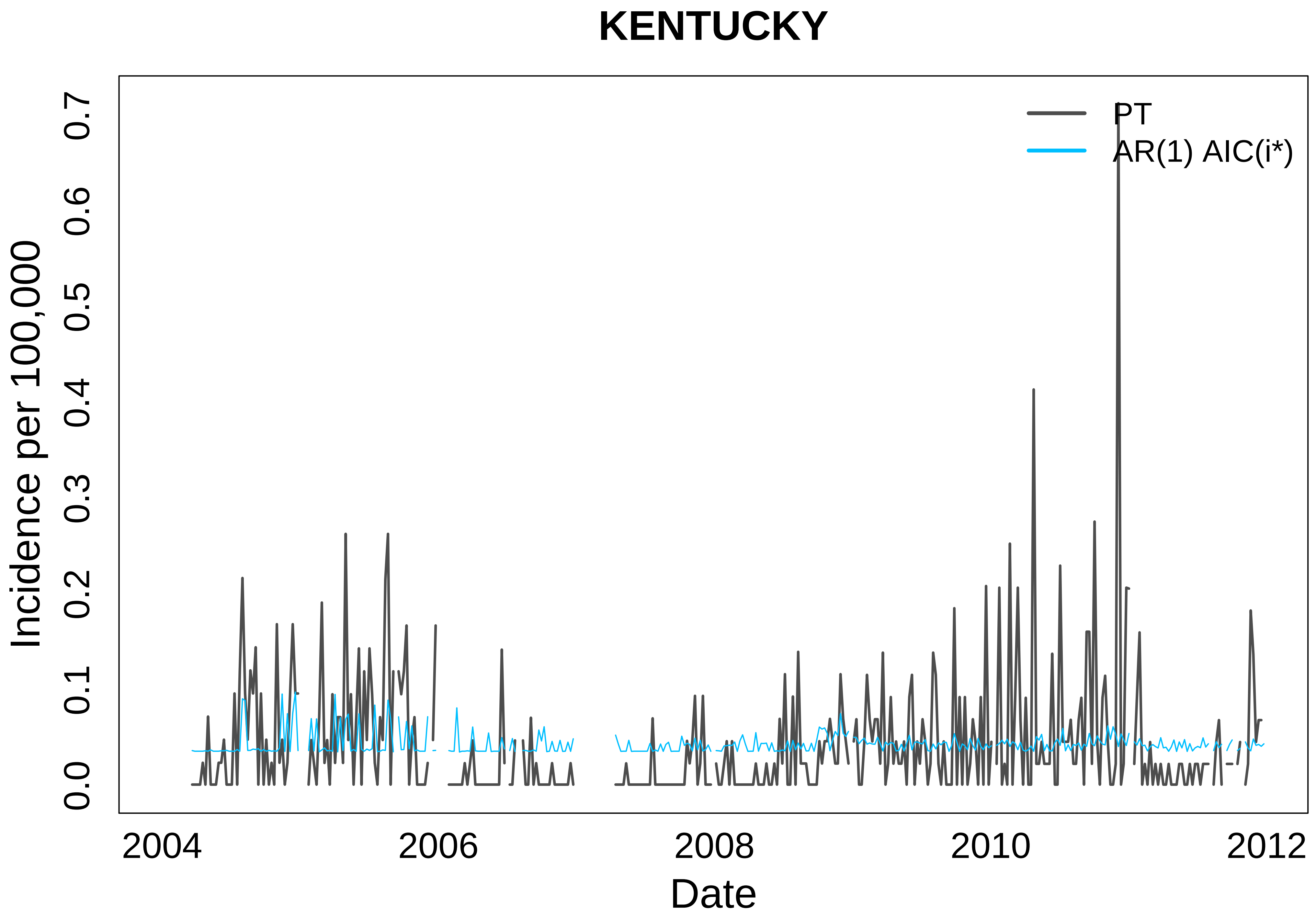
KENTUCKY
PT
AR(1) AIC(i*)
Incidence per 100,000
0.0
0.1
0.2
0.3
0.4
0.5
0.6
0.7
2004
2006
2008
2010
2012
Date

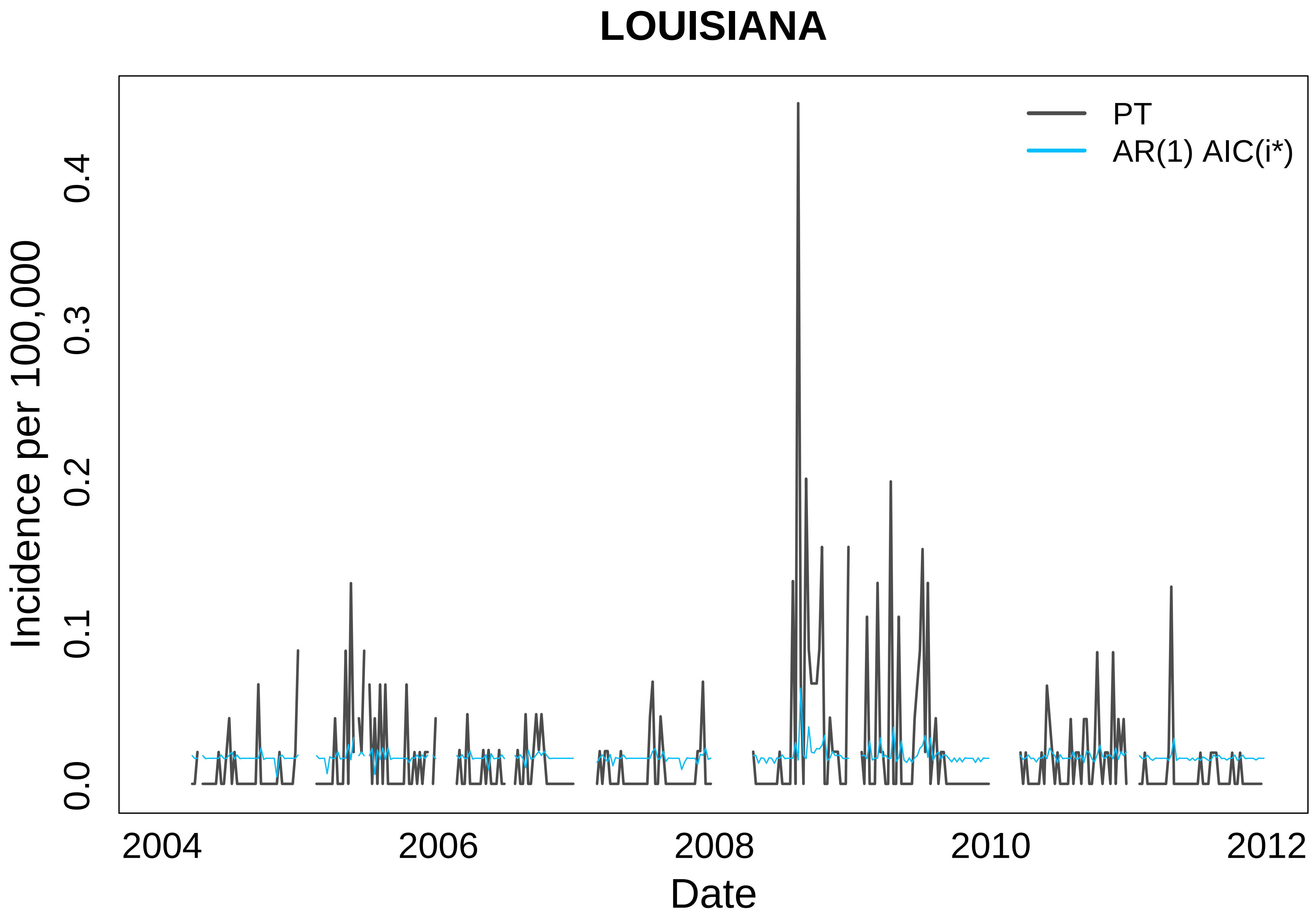
LOUISIANA
PT
AR(1) AIC(i*)
Incidence per 100,000
0.0
0.1
0.2
0.3
0.4
2004
2006
2008
2010
2012
Date

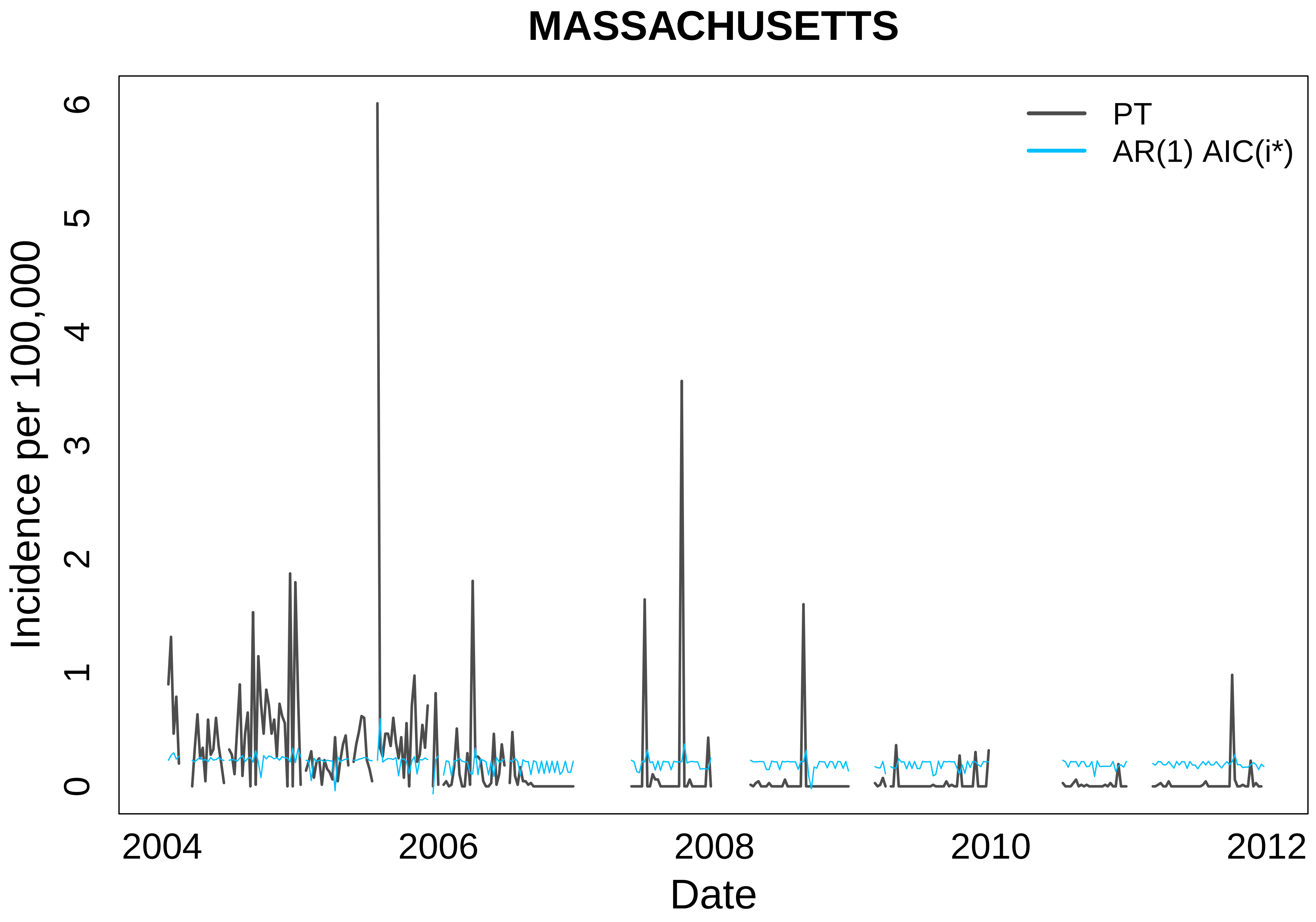
MASSACHUSETTS
PT
AR(1) AIC(i*)
Incidence per 100,000
0
1
2
3
4
5
6
2004
2006
2008
2010
2012
Date

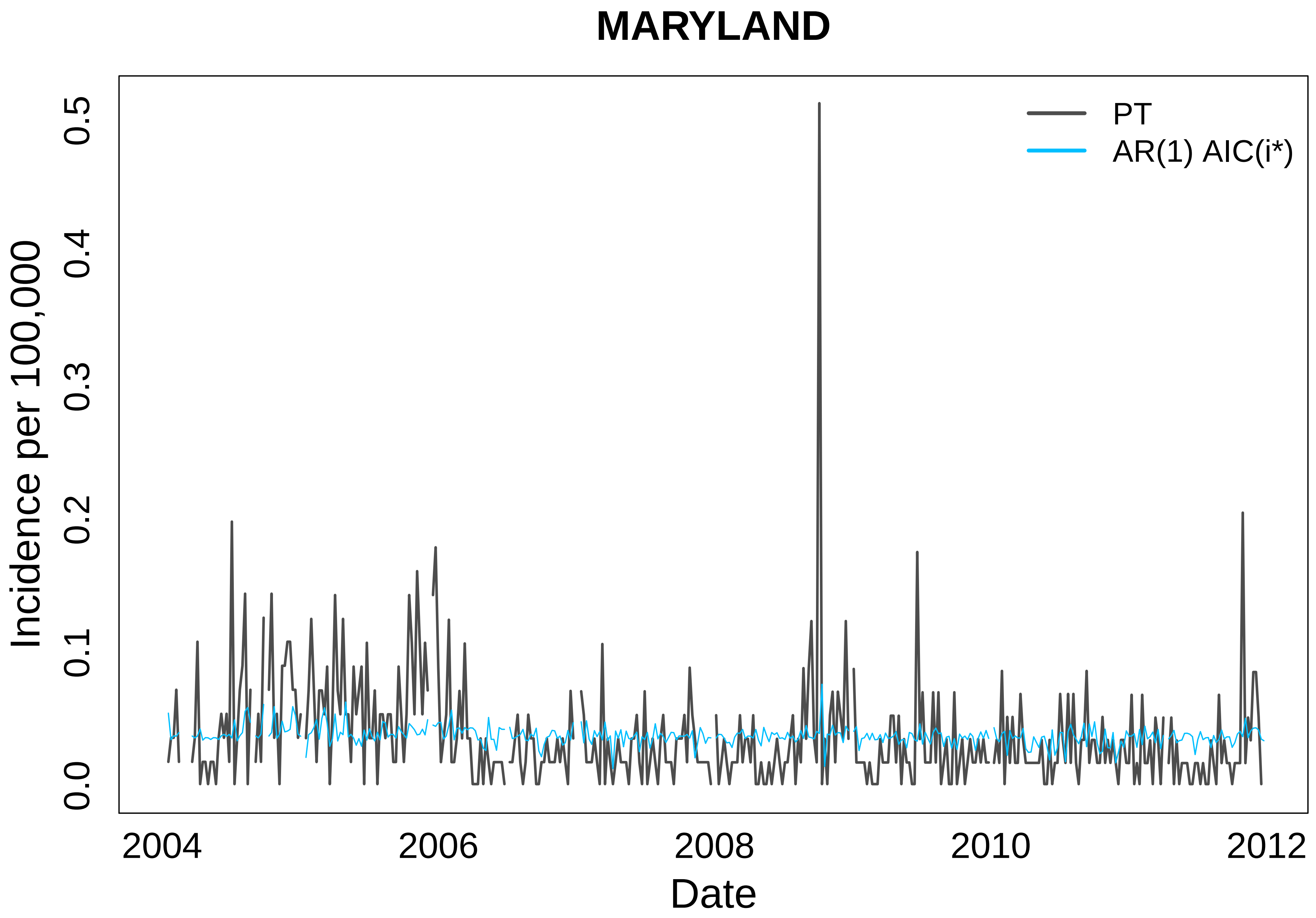

MARYLAND
PT
AR(1) AIC(i*)
Incidence per 100,000
0.0
0.1
0.2
0.3
0.4
0.5
2004
2006
2008
2010
2012
Date

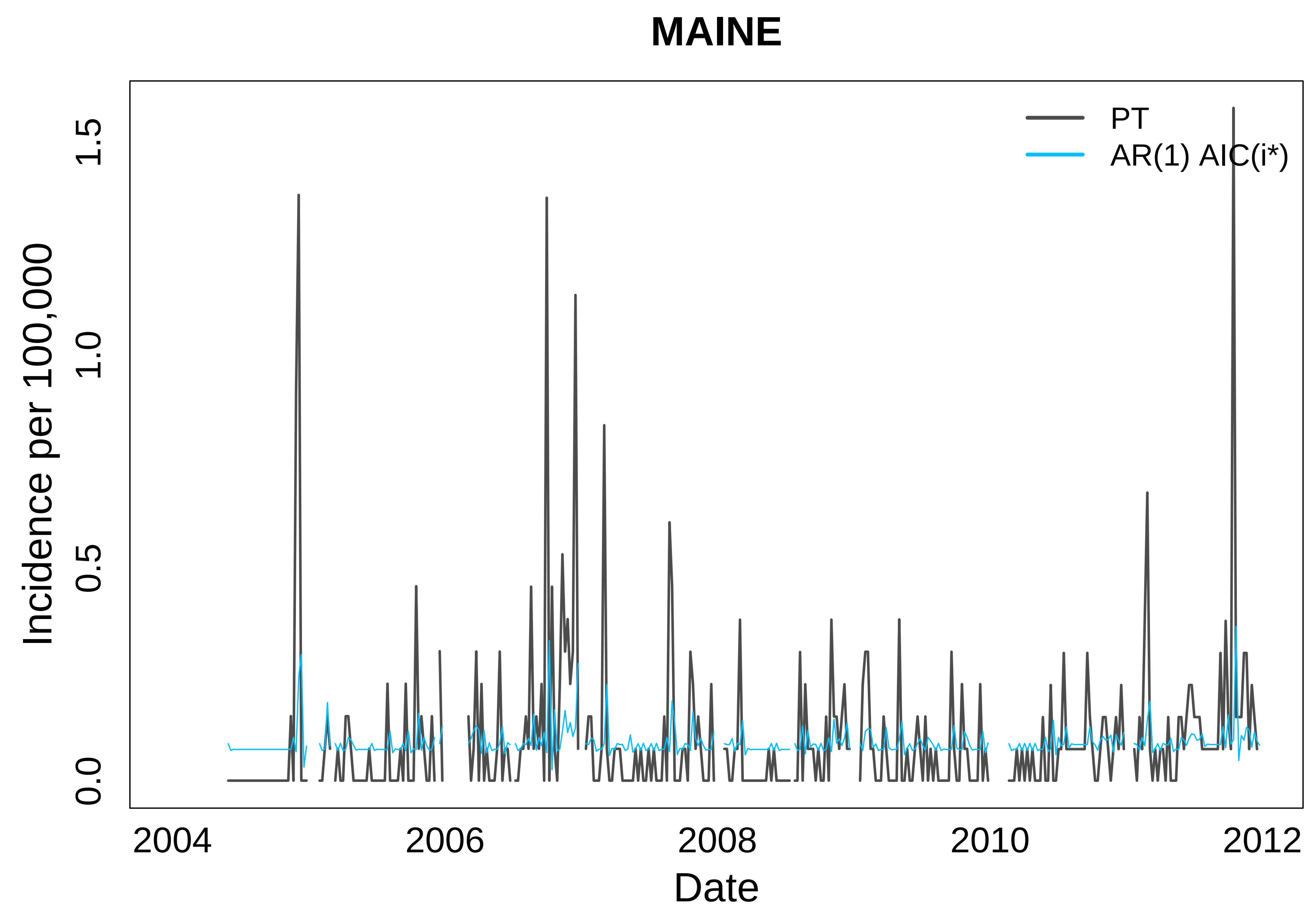
MAINE
PT
AR(1) AIC(i*)
Incidence per 100,000
0.0
0.5
1.0
1.5
2004
2006
2008
2010
2012
Date

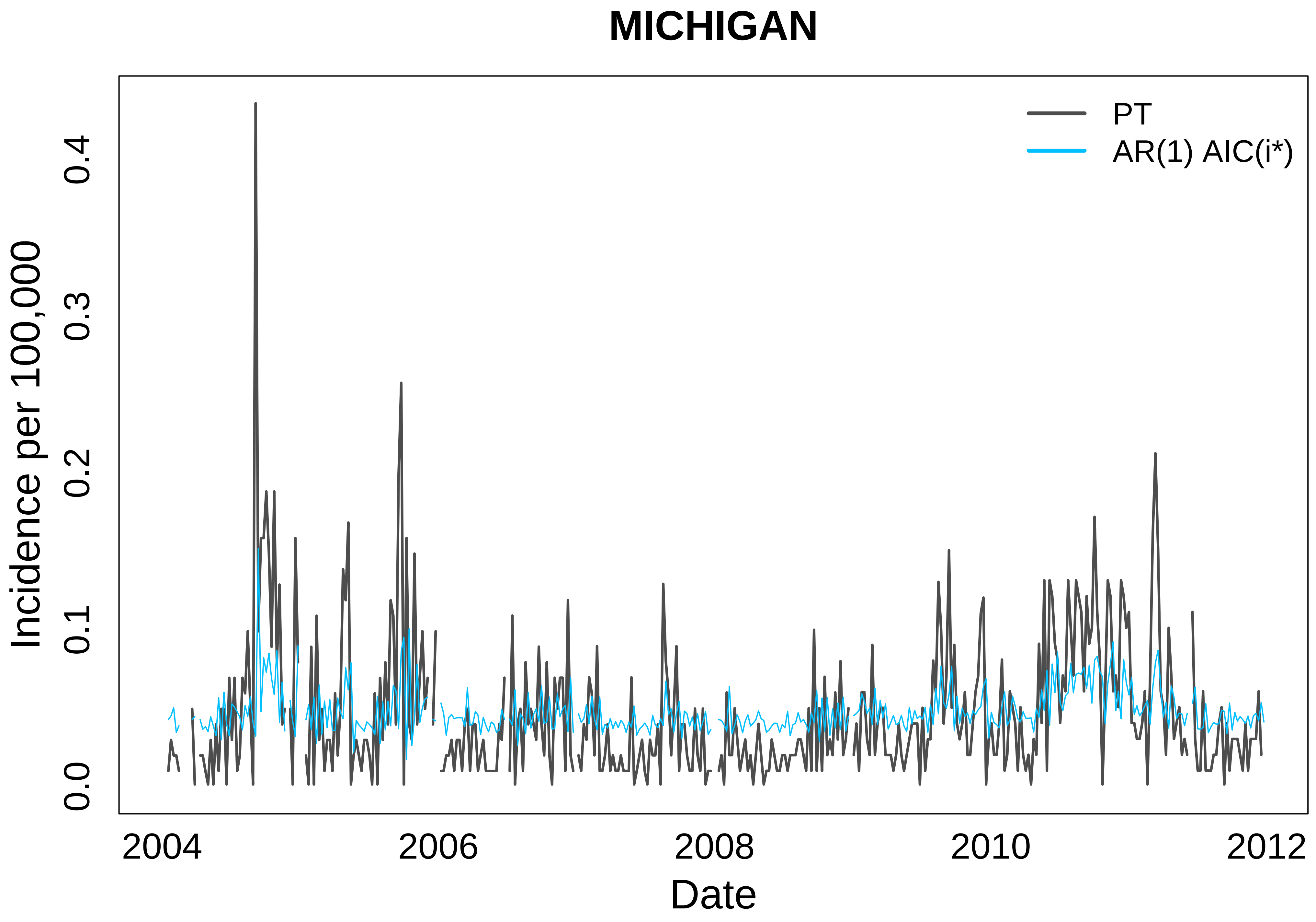

MICHIGAN
PT
AR(1) AIC(i*)
Incidence per 100,000
0.0
0.1
0.2
0.3
0.4
2004
2006
2008
2010
2012
Date

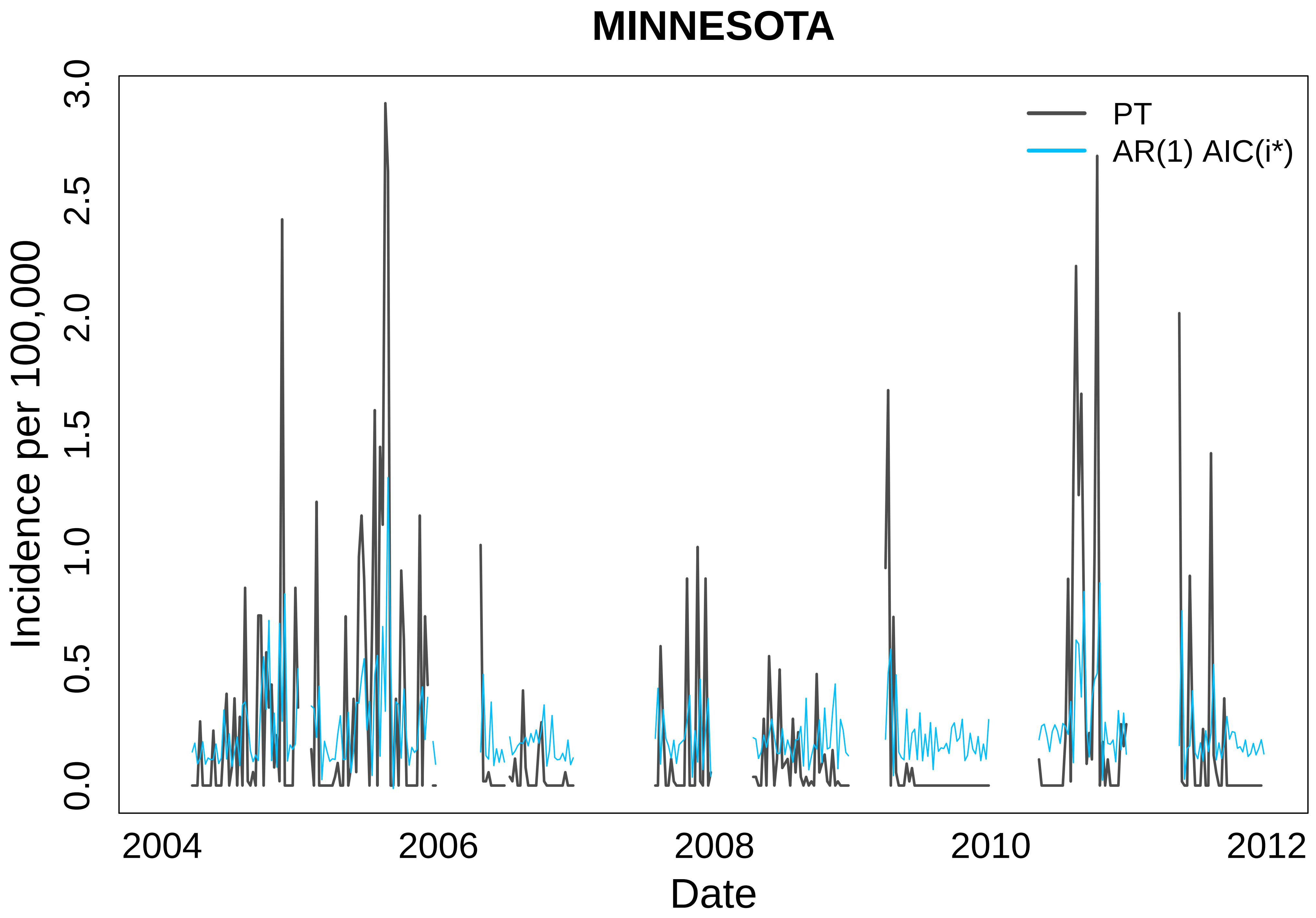
MINNESOTA
PT
AR(1) AIC(i*)
Incidence per 100,000
0.0
0.5
1.0
1.5
2.0
2.5
3.0
2004
2006
2008
2010
2012
Date

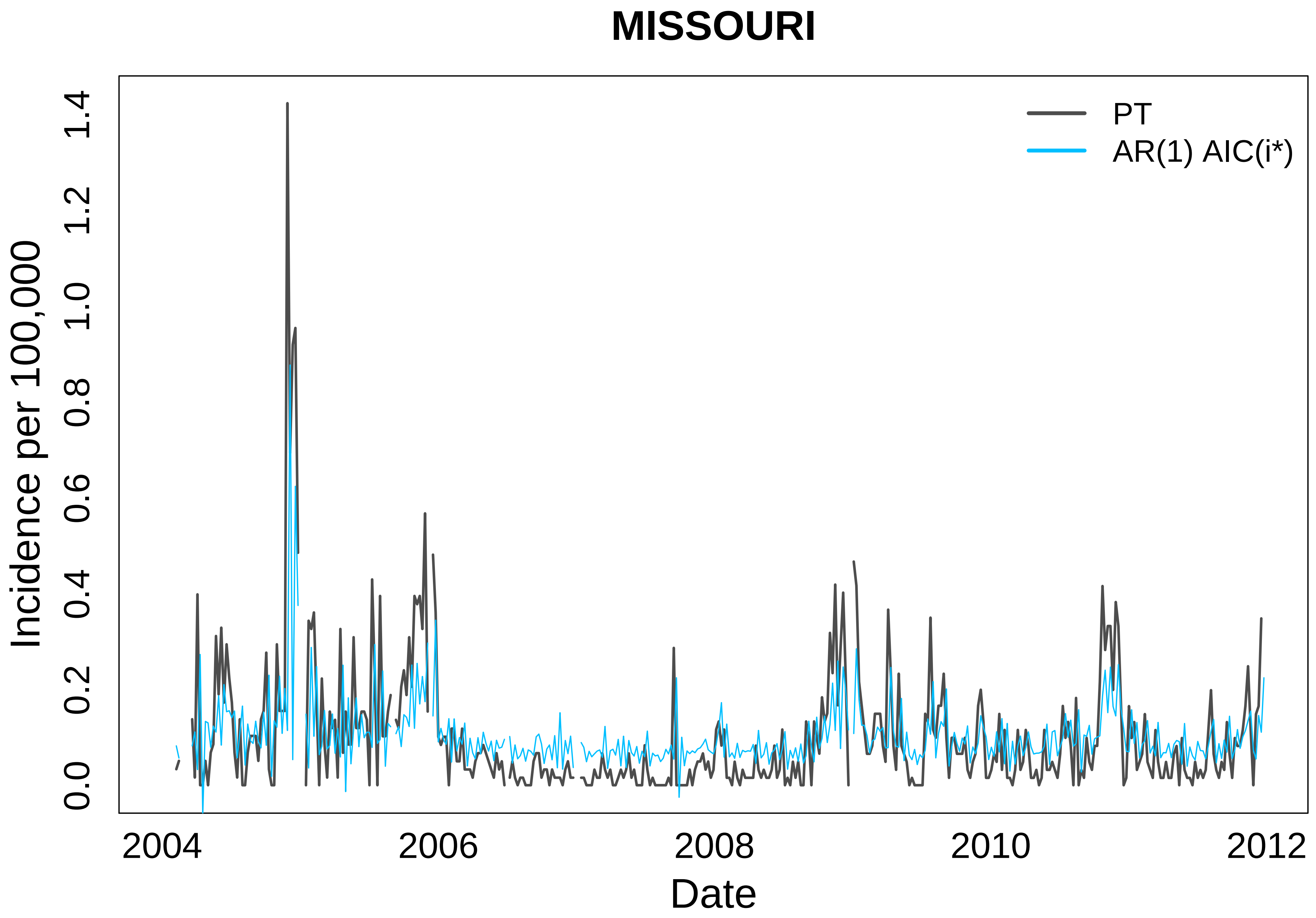
MISSOURI
PT
AR(1) AIC(i*)
Incidence per 100,000
0.0
0.2
0.4
0.6
0.8
1.0
1.2
1.4
2004
2006
2008
2010
2012
Date

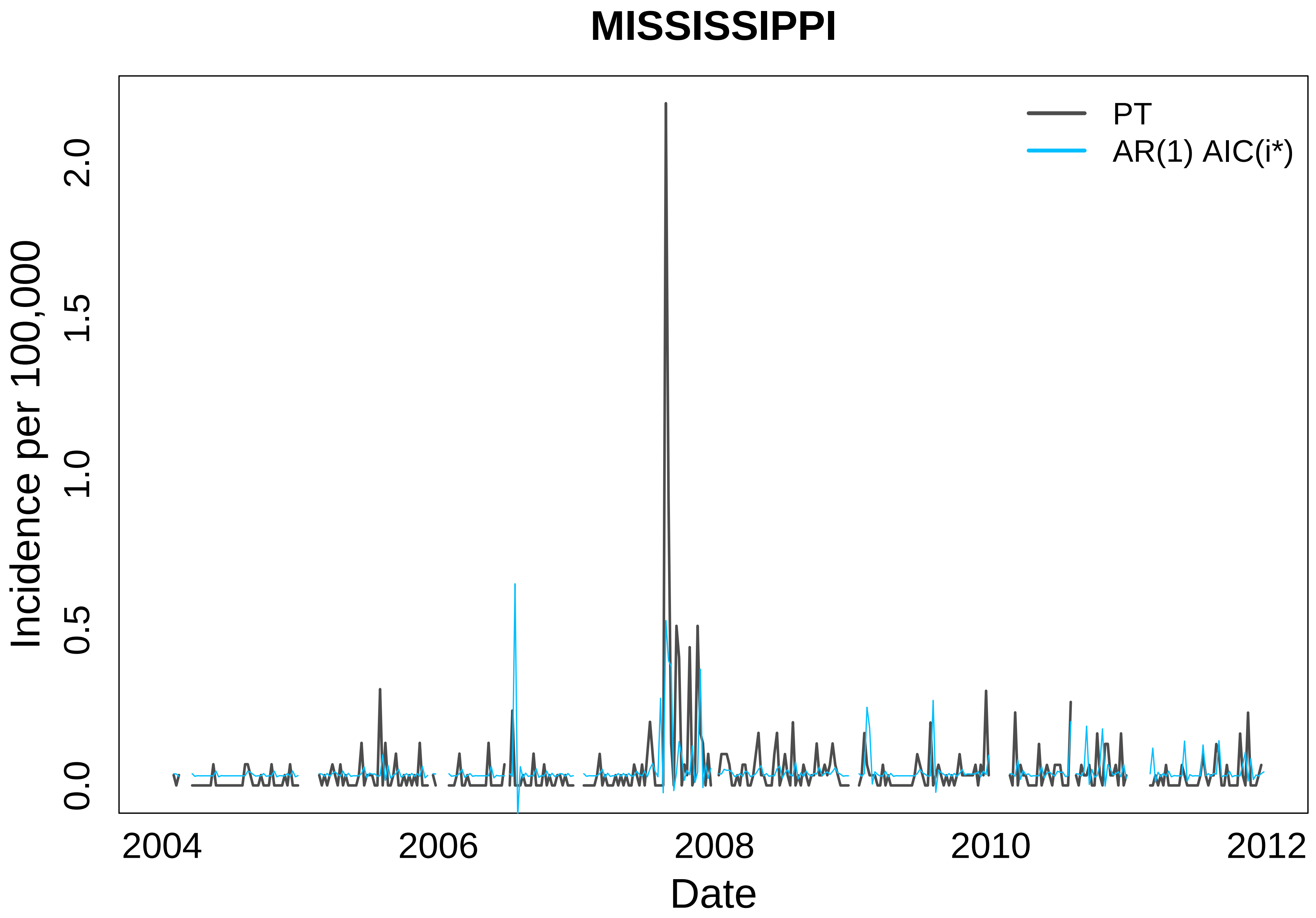

MISSISSIPPI
PT
AR(1) AIC(i*)
Incidence per 100,000
0.0
0.5
1.0
1.5
2.0
2004
2006
2008
2010
2012
Date

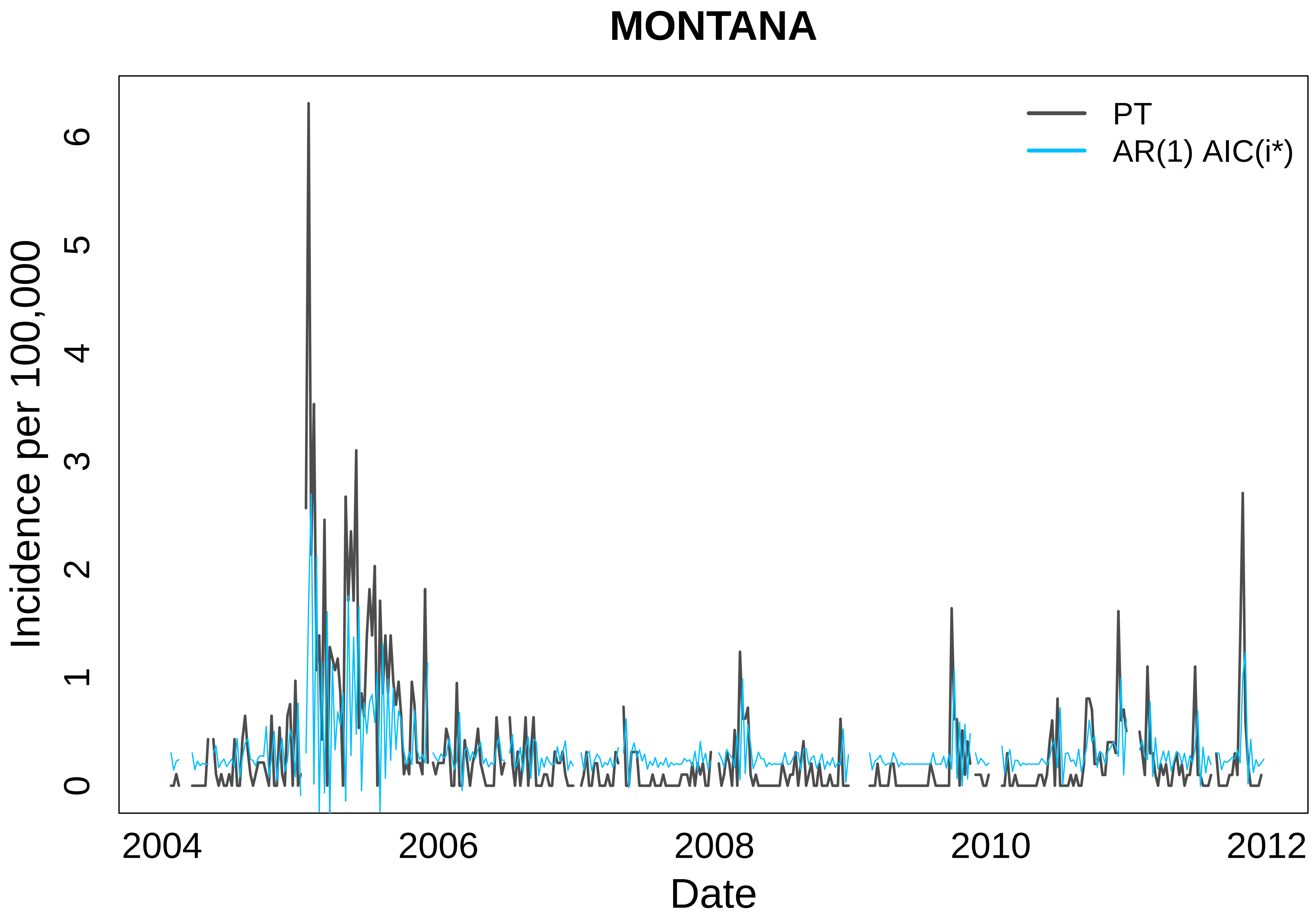

MONTANA
PT
AR(1) AIC(i*)
Incidence per 100,000
0
1
2
3
4
5
6
2004
2006
2008
2010
2012
Date

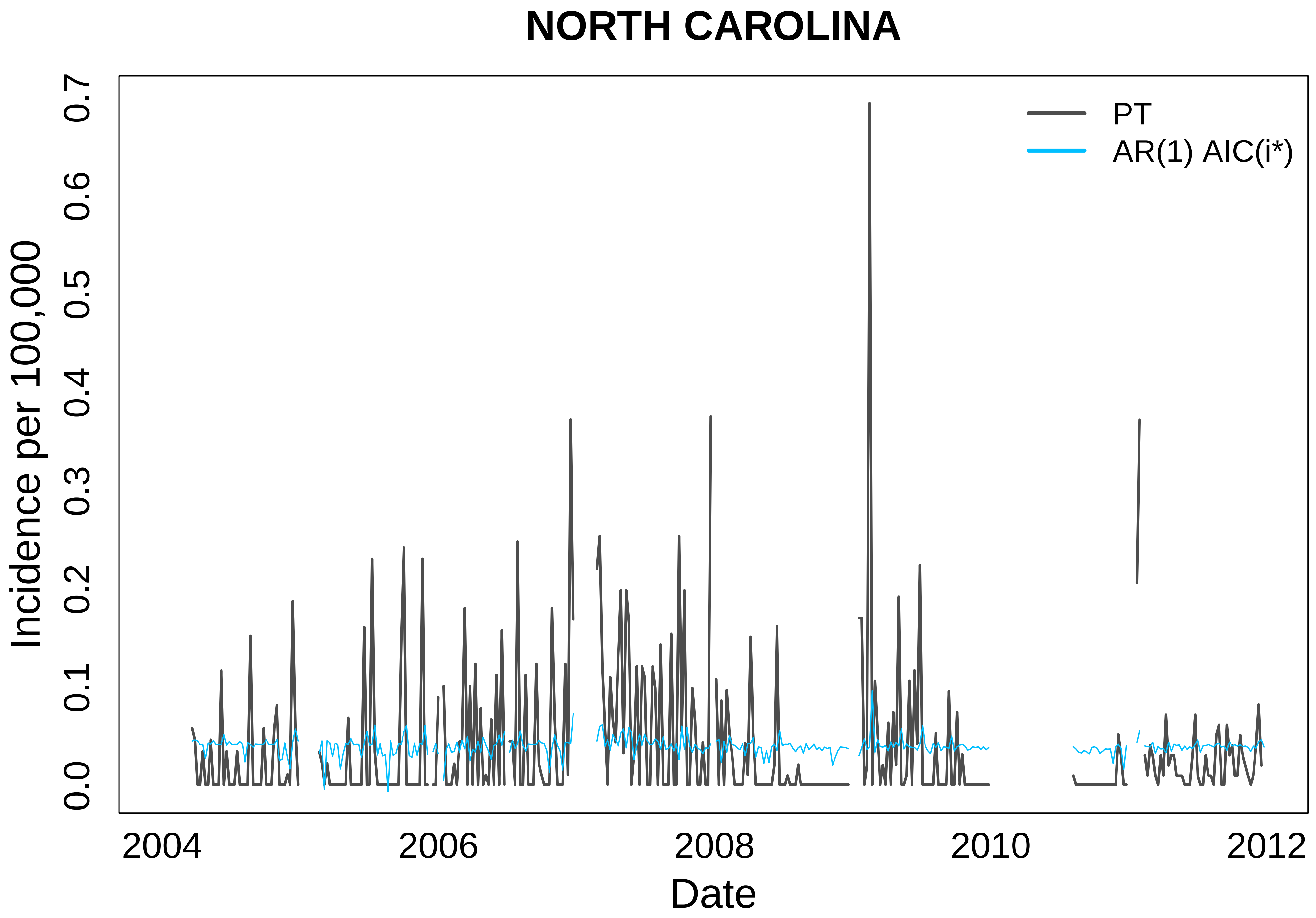
NORTH CAROLINA
PT
AR(1) AIC(i*)
Incidence per 100,000
0.0
0.1
0.2
0.3
0.4
0.5
0.6
0.7
2004
2006
2008
2010
2012
Date

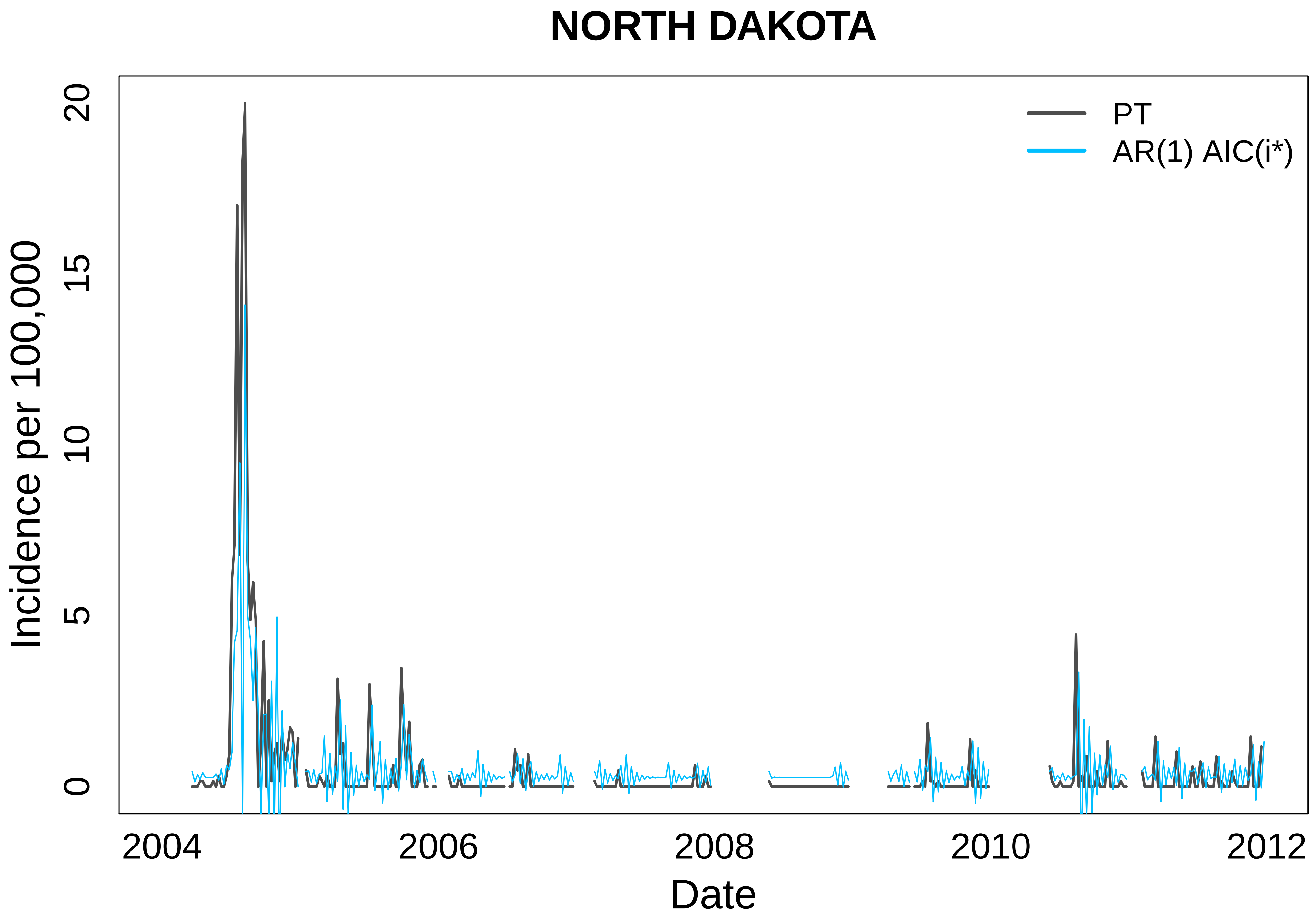

NORTH DAKOTA
PT
AR(1) AIC(i*)
Incidence per 100,000
0
5
10
15
20
2004
2006
2008
2010
2012
Date

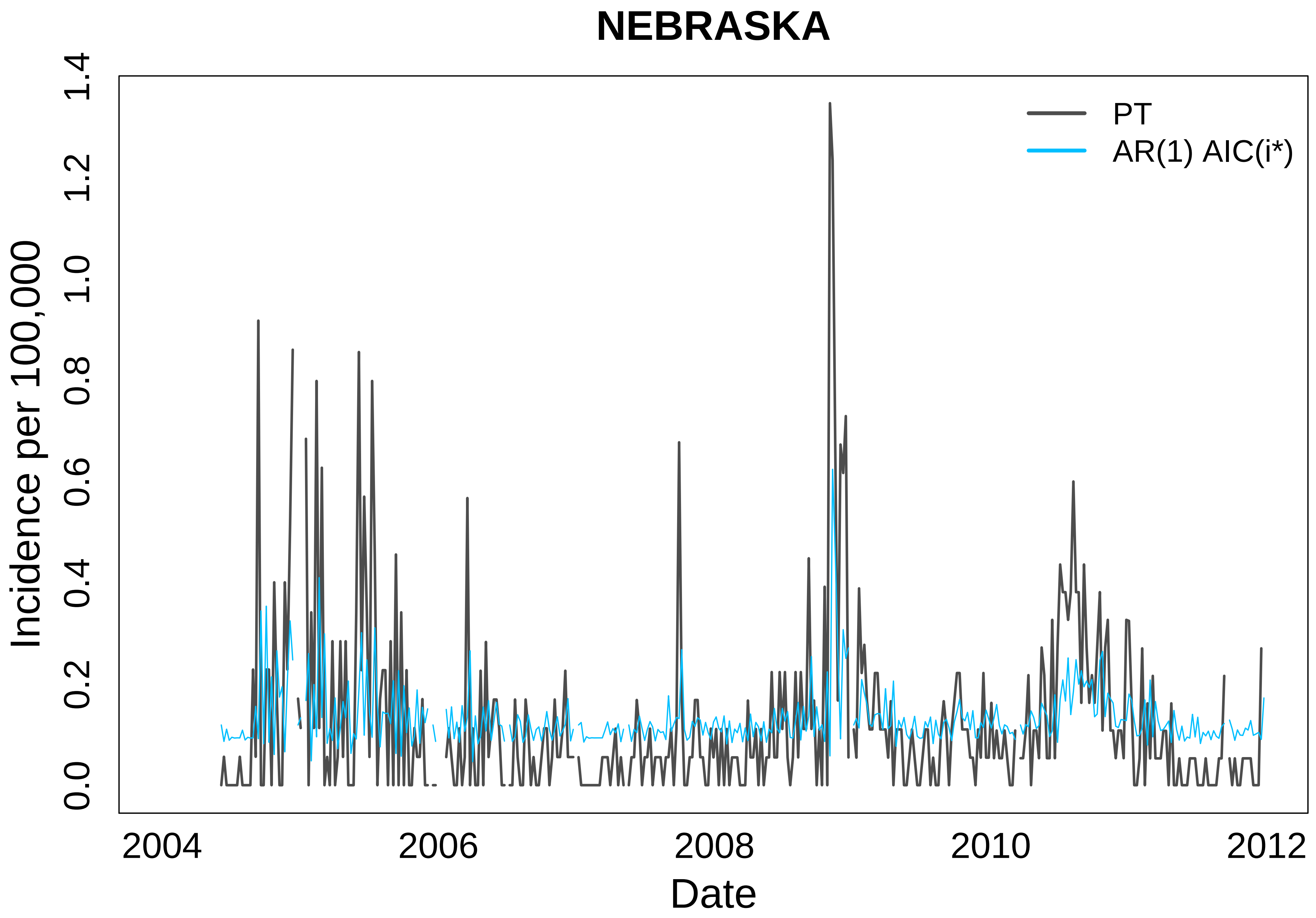

NEBRASKA
PT
AR(1) AIC(i*)
Incidence per 100,000
0.0
0.2
0.4
0.6
0.8
1.0
1.2
1.4
2004
2006
2008
2010
2012
Date

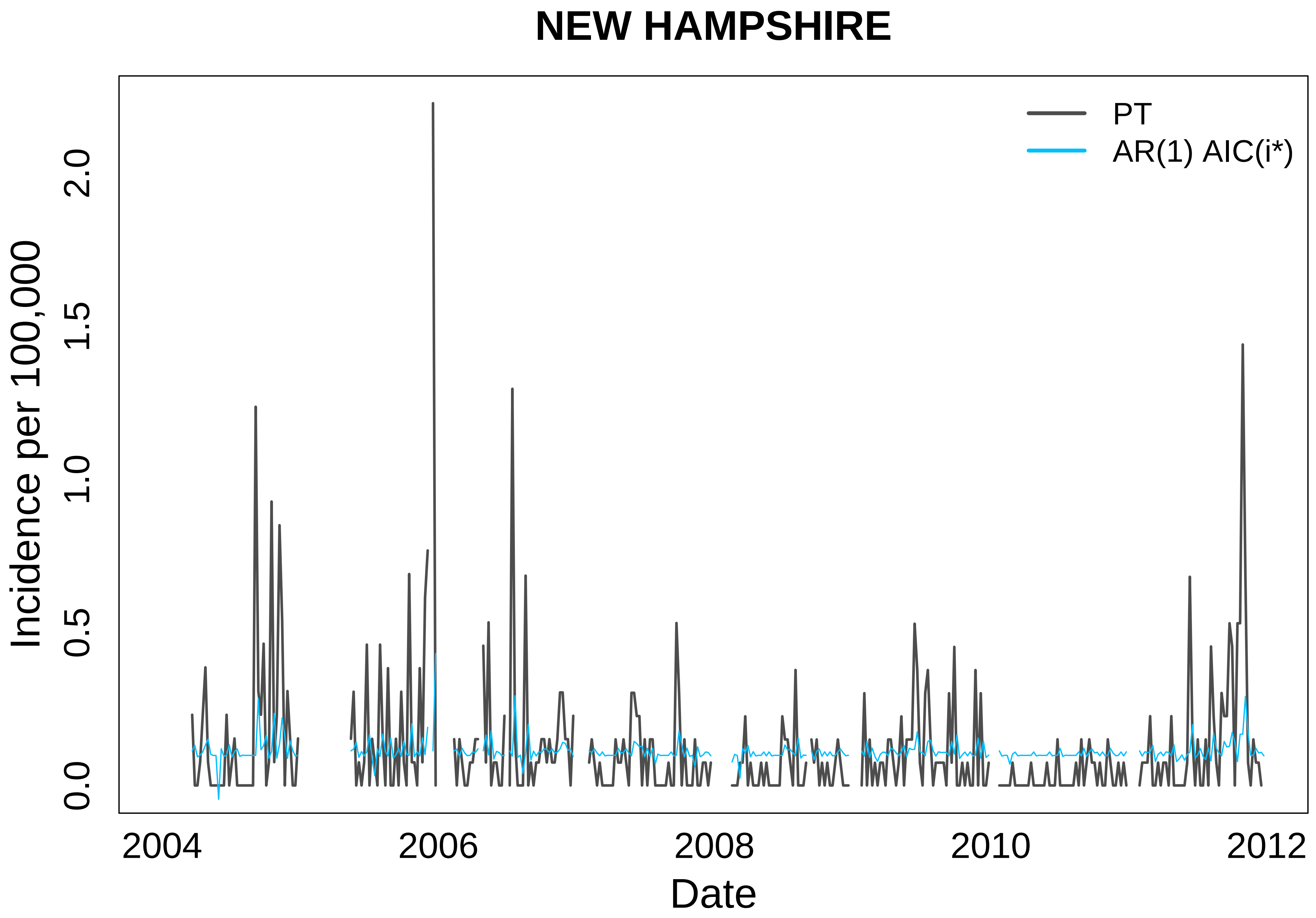

NEW HAMPSHIRE
PT
AR(1) AIC(i*)
Incidence per 100,000
0.0
0.5
1.0
1.5
2.0
2004
2006
2008
2010
2012
Date

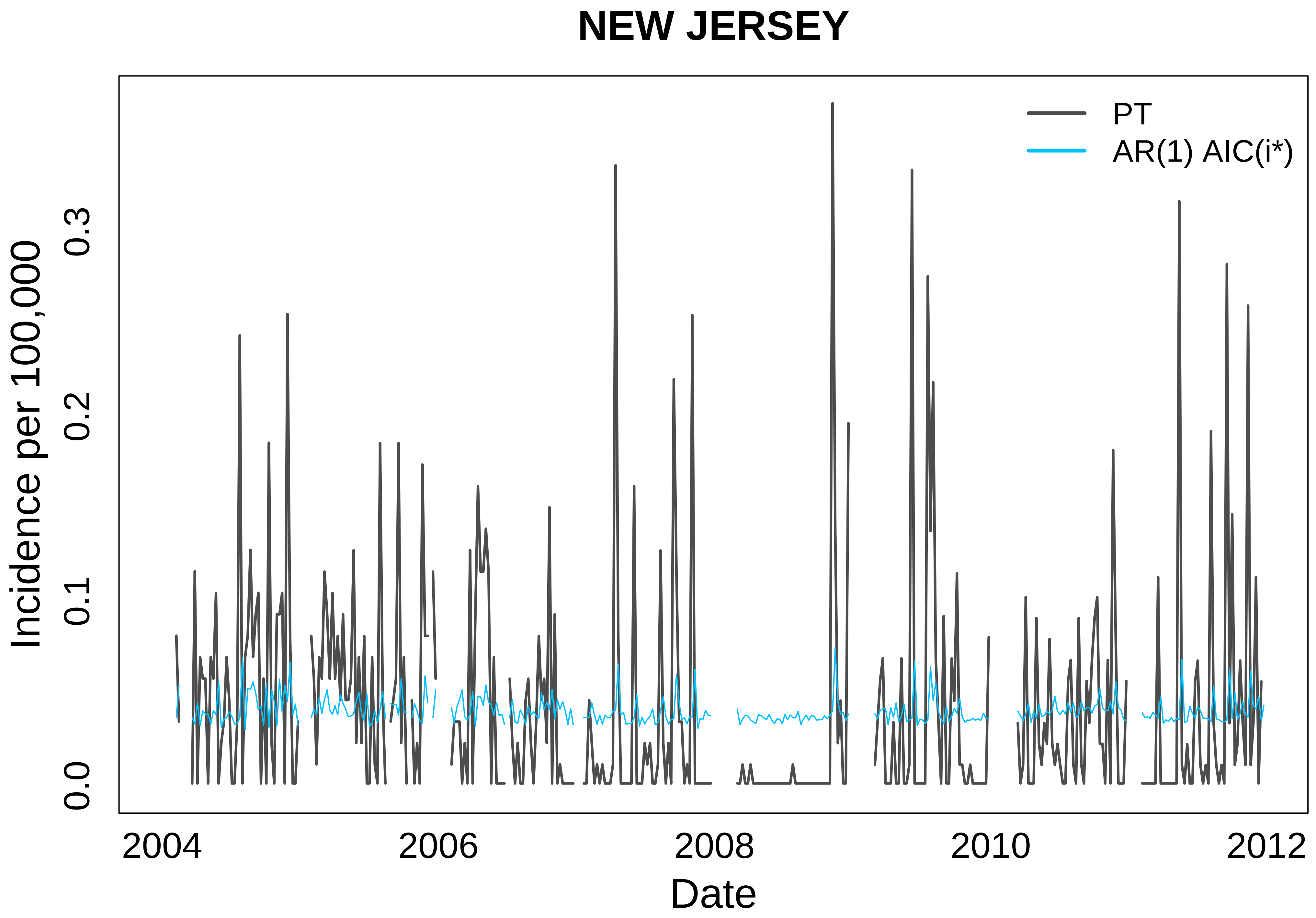
NEW JERSEY
PT
AR(1) AIC(i*)
Incidence per 100,000
0.0
0.1
0.2
0.3
2004
2006
2008
2010
2012
Date

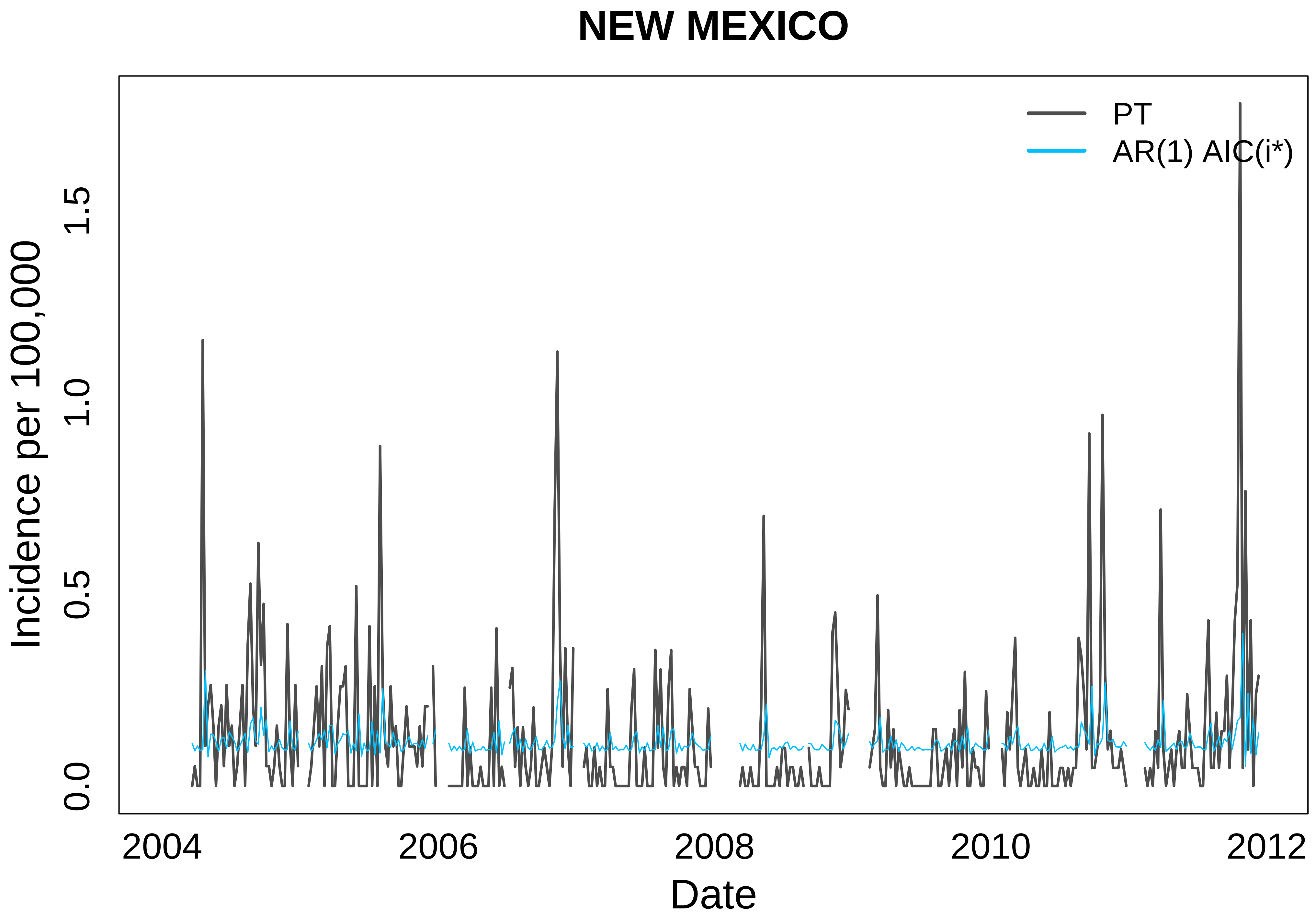
NEW MEXICO
PT
AR(1) AIC(i*)
Incidence per 100,000
0.0
0.5
1.0
1.5
2004
2006
2008
2010
2012
Date

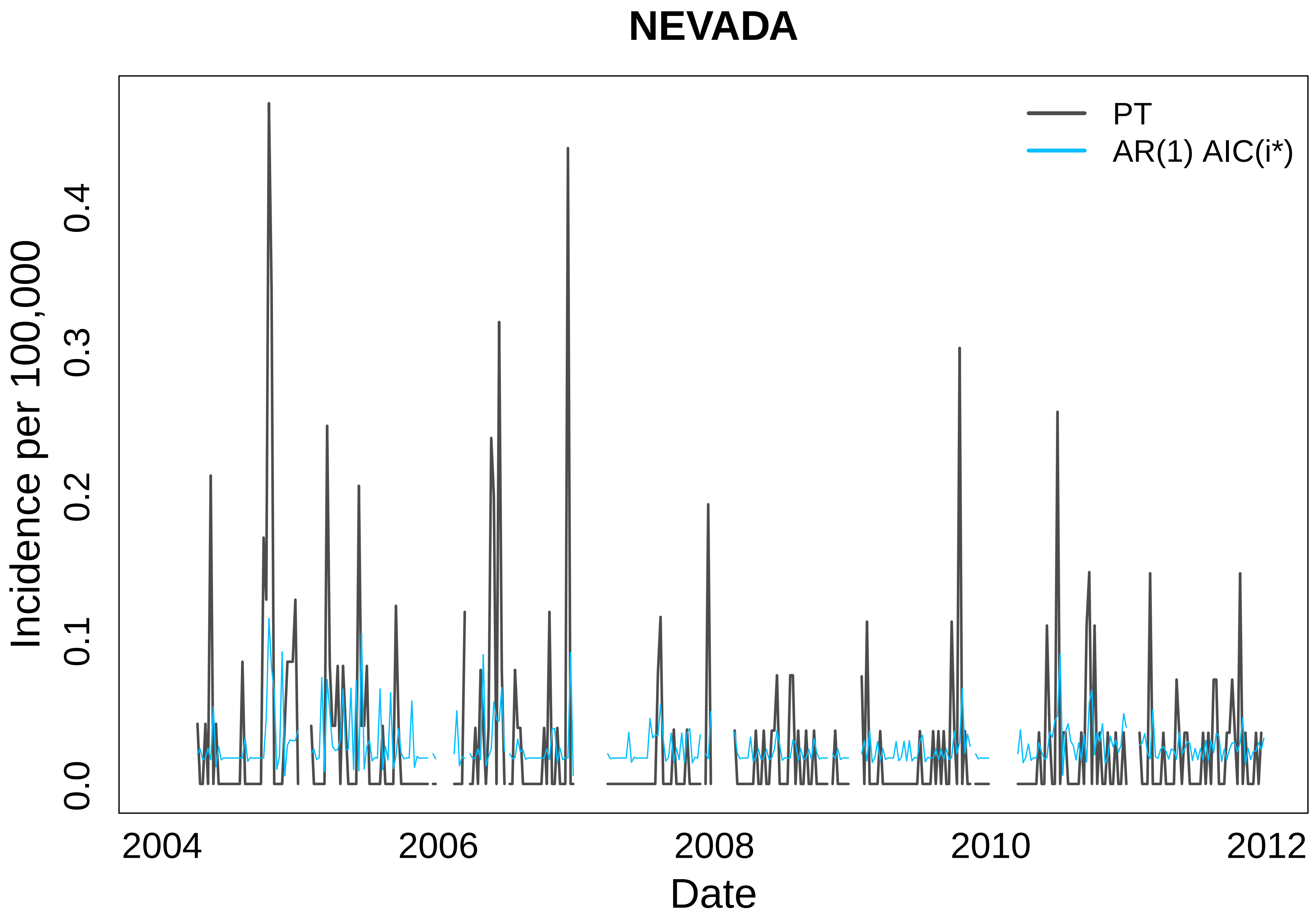

NEVADA
PT
AR(1) AIC(i*)
Incidence per 100,000
0.0
0.1
0.2
0.3
0.4
2004
2006
2008
2010
2012
Date

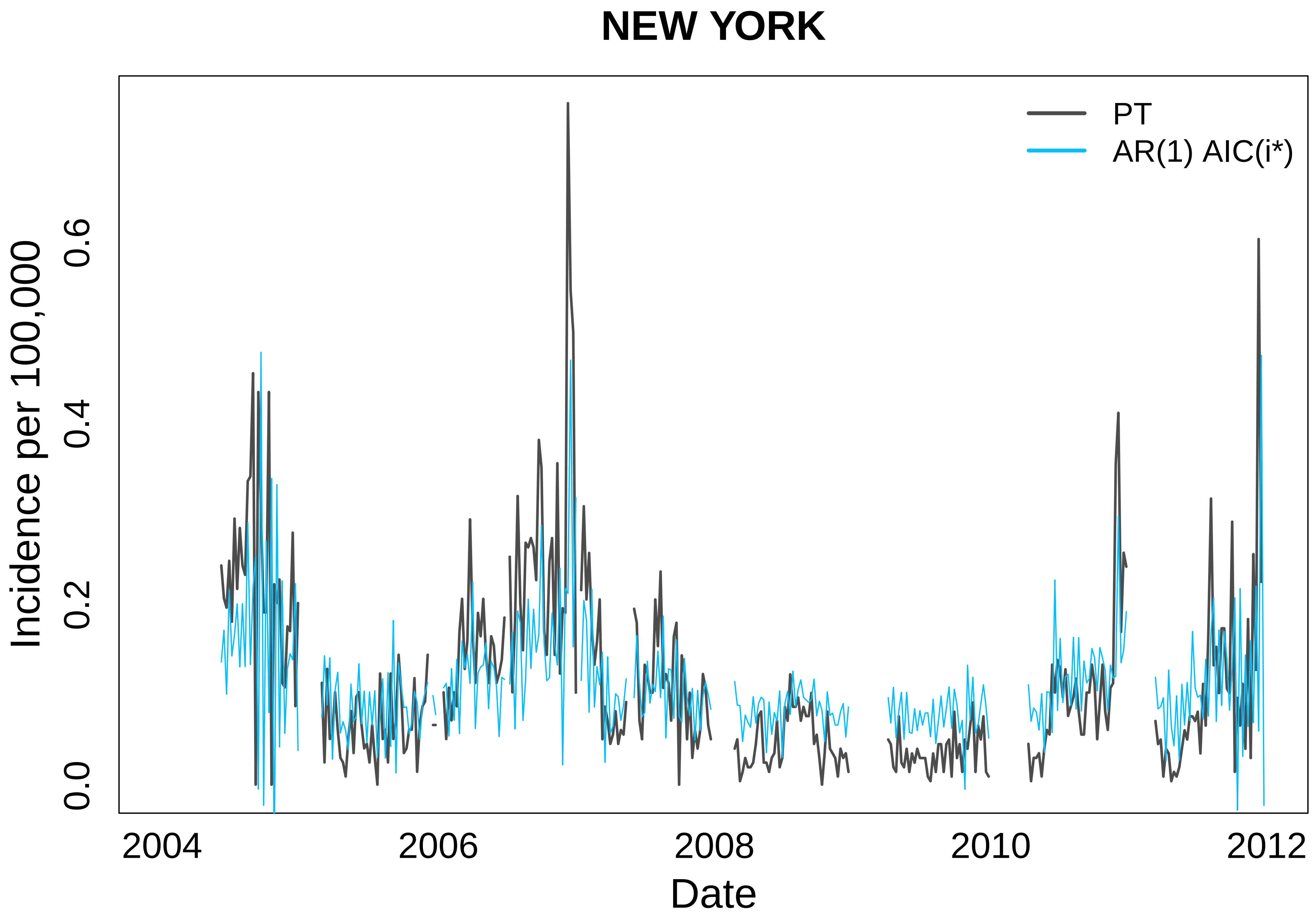
NEW YORK
PT
AR(1) AIC(i*)
Incidence per 100,000
0.0
0.2
0.4
0.6
2004
2006
2008
2010
2012
Date

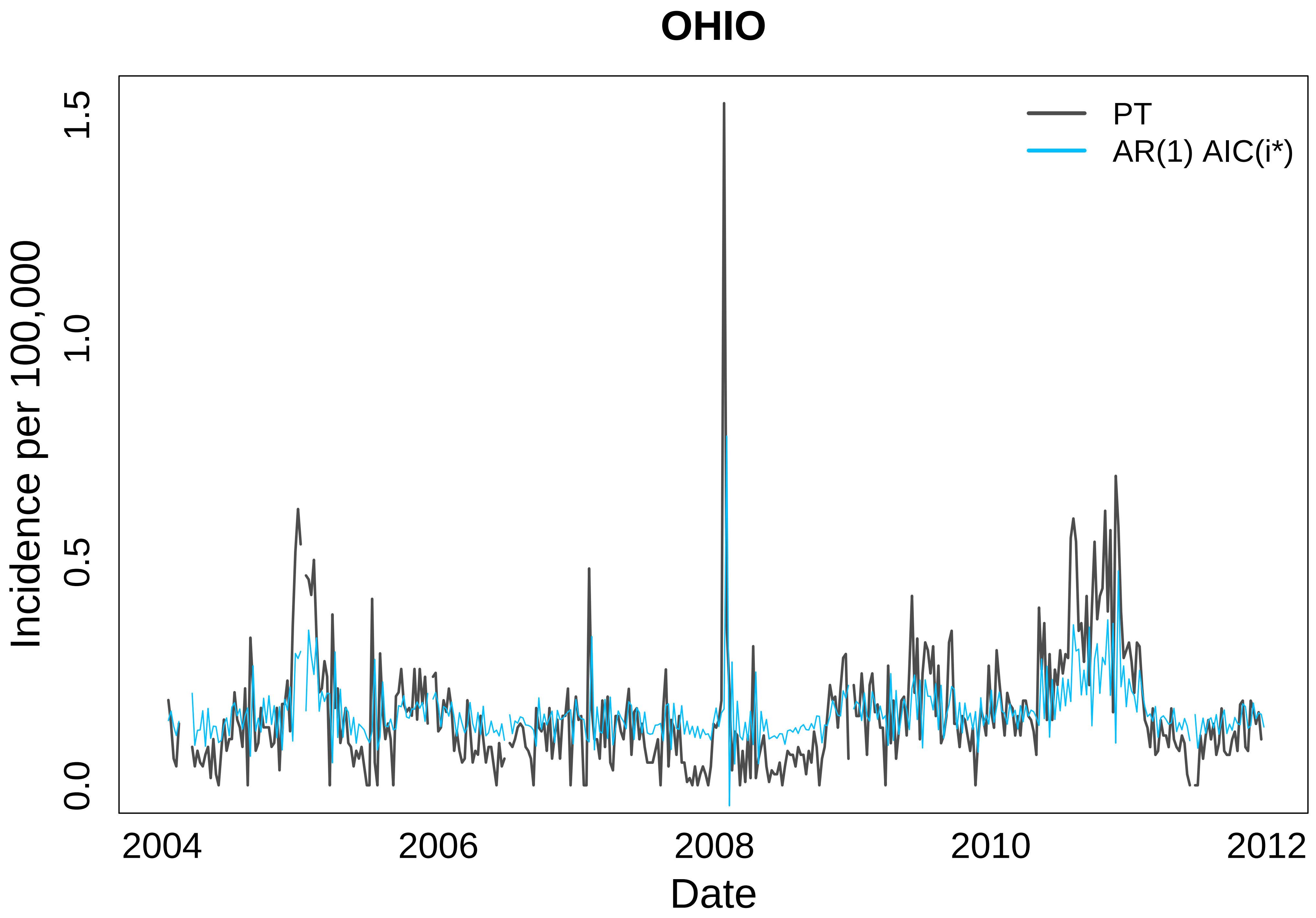
OHIO
PT
AR(1) AIC(i*)
Incidence per 100,000
0.0
0.5
1.0
1.5
2004
2006
2008
2010
2012
Date

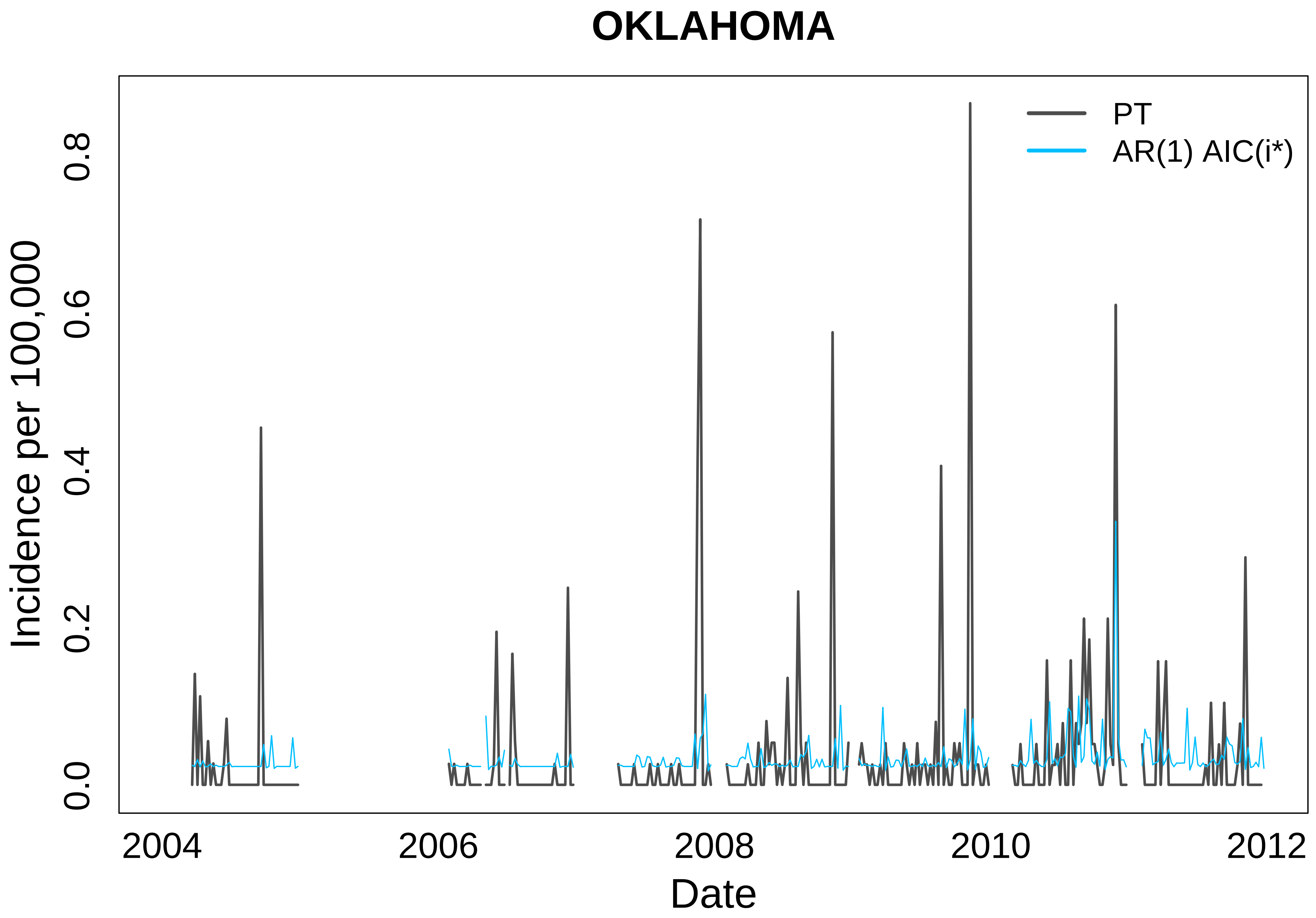
OKLAHOMA
PT
AR(1) AIC(i*)
Incidence per 100,000
0.0
0.2
0.4
0.6
0.8
2004
2006
2008
2010
2012
Date

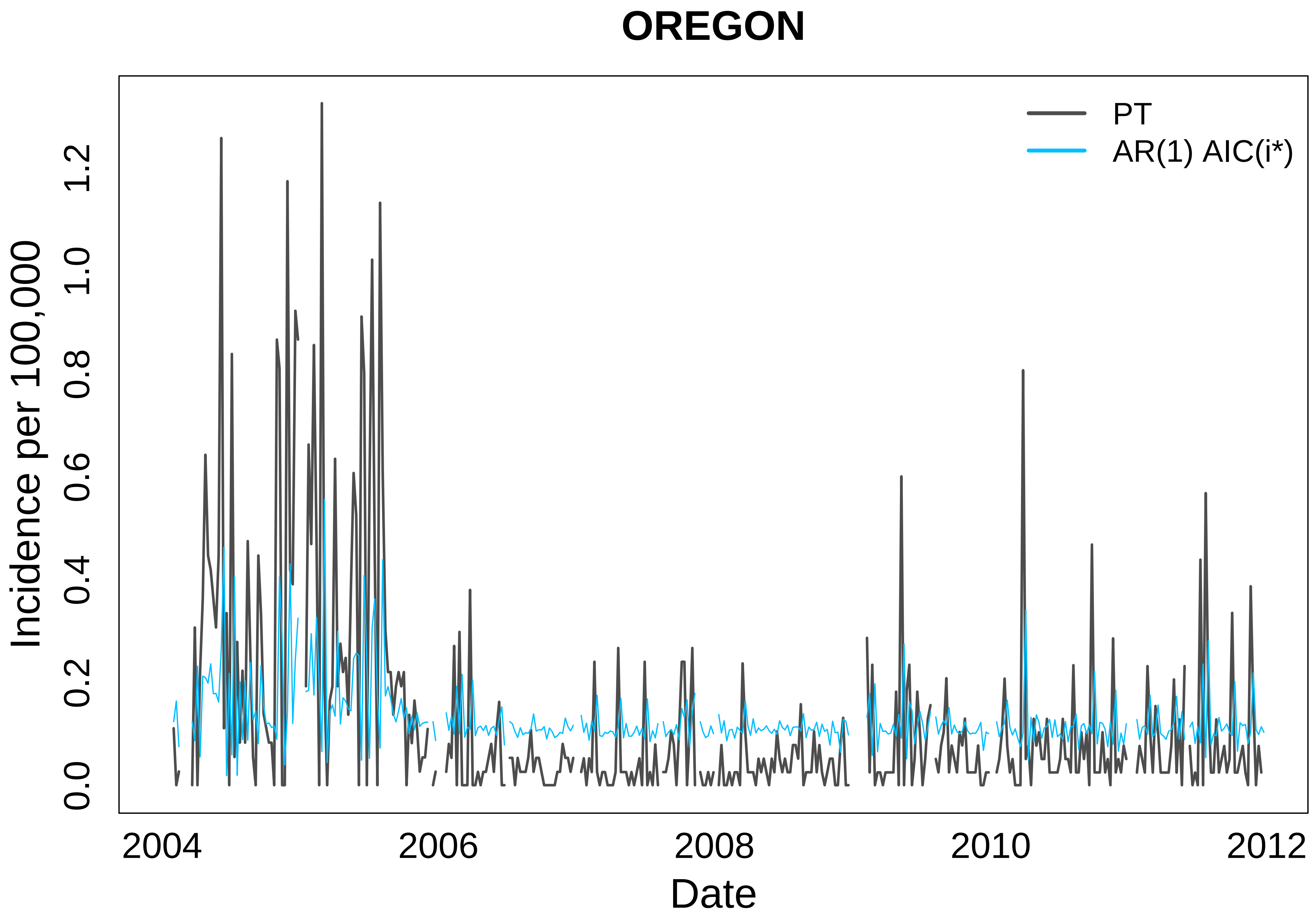

OREGON
PT
AR(1) AIC(i*)
Incidence per 100,000
0.0
0.2
0.4
0.6
0.8
1.0
1.2
2004
2006
2008
2010
2012
Date

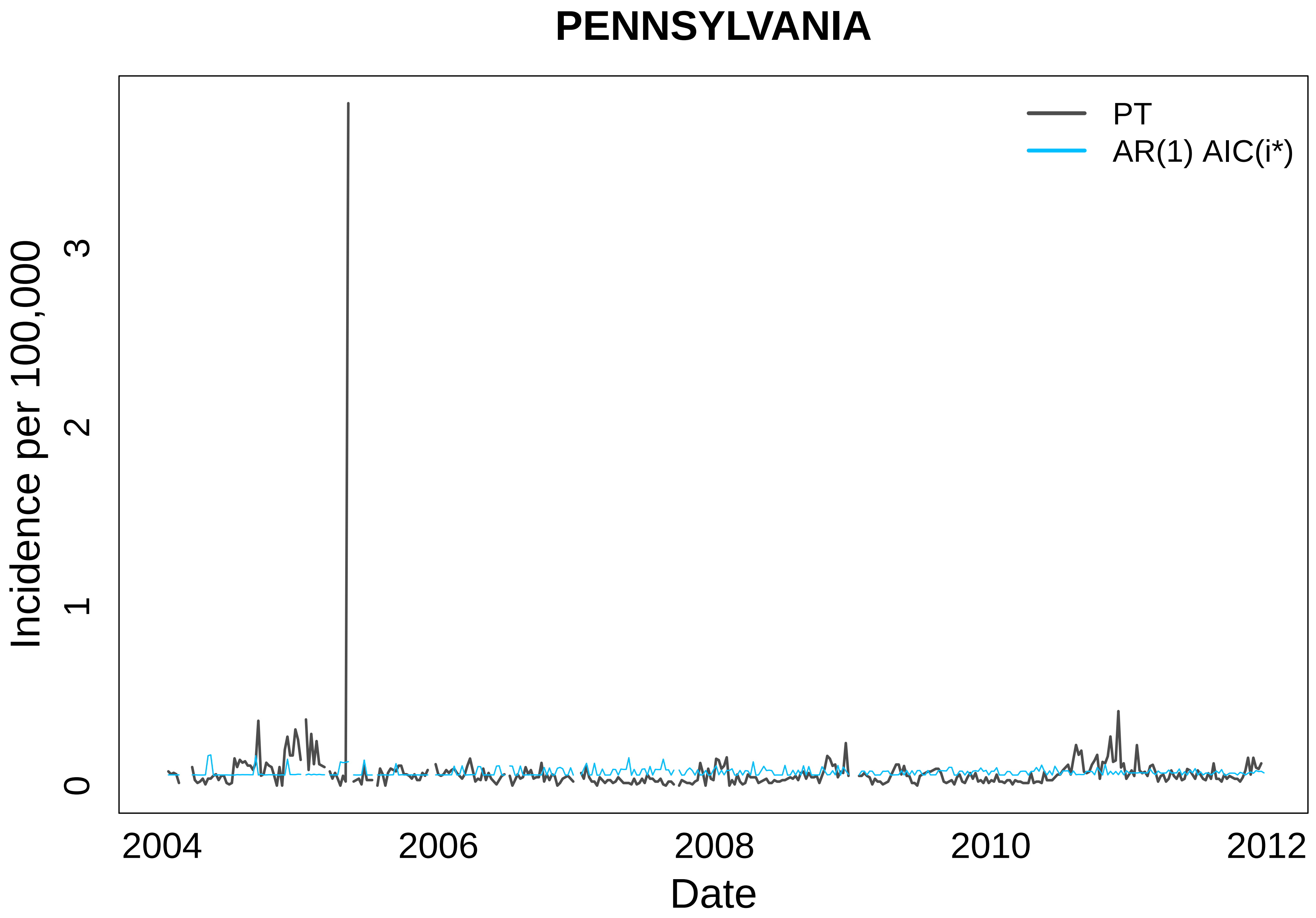

PENNSYLVANIA
PT
AR(1) AIC(i*)
Incidence per 100,000
0
1
2
3
2004
2006
2008
2010
2012
Date

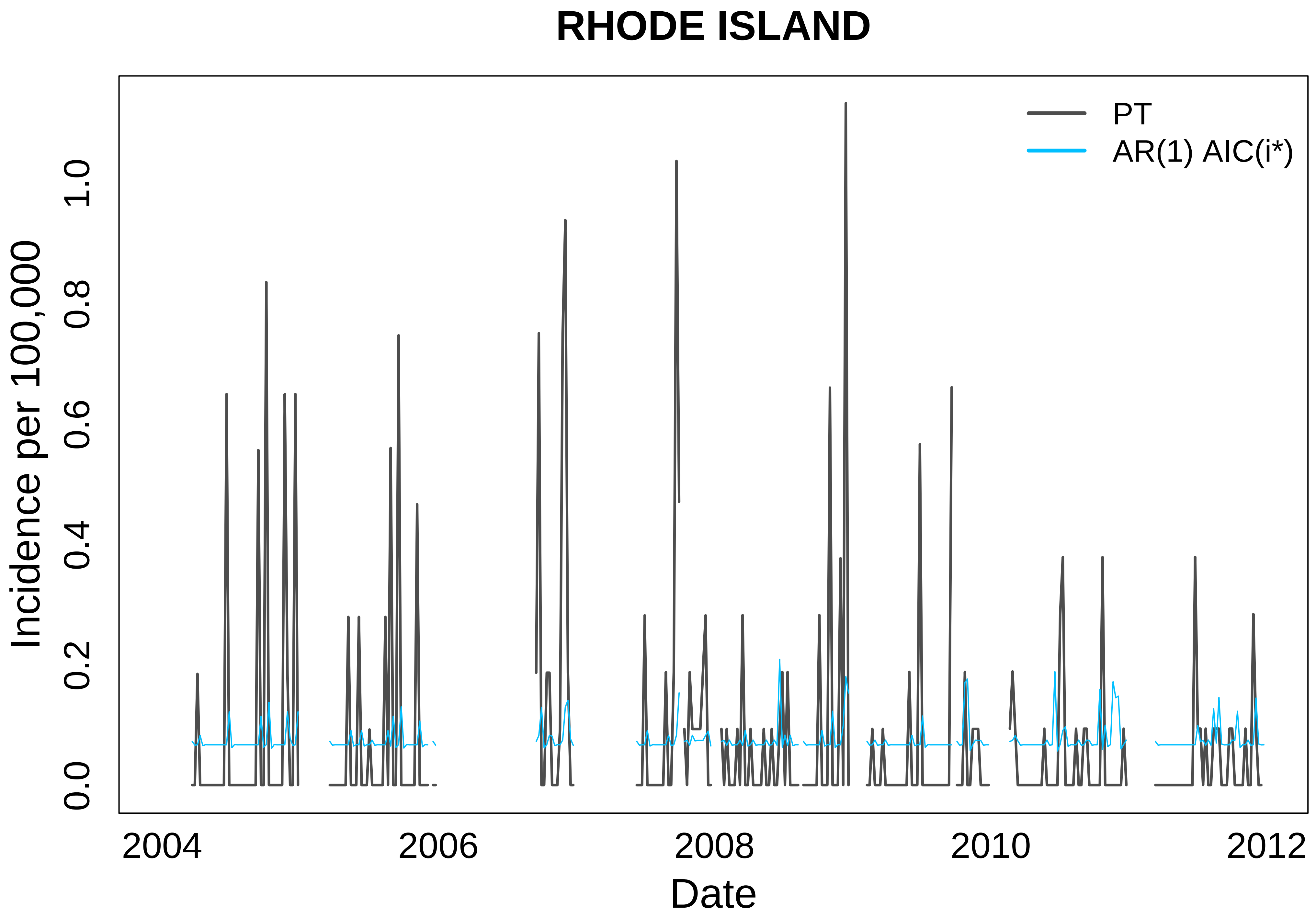
RHODE ISLAND
PT
AR(1) AIC(i*)
Incidence per 100,000
0.0
0.2
0.4
0.6
0.8
1.0
2004
2006
2008
2010
2012
Date

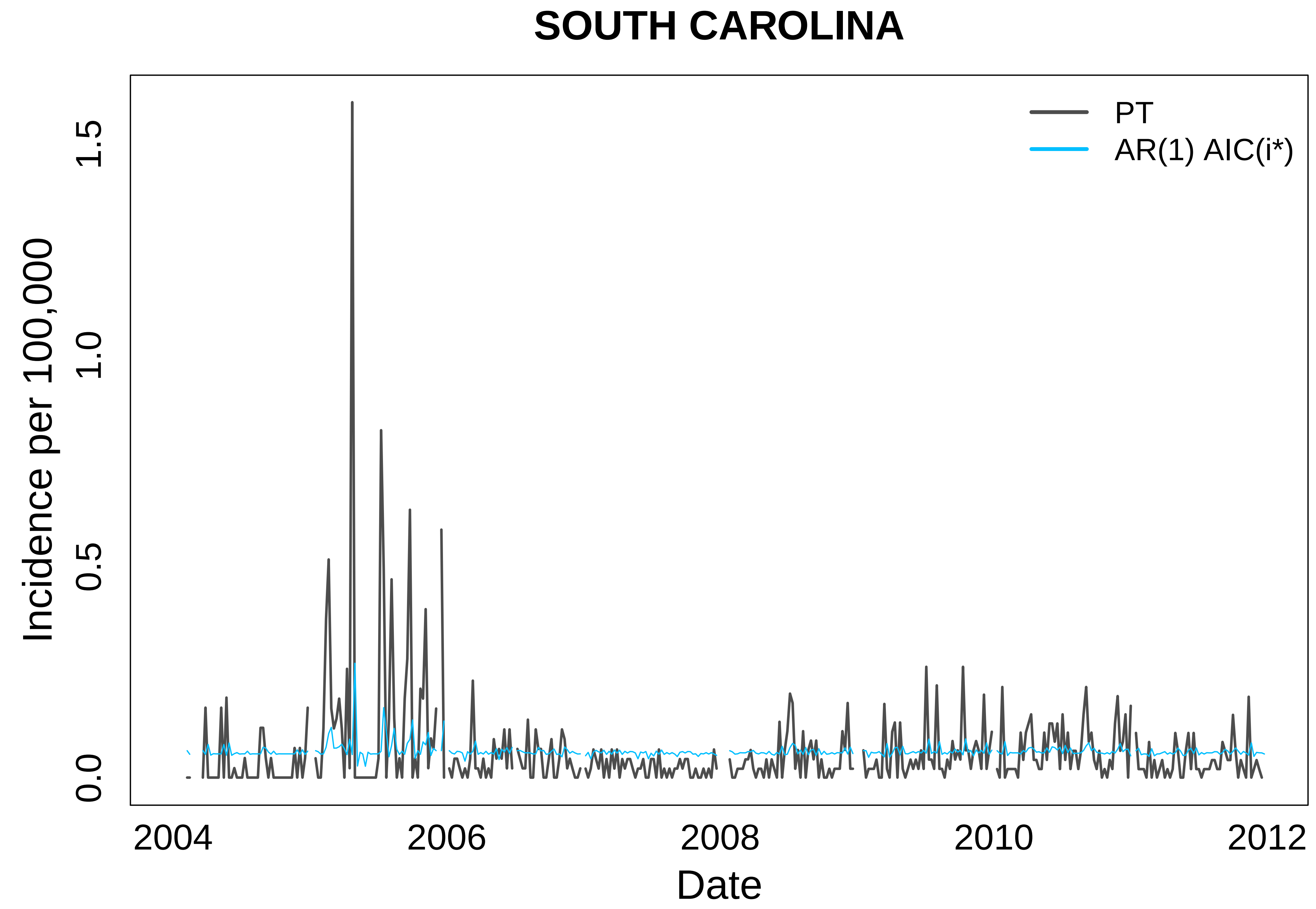

SOUTH CAROLINA
PT
AR(1) AIC(i*)
Incidence per 100,000
0.0
0.5
1.0
1.5
2004
2006
2008
2010
2012
Date

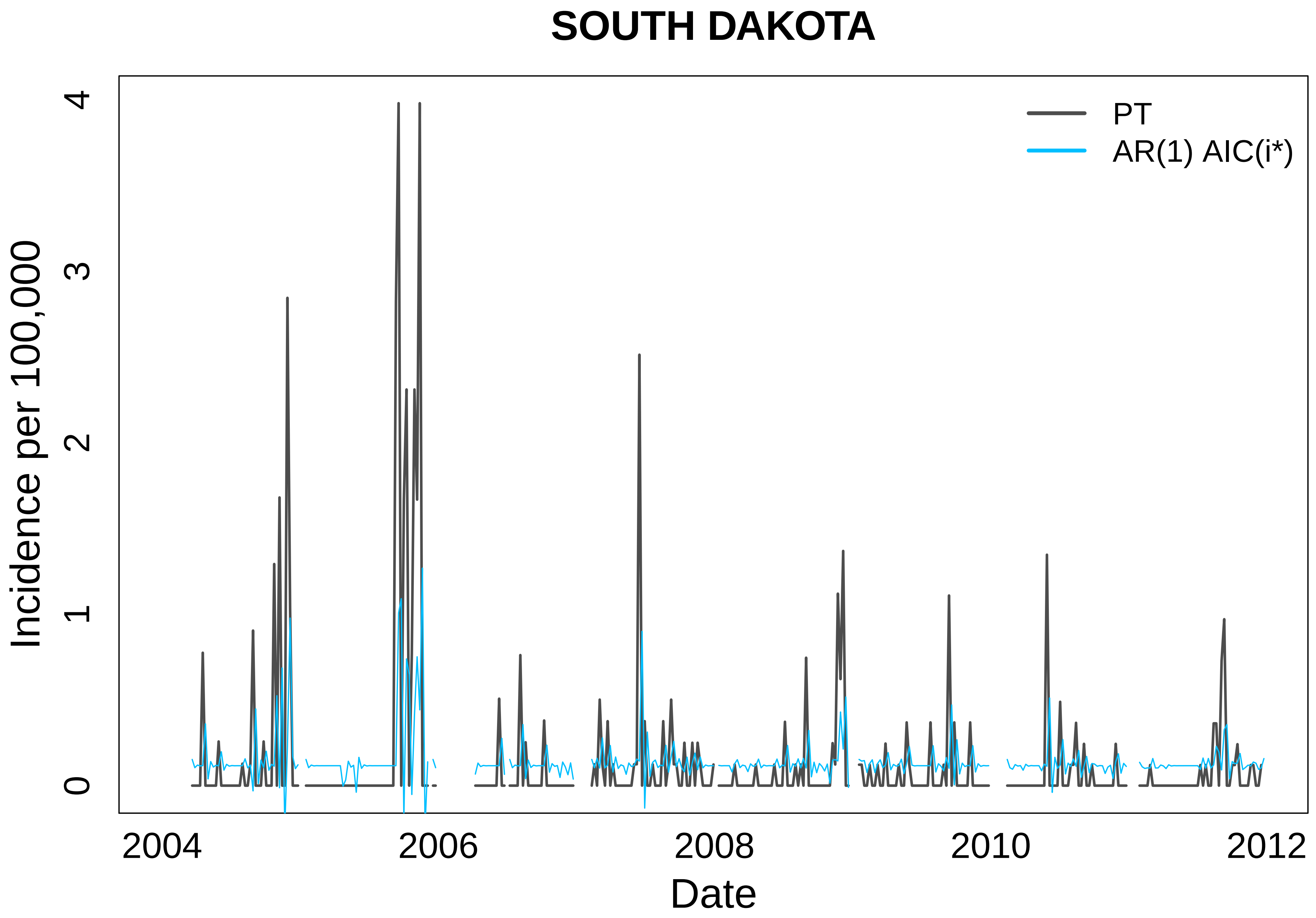

SOUTH DAKOTA
PT
AR(1) AIC(i*)
Incidence per 100,000
0
1
2
3
4
2004
2006
2008
2010
2012
Date

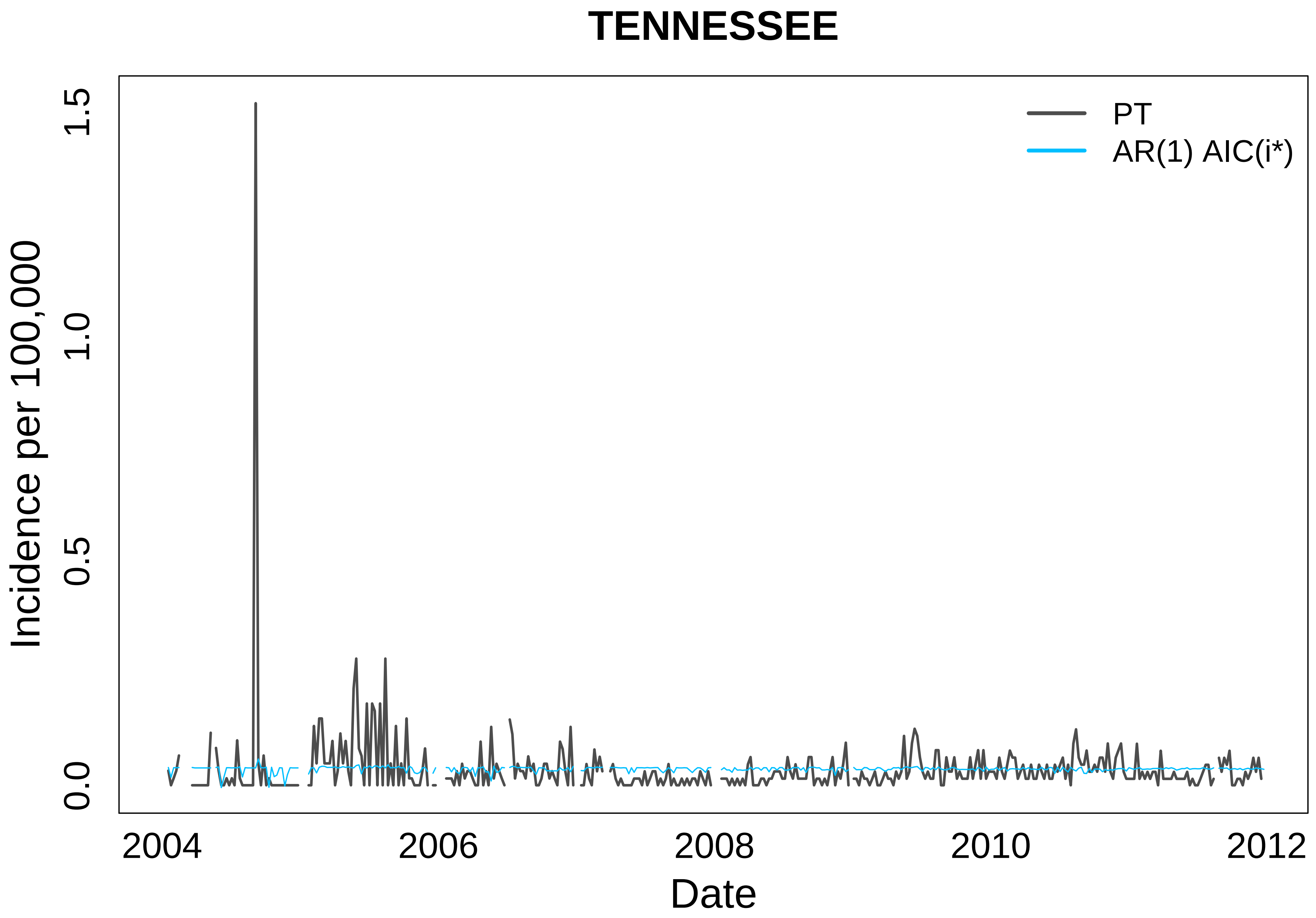
TENNESSEE
PT
AR(1) AIC(i*)
Incidence per 100,000
0.0
0.5
1.0
1.5
2004
2006
2008
2010
2012
Date

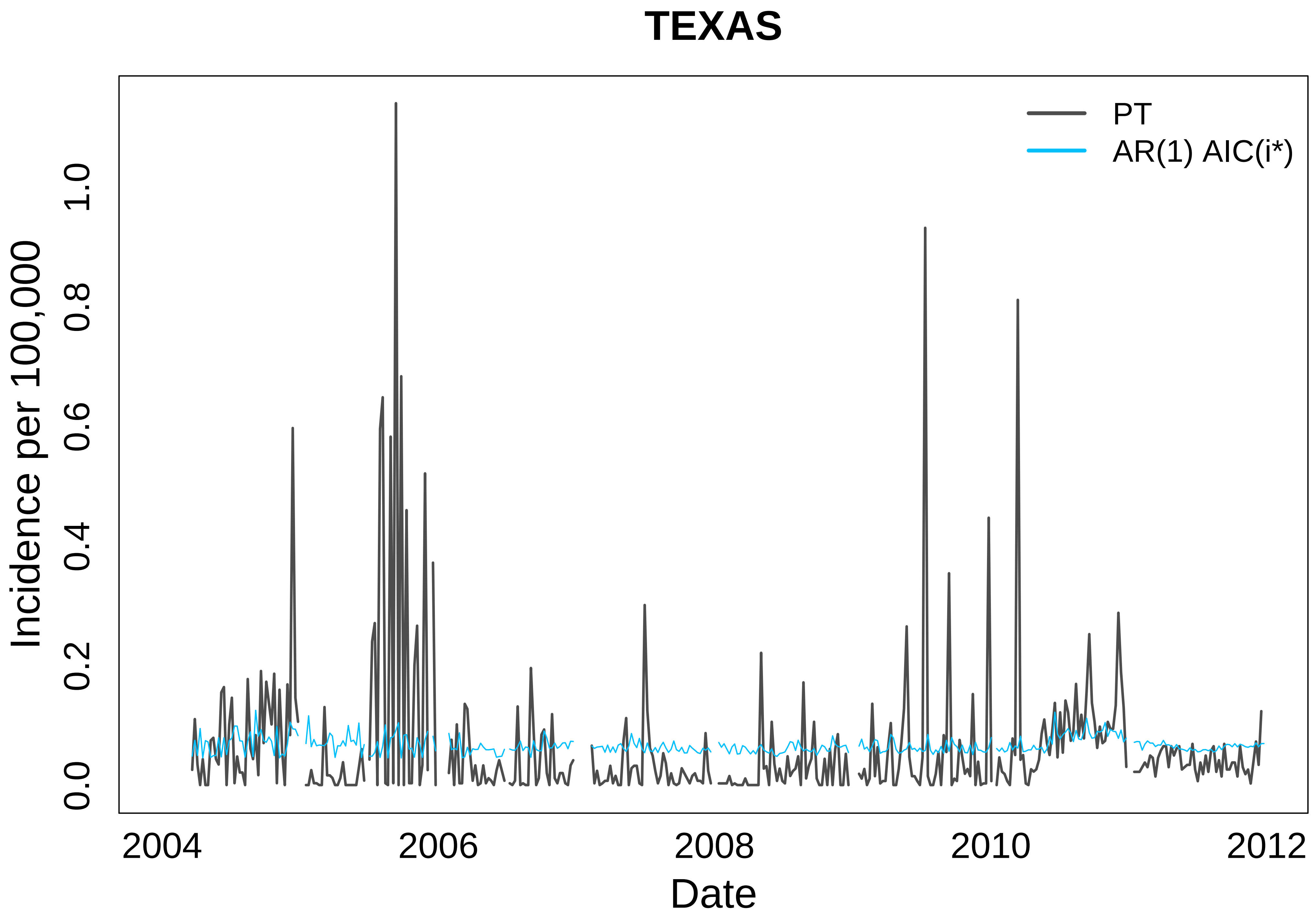
TEXAS
PT
AR(1) AIC(i*)
Incidence per 100,000
0.0
0.2
0.4
0.6
0.8
1.0
2004
2006
2008
2010
2012
Date

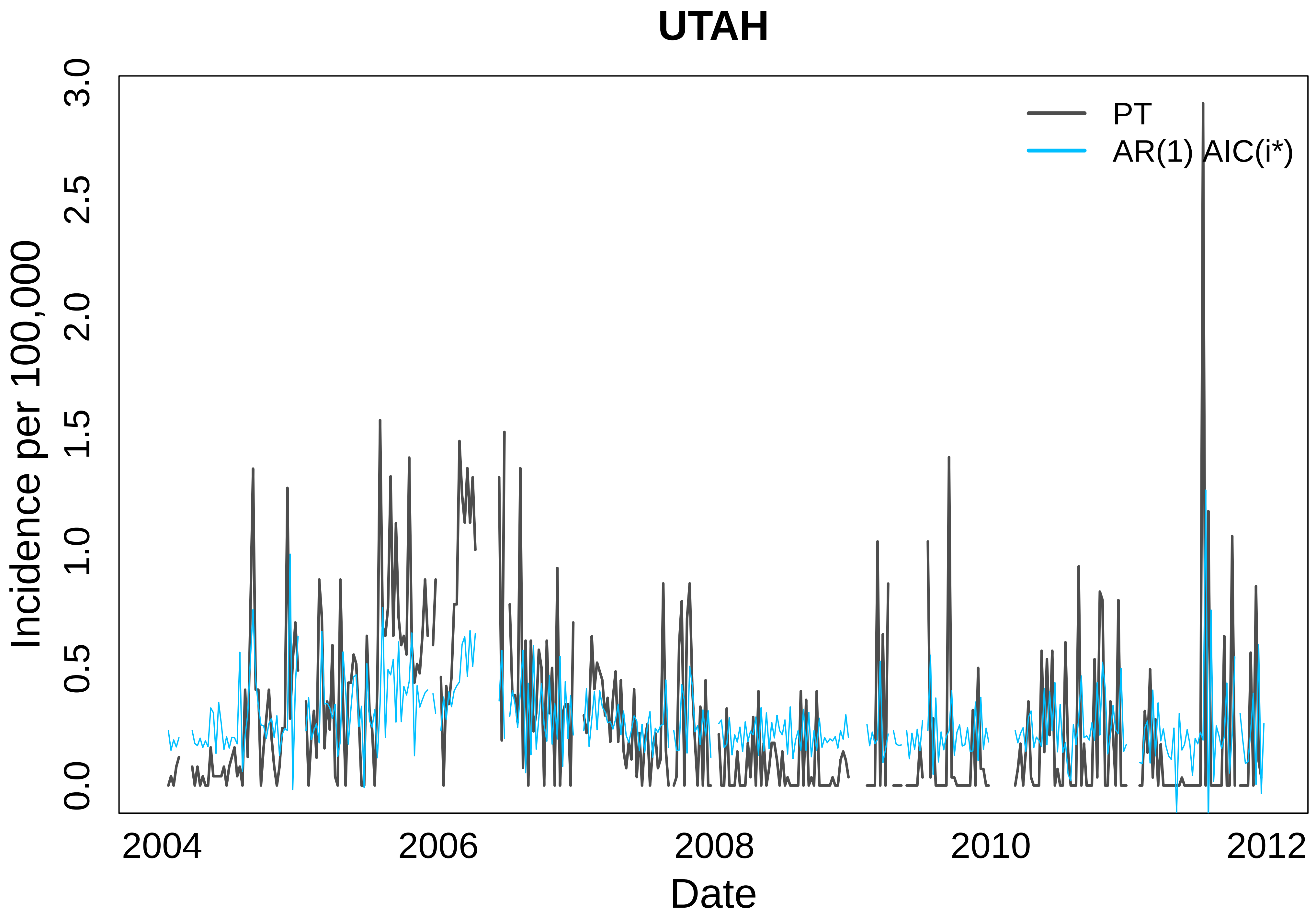
UTAH
PT
AR(1) AIC(i*)
Incidence per 100,000
0.0
0.5
1.0
1.5
2.0
2.5
3.0
2004
2006
2008
2010
2012
Date

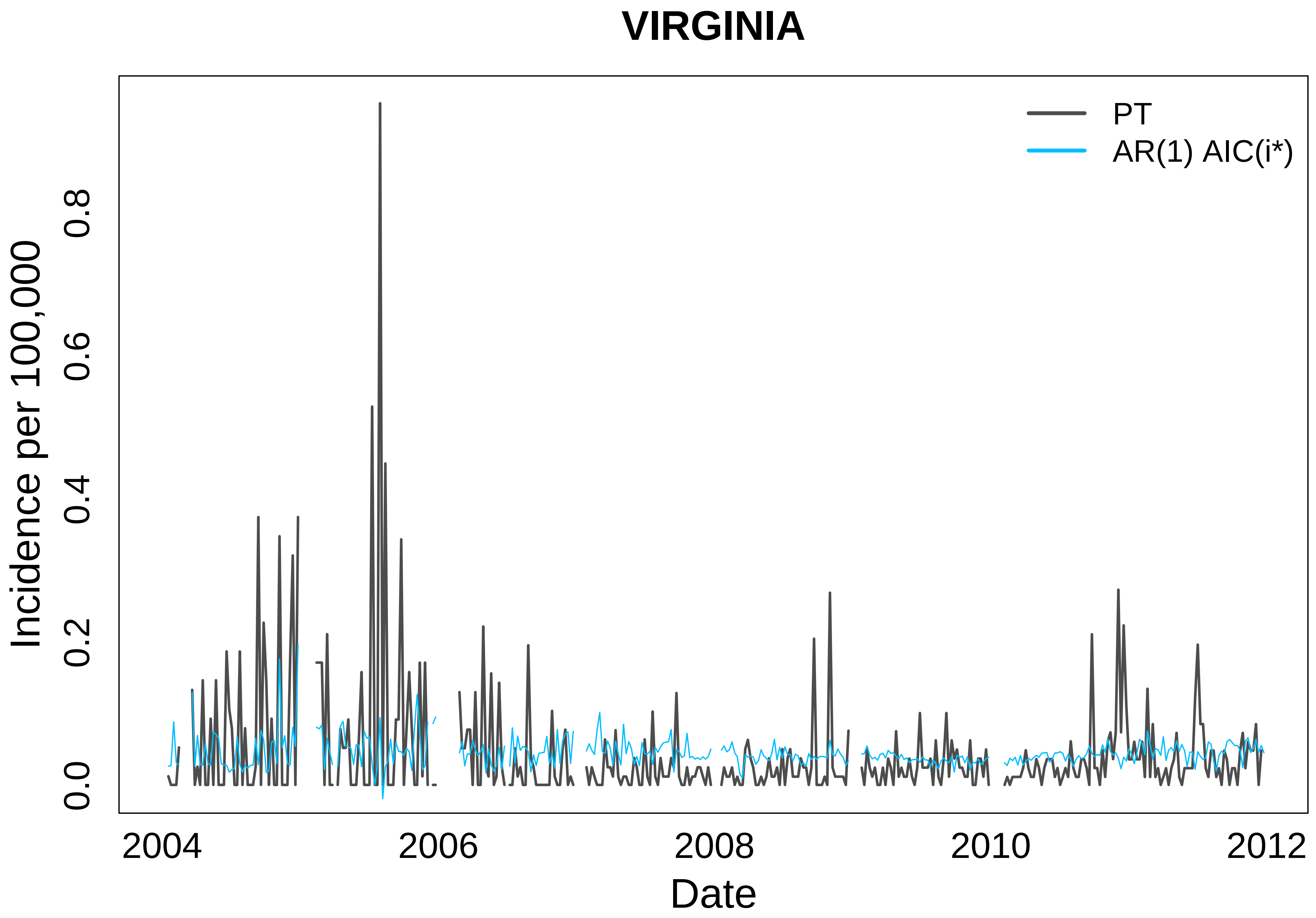

VIRGINIA
PT
AR(1) AIC(i*)
Incidence per 100,000
0.0
0.2
0.4
0.6
0.8
2004
2006
2008
2010
2012
Date

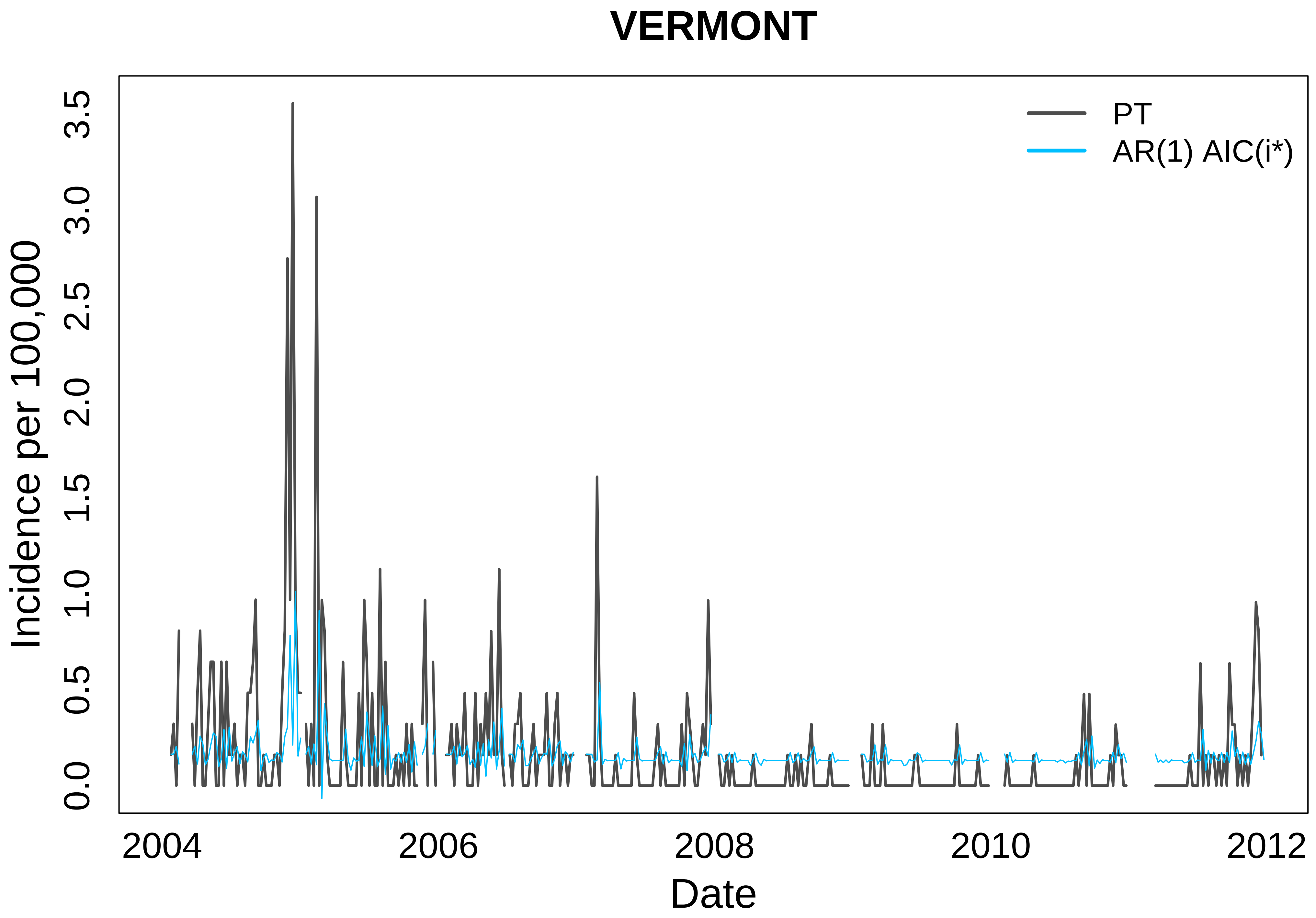
VERMONT
PT
AR(1) AIC(i*)
Incidence per 100,000
0.0
0.5
1.0
1.5
2.0
2.5
3.0
3.5
2004
2006
2008
2010
2012
Date

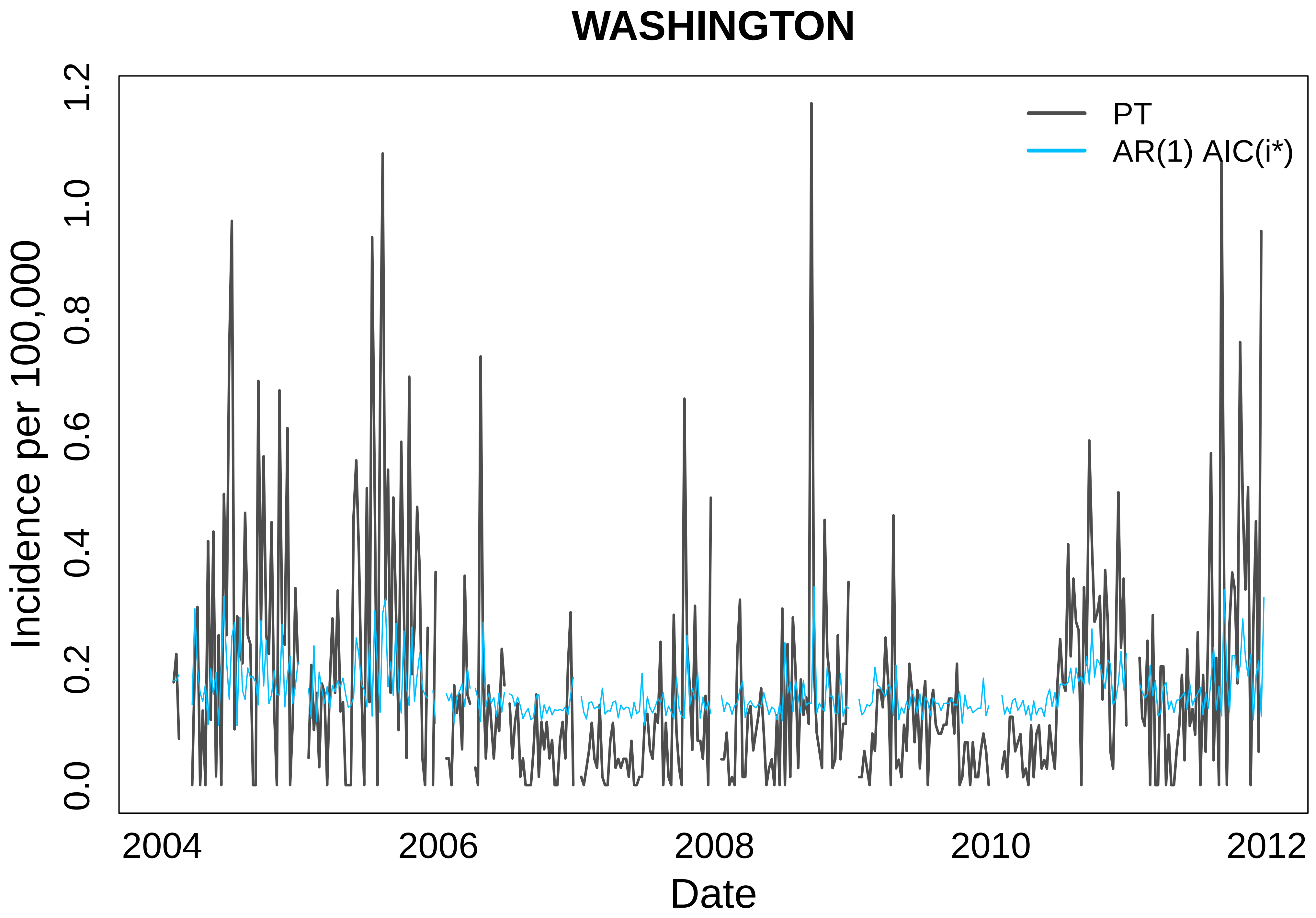
WASHINGTON
Incidence per 100,000
0.0
0.2
0.4
0.6
0.8
1.0
1.2
PT
AR(1) AIC(i*)
2004
2006
2008
2010
2012
Date

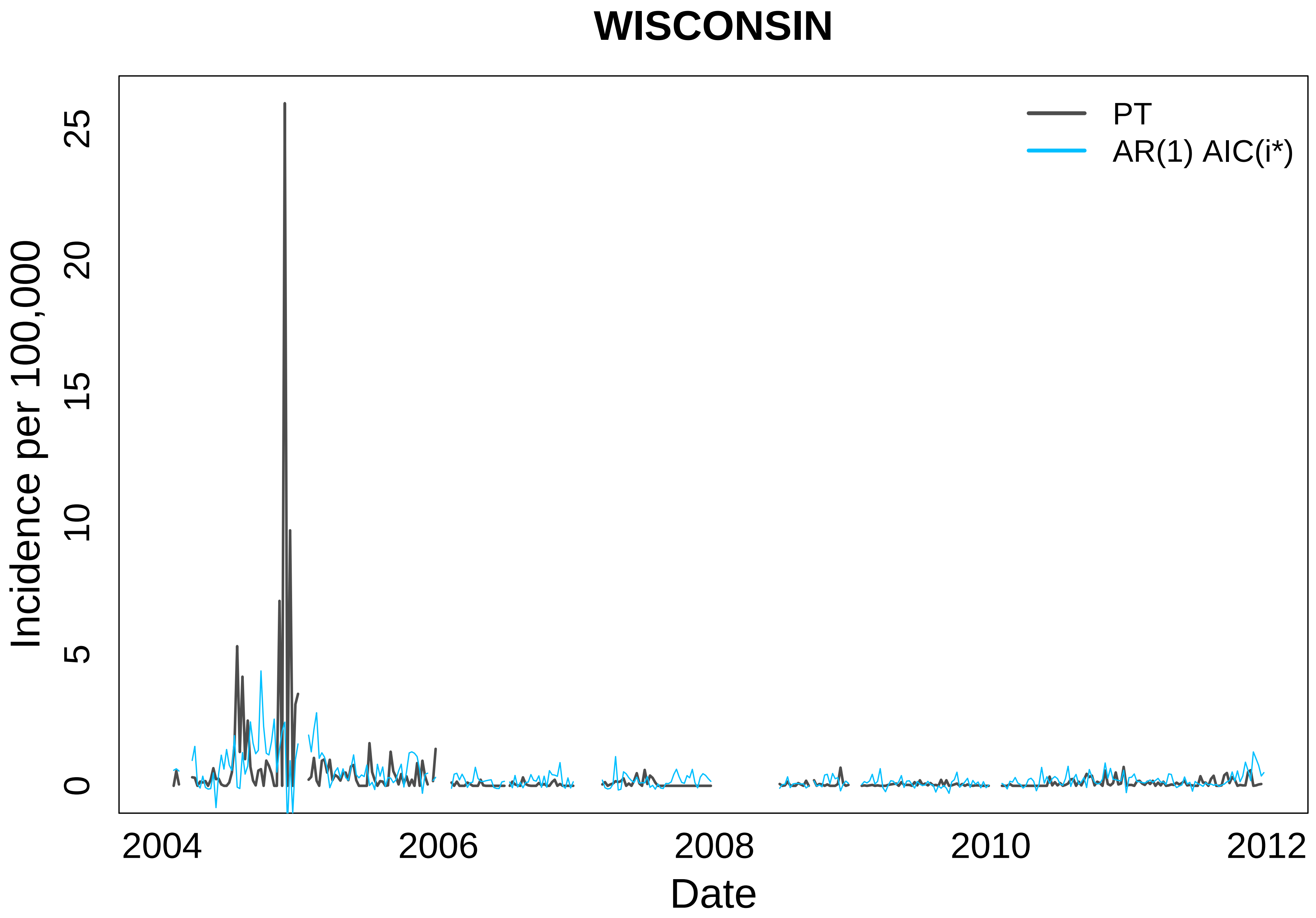

WISCONSIN
PT
AR(1) AIC(i*)
Incidence per 100,000
0
5
10
15
20
25
2004
2006
2008
2010
2012
Date

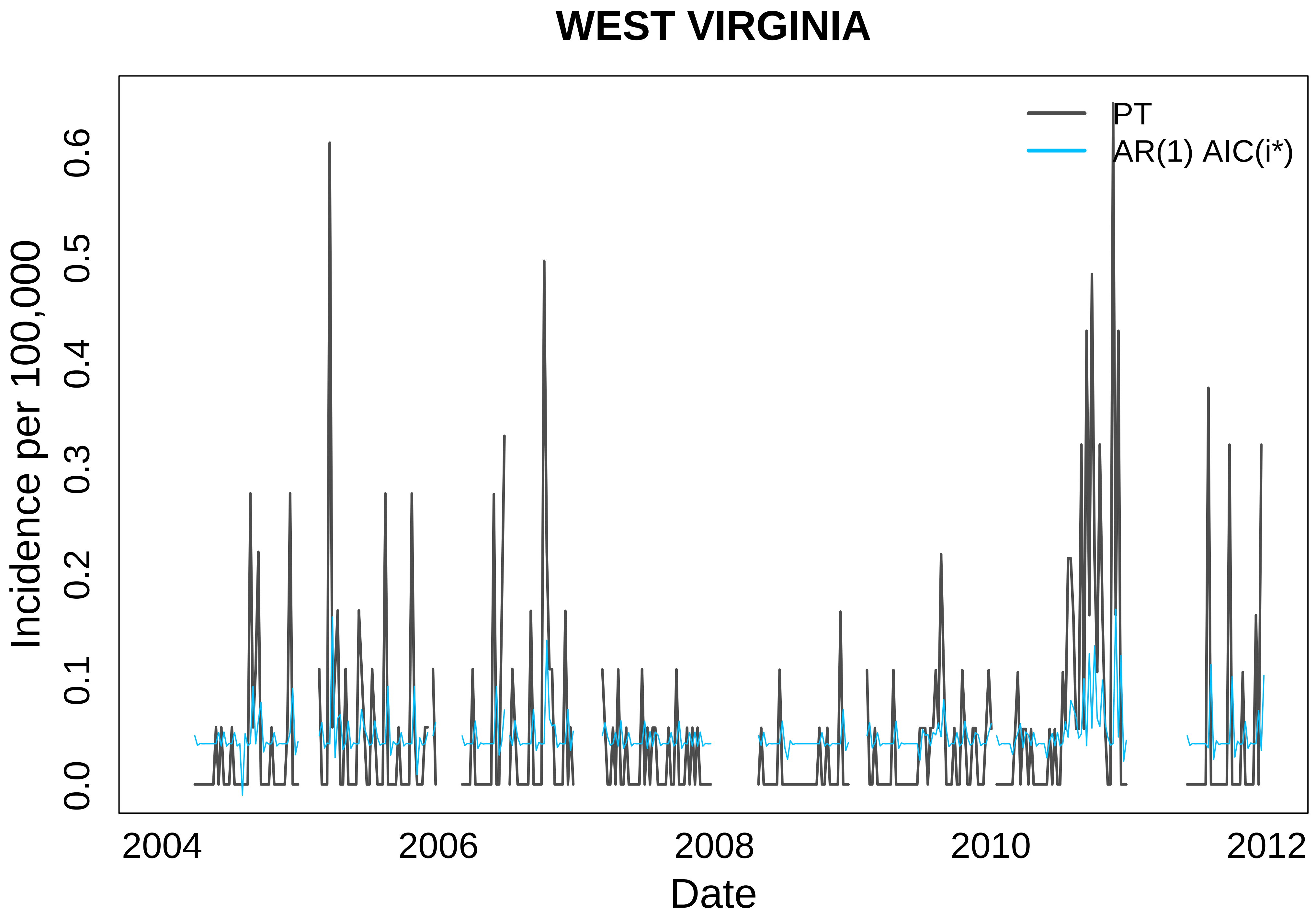
WEST VIRGINIA
PT
AR(1) AIC(i*)
Incidence per 100,000
0.0
0.1
0.2
0.3
0.4
0.5
0.6
2004
2006
2008
2010
2012
Date

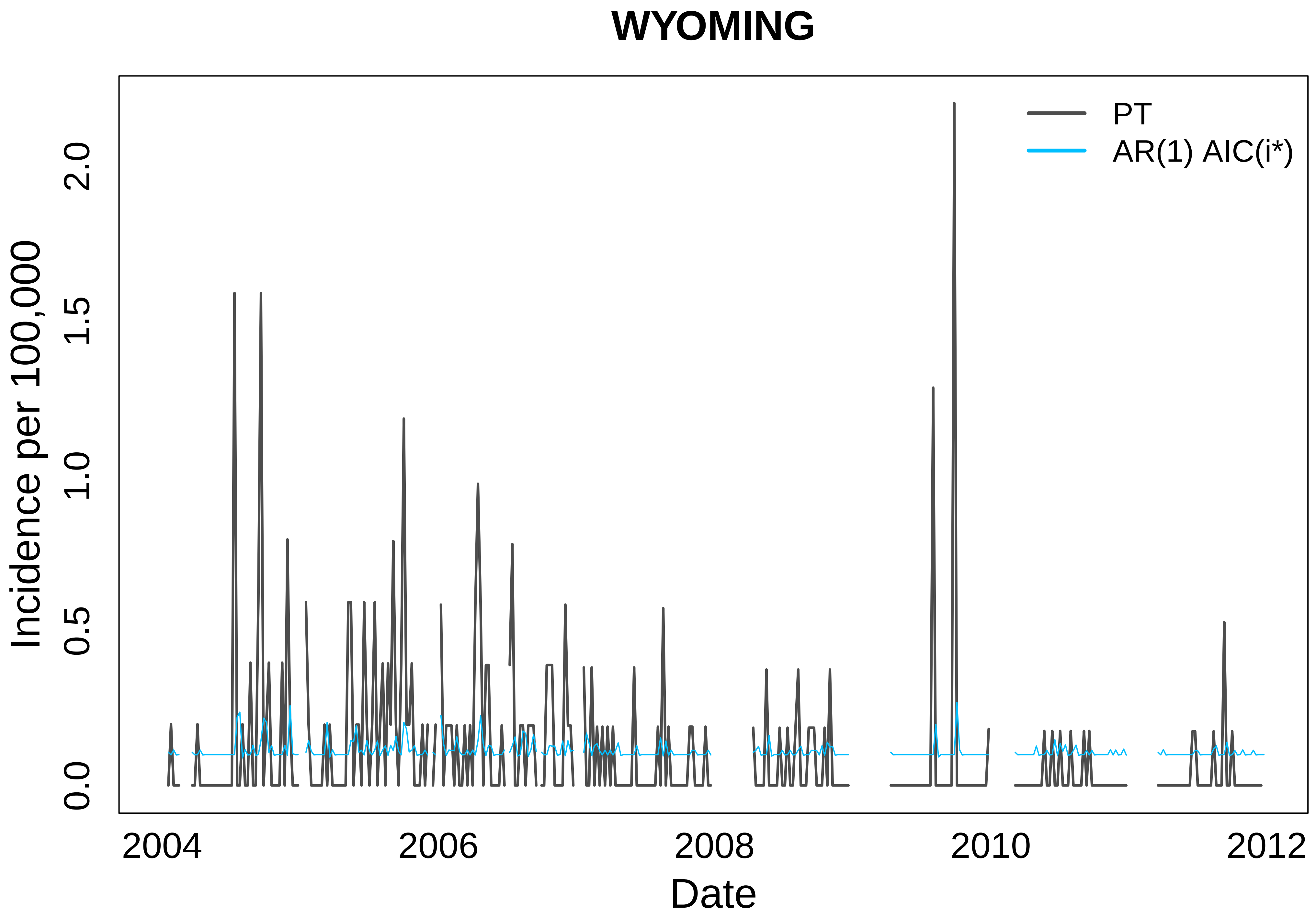

WYOMING
PT
AR(1) AIC(i*)
Incidence per 100,000
0.0
0.5
1.0
1.5
2.0
2004
2006
2008
2010
2012
Date

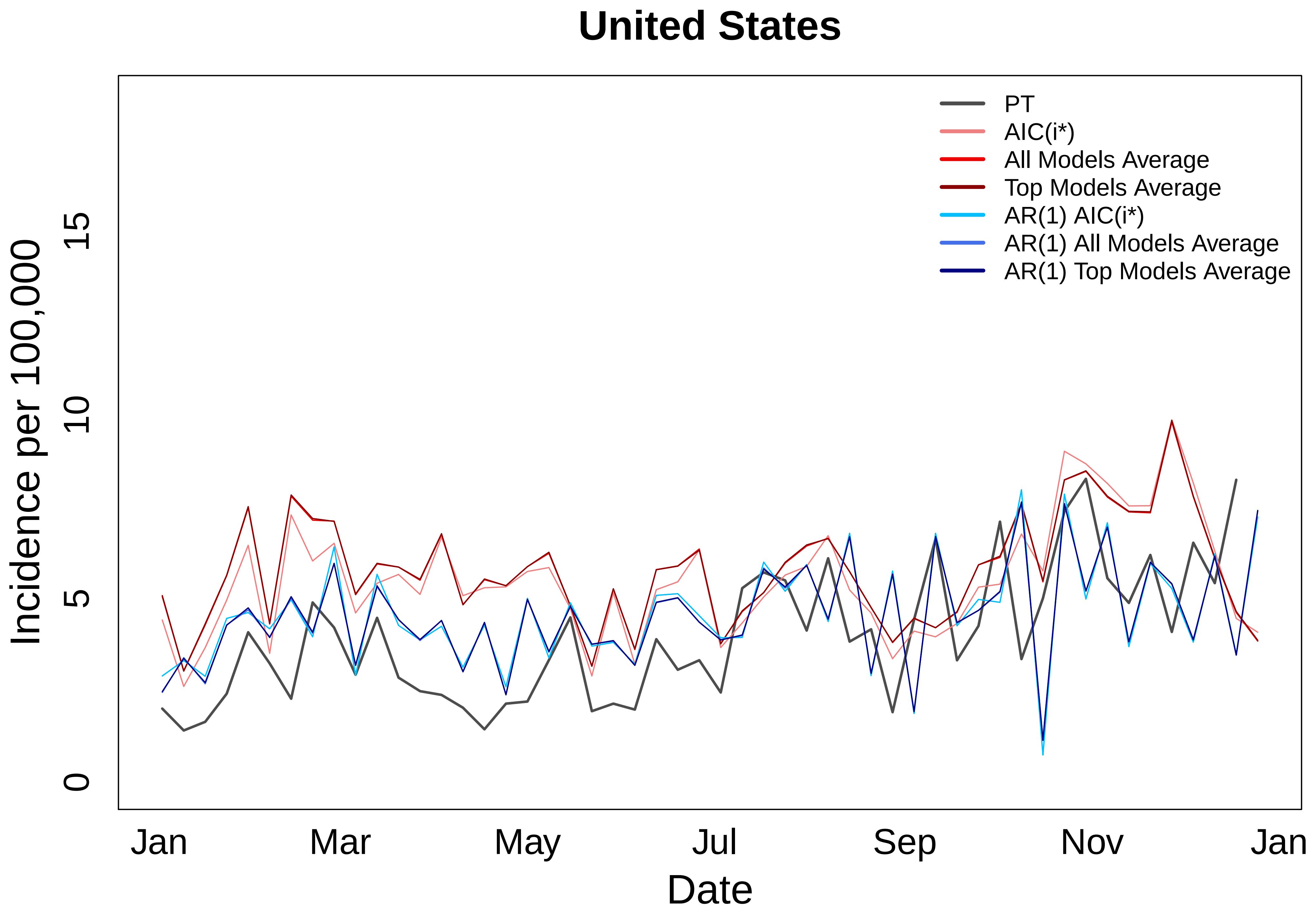
United States
PT
AIC(i*)
All Models Average
Top Models Average
AR(1) AIC(i*)
AR(1) All Models Average
AR(1) Top Models Average
Incidence per 100,000
0
5
10
15
Jan
Mar
May
Jul
Sep
Nov
Jan
Date

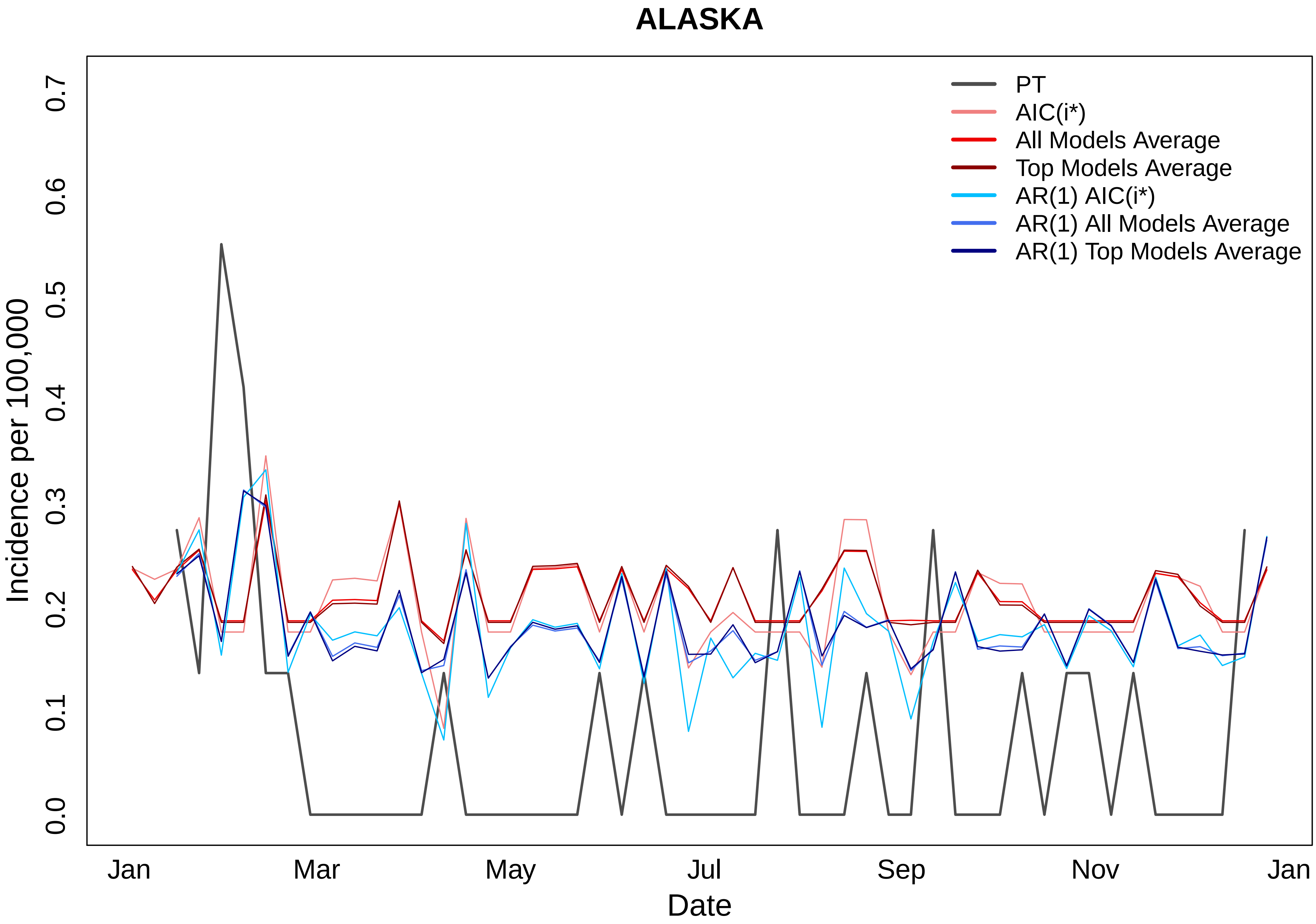
ALASKA
PT
AIC(i*)
All Models Average
Top Models Average
AR(1) AIC(i*)
AR(1) All Models Average
AR(1) Top Models Average
Incidence per 100,000
0.0
0.1
0.2
0.3
0.4
0.5
0.6
0.7
Jan
Mar
May
Jul
Sep
Nov
Jan
Date

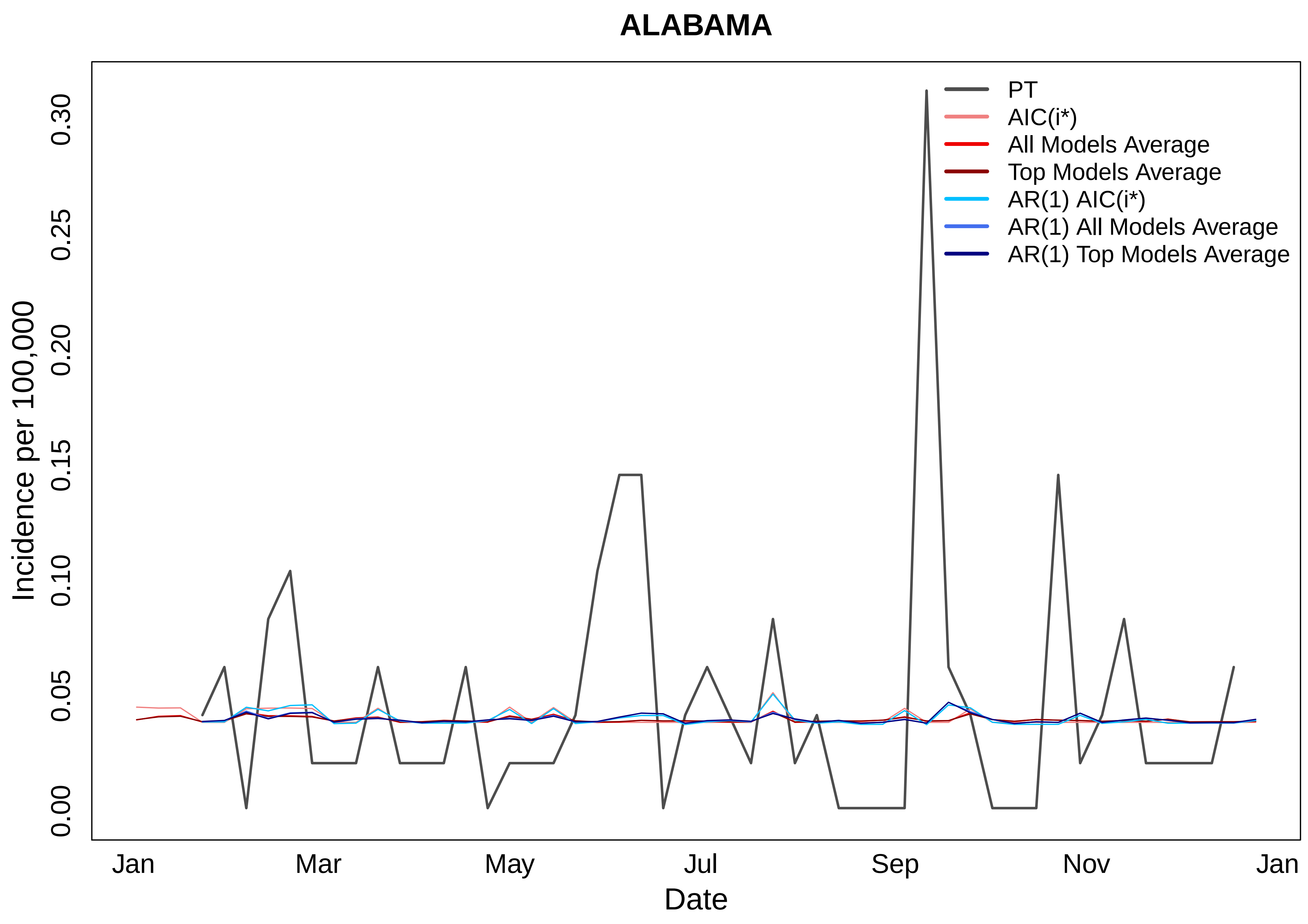
ALABAMA
PT
AIC(i*)
All Models Average
Top Models Average
AR(1) AIC(i*)
AR(1) All Models Average
AR(1) Top Models Average
Incidence per 100,000
0.00
0.05
0.10
0.15
0.20
0.25
0.30
Jan
Mar
May
Jul
Sep
Nov
Jan
Date

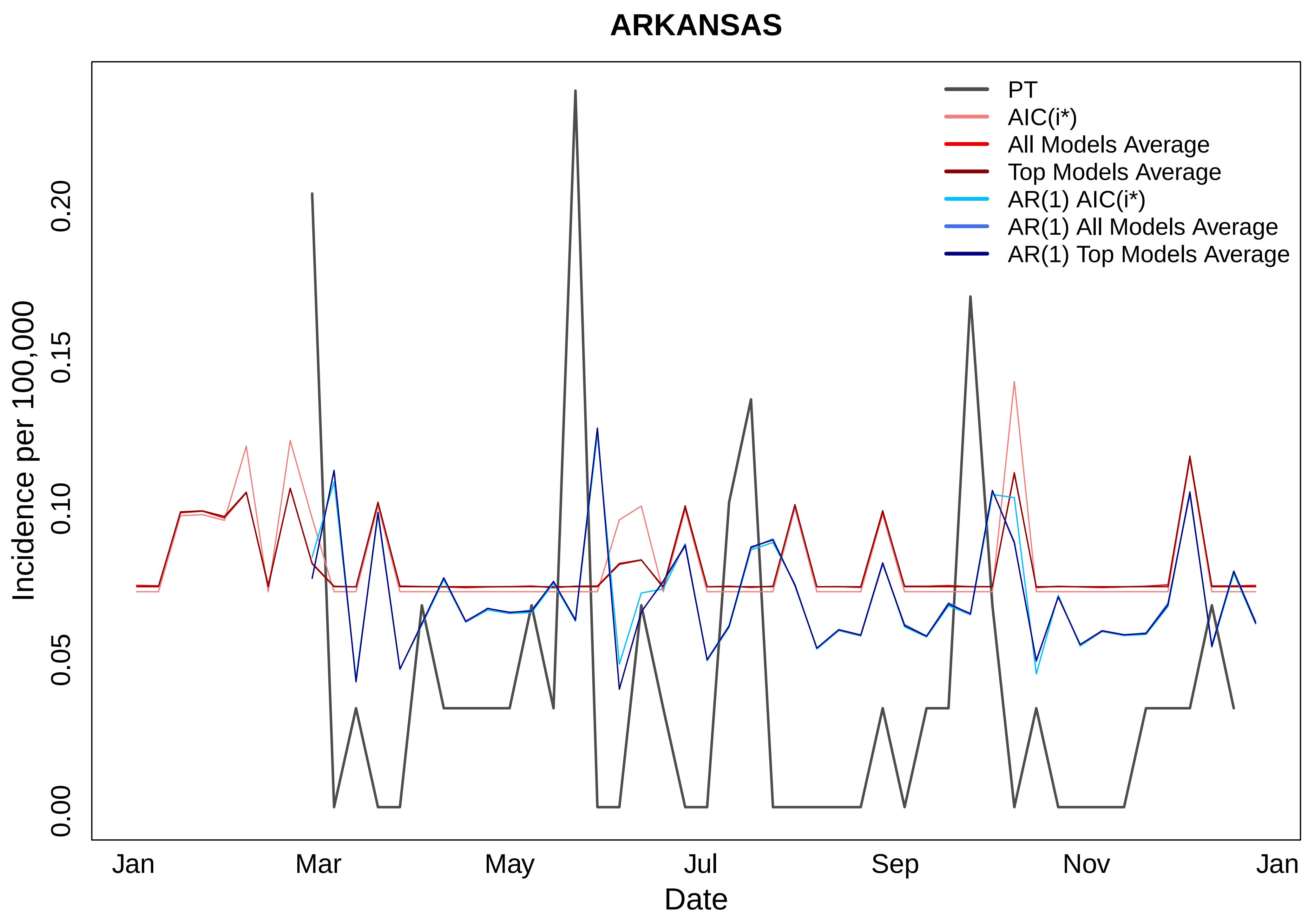

ARKANSAS
PT
AIC(i*)
All Models Average
Top Models Average
AR(1) AIC(i*)
AR(1) All Models Average
AR(1) Top Models Average
Incidence per 100,000
0.00
0.05
0.10
0.15
0.20
Jan
Mar
May
Jul
Sep
Nov
Jan
Date

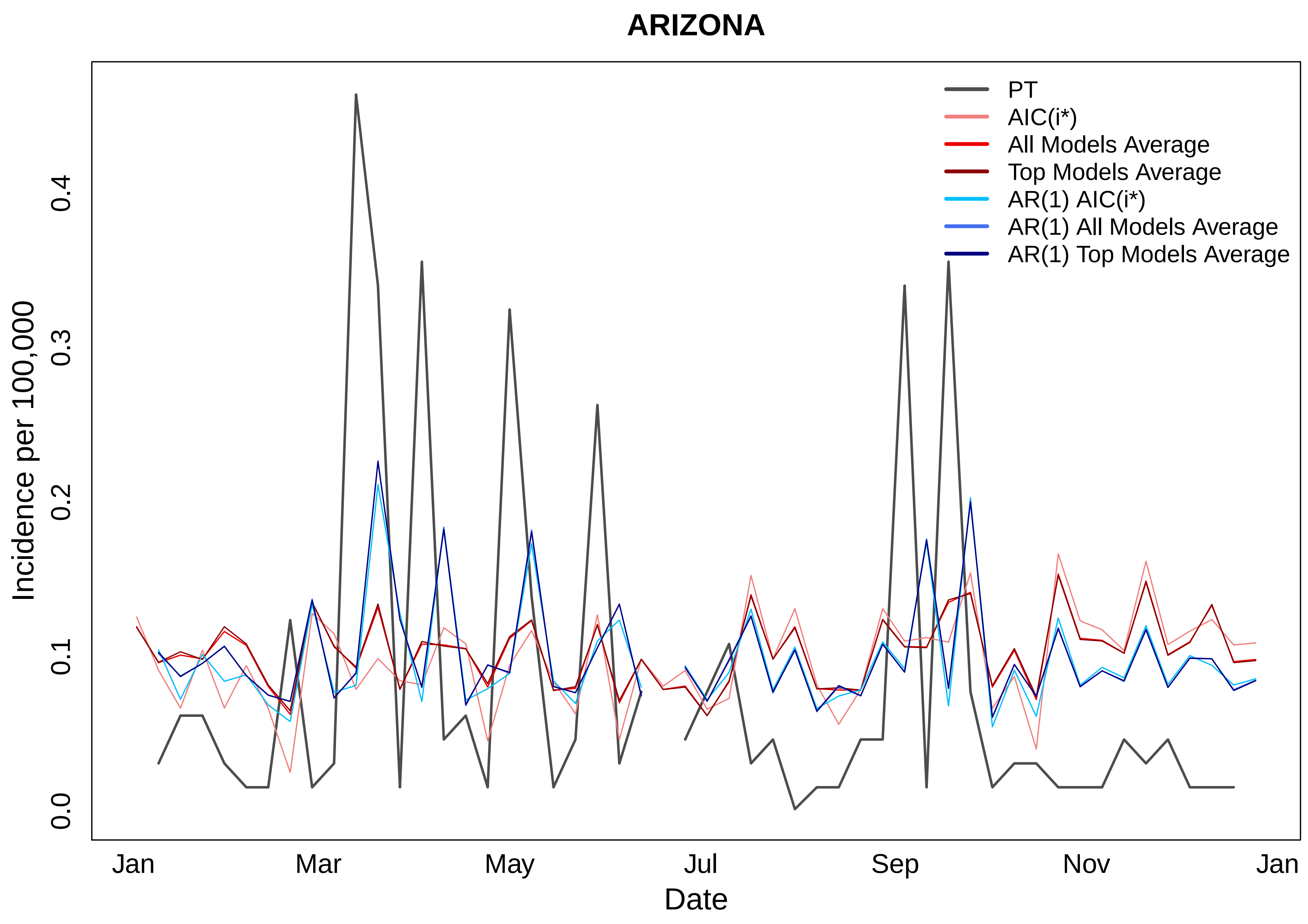

ARIZONA
PT
AIC(i*)
All Models Average
Top Models Average
AR(1) AIC(i*)
AR(1) All Models Average
AR(1) Top Models Average
Incidence per 100,000
0.0
0.1
0.2
0.3
0.4
Jan
Mar
May
Jul
Sep
Nov
Jan
Date

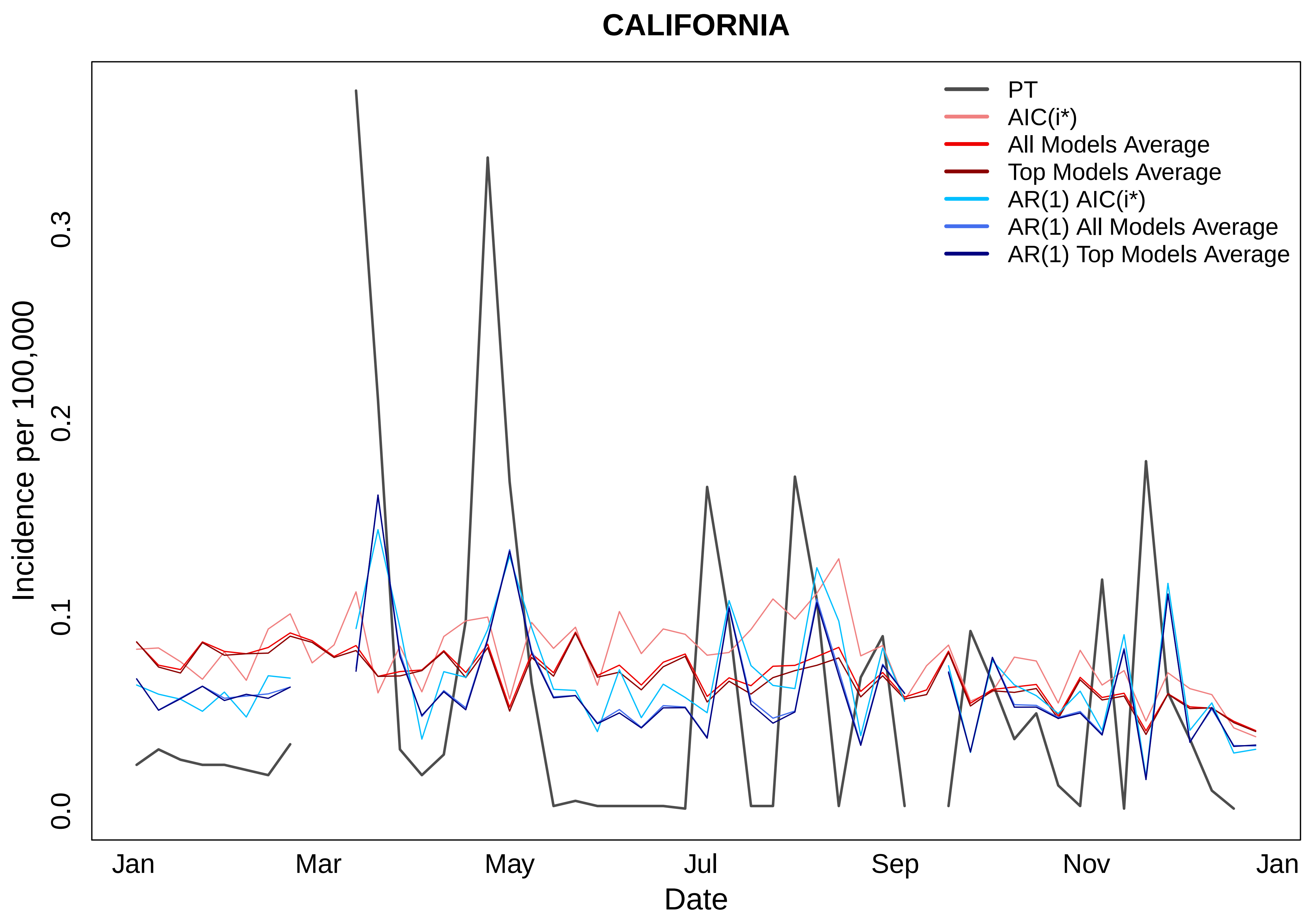

CALIFORNIA
PT
AIC(i*)
All Models Average
Top Models Average
AR(1) AIC(i*)
AR(1) All Models Average
AR(1) Top Models Average
Incidence per 100,000
0.0
0.1
0.2
0.3
Jan
Mar
May
Jul
Sep
Nov
Jan
Date

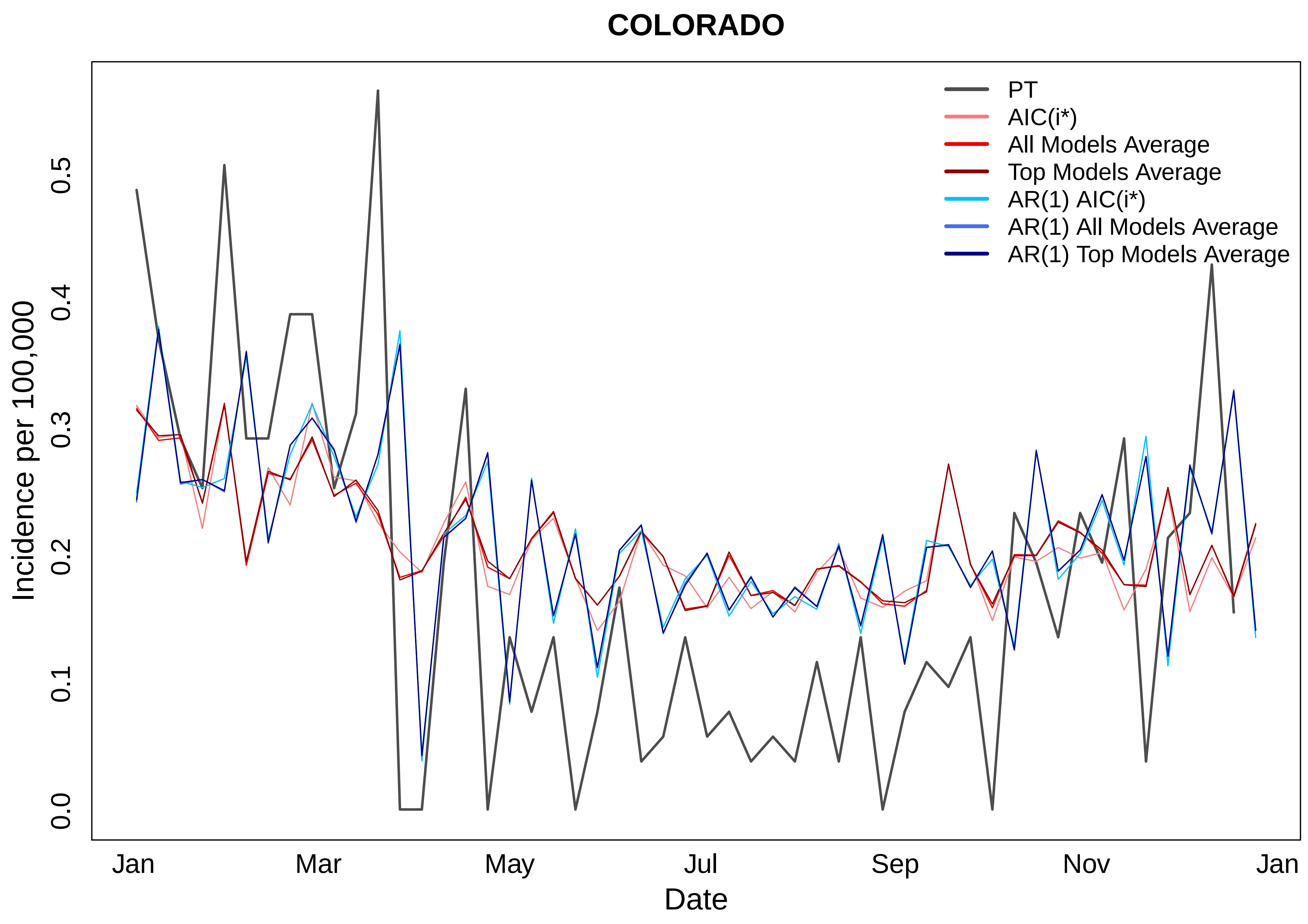
COLORADO
PT
AIC(i*)
All Models Average
Top Models Average
AR(1) AIC(i*)
AR(1) All Models Average
AR(1) Top Models Average
Incidence per 100,000
0.0
0.1
0.2
0.3
0.4
0.5
Jan
Mar
May
Jul
Sep
Nov
Jan
Date

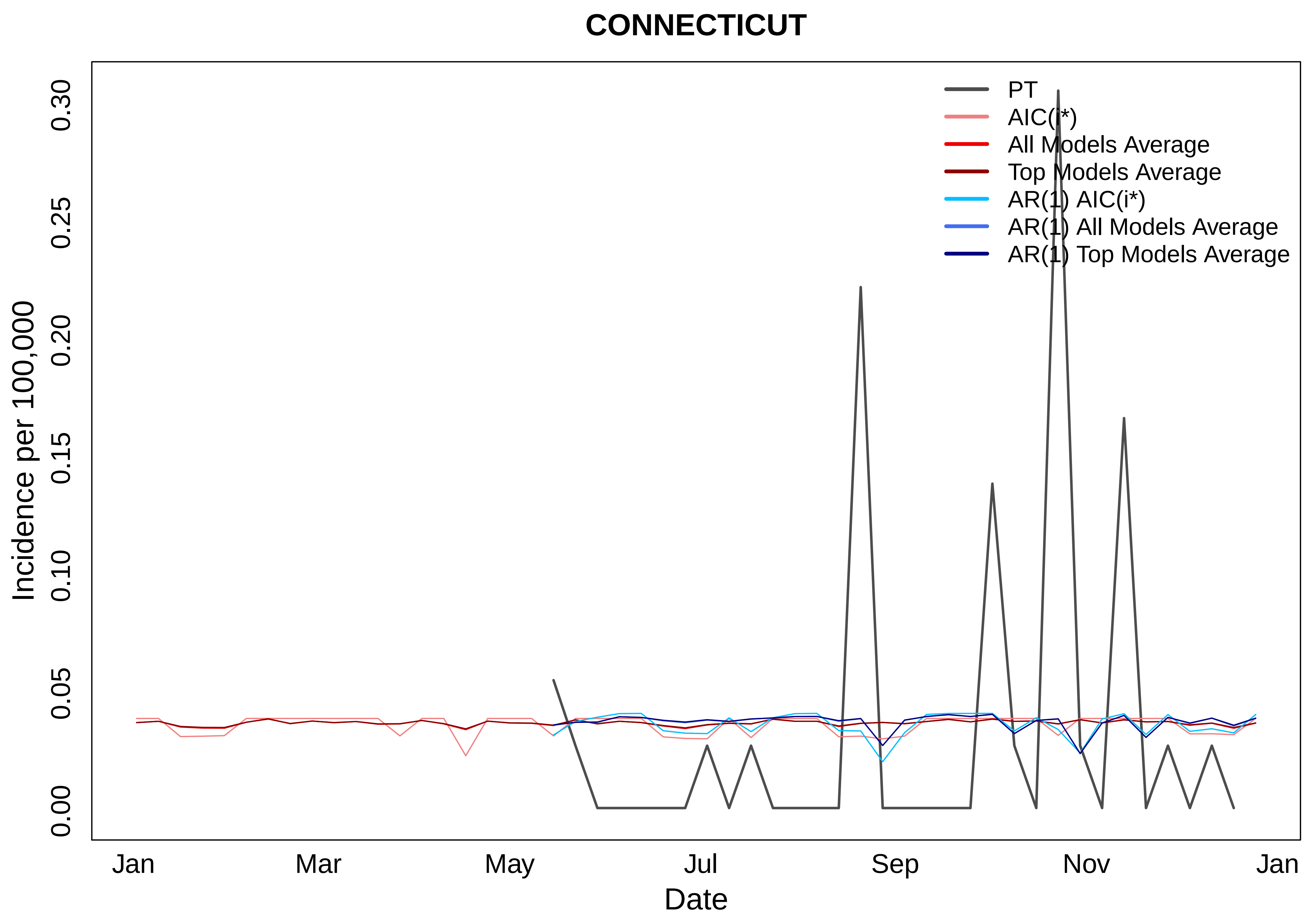

CONNECTICUT
PT
AIC(i*)
All Models Average
Top Models Average
AR(1) AIC(i*)
AR(1) All Models Average
AR(1) Top Models Average
Incidence per 100,000
0.00
0.05
0.10
0.15
0.20
0.25
0.30
Jan
Mar
May
Jul
Sep
Nov
Jan
Date

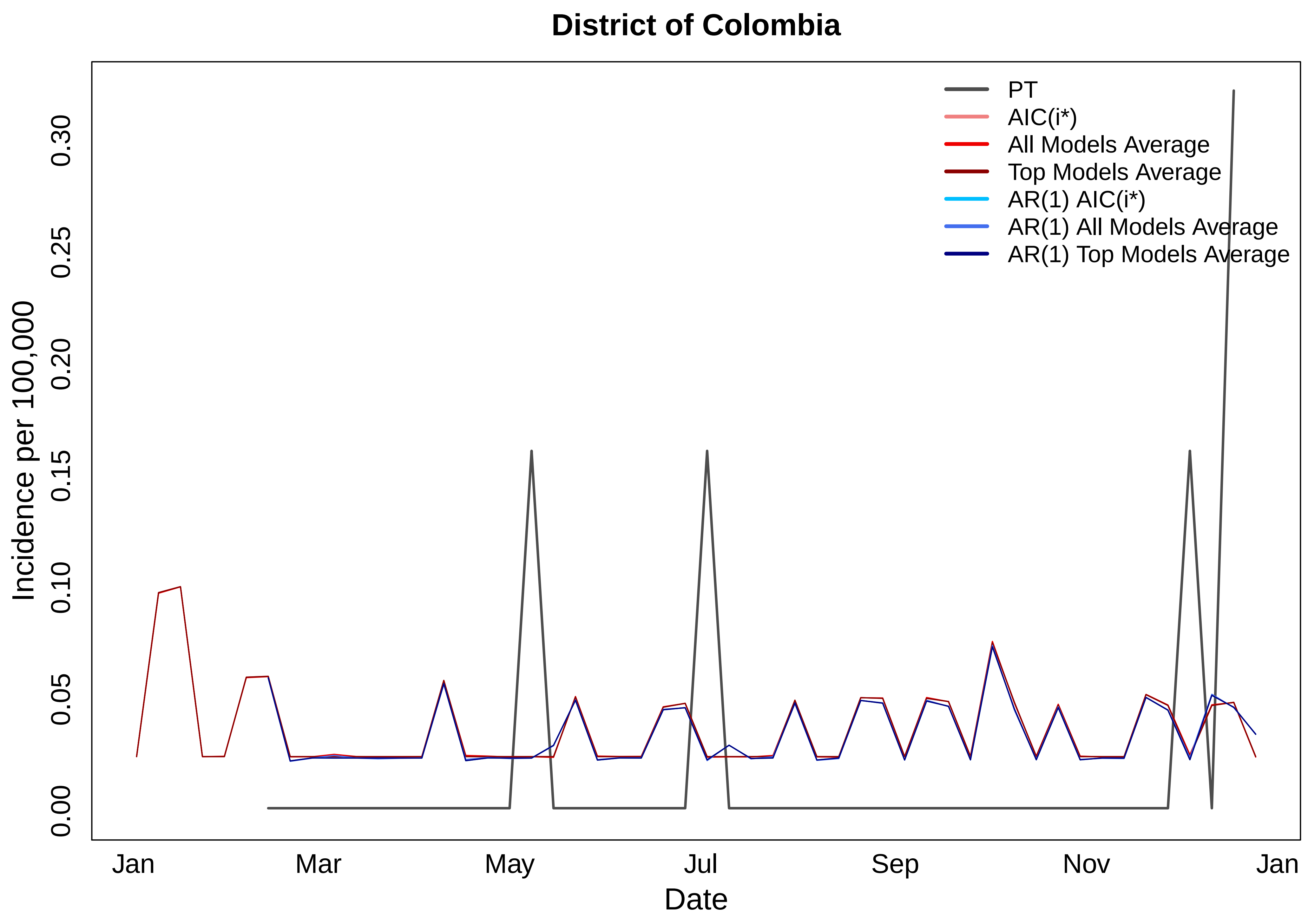
District of Colombia
PT
AIC(i*)
All Models Average
Top Models Average
AR(1) AIC(i*)
AR(1) All Models Average
AR(1) Top Models Average
Incidence per 100,000
0.00
0.05
0.10
0.15
0.20
0.25
0.30
Jan
Mar
May
Jul
Sep
Nov
Jan
Date

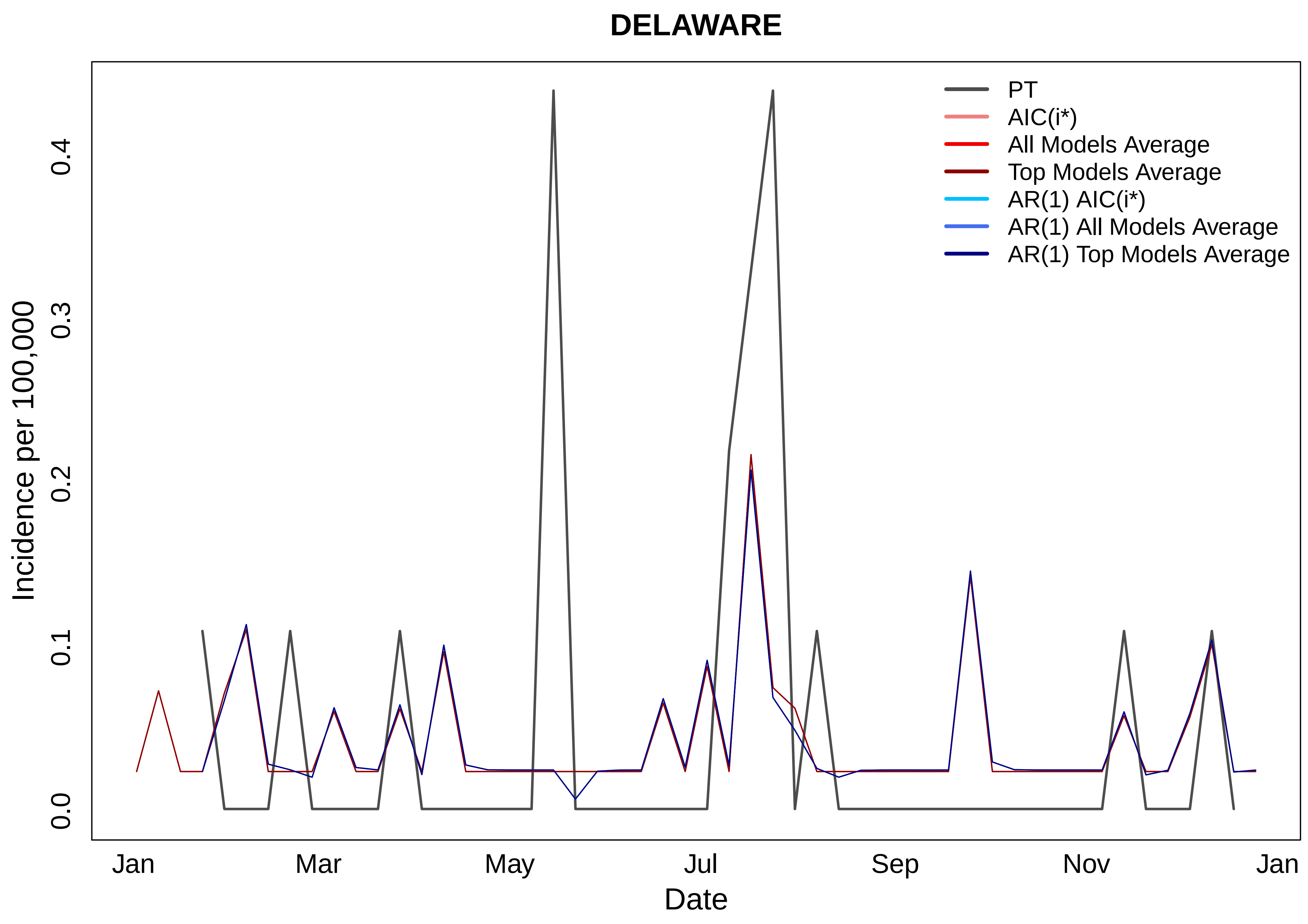
DELAWARE
PT
AIC(i*)
All Models Average
Top Models Average
AR(1) AIC(i*)
AR(1) All Models Average
AR(1) Top Models Average
Incidence per 100,000
0.0
0.1
0.2
0.3
0.4
Jan
Mar
May
Jul
Sep
Nov
Jan
Date

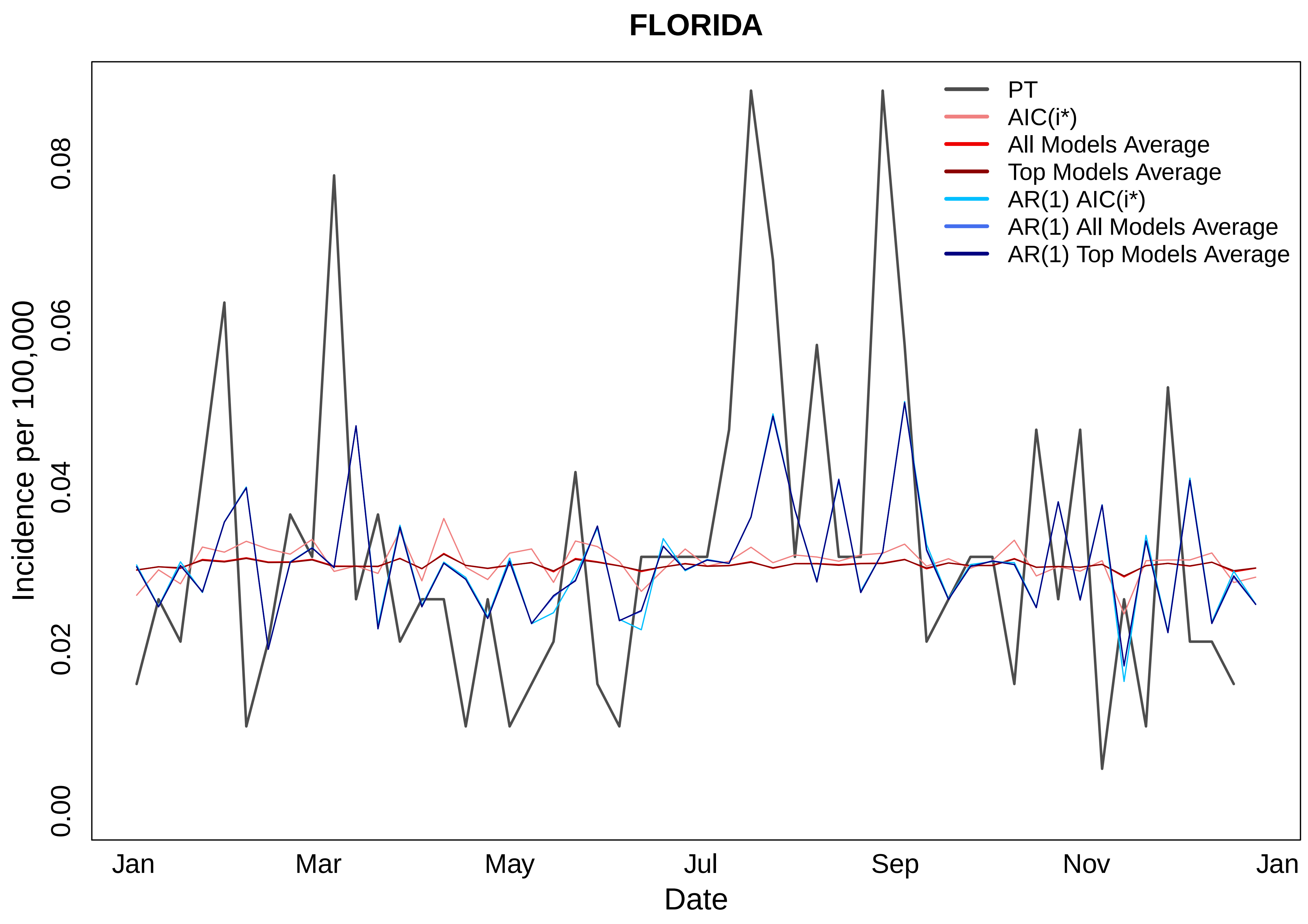

FLORIDA
PT
AIC(i*)
All Models Average
Top Models Average
AR(1) AIC(i*)
AR(1) All Models Average
AR(1) Top Models Average
Incidence per 100,000
0.00
0.02
0.04
0.06
0.08
Jan
Mar
May
Jul
Sep
Nov
Jan
Date

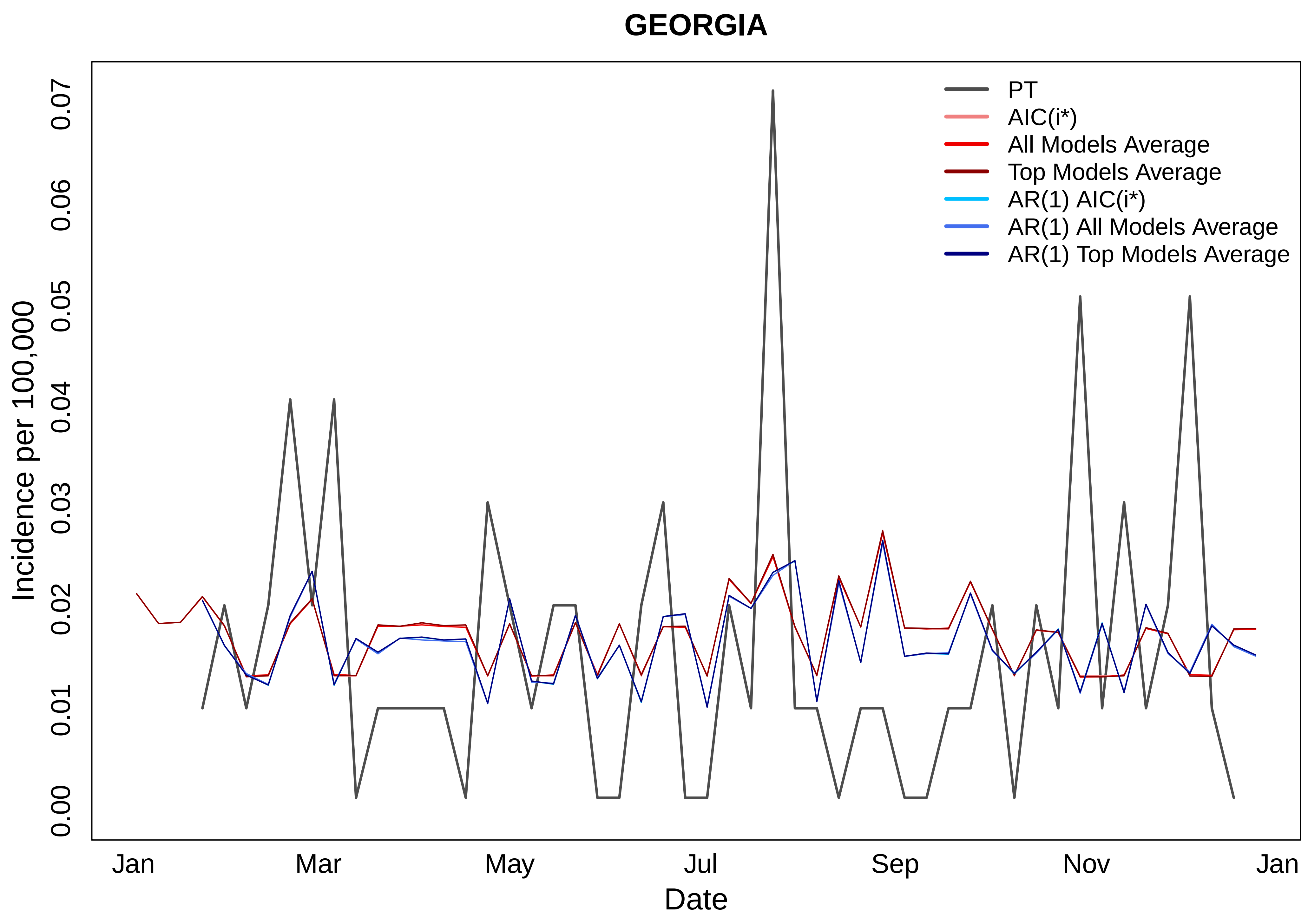

GEORGIA
PT
AIC(i*)
All Models Average
Top Models Average
AR(1) AIC(i*)
AR(1) All Models Average
AR(1) Top Models Average
Incidence per 100,000
0.00
0.01
0.02
0.03
0.04
0.05
0.06
0.07
Jan
Mar
May
Jul
Sep
Nov
Jan
Date

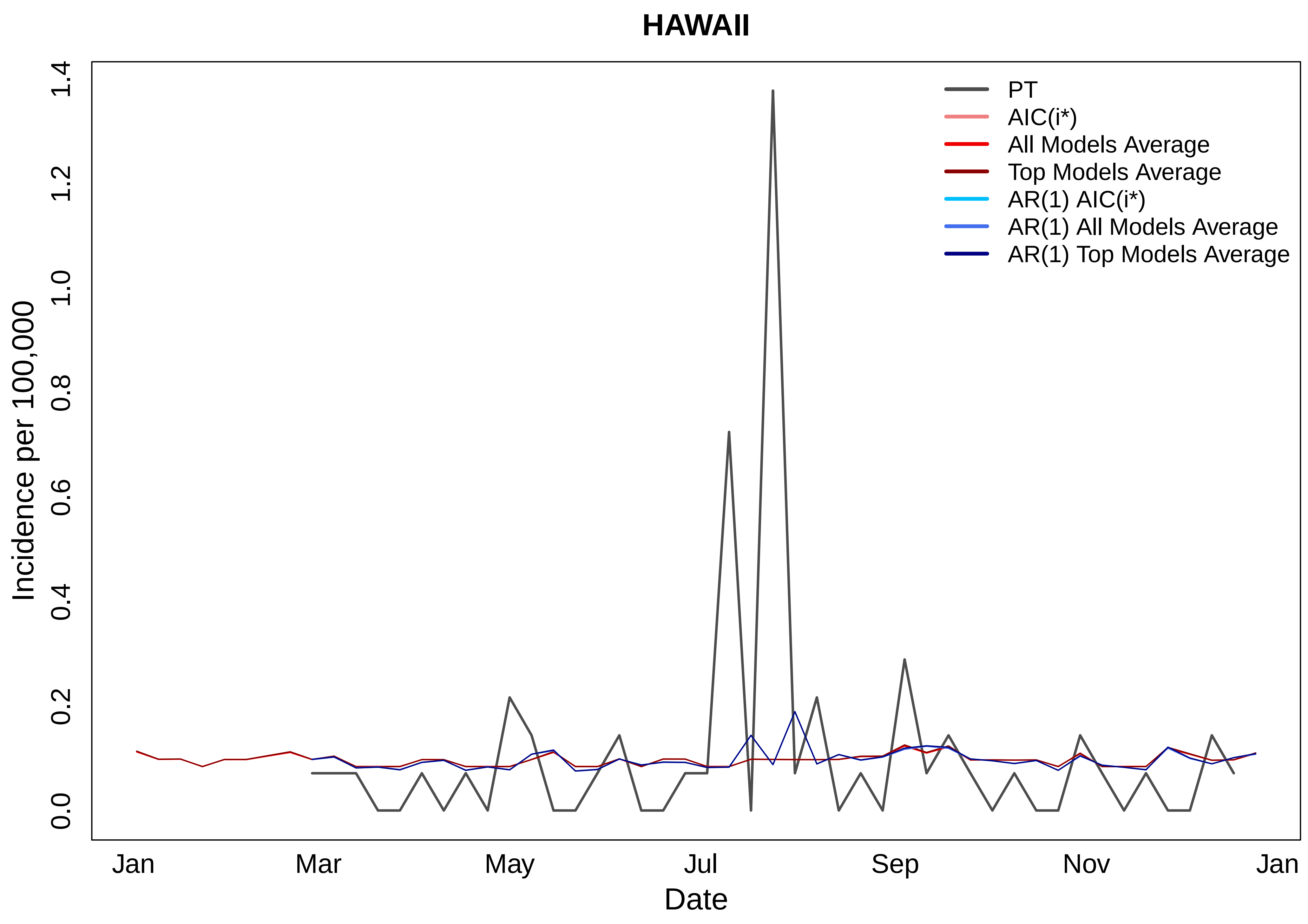

HAWAII
PT
AIC(i*)
All Models Average
Top Models Average
AR(1) AIC(i*)
AR(1) All Models Average
AR(1) Top Models Average
Incidence per 100,000
0.0
0.2
0.4
0.6
0.8
1.0
1.2
1.4
Jan
Mar
May
Jul
Sep
Nov
Jan
Date

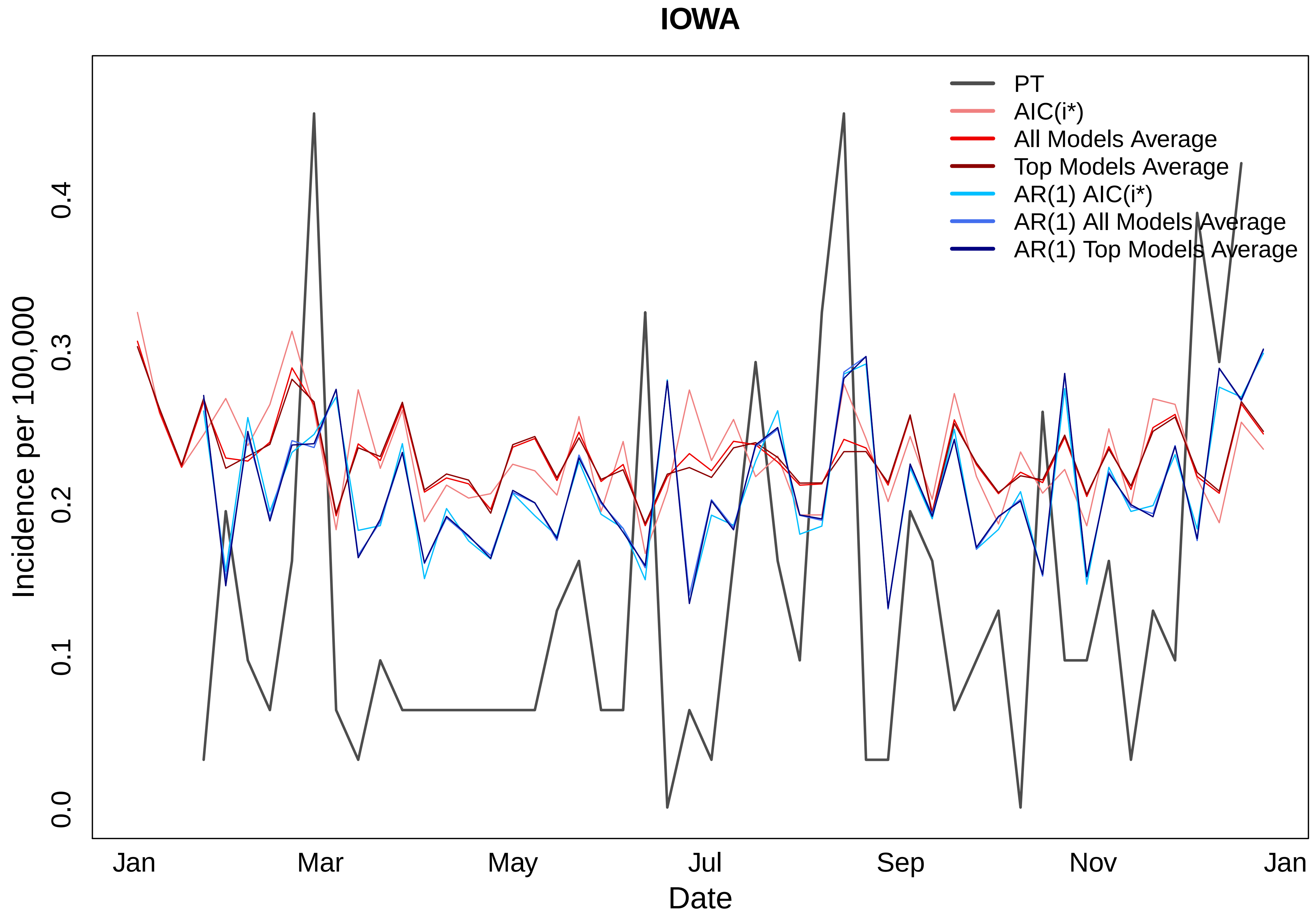

IOWA
PT
AIC(i*)
All Models Average
Top Models Average
AR(1) AIC(i*)
AR(1) All Models Average
AR(1) Top Models Average
Incidence per 100,000
0.0
0.1
0.2
0.3
0.4
Jan
Mar
May
Jul
Sep
Nov
Jan
Date

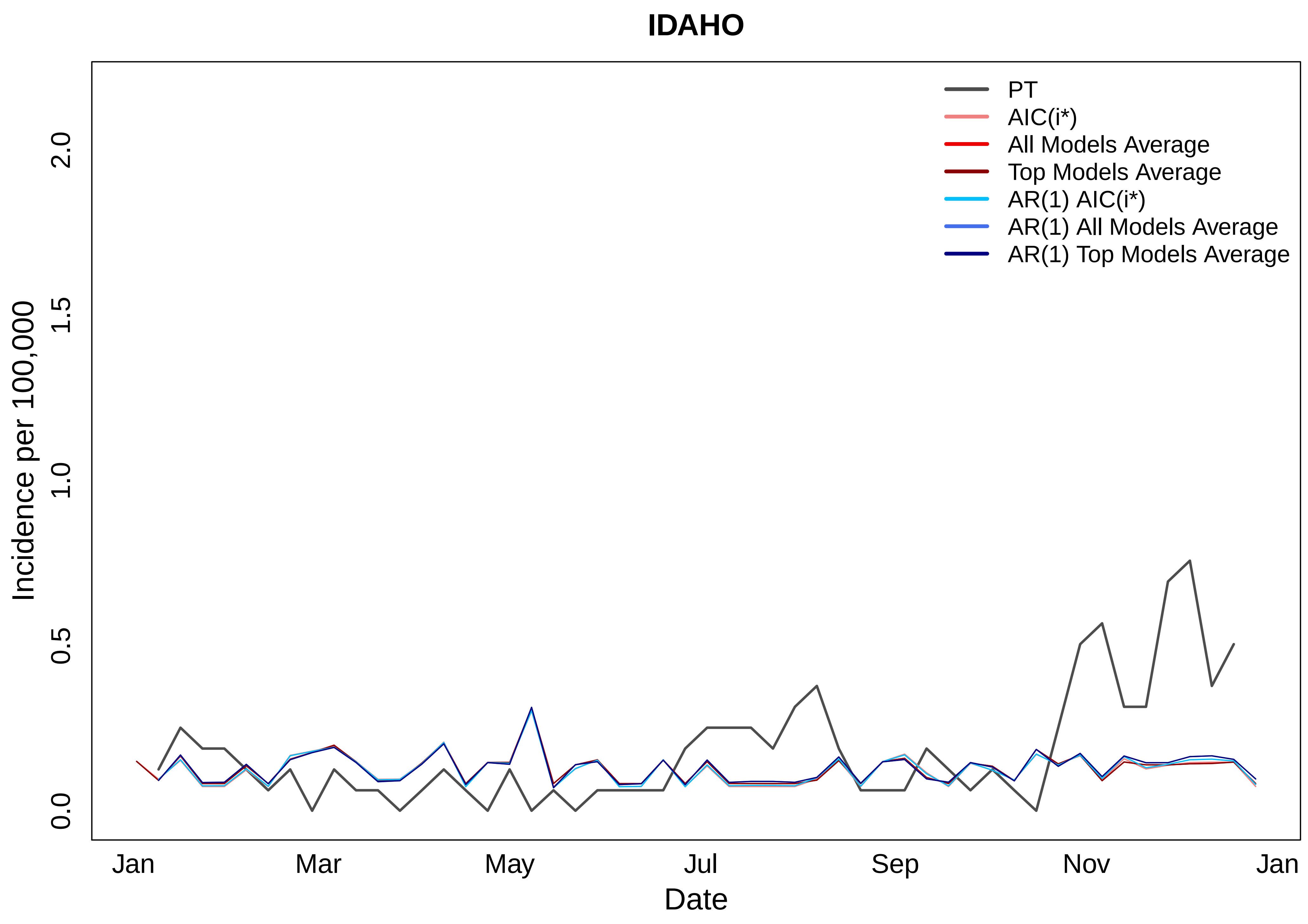

IDAHO
PT
AIC(i*)
All Models Average
Top Models Average
AR(1) AIC(i*)
AR(1) All Models Average
AR(1) Top Models Average
Incidence per 100,000
0.0
0.5
1.0
1.5
2.0
Jan
Mar
May
Jul
Sep
Nov
Jan
Date

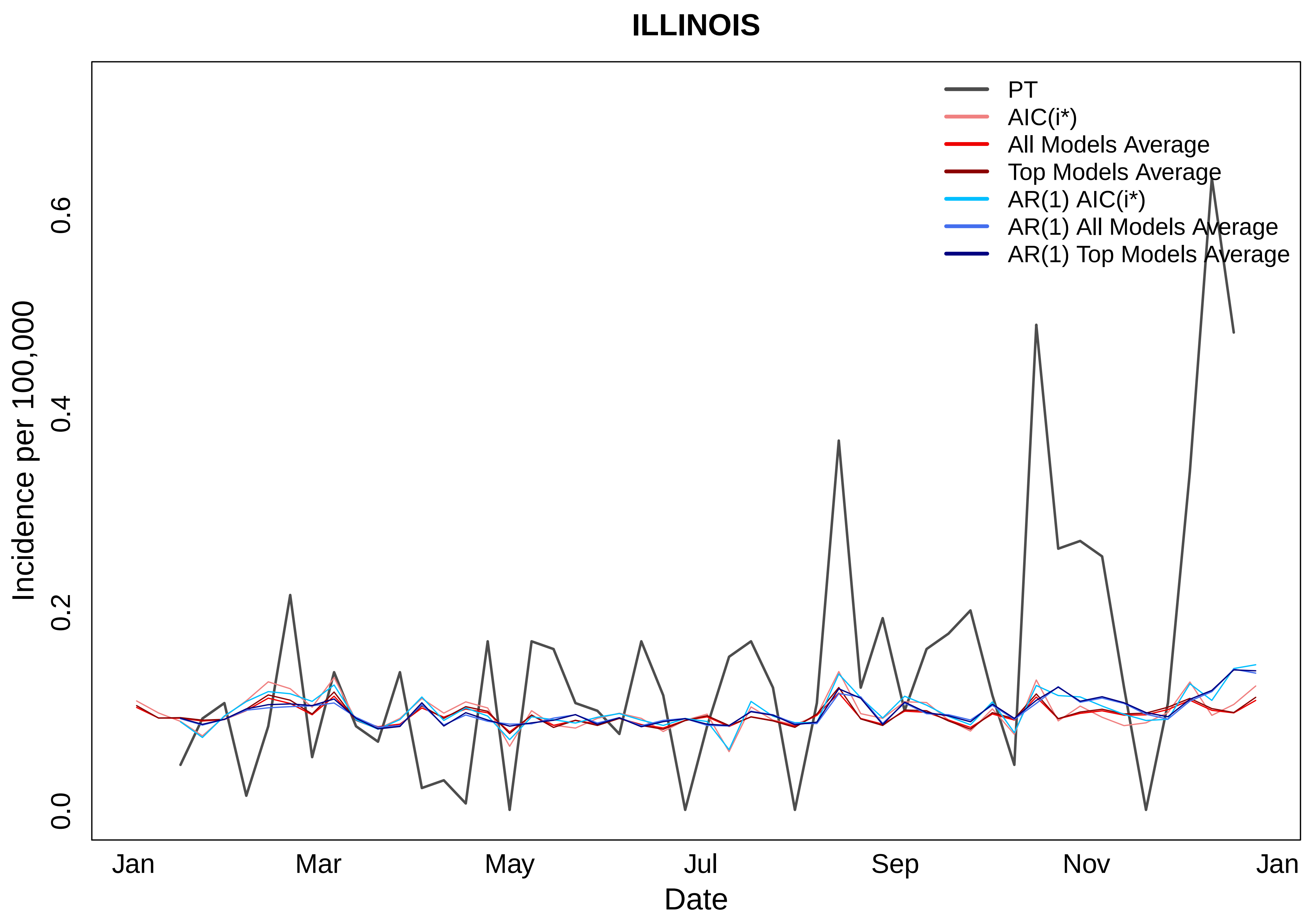
ILLINOIS
PT
AIC(i*)
All Models Average
Top Models Average
AR(1) AIC(i*)
AR(1) All Models Average
AR(1) Top Models Average
Incidence per 100,000
0.0
0.2
0.4
0.6
Jan
Mar
May
Jul
Sep
Nov
Jan
Date

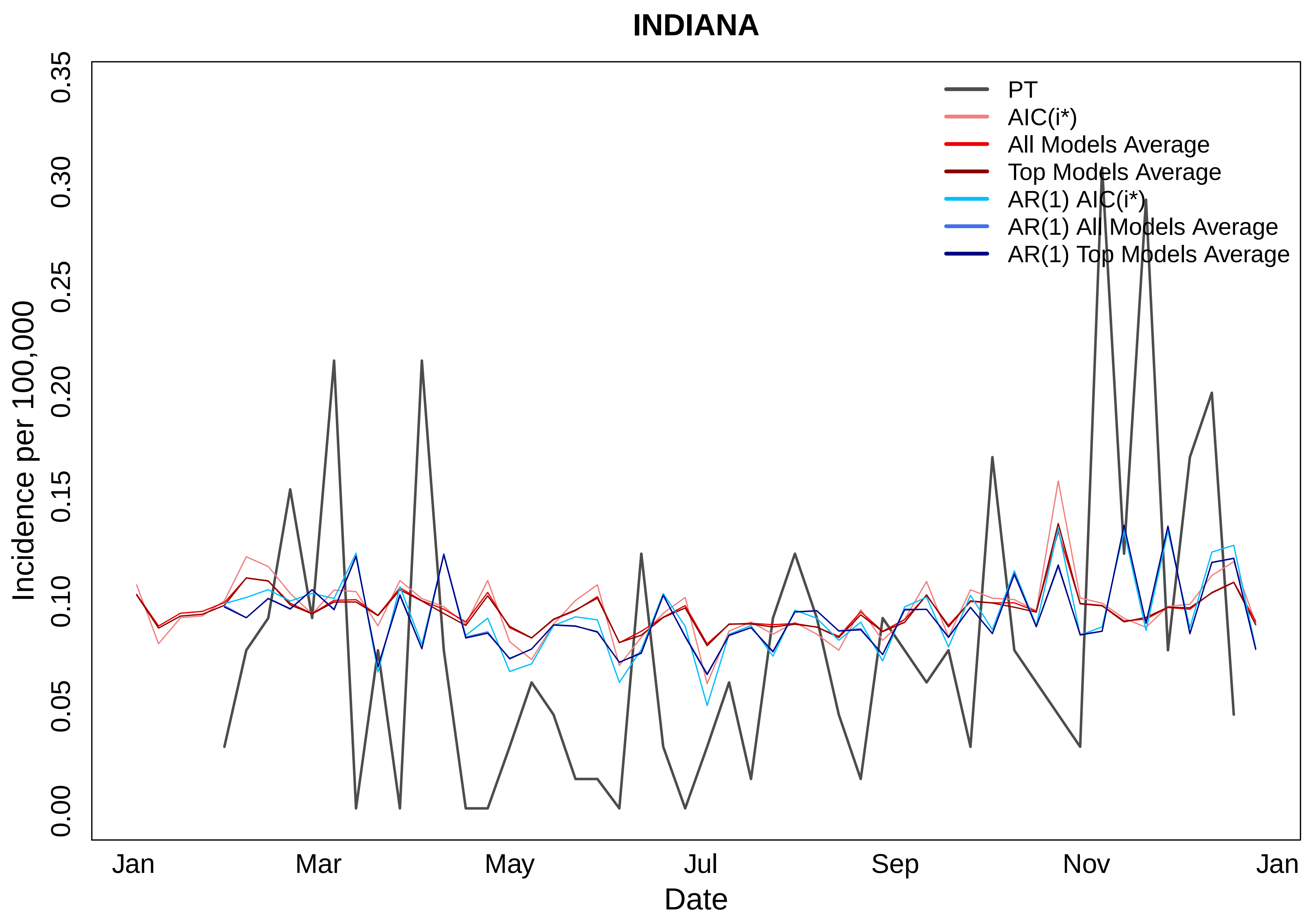
INDIANA
PT
AIC(i*)
All Models Average
Top Models Average
AR(1) AIC(i*)
AR(1) All Models Average
AR(1) Top Models Average
Incidence per 100,000
0.00
0.05
0.10
0.15
0.20
0.25
0.30
0.35
Jan
Mar
May
Jul
Sep
Nov
Jan
Date

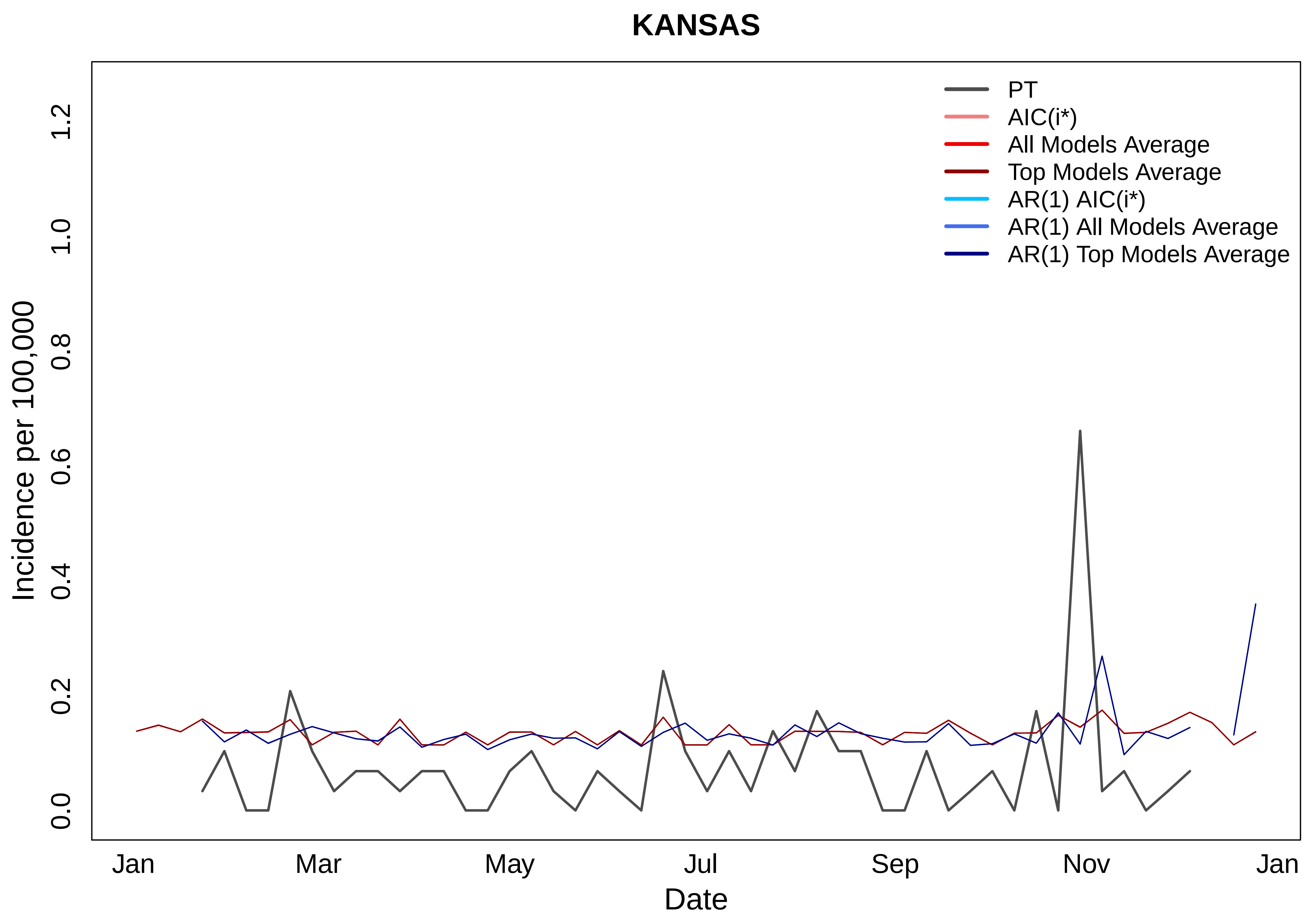
KANSAS
PT
AIC(i*)
All Models Average
Top Models Average
AR(1) AIC(i*)
AR(1) All Models Average
AR(1) Top Models Average
Incidence per 100,000
0.0
0.2
0.4
0.6
0.8
1.0
1.2
Jan
Mar
May
Jul
Sep
Nov
Jan
Date

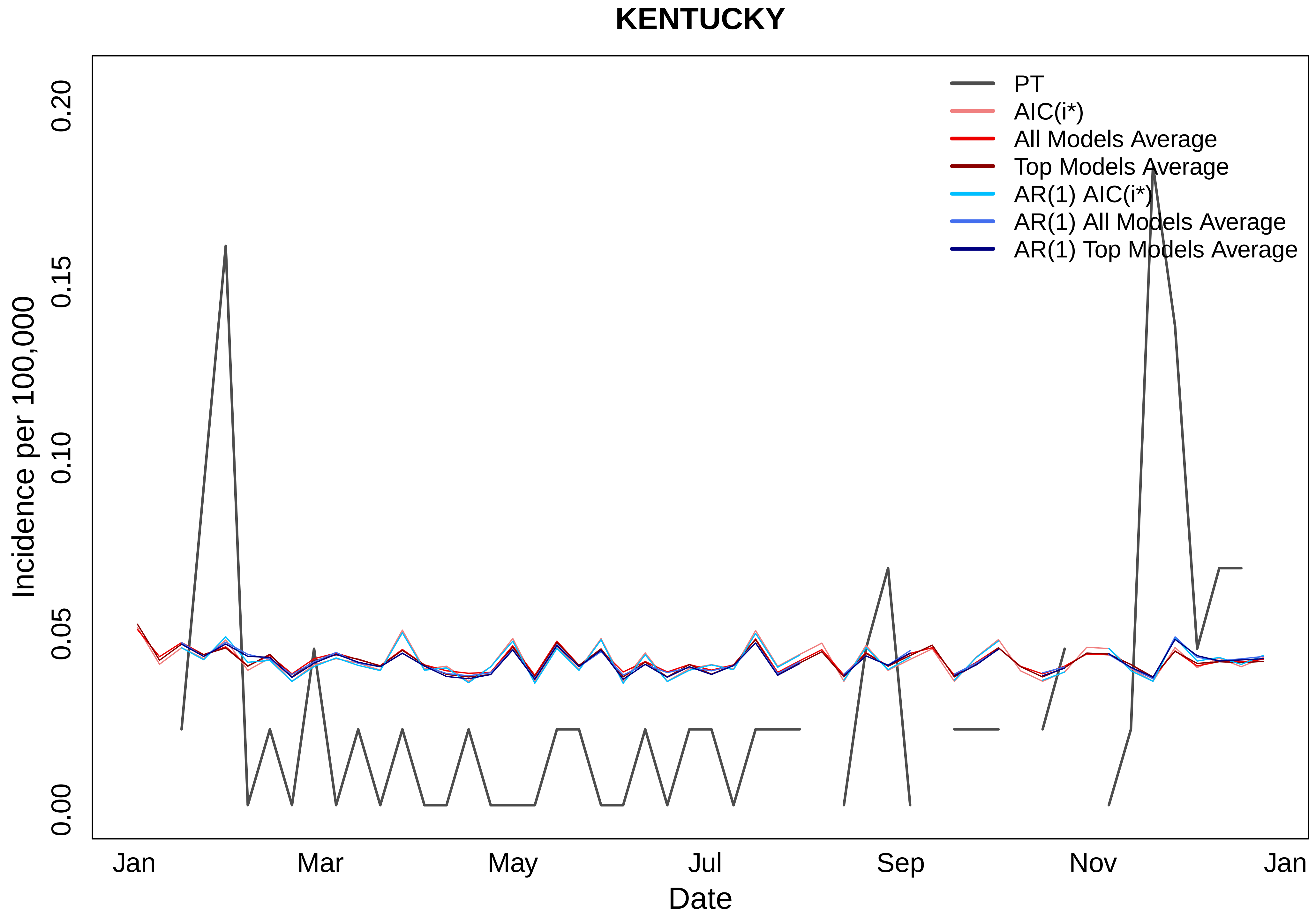

KENTUCKY
PT
AIC(i*)
All Models Average
Top Models Average
AR(1) AIC(i*)
AR(1) All Models Average
AR(1) Top Models Average
Incidence per 100,000
0.00
0.05
0.10
0.15
0.20
Jan
Mar
May
Jul
Sep
Nov
Jan
Date

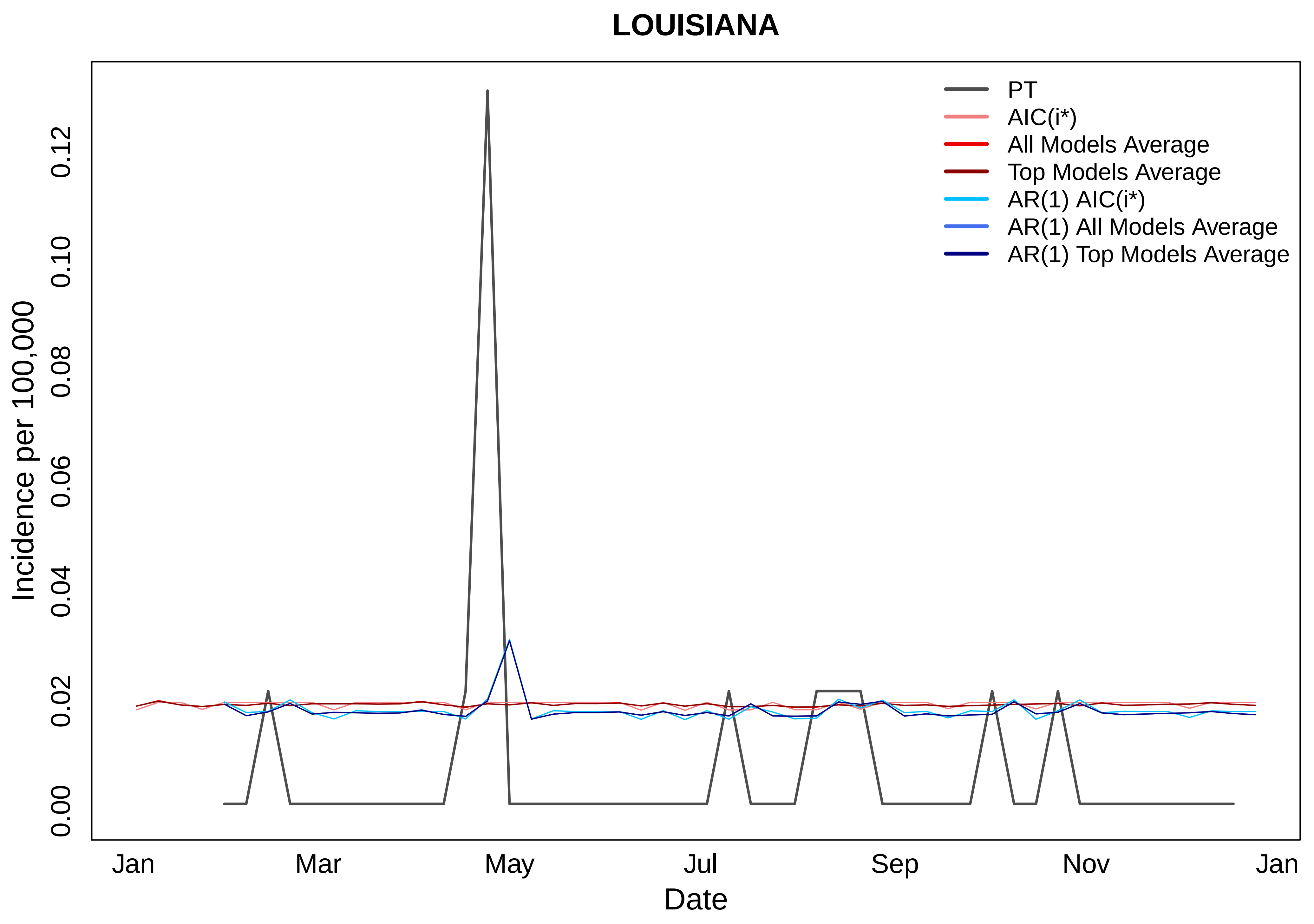

LOUISIANA
PT
AIC(i*)
All Models Average
Top Models Average
AR(1) AIC(i*)
AR(1) All Models Average
AR(1) Top Models Average
Incidence per 100,000
0.00
0.02
0.04
0.06
0.08
0.10
0.12
Jan
Mar
May
Jul
Sep
Nov
Jan
Date

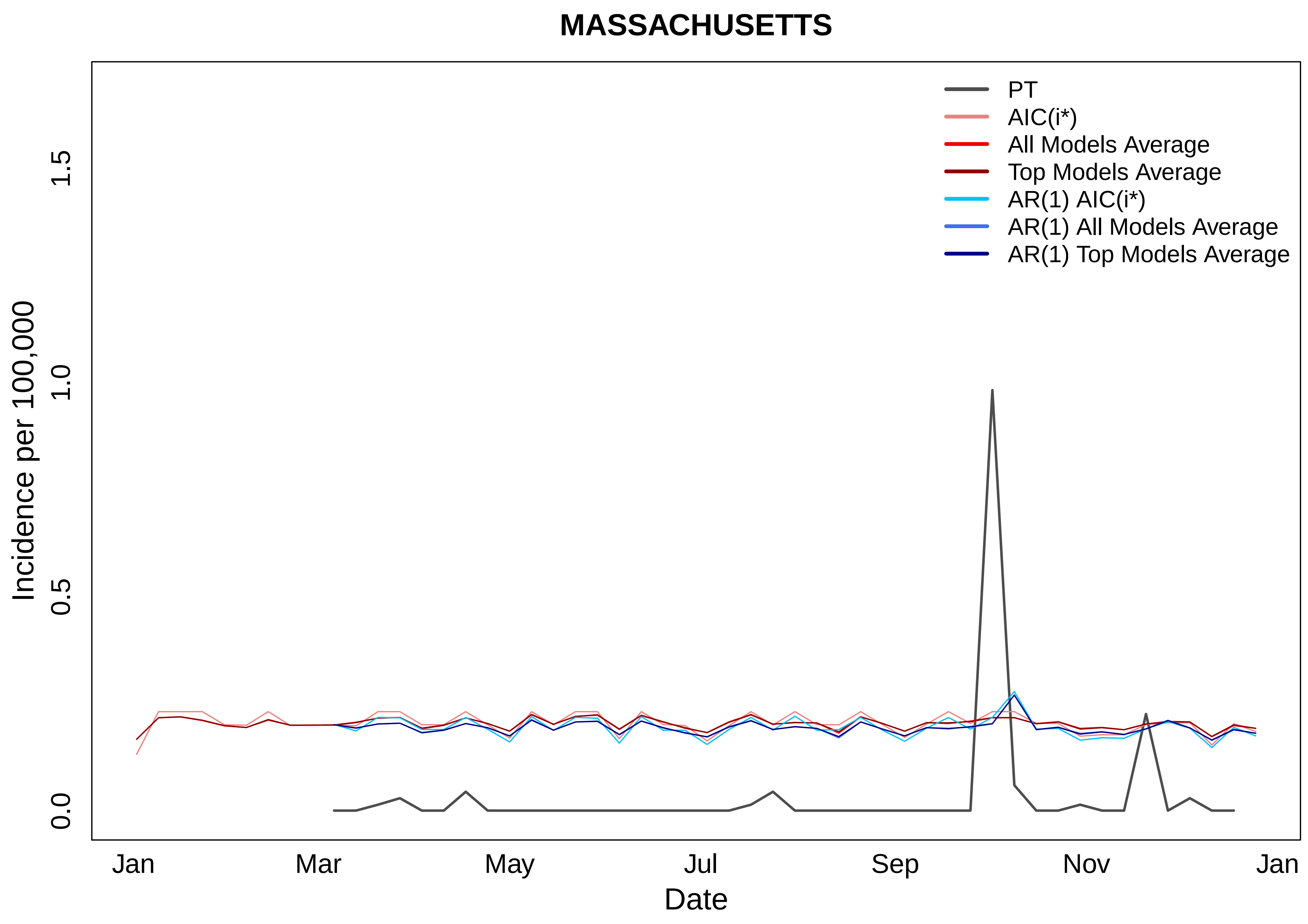
MASSACHUSETTS
PT
AIC(i*)
All Models Average
Top Models Average
AR(1) AIC(i*)
AR(1) All Models Average
AR(1) Top Models Average
Incidence per 100,000
0.0
0.5
1.0
1.5
Jan
Mar
May
Jul
Sep
Nov
Jan
Date

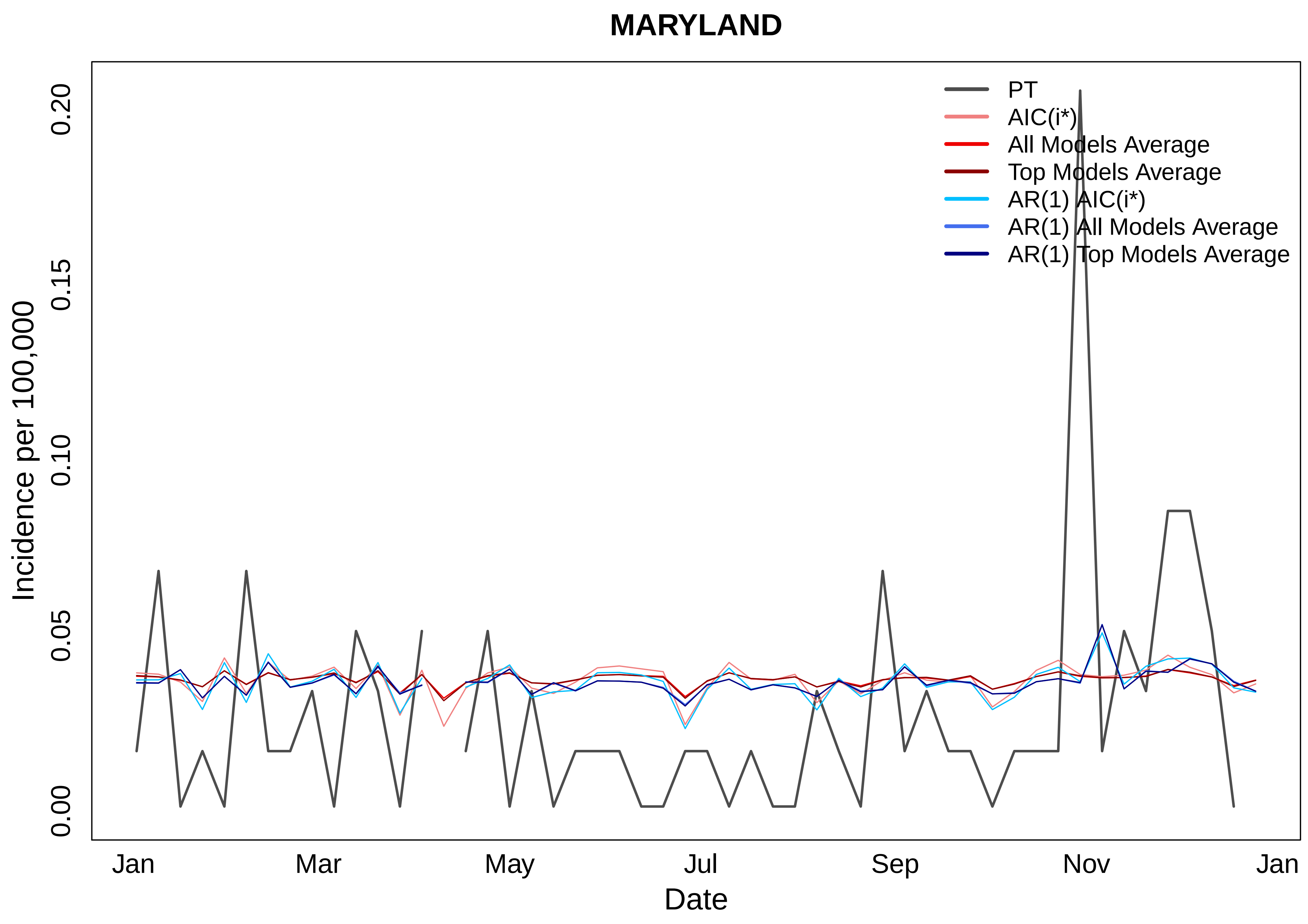
MARYLAND
PT
AIC(i*)
All Models Average
Top Models Average
AR(1) AIC(i*)
AR(1) All Models Average
AR(1) Top Models Average
Incidence per 100,000
0.00
0.05
0.10
0.15
0.20
Jan
Mar
May
Jul
Sep
Nov
Jan
Date

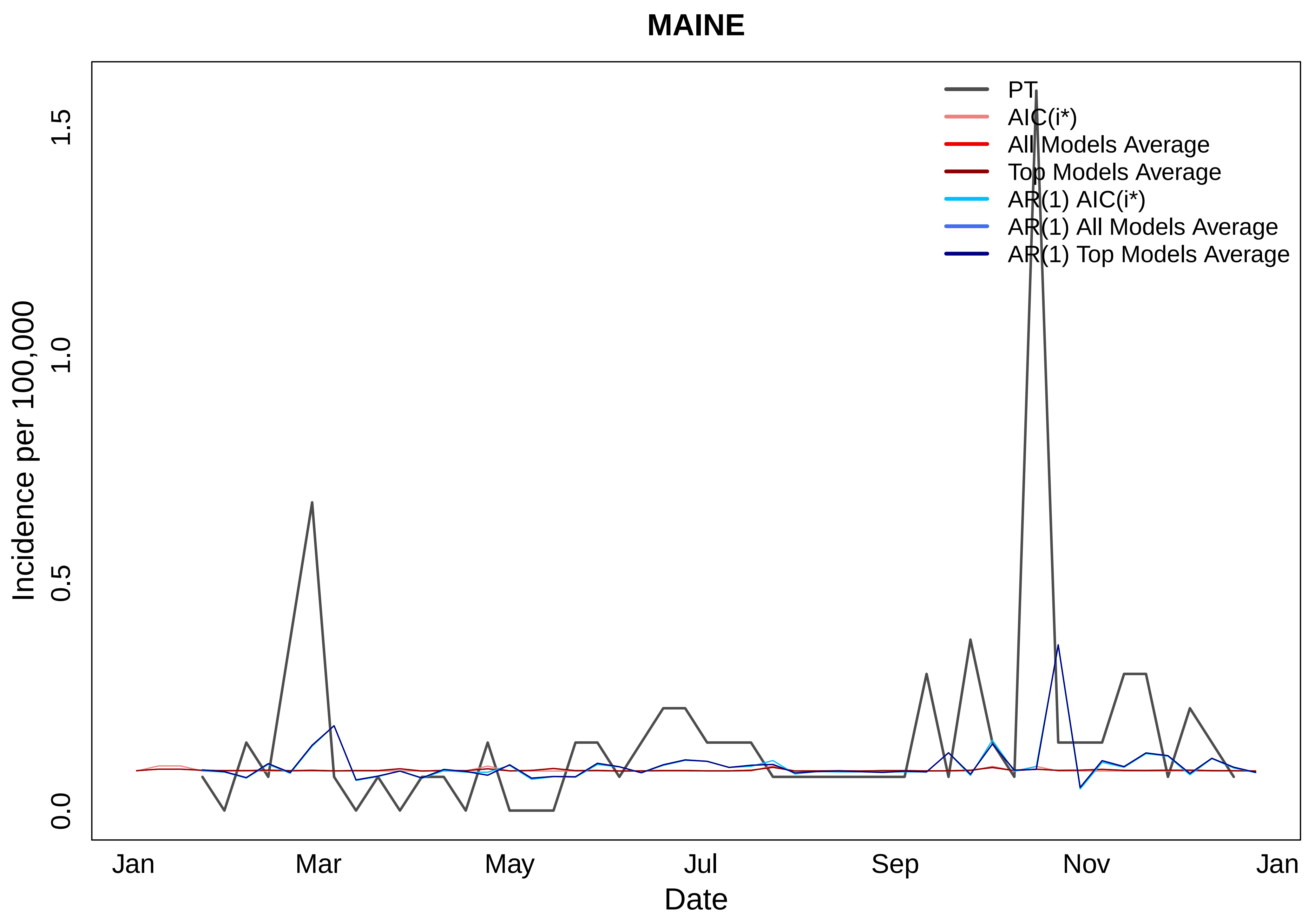

MAINE
PT
AIC(i*)
All Models Average
Top Models Average
AR(1) AIC(i*)
AR(1) All Models Average
AR(1) Top Models Average
Incidence per 100,000
0.0
0.5
1.0
1.5
Jan
Mar
May
Jul
Sep
Nov
Jan
Date

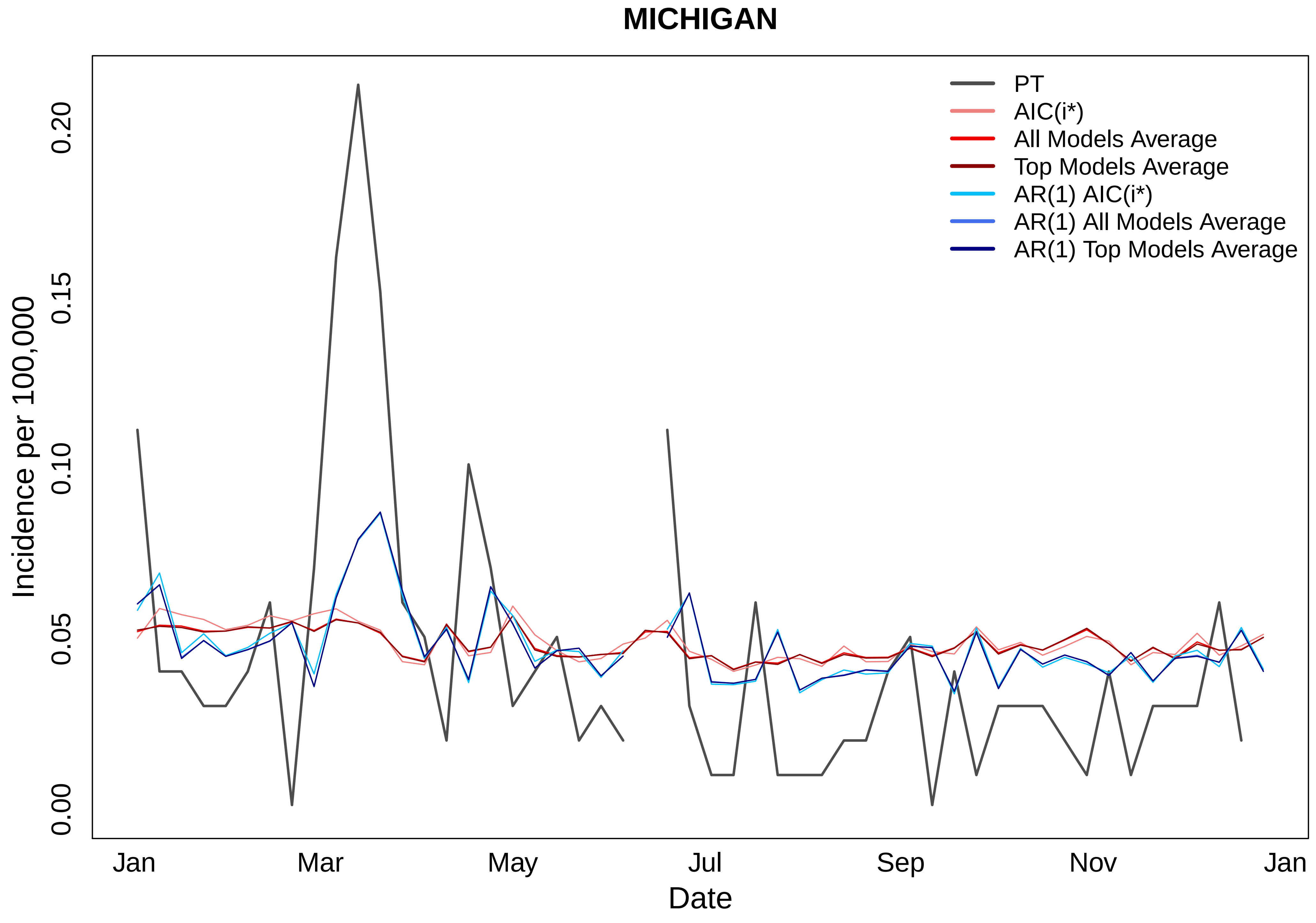
MICHIGAN
PT
AIC(i*)
All Models Average
Top Models Average
AR(1) AIC(i*)
AR(1) All Models Average
AR(1) Top Models Average
Incidence per 100,000
0.00
0.05
0.10
0.15
0.20
Jan
Mar
May
Jul
Sep
Nov
Jan
Date

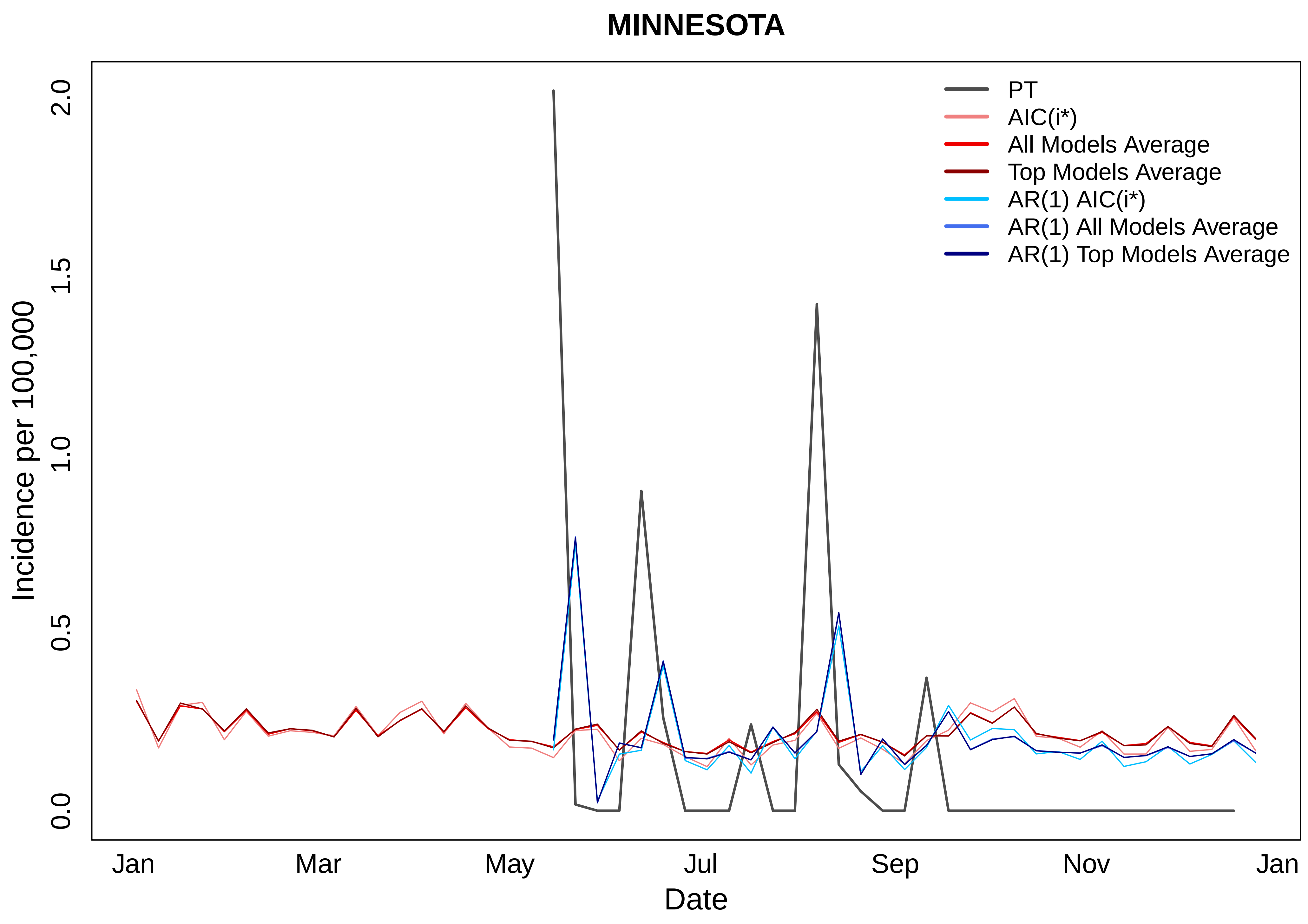

MINNESOTA
PT
AIC(i*)
All Models Average
Top Models Average
AR(1) AIC(i*)
AR(1) All Models Average
AR(1) Top Models Average
Incidence per 100,000
0.0
0.5
1.0
1.5
2.0
Jan
Mar
May
Jul
Sep
Nov
Jan
Date

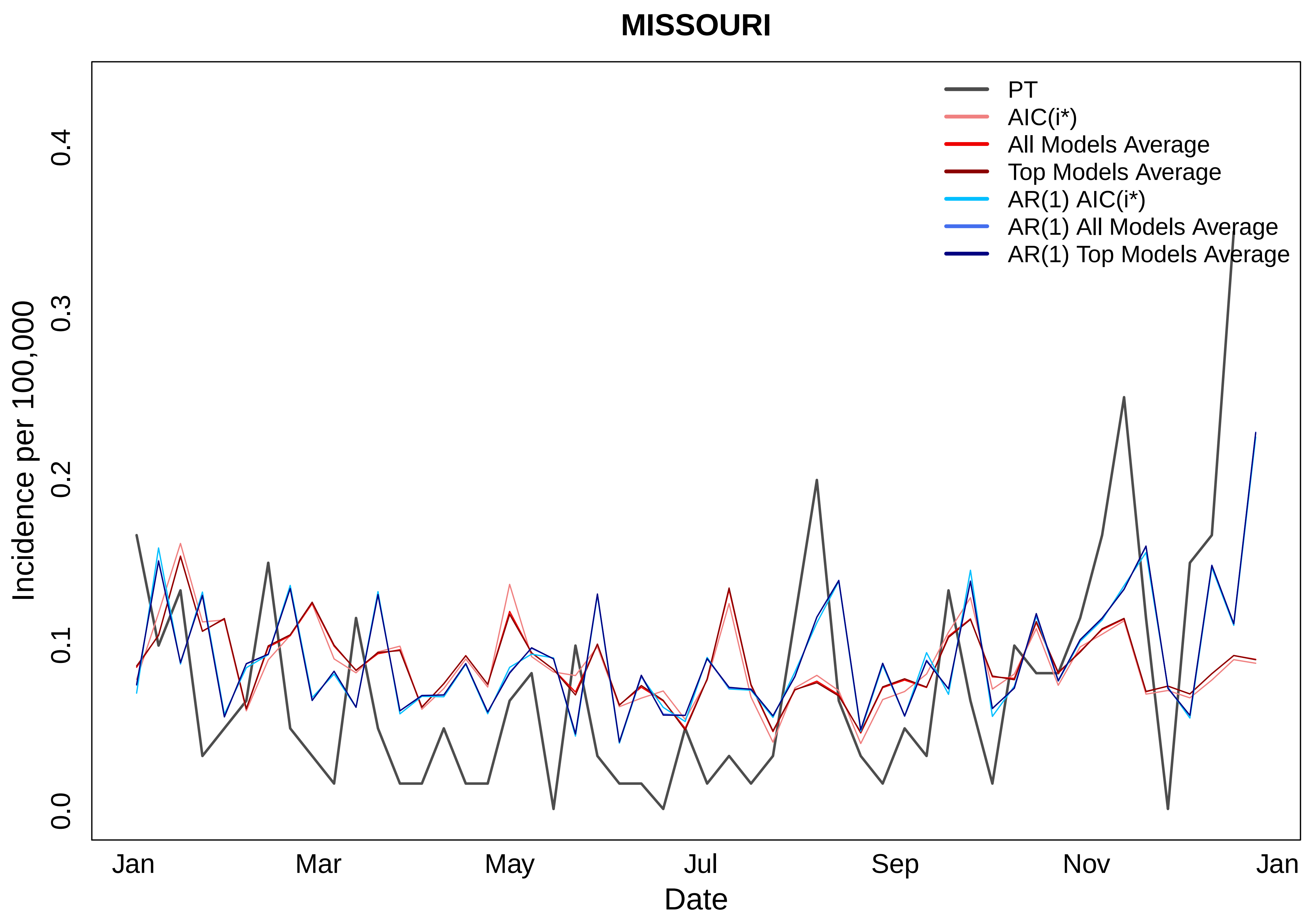
MISSOURI
PT
AIC(i*)
All Models Average
Top Models Average
AR(1) AIC(i*)
AR(1) All Models Average
AR(1) Top Models Average
Incidence per 100,000
0.0
0.1
0.2
0.3
0.4
Jan
Mar
May
Jul
Sep
Nov
Jan
Date

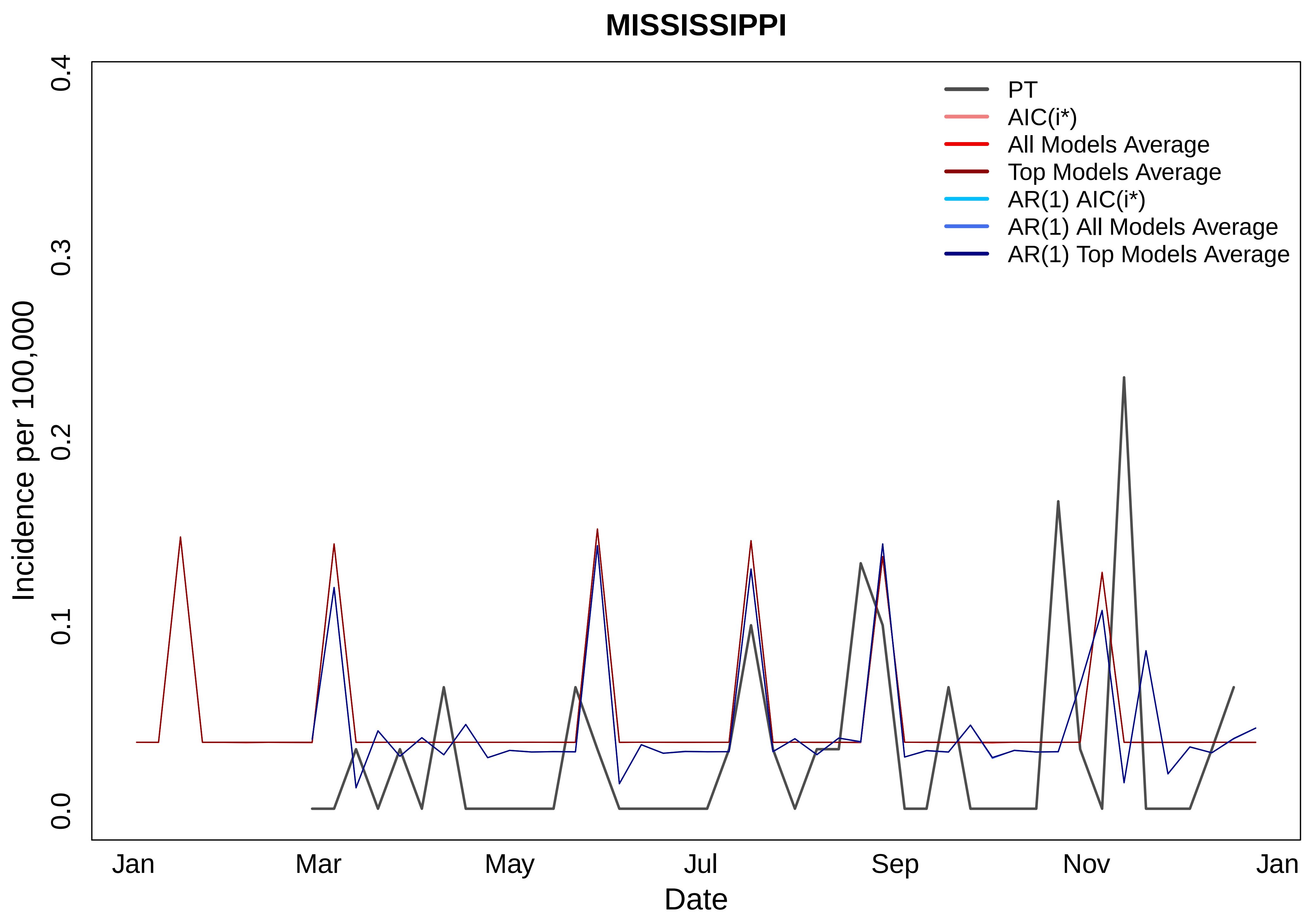

MISSISSIPPI
Incidence per 100,000
0.0
0.1
0.2
0.3
0.4
Jan
Mar
May
Jul
Sep
Nov
Jan
Date
PT
AIC(i*)
All Models Average
Top Models Average
AR(1) AIC(i*)
AR(1) All Models Average
AR(1) Top Models Average

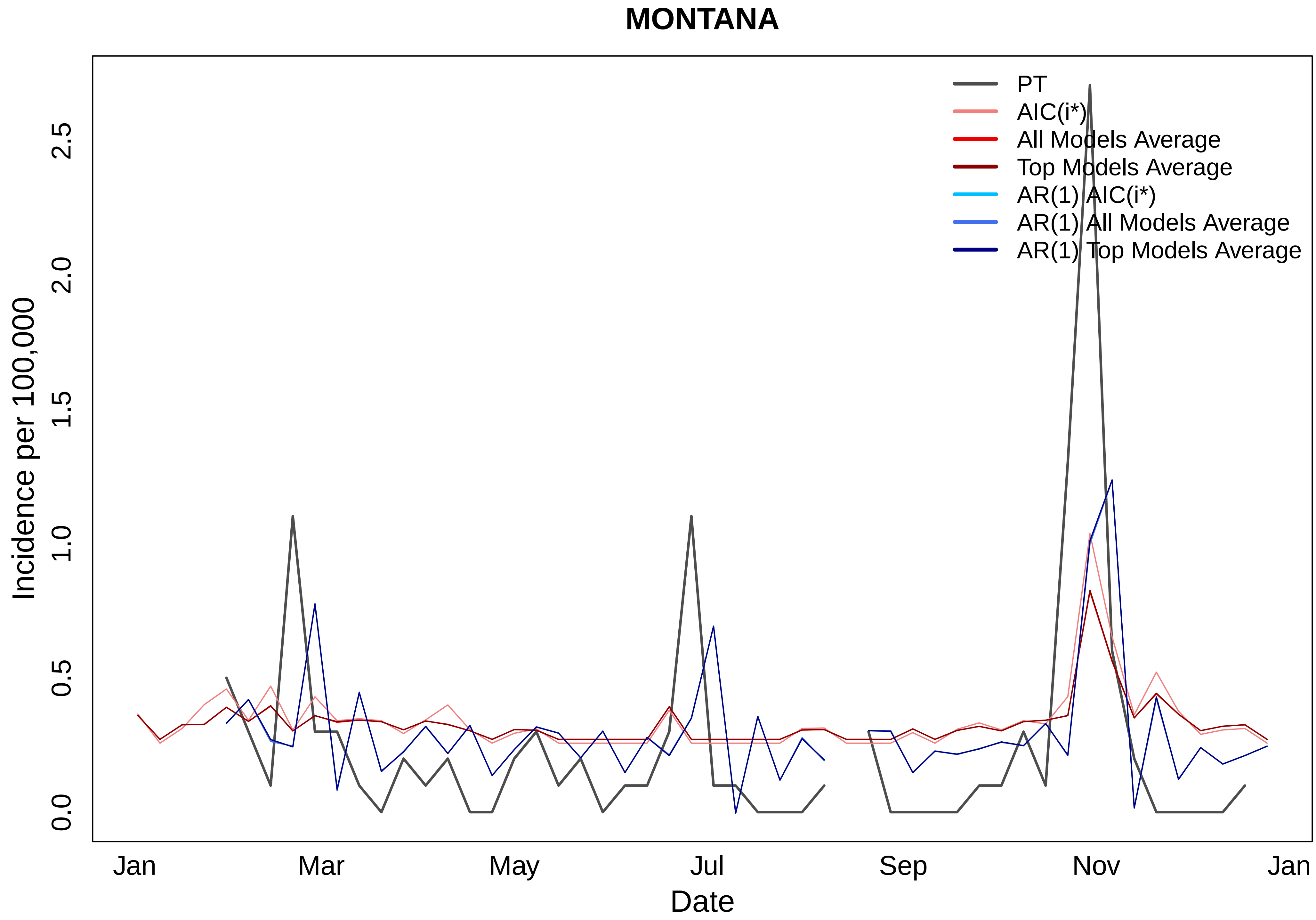
MONTANA
PT
AIC(i*)
All Models Average
Top Models Average
AR(1) AIC(i*)
AR(1) All Models Average
AR(1) Top Models Average
Incidence per 100,000
0.0
0.5
1.0
1.5
2.0
2.5
Jan
Mar
May
Jul
Sep
Nov
Jan
Date

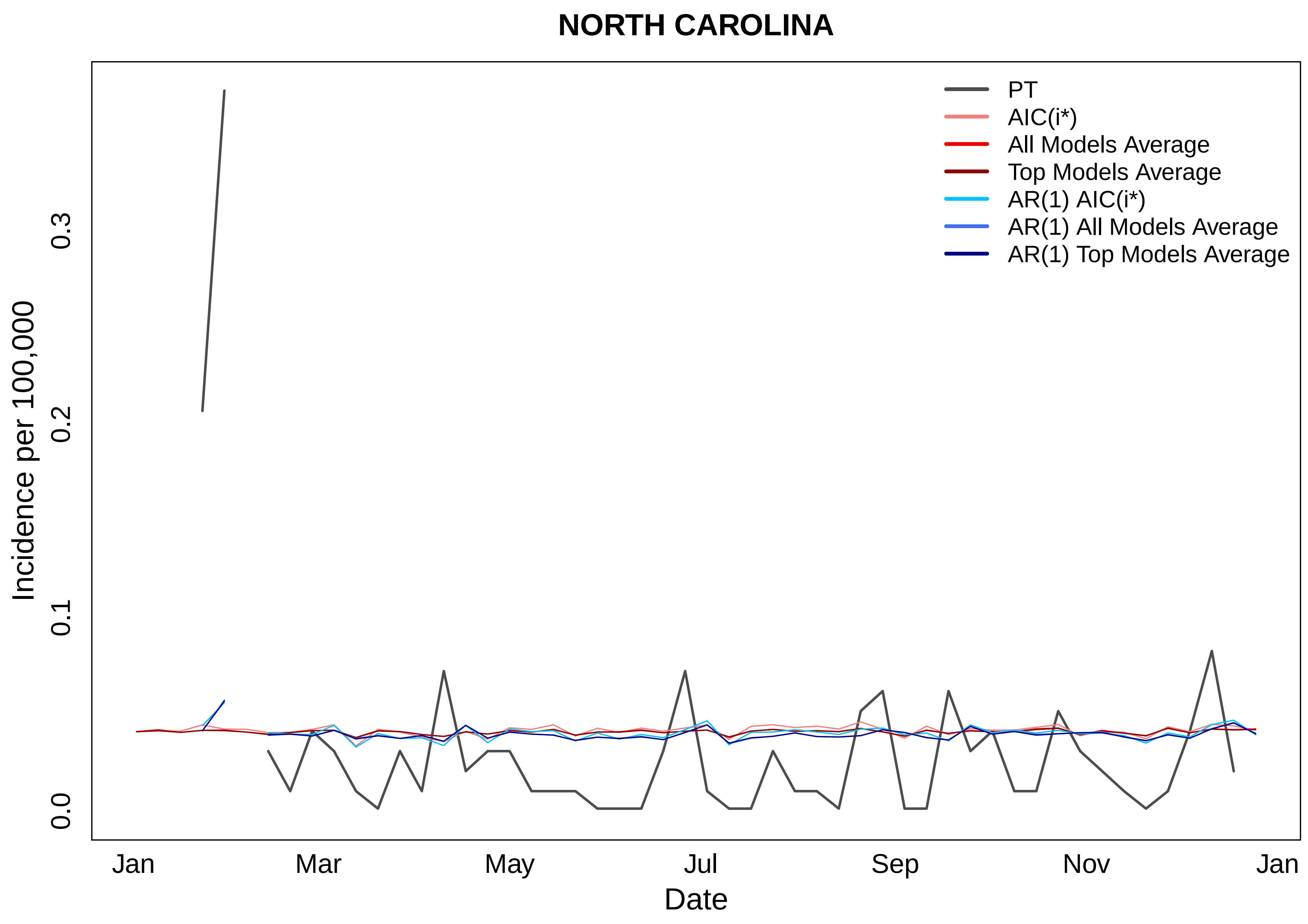

NORTH CAROLINA
PT
AIC(i*)
All Models Average
Top Models Average
AR(1) AIC(i*)
AR(1) All Models Average
AR(1) Top Models Average
Incidence per 100,000
0.0
0.1
0.2
0.3
Jan
Mar
May
Jul
Sep
Nov
Jan
Date

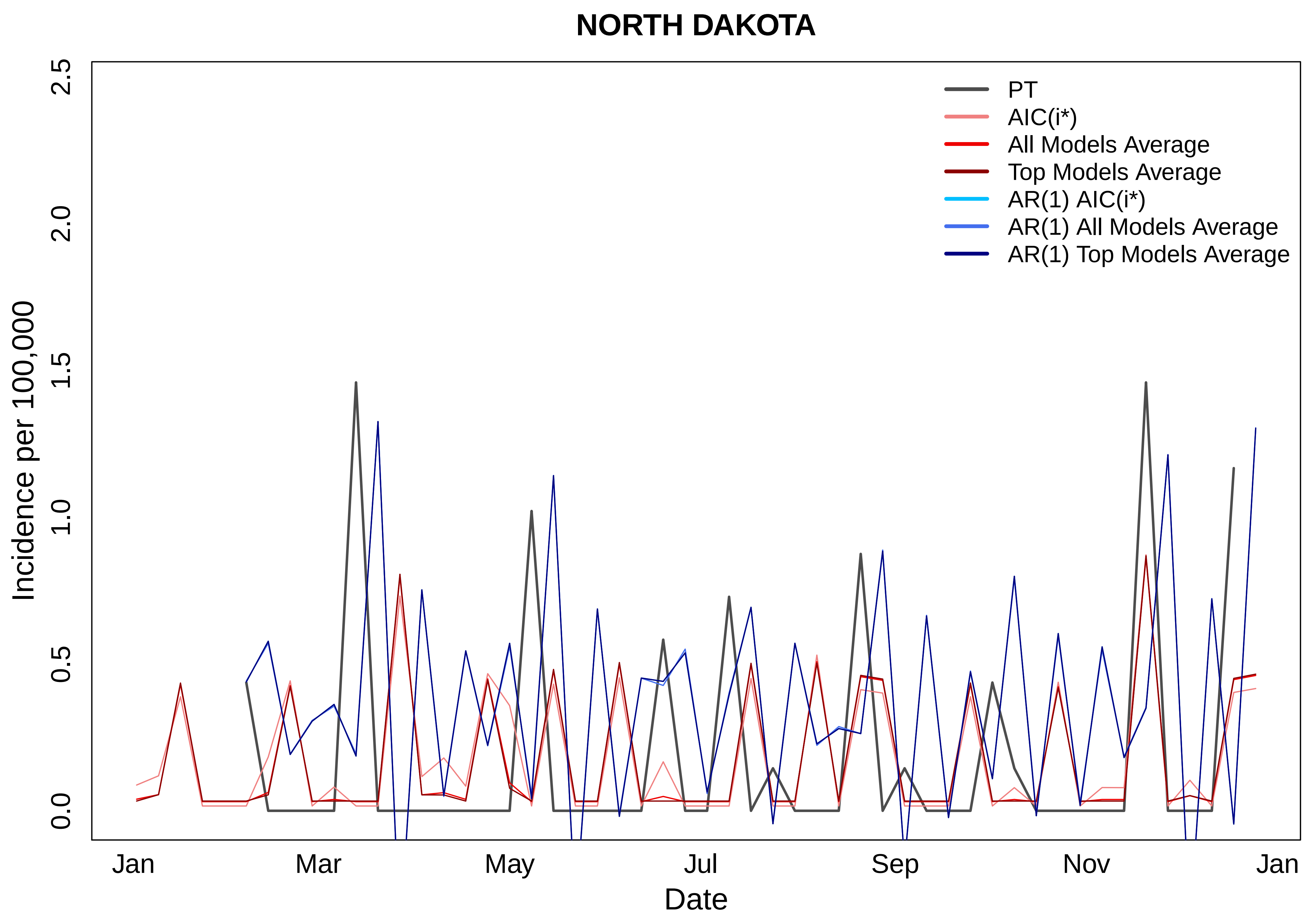
NORTH DAKOTA
PT
AIC(i*)
All Models Average
Top Models Average
AR(1) AIC(i*)
AR(1) All Models Average
AR(1) Top Models Average
Incidence per 100,000
0.0
0.5
1.0
1.5
2.0
2.5
Jan
Mar
May
Jul
Sep
Nov
Jan
Date

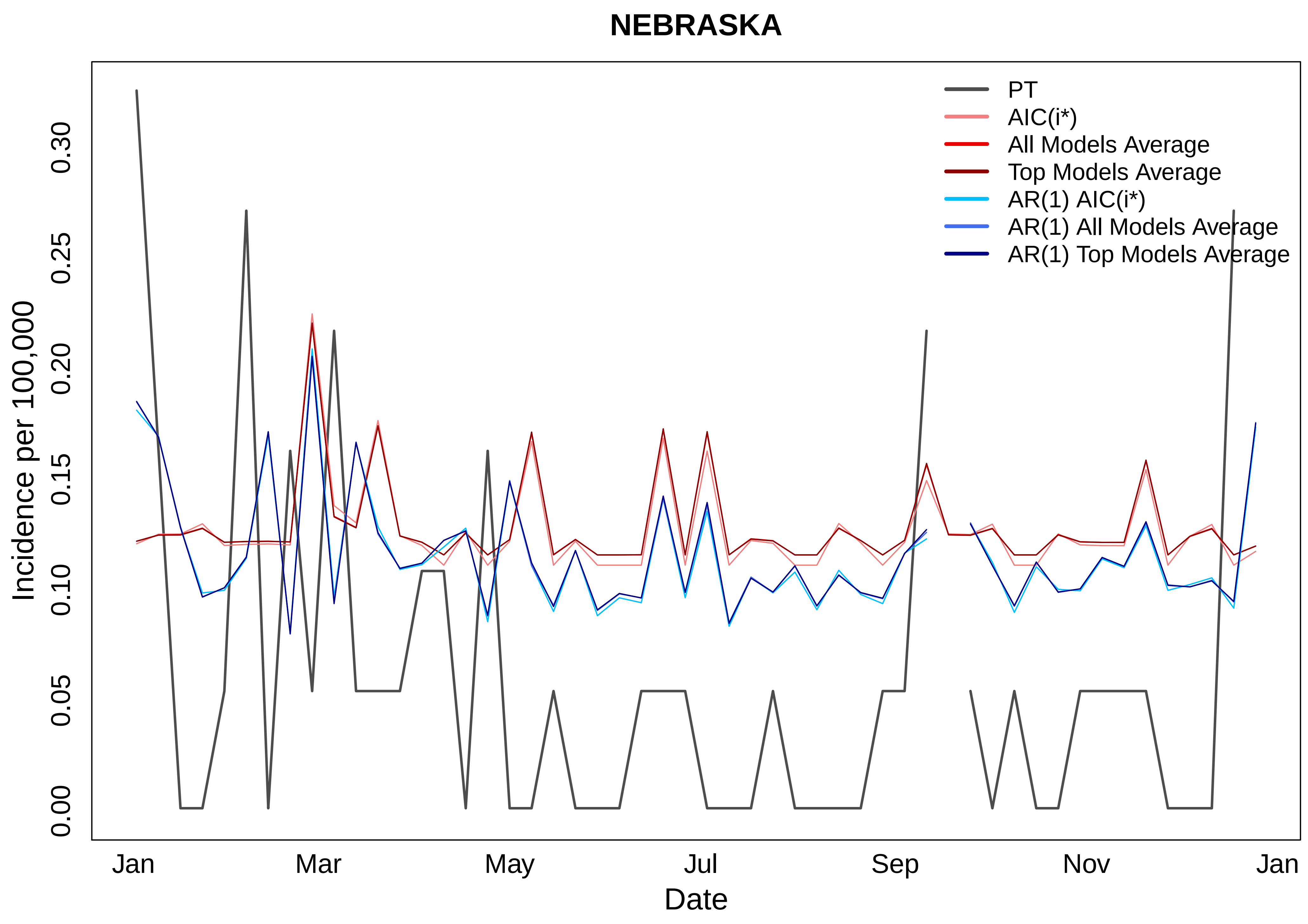
NEBRASKA
PT
AIC(i*)
All Models Average
Top Models Average
AR(1) AIC(i*)
AR(1) All Models Average
AR(1) Top Models Average
Incidence per 100,000
0.00
0.05
0.10
0.15
0.20
0.25
0.30
Jan
Mar
May
Jul
Sep
Nov
Jan
Date

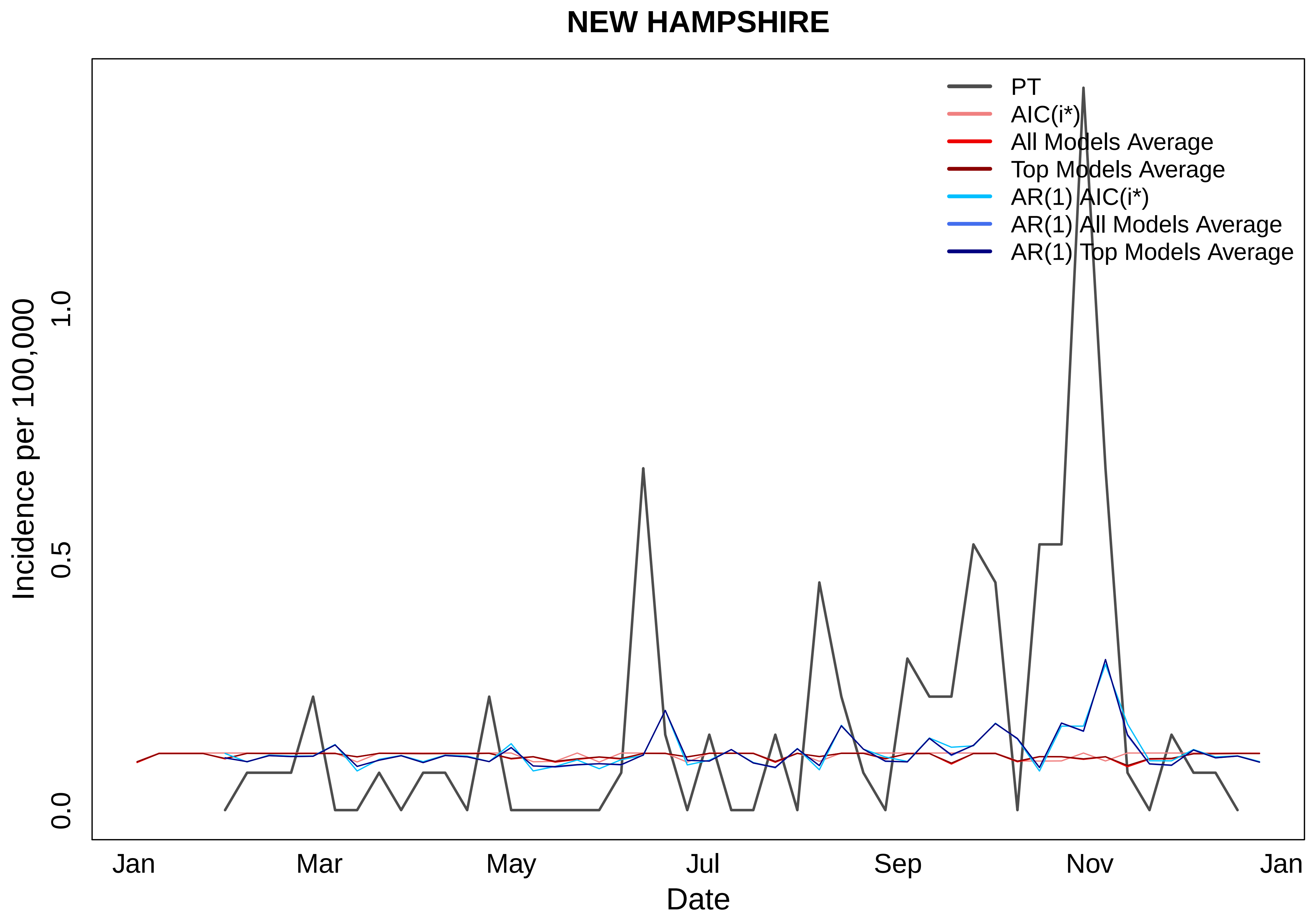

NEW HAMPSHIRE
PT
AIC(i*)
All Models Average
Top Models Average
AR(1) AIC(i*)
AR(1) All Models Average
AR(1) Top Models Average
Incidence per 100,000
0.0
0.5
1.0
Jan
Mar
May
Jul
Sep
Nov
Jan
Date

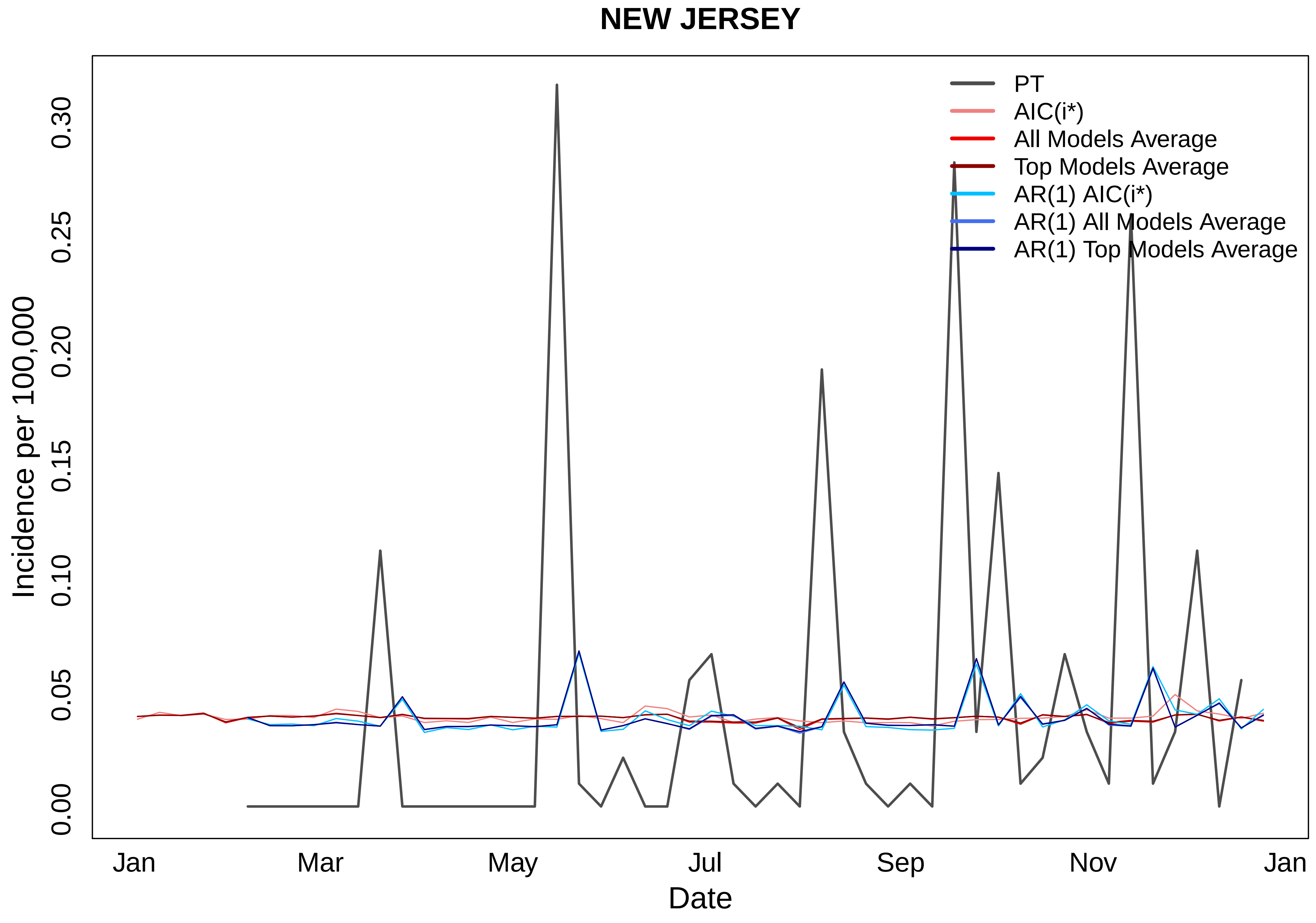
NEW JERSEY
PT
AIC(i*)
All Models Average
Top Models Average
AR(1) AIC(i*)
AR(1) All Models Average
AR(1) Top Models Average
Incidence per 100,000
0.00
0.05
0.10
0.15
0.20
0.25
0.30
Jan
Mar
May
Jul
Sep
Nov
Jan
Date

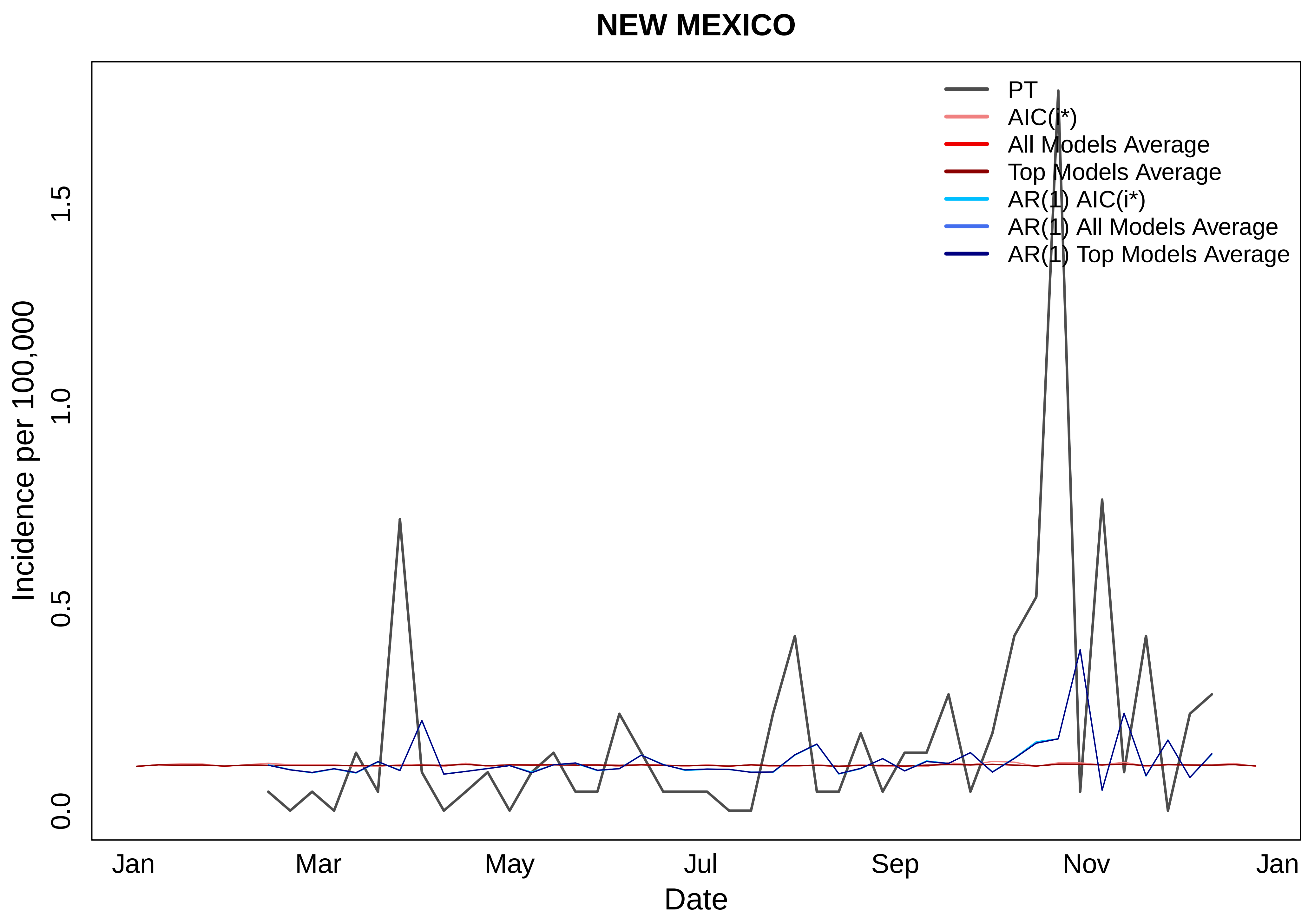
NEW MEXICO
PT
AIC(i*)
All Models Average
Top Models Average
AR(1) AIC(i*)
AR(1) All Models Average
AR(1) Top Models Average
Incidence per 100,000
0.0
0.5
1.0
1.5
Jan
Mar
May
Jul
Sep
Nov
Jan
Date

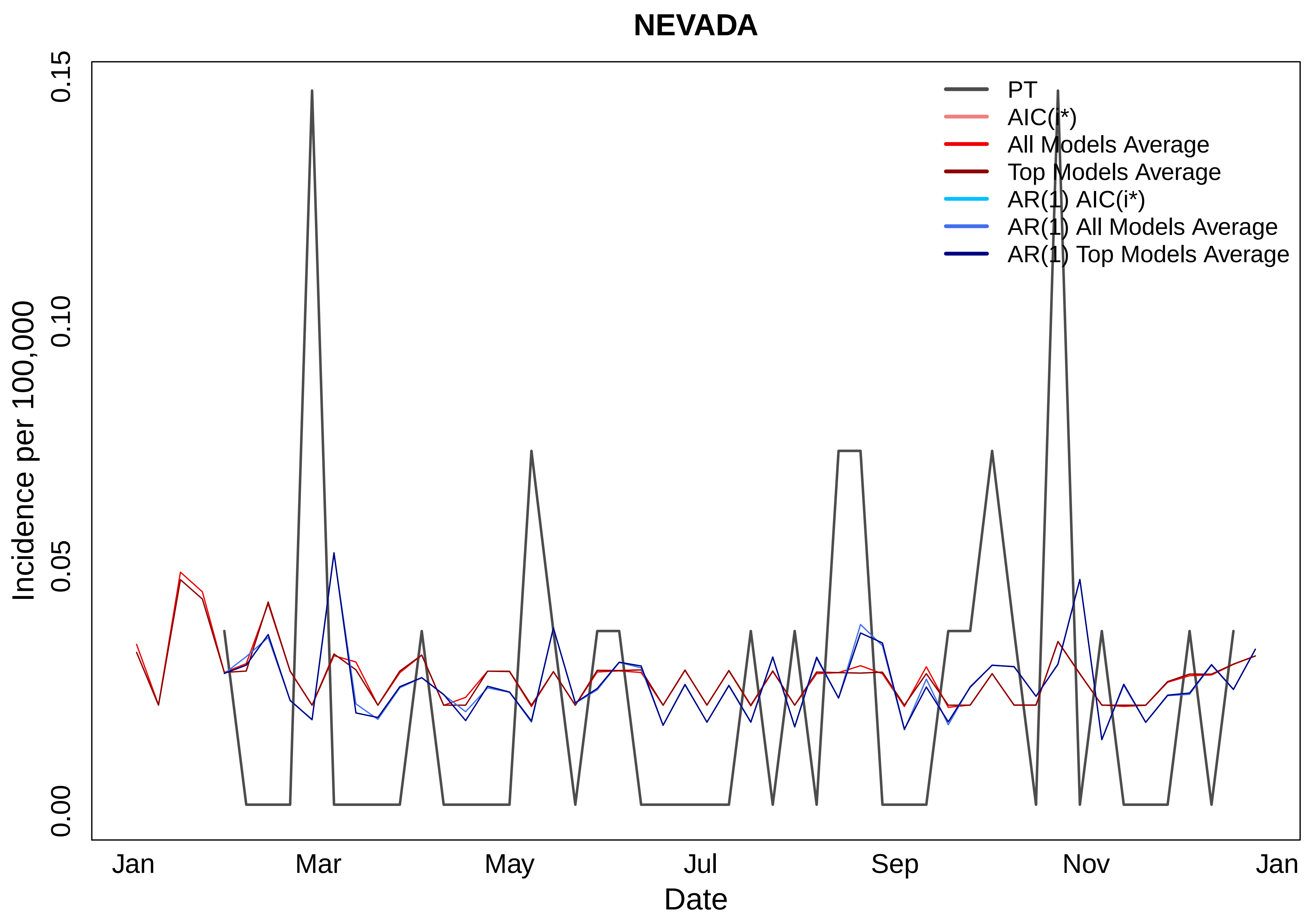

NEVADA
0.15
0.10
0.05
0.00
Incidence per 100,000
Jan
Mar
May
Jul
Sep
Nov
Jan
Date
PT
AIC(i*)
All Models Average
Top Models Average
AR(1) AIC(i*)
AR(1) All Models Average
AR(1) Top Models Average

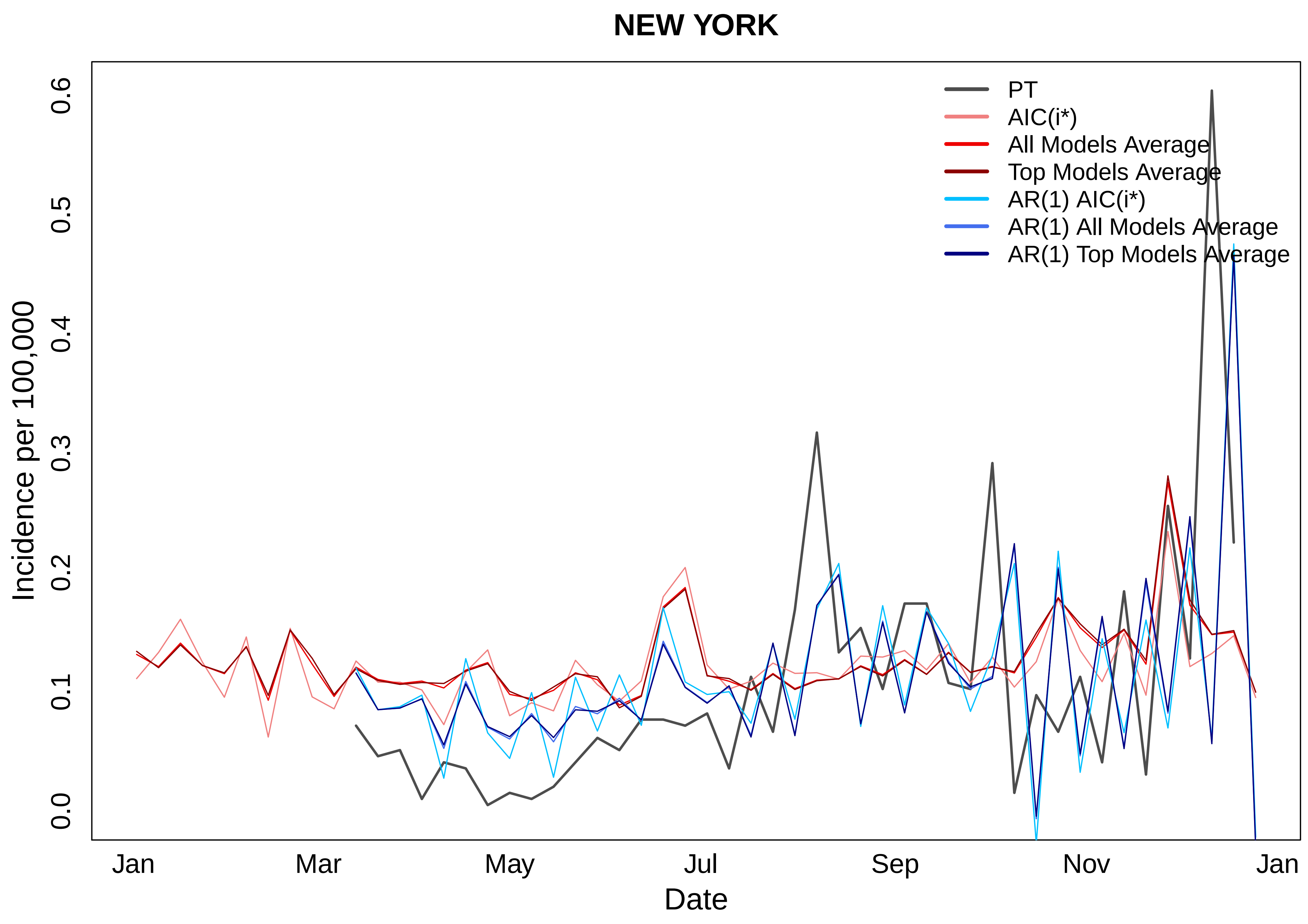

NEW YORK
PT
AIC(i*)
All Models Average
Top Models Average
AR(1) AIC(i*)
AR(1) All Models Average
AR(1) Top Models Average
Incidence per 100,000
0.0
0.1
0.2
0.3
0.4
0.5
0.6
Jan
Mar
May
Jul
Sep
Nov
Jan
Date

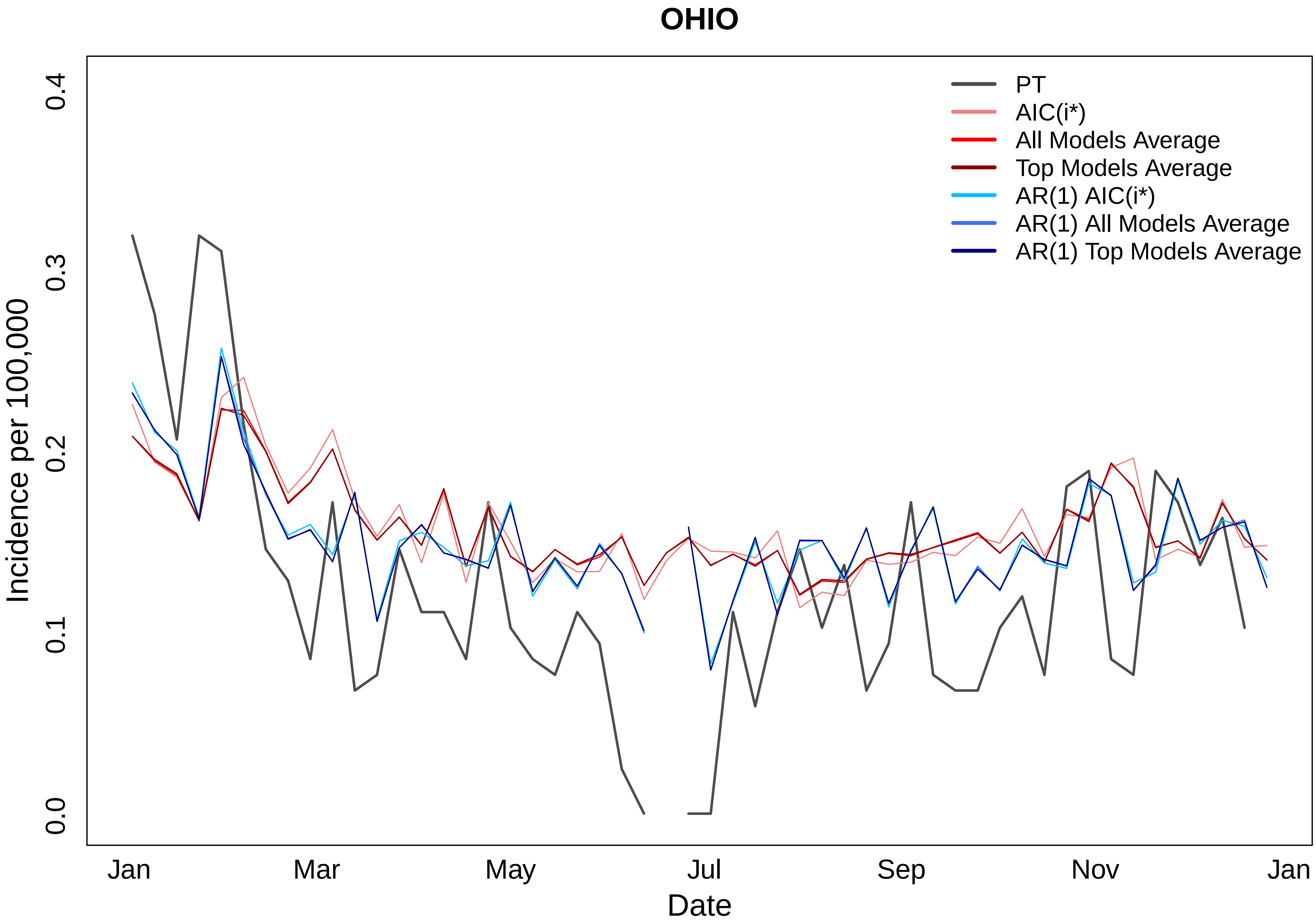

OHIO
PT
AIC(i*)
All Models Average
Top Models Average
AR(1) AIC(i*)
AR(1) All Models Average
AR(1) Top Models Average
Incidence per 100,000
0.0
0.1
0.2
0.3
0.4
Jan
Mar
May
Jul
Sep
Nov
Jan
Date

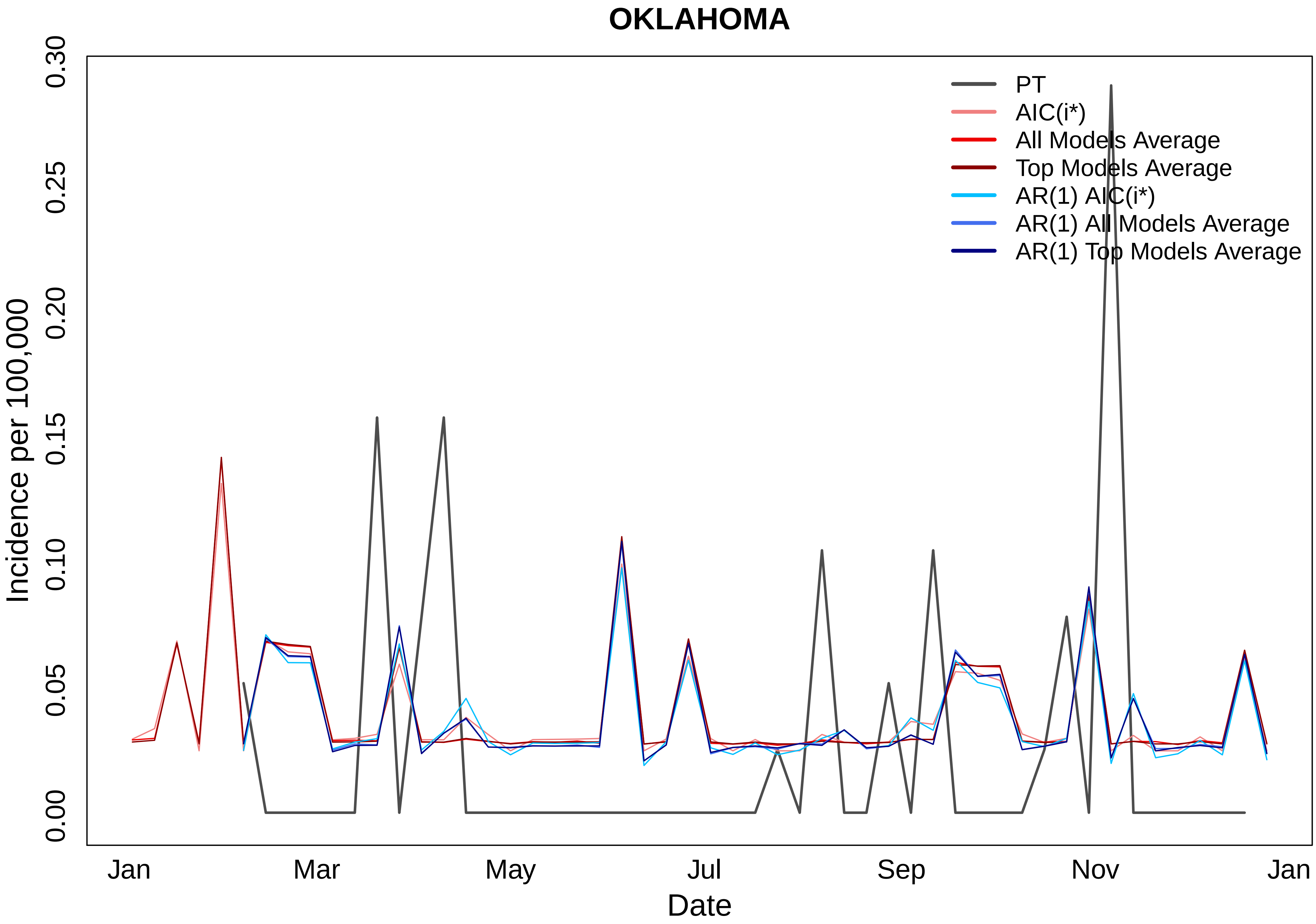

OKLAHOMA
PT
AIC(i*)
All Models Average
Top Models Average
AR(1) AIC(i*)
AR(1) All Models Average
AR(1) Top Models Average
Incidence per 100,000
0.00
0.05
0.10
0.15
0.20
0.25
0.30
Jan
Mar
May
Jul
Sep
Nov
Jan
Date

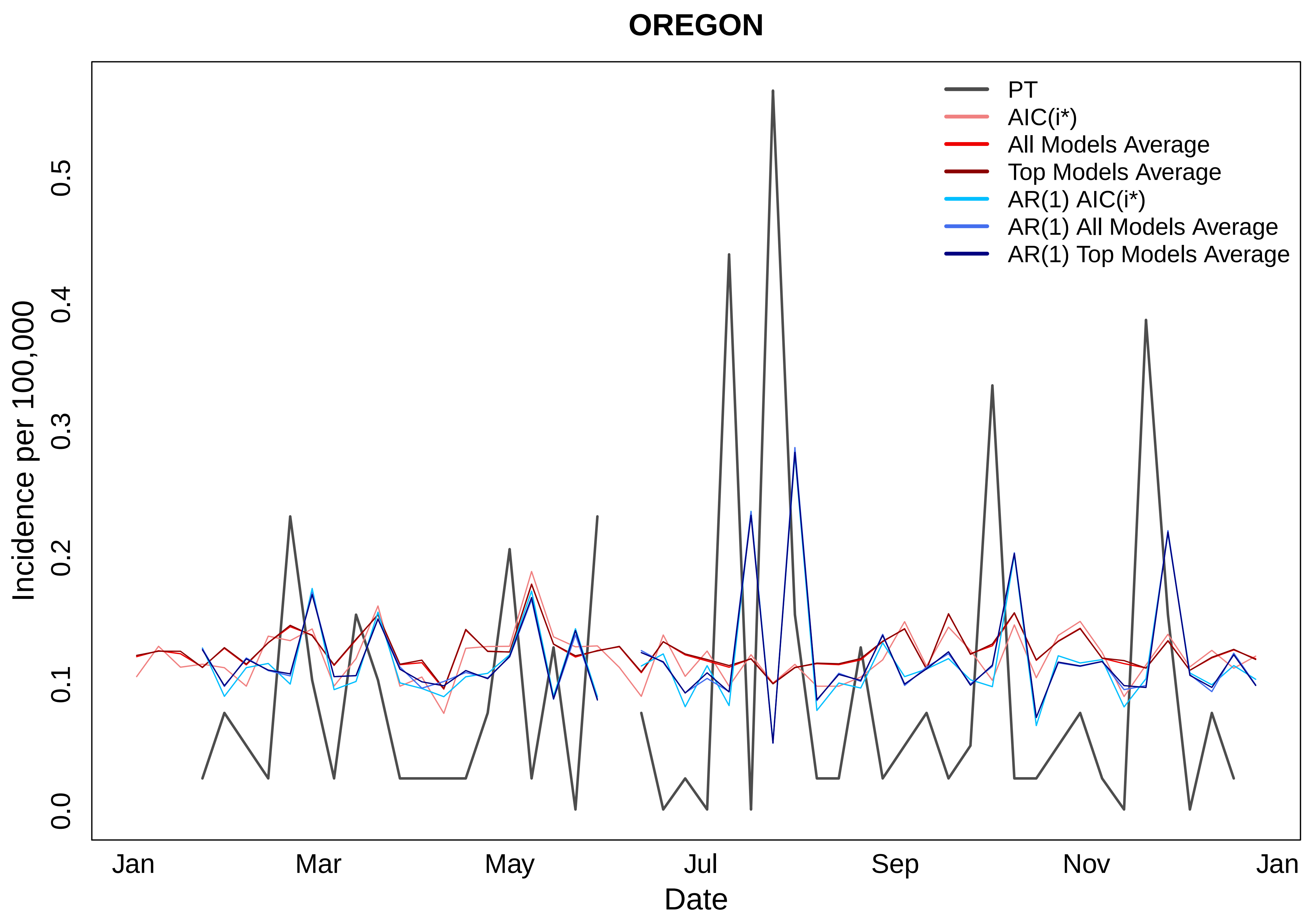

OREGON
PT
AIC(i*)
All Models Average
Top Models Average
AR(1) AIC(i*)
AR(1) All Models Average
AR(1) Top Models Average
Incidence per 100,000
0.0
0.1
0.2
0.3
0.4
0.5
Jan
Mar
May
Jul
Sep
Nov
Jan
Date

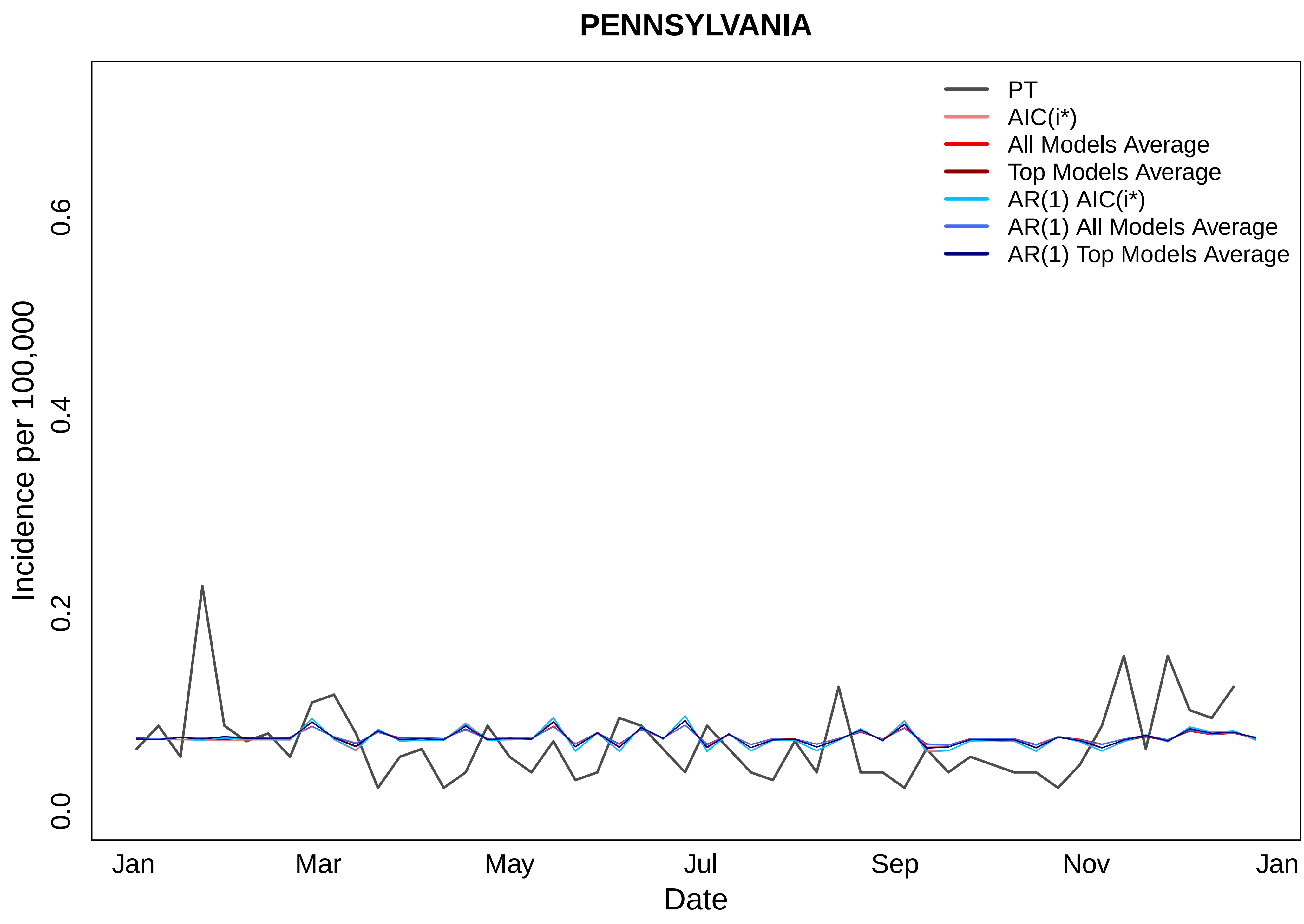
PENNSYLVANIA
PT
AIC(i*)
All Models Average
Top Models Average
AR(1) AIC(i*)
AR(1) All Models Average
AR(1) Top Models Average
Incidence per 100,000
0.0
0.2
0.4
0.6
Jan
Mar
May
Jul
Sep
Nov
Jan
Date

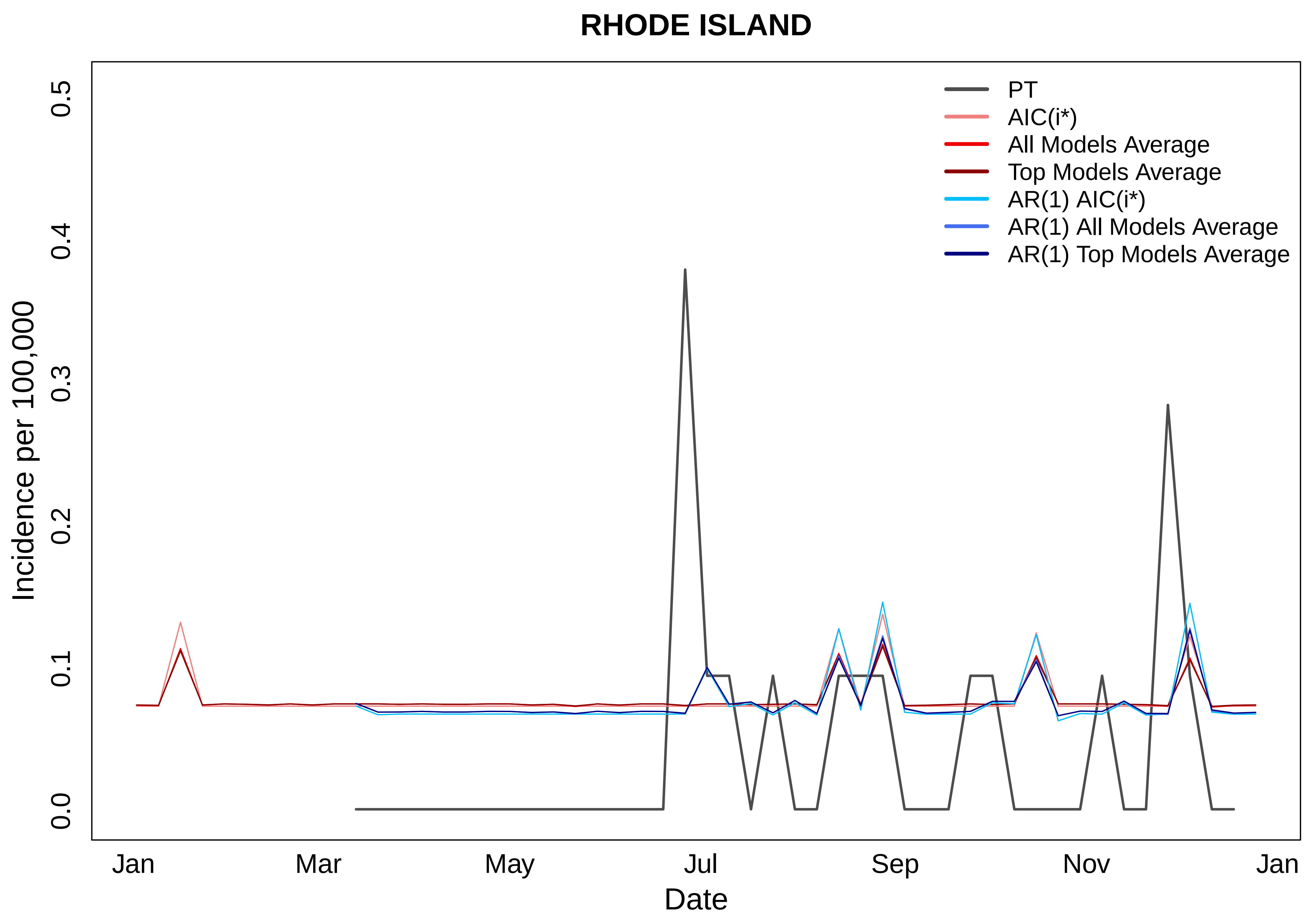
RHODE ISLAND
PT
AIC(i*)
All Models Average
Top Models Average
AR(1) AIC(i*)
AR(1) All Models Average
AR(1) Top Models Average
Incidence per 100,000
0.0
0.1
0.2
0.3
0.4
0.5
Jan
Mar
May
Jul
Sep
Nov
Jan
Date

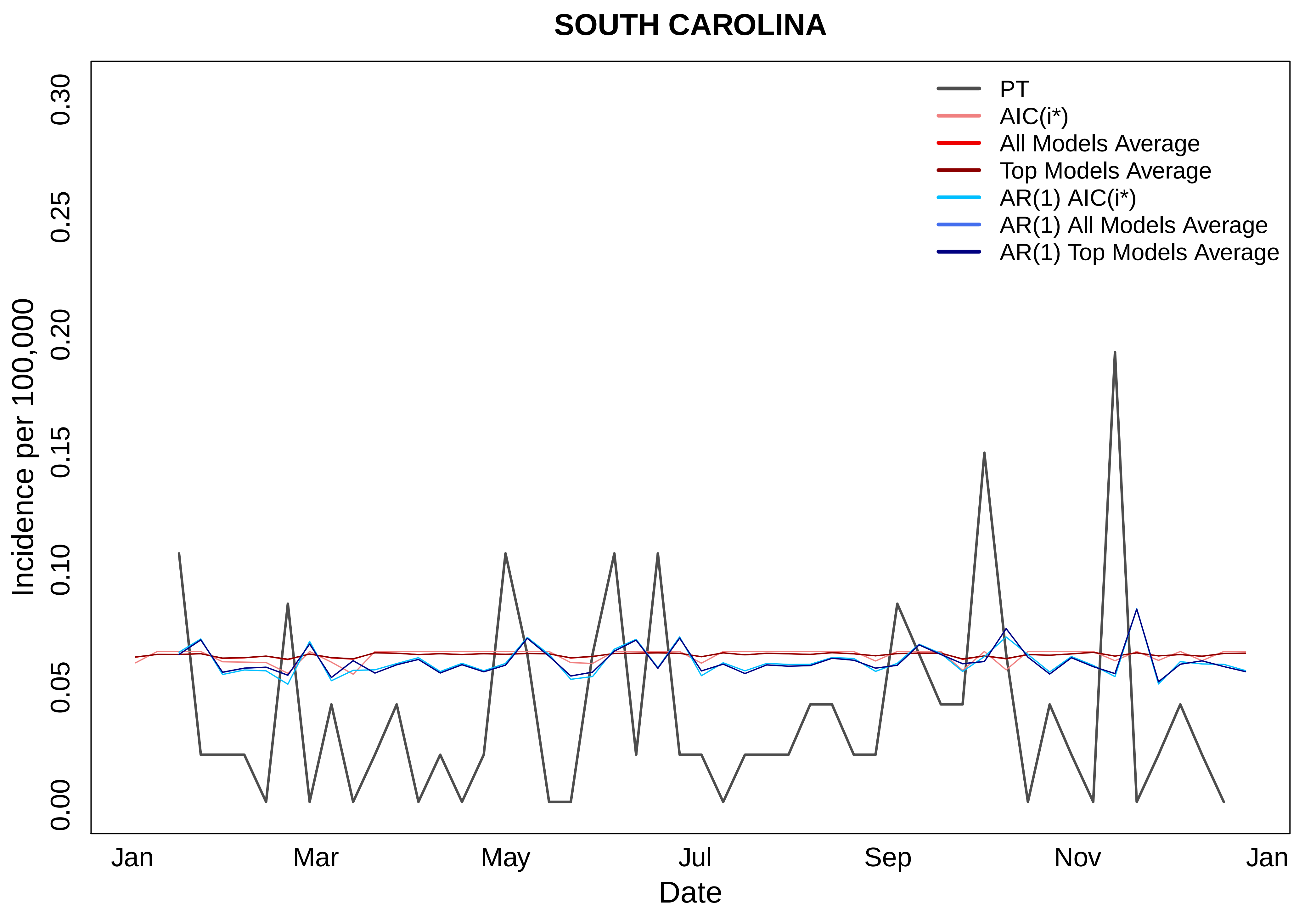
SOUTH CAROLINA
PT
AIC(i*)
All Models Average
Top Models Average
AR(1) AIC(i*)
AR(1) All Models Average
AR(1) Top Models Average
Incidence per 100,000
0.00
0.05
0.10
0.15
0.20
0.25
0.30
Jan
Mar
May
Jul
Sep
Nov
Jan
Date

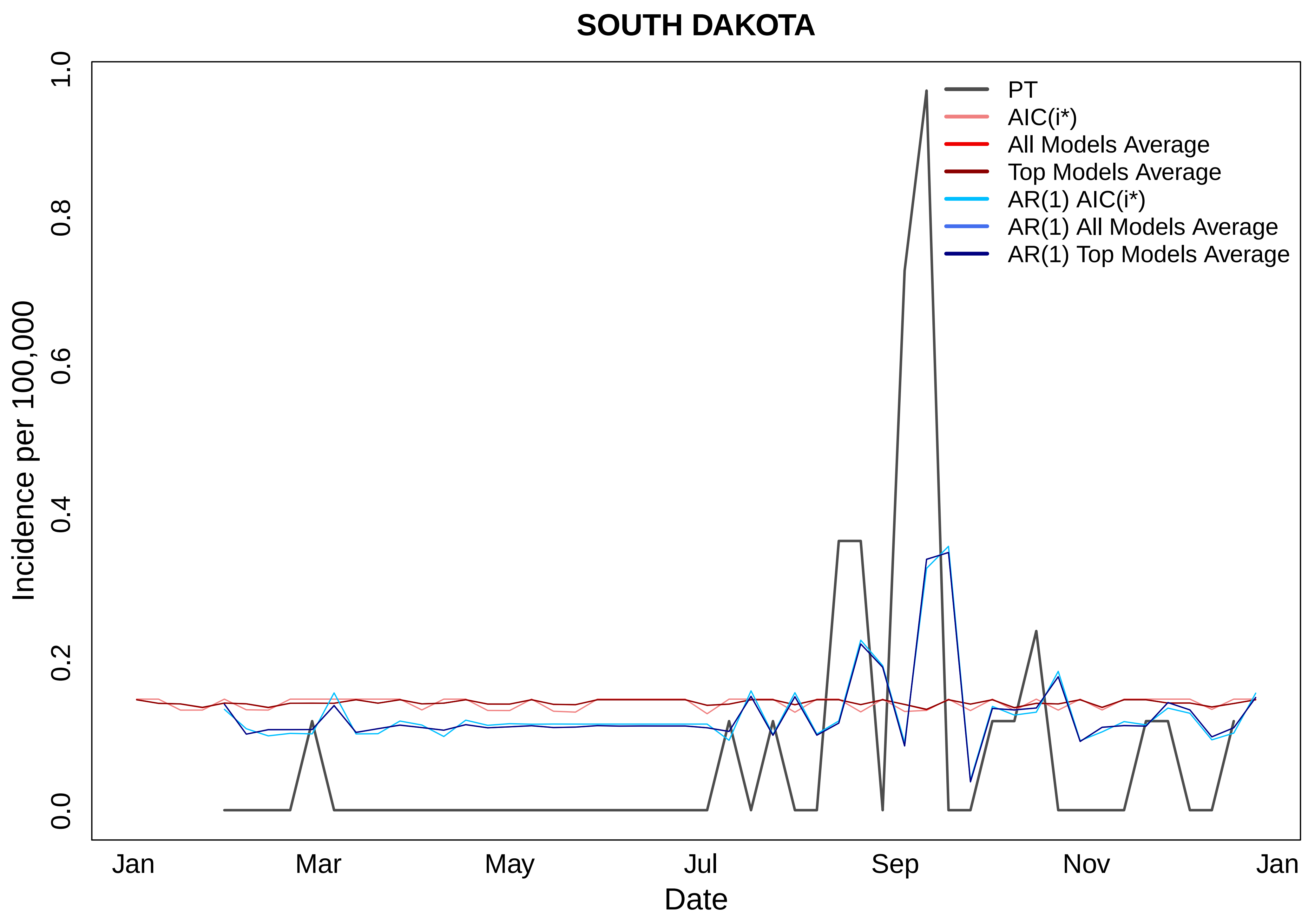
SOUTH DAKOTA
PT
AIC(i*)
All Models Average
Top Models Average
AR(1) AIC(i*)
AR(1) All Models Average
AR(1) Top Models Average
Incidence per 100,000
0.0
0.2
0.4
0.6
0.8
1.0
Jan
Mar
May
Jul
Sep
Nov
Jan
Date

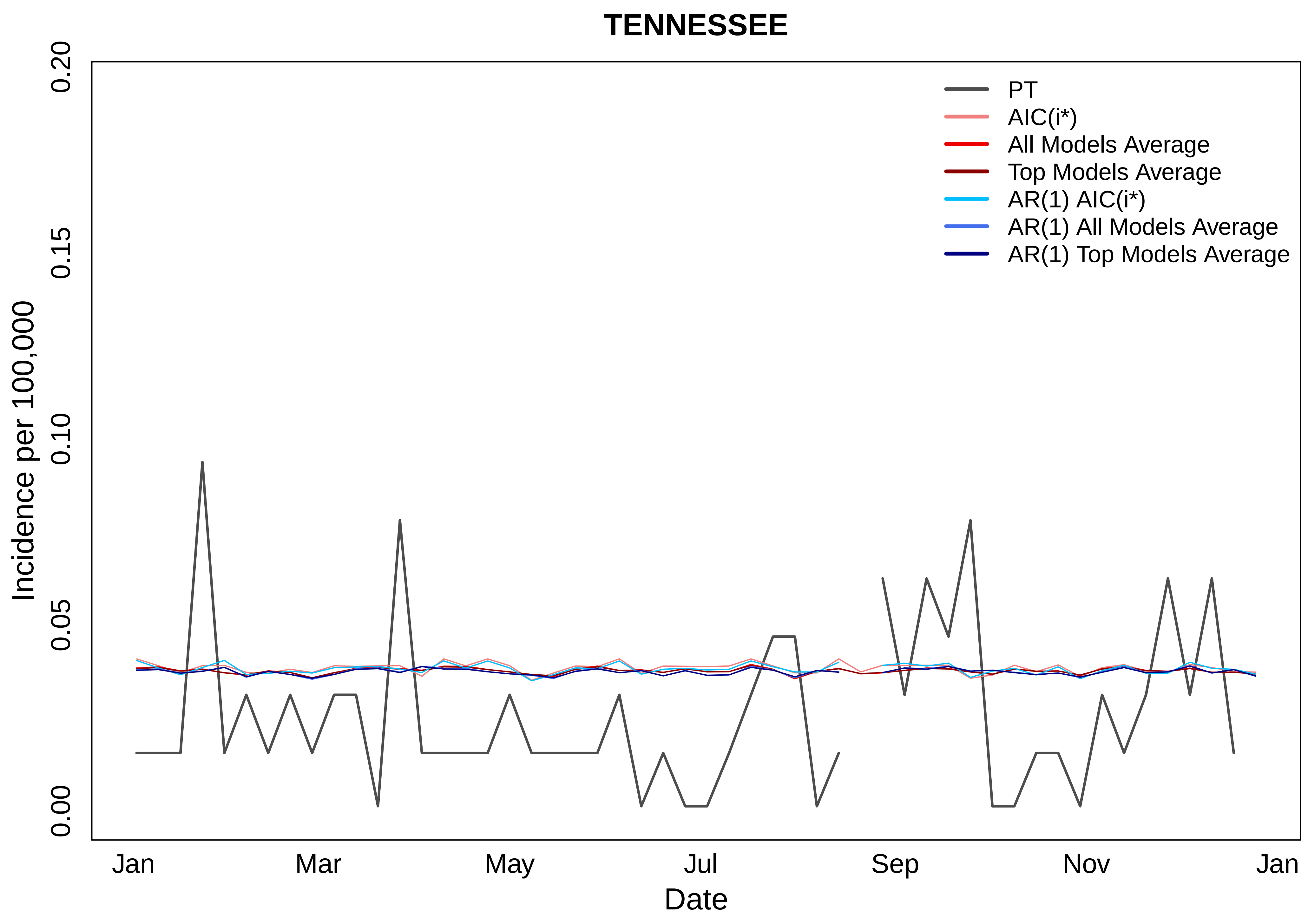

TENNESSEE
PT
AIC(i*)
All Models Average
Top Models Average
AR(1) AIC(i*)
AR(1) All Models Average
AR(1) Top Models Average
Incidence per 100,000
0.00
0.05
0.10
0.15
0.20
Jan
Mar
May
Jul
Sep
Nov
Jan
Date

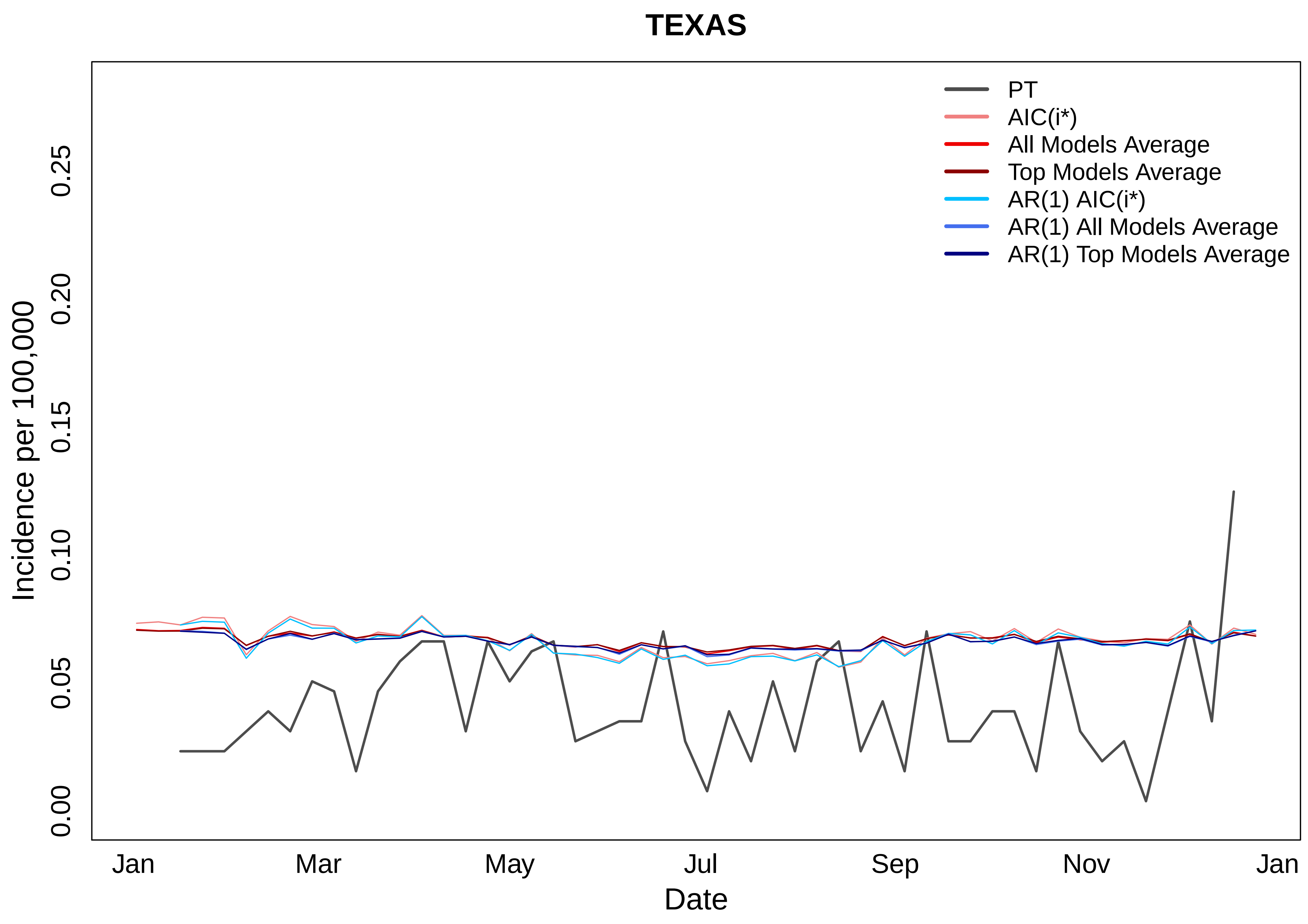

TEXAS
PT
AIC(i*)
All Models Average
Top Models Average
AR(1) AIC(i*)
AR(1) All Models Average
AR(1) Top Models Average
Incidence per 100,000
0.00
0.05
0.10
0.15
0.20
0.25
Jan
Mar
May
Jul
Sep
Nov
Jan
Date

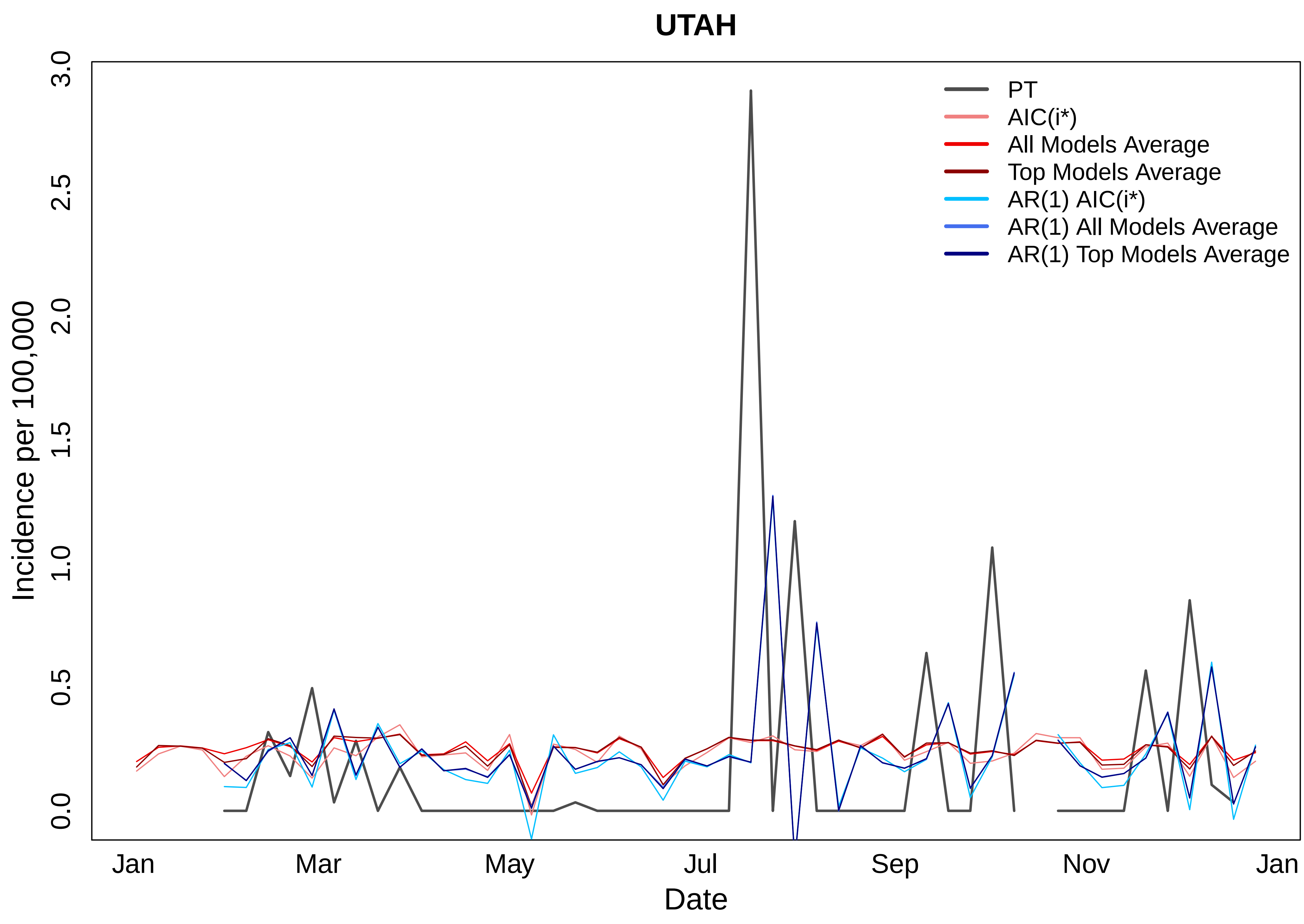

UTAH
PT
AIC(i*)
All Models Average
Top Models Average
AR(1) AIC(i*)
AR(1) All Models Average
AR(1) Top Models Average
Incidence per 100,000
0.0
0.5
1.0
1.5
2.0
2.5
3.0
Jan
Mar
May
Jul
Sep
Nov
Jan
Date

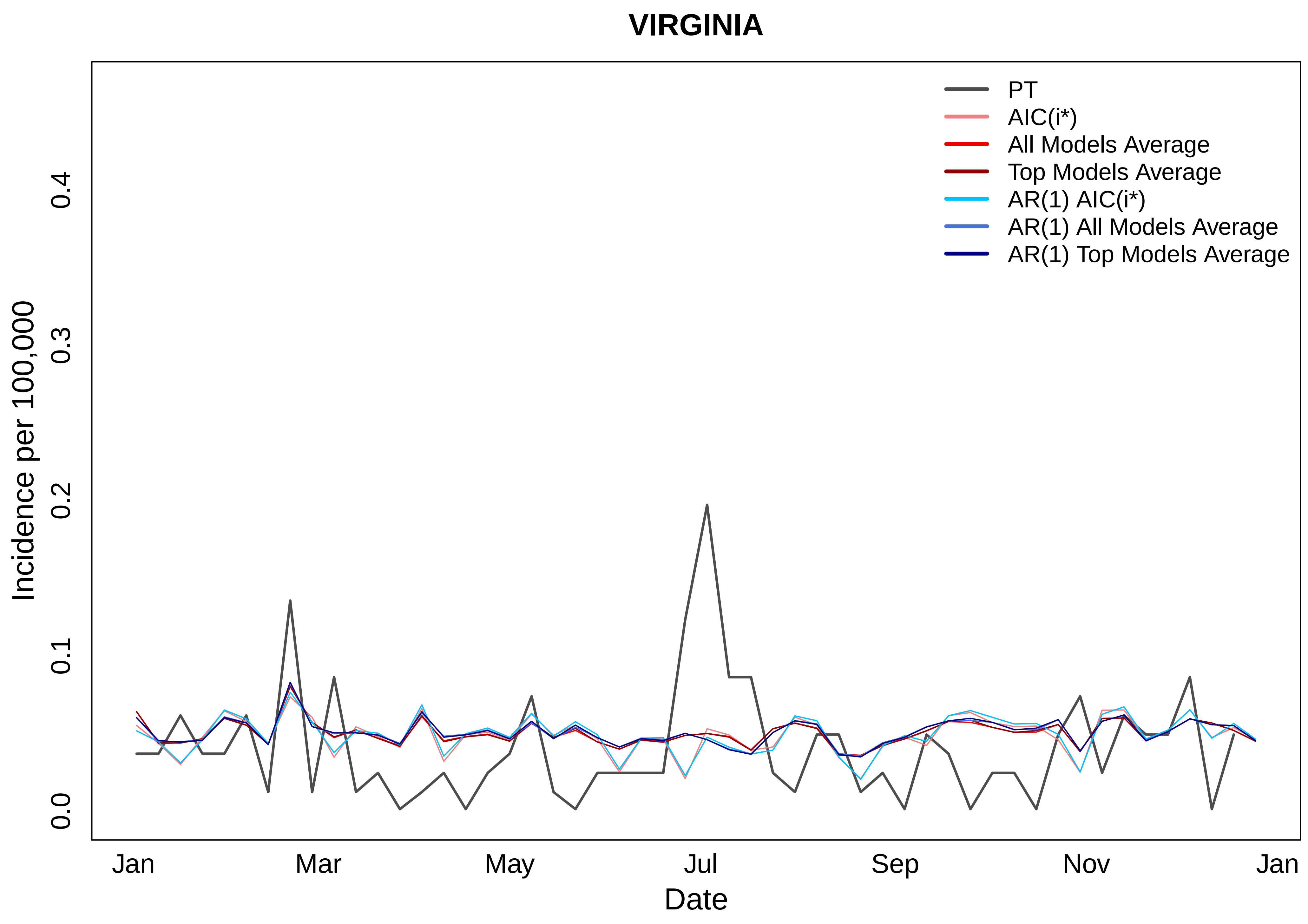
VIRGINIA
PT
AIC(i*)
All Models Average
Top Models Average
AR(1) AIC(i*)
AR(1) All Models Average
AR(1) Top Models Average
Incidence per 100,000
0.0
0.1
0.2
0.3
0.4
Jan
Mar
May
Jul
Sep
Nov
Jan
Date

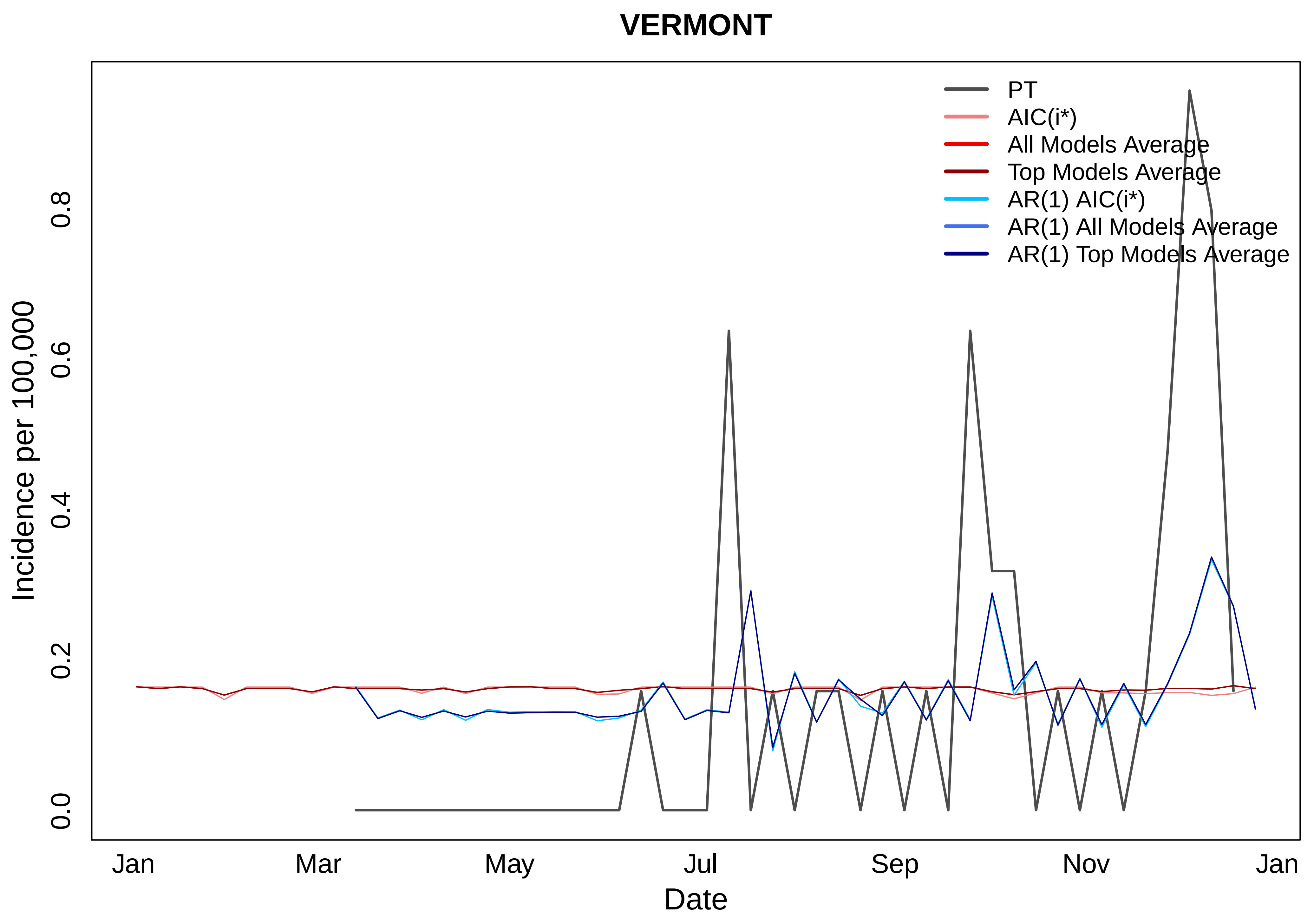
VERMONT
PT
AIC(i*)
All Models Average
Top Models Average
AR(1) AIC(i*)
AR(1) All Models Average
AR(1) Top Models Average
Incidence per 100,000
0.0
0.2
0.4
0.6
0.8
Jan
Mar
May
Jul
Sep
Nov
Jan
Date

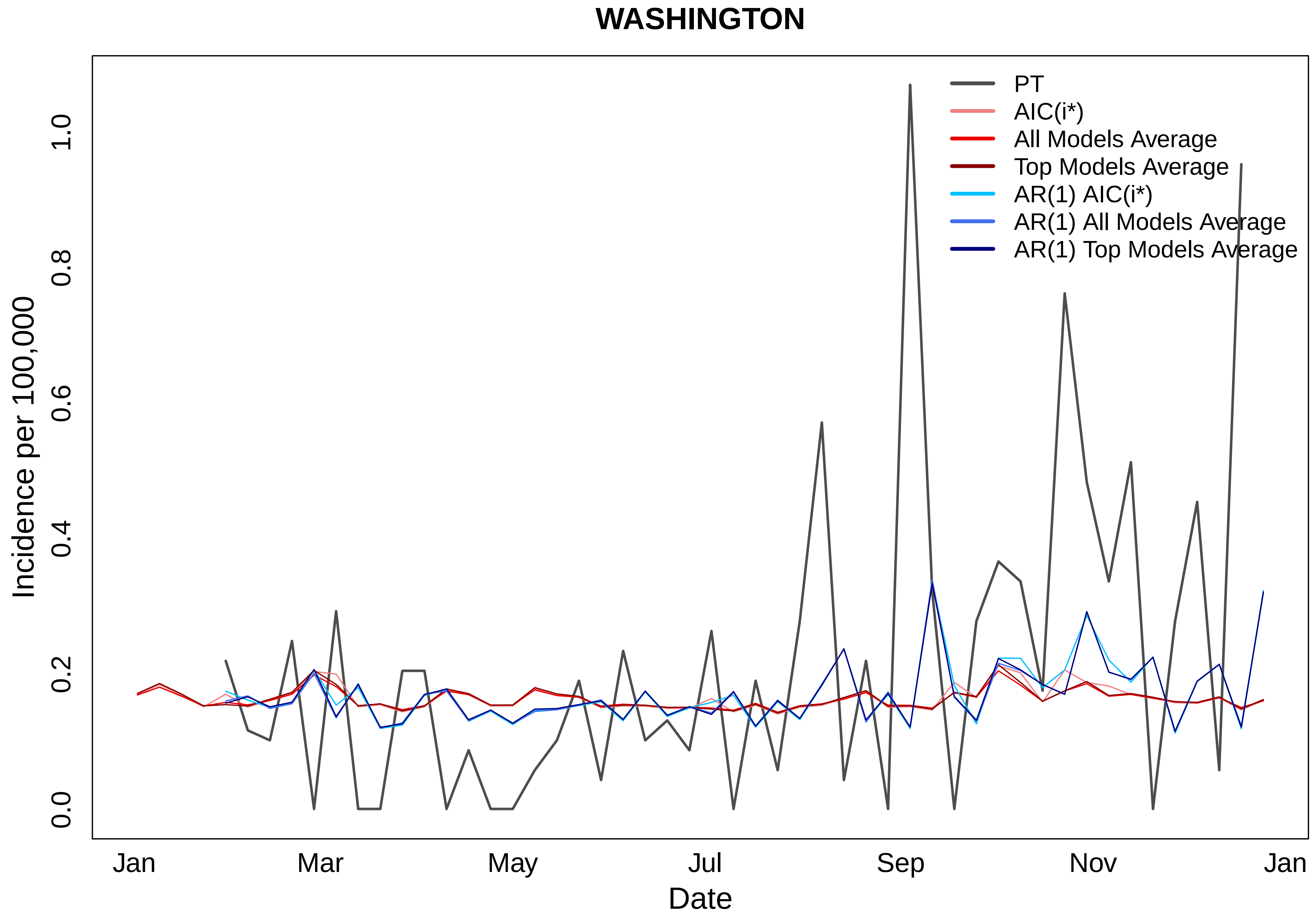

WASHINGTON
PT
AIC(i*)
All Models Average
Top Models Average
AR(1) AIC(i*)
AR(1) All Models Average
AR(1) Top Models Average
Incidence per 100,000
0.0
0.2
0.4
0.6
0.8
1.0
Jan
Mar
May
Jul
Sep
Nov
Jan
Date

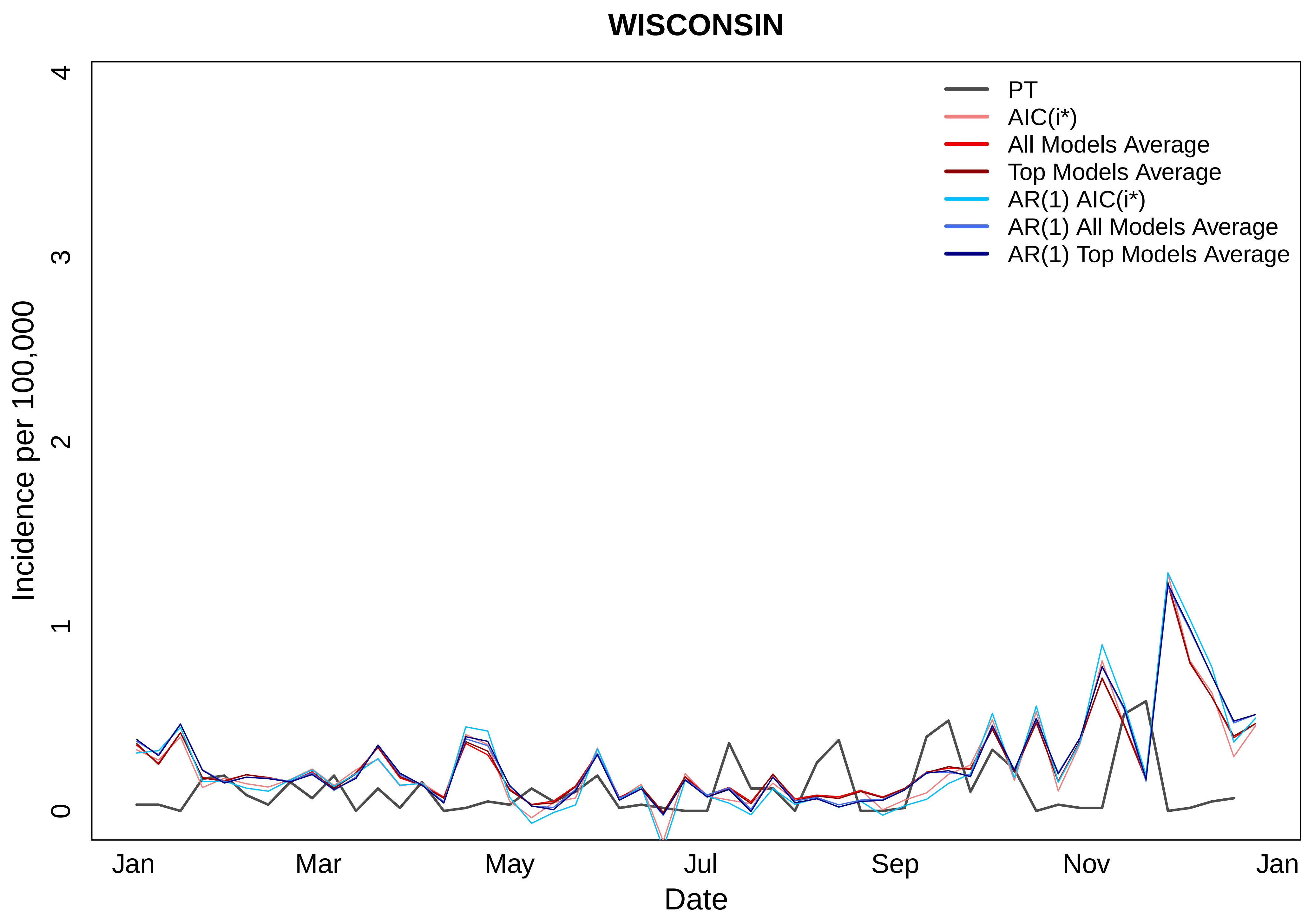

WISCONSIN
PT
AIC(i*)
All Models Average
Top Models Average
AR(1) AIC(i*)
AR(1) All Models Average
AR(1) Top Models Average
Incidence per 100,000
0
1
2
3
4
Jan
Mar
May
Jul
Sep
Nov
Jan
Date

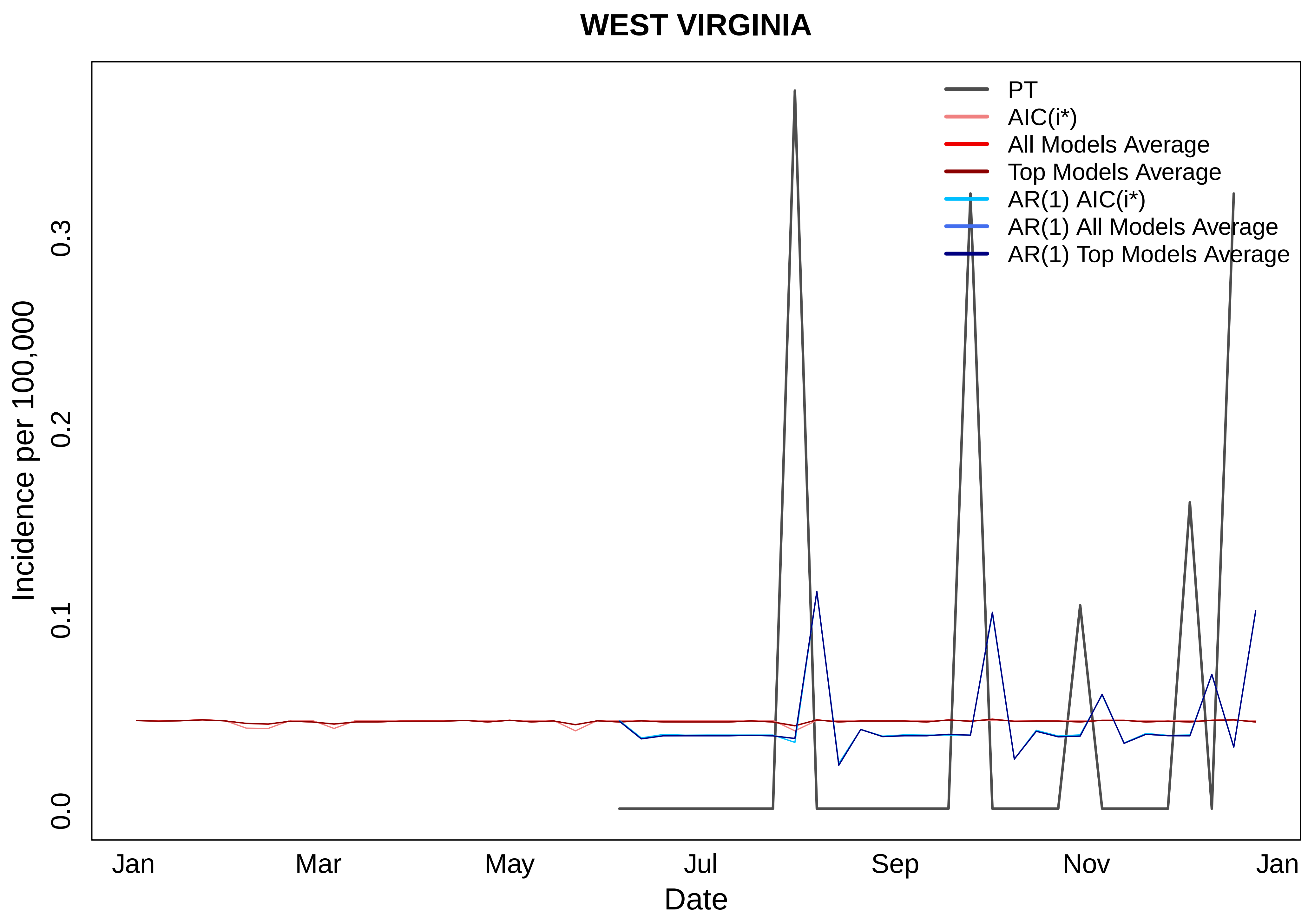

WEST VIRGINIA
PT
AIC(i*)
All Models Average
Top Models Average
AR(1) AIC(i*)
AR(1) All Models Average
AR(1) Top Models Average
Incidence per 100,000
0.0
0.1
0.2
0.3
Jan
Mar
May
Jul
Sep
Nov
Jan
Date

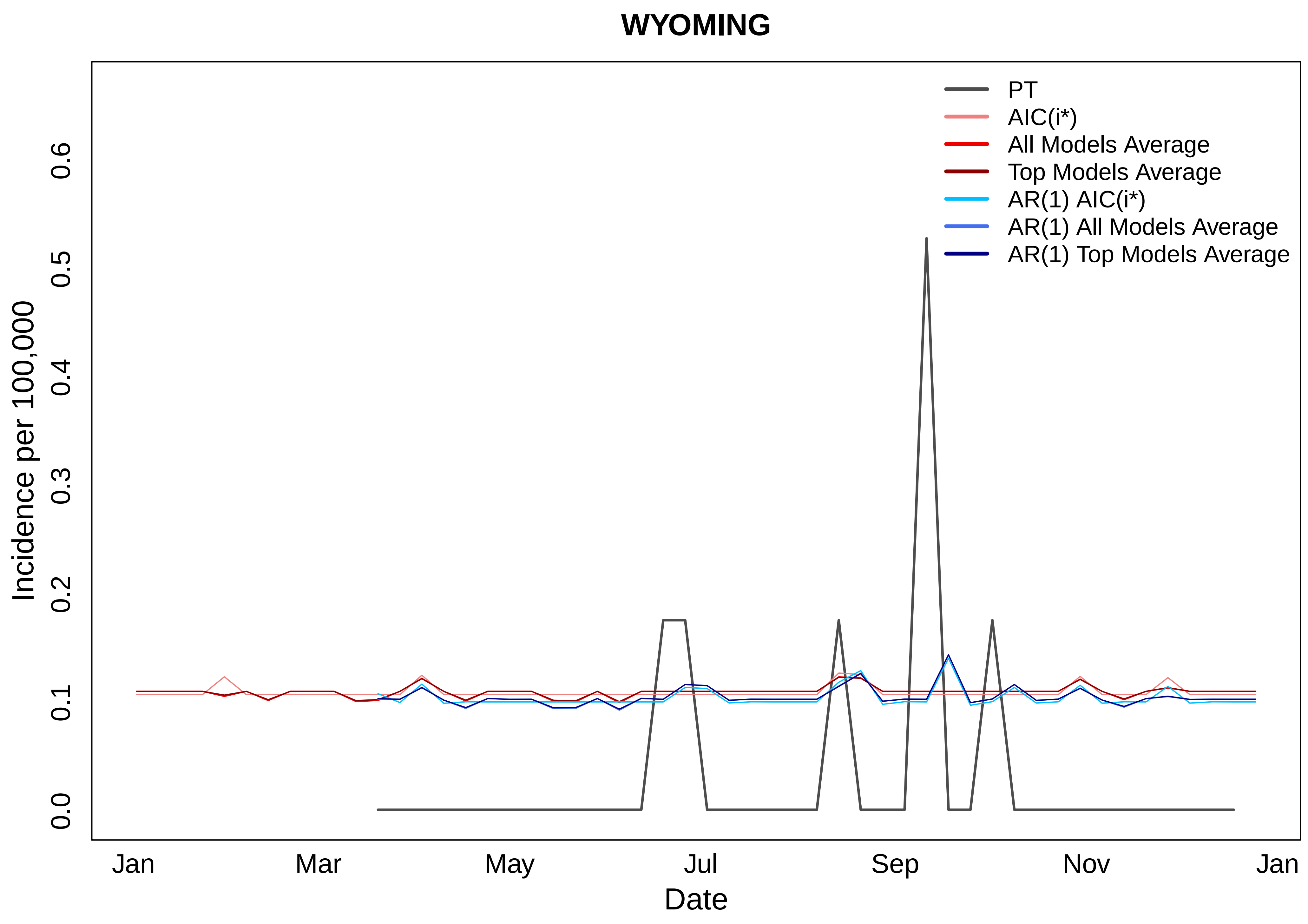
WYOMING
PT
AIC(i*)
All Models Average
Top Models Average
AR(1) AIC(i*)
AR(1) All Models Average
AR(1) Top Models Average
Incidence per 100,000
0.0
0.1
0.2
0.3
0.4
0.5
0.6
Jan
Mar
May
Jul
Sep
Nov
Jan
Date
